# Toripalimab plus chemotherapy for first line treatment of advanced non-small cell lung cancer (CHOICE-01): final OS and biomarker exploration of a randomized, double-blind, phase 3 trial

**DOI:** 10.1038/s41392-024-02087-6

**Published:** 2024-12-24

**Authors:** Jia Zhong, Kailun Fei, Lin Wu, Baolan Li, Zhijie Wang, Ying Cheng, Xiaoling Li, Xicheng Wang, Liang Han, Xiaohong Wu, Yun Fan, Yan Yu, Dongqing Lv, Jianhua Shi, Jianjin Huang, Shaozhang Zhou, Baohui Han, Guogui Sun, Qisen Guo, Youxin Ji, Xiaoli Zhu, Sheng Hu, Wei Zhang, Qiming Wang, Yuming Jia, Ziping Wang, Yong Song, Jingxun Wu, Meiqi Shi, Xingya Li, Zhigang Han, Yunpeng Liu, Zhuang Yu, An-Wen Liu, Xiuwen Wang, Caicun Zhou, Diansheng Zhong, Liyun Miao, Zhihong Zhang, Hui Zhao, Jun Yang, Dong Wang, Yingyi Wang, Qiang Li, Xiaodong Zhang, Mei Ji, Zhenzhou Yang, Jiuwei Cui, Beili Gao, Buhai Wang, Hu Liu, Lei Nie, Mei He, Shi Jin, Wei Gu, Yongqian Shu, Tong Zhou, Jian Feng, Xinmei Yang, Cheng Huang, Bo Zhu, Yu Yao, Sheng Yao, Jianjun Yu, Shang li Cai, Yiran Cai, Jiachen Xu, Wei Zhuang, Xianmin Luo, Jianchun Duan, Jie Wang

**Affiliations:** 1 State Key Laboratory of Molecular Oncology, CAMS Key Laboratory of Translational Research on Lung Cancer, Department of Medical Oncology, National Cancer Center/National Clinical Research Center for Cancer/Cancer Hospital, Chinese Academy of Medical Sciences and Peking Union Medical College, Beijing, China; 2 Hunan Cancer Hospital, The Affiliated Cancer Hospital of Xiangya School of Medicine, Central South University, Changsha, China; 3 Beijing Chest Hospital, Capital Medical University, Beijing, China; 4 Jilin Cancer Hospital, Changchun, China; 5 Liaoning Cancer Hospital & Institute, Shenyang, China; 6 The First Affiliated Hospital, School of Clinical Medicine of Guangdong Pharmaceutical University, Guangzhou, China; 7 Xuzhou Central Hospital, Xuzhou, China; 8 Jiangnan University Affiliated Hospital, Wuxi, China; 9 Cancer Hospital of the University of Chinese Academy of Sciences, Hangzhou, China; 10 Harbin Medical University Cancer Hospital, Harbin, China; 11 Taizhou Hospital of Zhejiang Province, Linhai, China; 12 Linyi Cancer Hospital, Linyi, China; 13 The Second Affiliated Hospital of Zhejiang University School of Medicine, Hangzhou, China; 14 Guangxi Medical University Affiliated Tumor Hospital, Nanning, China; 15 Shanghai Chest Hospital, Shanghai, China; 16 Tangshan People’s Hospital, Tangshan, China; 17 Shangdong Cancer Hospital, Jinan, China; 18 Qingdao Central Hospital, Qingdao, China; 19 Zhongda Hospital Southeast University, Nanjing, China; 20 Hubei Cancer Hospital, Wuhan, China; 21 The First Affiliated Hospital of Nanchang University, Nanchang, China; 22 Henan Cancer Hospital, Zhengzhou, China; 23 The Second People’s Hospital of Yibin, Yibin, China; 24 Peking University Cancer Hospital, Beijing, China; 25 Jinling Hospital, Nanjing University School of Medicine, Nanjing, China; 26 The First Affiliated Hospital of Xiamen University, Xiamen, China; 27 Jiangsu Cancer Hospital, Nanjing, China; 28 The First Affiliated Hospital of Zhengzhou University, Zhengzhou, China; 29 Affiliated Tumor Hospital of Xinjiang Medical University, Urumqi, China; 30 The First Hospital of China Medical University, Shenyang, China; 31 The Affiliated Hospital of Qingdao University, Qingdao, China; 32 The Second Affiliated Hospital of Nanchang University, Nanchang, China; 33 Qilu Hospital of Shandong University, Jinan, China; 34 Shanghai Pulmonary Hospital, Shanghai, China; 35 Tianjin Medical University General Hospital, Tianjin, China; 36 Affiliated Drum Tower Hospital, Medical School of Nanjing University, Nanjing, China; 37 Anhui Provincial Cancer Hospital, Hefei, China; 38 The Second Hospital of Anhui Medical University, Hefei, China; 39 The Second Affiliated Hospital of Guangzhou Medical University, Guangzhou, China; 40 Army Medical Center of PLA, Daping Hospital, Daping, China; 41 Peking Union Medical College Hospital, Beijing, China; 42 Shanghai East Hospital of Tongji University, Shanghai, China; 43 Nantong Tumor Hospital, Nantong, China; 44 The First People’s Hospital of Changzhou, Changzhou, China; 45 The Second Affiliated Hospital of Chongqing University, Chongqing, China; 46 The First Hospital of Jilin University, Jilin, China; 47 Ruijin Hospital Shanghai Jiaotong University, School of Medicine, Shanghai, China; 48 Subei People’s Hospital of Jiangsu Province, Yanghzou, China; 49 Shaanxi Provincial Cancer Hospital, Xian, China; 50 Shanxi Provincial People’s Hospital, Taiyuan, China; 51 Cancer Hospital of Chinese Academy of Medical Sciences, Shenzhen Center, Shenzhen, China; 52 Nanjing First Hospital, Nanjing, China; 53 The First Affiliated Hospital of Nanjing Medical University, Nanjing, China; 54 ChangZhou Cancer Hospital, Changzhou, China; 55 Affiliated Hospital of Nantong University, Nantong, China; 56 Jiaxing No. 1 Hospital, Jiaxing, China; 57 Fujian Cancer Hospital, Fuzhou, China; 58 Xinqiao Hospital of Army Medical University, Chongqing, China; 59 First Affiliated Hospital of Xi’an Jiaotong University, Xian, China; 60 TopAlliance Biosciences, Rockville, MD USA; 61 Shanghai Junshi Biosciences, Shanghai, China; 62 Burning Rock Biotech, Guangdong, China; 63 Department of Respiratory Medicine, Shanxi Province Cancer Hospital/Shanxi Hospital Affiliated to Cancer Hospital, Chinese Academy of Medical Sciences/Cancer Hospital Affiliated to Shanxi Medical University, Taiyuan, Shanxi China

**Keywords:** Lung cancer, Predictive markers, Tumour biomarkers

## Abstract

A randomized double-blind phase 3 trial (CHOICE-01, NCT03856411) demonstrated that combining toripalimab with chemotherapy substantially improves progression-free survival (PFS) in advanced non-small cell lung cancer (NSCLC) patients without pretreatment. This study presents the prespecified final analysis of overall survival (OS) and biomarkers utilizing circulating tumor DNA (ctDNA) and tissue-based sequencing. Additionally, the analysis revealed a higher median overall survival (OS, 23.8 months) in the toripalimab group than that in the control group (17.0 months). (HR = 0.69, 95%CI: 0.57–0.93, nominal *P* = 0.01). This survival benefit was particularly notable in the non-squamous subgroup. As the first phase 3 study to perform both baseline tissue whole-exome sequencing (WES) and peripheral blood ctDNA testing, we investigated efficacy predictive biomarkers based on both tissue and ctDNA, Genomic sequencing of ctDNA showed high concordance with tumor tissue independently confirmed that individuals exhibiting a high tumor mutational burden, as well as mutations in the *FA-PI3K-Akt* and *IL-7* signaling pathways benefited more from the toripalimab treatment. Furthermore, a ctDNA response observed on cycle 3 day 1, was associated with improved clinical outcomes for patients treated with the combination therapy. In conclusion, Toripalimab plus chemotherapy yields significant improvements in OS as a first-line treatment. The study highlights the utility of ctDNA as a proxy for tumor tissue, providing novel prospects for predicting efficacy of immuno-chemotherapy through continuous ctDNA monitoring.

## Introduction

Worldwide cancer mortality statistics continue to identify non-small cell lung cancer (NSCLC) as the predominant death caused by cancer.^1^ Recent therapeutic advances have centered on immune checkpoint blockade, particularly antibodies designed to target the PD-1 receptor and its corresponding ligand PD-L1. These immune checkpoint inhibitors (ICIs), have revolutionized treatment for advanced NSCLC, particularly in patients lacking driver mutations.^2–4^ PD-1/PD-L1 inhibitors have also been extended to first-line clinical applications, marking a significant advancement in NSCLC treatment paradigms. A serious of clinical trials demonstrated that PD-1/PD-L1 inhibitors combined with standard platinum-based chemotherapy significantly improved progression-free survival (PFS) compared with chemotherapy alone.^2–4^ Albeit, besides Keynote 189^5^ and 407 studies,^6^ most phase 3 clinical trials comparing chemotherapy versus ICIs combination approaches for treatment-naive NSCLC treatment designate overall survival as a secondary endpoint, with many lacking comprehensive long-term survival data.

The combination of PD-1/PD-L1 antibodies with chemotherapy is more effective than the PD-1 or PD-L1 monoclonal antibody monotherapy or chemotherapy alone for a broader patient population. However, it is still necessary to develop biomarkers to guide immuno-chemotherapy treatment decisions. The predictive value of PD-L1 expression and tumor mutational burden (TMB) mainly focuse on PD-1 and/or PD-L1 monotherapy.^7^ Notably, both PD-L1 expression^8^ and TMB^9^ has limited predictive value for the efficacy of combined chemotherapy and immunotherapy. Tumor immune-related gene expression signature and NRF2 pathway activation has been associated with efficacy of PD-1inhibitor combined with chemotherapy in lung caner.^9^Up to now, the above predictive biomarkers are tumor tissue-based. Blood-based liquid biopsy has become as an alternative, enabling the detection of circulating tumor DNA (ctDNA) and through peripheral blood analysis. The relationship between tissue-based whole-exome sequencing (WES) and ctDNA sequencing has been examined, particularly for estimating TMB.^10^ However, there remains no prospective sample collection and validation through phase 3 randomized clinical trials to assess the consistency of efficacy-predictive biomarkers derived from ctDNA and those established via tissue-based WES. This gap limits the use of ctDNA as a substitute for tissue in predicting clinical outcomes.

Given the heterogeneous patterns of somatic mutation and distinct immune landscape between lung adenocarcinoma and squamous carcinoma^11,12^ as well as treatment-response diversity between squamous and non-squamous NSCLC, most studies investigate these pathological subtypes separately. Studies involving both NSCLC pathological subtypes, with a uniform regimen after randomization, are more conductive to investigating the differences between the two subtypes.

Toriplimab, a humanized IgG4 monoclonal antibody targeting PD-1, is the first domestic PD-1 mono-antibody approved in China.^13^ Toriplimab has demonstrated efficacy in melanoma,^13^ lung cancer,^14,15^ esophageal squamous cell carcinoma,^16^ urothial carcinoma,^17^ and nasopharyngeal carcinoma.^18^ The CHOICE-01study, is a phase-3 clinical trial, evaluated the clinical impact of toripalimab, an engineered IgG4 monoclonal antibody targeting PD-1, when used in conjunction with chemotherapy for previously untreated NSCLC patients. This study includes both squamous and non-squamous NSCLC subtype. This provides valuable exploratory insights into the timing of immune checkpoint inhibitor use for patients with different pathological subtypes. At the interim analysis, significant improvements were observed in both progression-free survival (PFS) and overall survival (OS), several efficacy predictive biomarkers based on tissue-based WES analysis were also explored.^15^ This trial also finds stratification strategy as predictive marker for chemotherapy plus PD-1 inhibitor based on dynamic monitoring of ctDNA.^19^

Here we report the final OS analysis from the CHOICE-01 study, along with the subgroups OS analysis among different pathological or PD-L1 subtypes. Updated biomarkers that integrate tissue-based and liquid biopsy sequencing results. This study report concordance of biomarkers analysis based on tissue and ctDNA, and addressed the gap in validating the heterogeneity of efficacy-predictive biomarkers derived from ctDNA versus tissue-based WES.

## Results

**Fig. 1:**
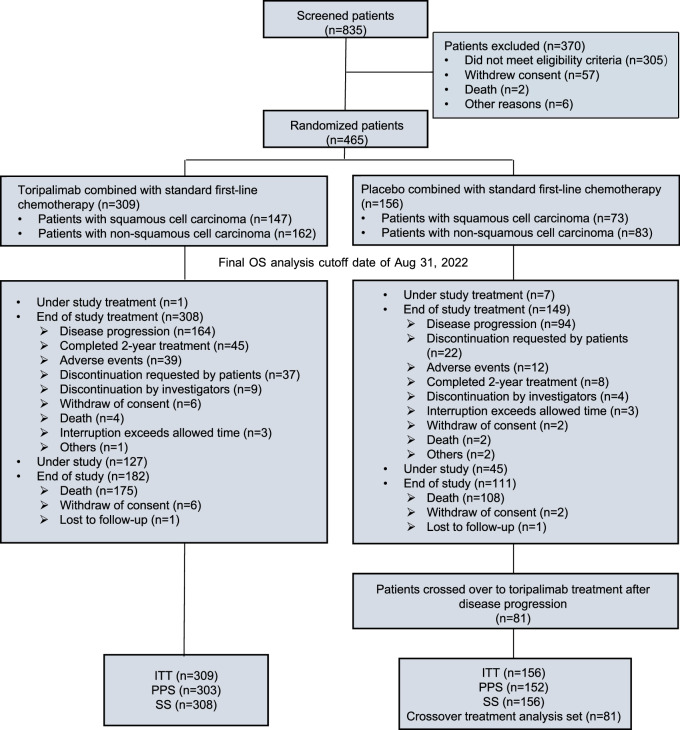
CONSORT diagram of the CHOICE-01 study. Between April 2, 2019 and August 5, 2020, 835 patients with advanced non-small-cell lung cancer were screened for eligibility from 59 centers across China. 465 were enrolled and randomly assigned in a 2:1 ratio to the toripalimab plus standard first-line chemotherapy group (*n* = 309) or the placebo plus standard first-line chemotherapy group (*n* = 156). By the cutoff date of August 31, 2022, one patient from the toripalimab group and seven patients from the placebo group were still under study treatment. Among the patients who ended study treatment, 127 patients from the toripalimab group and 45 patients from the placebo group were still under study. Among patients in the placebo group, 81 crossed over to toripalimab treatment after disease progression. ITT, intention-to-treat; PPS, per protocol set; SS, safety set

Between 2 April 2019 and 5 August 2020, 465 patients were randomized in a 2:1 ratio to either the toripalimab group (*n* = 309) or the placebo group (*n* = 156). Stratification was performed based on PD-L1 expression status, histological classification (SCC vs. non-SCC), and smoking status (Fig. 1). Baseline demographic and disease characteristics were evenly distributed across both treatment groups (Table 1).

**Table 1:** Summary of patient demographics

	Toripalimab+ Chemo *N* = 309	Placebo + Chemo *N* = 156
Age		
Median Age	63	61
(Range) years	(36–75)	(29–75)
≥65 years	130 (42.1)	55 (35.3)
Gender, *N* (%)		
Male	247 (79.9)	130 (83.3)
ECOG, PS, *N* (%)		
0	66 (21.4)	36 (23.1)
1	243 (78.6)	120 (76.9)
PD-L1 Expressions^a^, *N* (%)		
TC < 1%	98 (31.7)	41 (26.3)
1% ≤ TC <50%	128 (41.4)	75 (48.1)
TC ≥ 50%	72 (23.3)	28 (17.9)
Not evaluable	11 (3.6)	12 (7.7)
Histology		
Non-squamous	162 (52.4)	83 (53.2)
Squamous	147 (47.6)	73 (46.8)
Smoking Status, *N* (%)		
Regular	213 (68.9)	107 (68.6)
Occasional or none	96 (31.1)	49 (31.4)
Clinical Stage at Study Entry, N (%)		
IIIB/IIIC	49 (15.9)	23 (14.7)
IVA/IVB	260 (84.1)	133 (85.3)
Sites of Metastases, *N* (%)		
Brain Metastases	5 (1.6)	0
Hepatic Metastases	26 (8.4)	14 (9.0)
≥3 Sites	53 (17.2)	24 (15.4)
Prior Therapy, *N* (%)		
Neoadjuvant/ Adjuvant	13 (4.2)	9 (5.8)
Prior Surgery	28 (9.1)	18 (11.5)
Radiation	11 (3.6)	1(0.6)
Tumor Tissue TMB		
≥ 10 mutations/Mb	77 (24.9)	45 (28.8)
<10 mutations/Mb	187 (60.5)	85 (54.5)
Not Available	45 (14.6)	26 (16.7)

ECOG PS Eastern Cooperative Oncology Group performance status, PD-L1 programmed death ligand-1, TC tumor cells

^a^Using a IHC assay with JS311 antibody

As of the data cutoff date of August 31, 2022, in the intention-to-treat (ITT) population, for one in the toripalimab group and seven in the placebo group (who crossed over to receive toripalimab as per the protocol), had completed all required study treatments. Notably, 45 patients (14.6%) in the toripalimab group completed the full two-year treatment course. Furthermore, 81 patients (51.9%) in the placebo group were switched to active crossover treatment following disease progression as assessed by the investigator, with 74% of these patients initiating treatment within three weeks. In total, 17.2% (53/309) of toripalimab patients and 65.4% (102/156) of placebo patients received subsequent anti-PD-1/PD-L1 therapy, including both active crossover and post-study treatments (Supplementary Table 1).

**Fig. 2:**
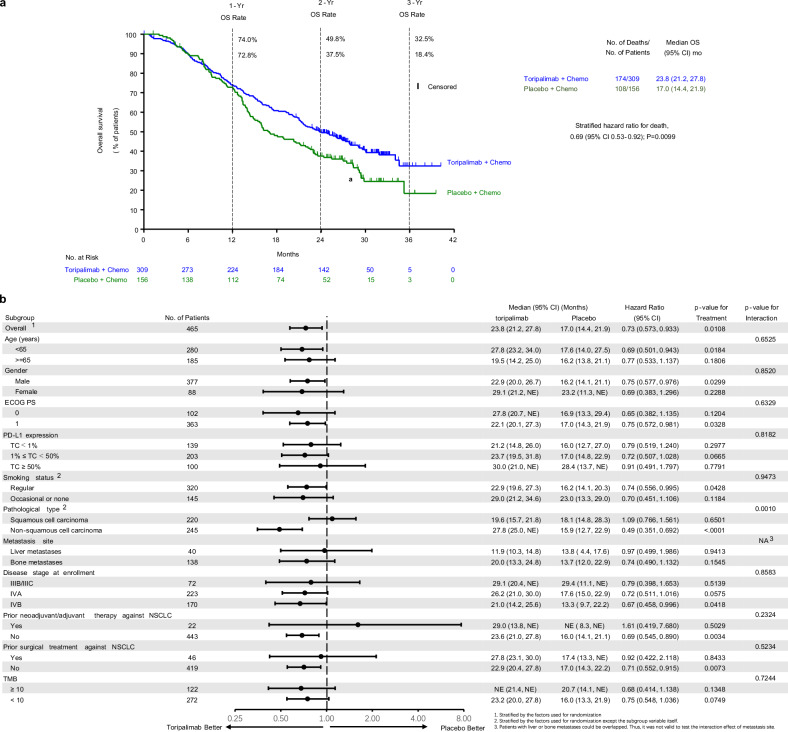
OS in the Intention-to-Treat Population. **a** The Kaplan-Meier estimates of OS comparing the toripalimab plus chemotherapy group with the placebo plus chemotherapy group as first-line treatment for patients with advanced NSCLC. Censored patients are marked with “┃” in the graph. Numbers of patients at risk at indicated time points are shown below the x-axis. Number of events, median OS, 1-, 2- and 3-year OS rates, and stratified hazard ratio for death are shown to the right of Kaplan-Meier curves. **b** Overall survivals in key subgroups. All hazard ratios were computed using the Cox proportional hazards model. All *P* values were two-sided with no adjustment of multiplicity. The *P* values of comparing the Kaplan-Meier curves were computed using the log-rank test stratified by the baseline PD-L1 expression status, histology, and smoking status. The *P* values of testing the interaction of the subgroup variables with the treatment were computed using the Cox proportional hazards regression model with the treatment group, the subgroup variable and their interaction as the covariates. HR, hazard ratio; NA, not available; NSCLC, non–small-cell lung cancer; PD-L1, programmed death ligand-1; PFS, progression-free survival

As noted in previous reports, the interim analysis had exceeded the pre-specified efficacy boundary,^5^ rendering the pre-specified final analysis descriptive. By the cutoff date of August 31, 2022, with a median survival follow-up of 21.2 months, the final analysis of overall survival (OS) was triggered by 283 death events. Consistent with interim results, a notable enhancement in OS was observed in the toripalimab group than that in the placebo group [median OS: 23.8 vs. 17.0 months, hazard ratio (HR) 0.69 (95% CI: 0.57–0.93), nominal P = 0.01]. The 1-, 2-, and 3-year OS rates were 74.0% vs. 72.8%, 49.8% vs. 37.5%, and 32.5% vs. 18.4%, respectively (Fig. 2a). The effect of toripalimab on OS was consistent across all major subgroups, with favorable outcomes observed in toripalimab-treated patients (Fig. 2b). Within subgroups based on PD-L1 expression (TC < 1% (*n* = 139), 1–49% (*n* = 203), ≥50% (*n* = 100), and not evaluable (*n* = 23)), the HRs were 0.79 (95% CI: 0.52–1.24), 0.72 (95% CI: 0.51–1.03), 0.91 (95% CI: 0.49–1.80), and 0.40 (95% CI: 0.15–1.10), respectively (Supplementary Fig. 1).

A significant survival benefit was seen in the non-SCC subgroup [median OS 27.8 vs. 15.9 months, HR 0.49 (95% CI: 0.35–0.69), *P* < 0.001]. However, no such difference was observed in the SCC subgroup, where the final OS analysis showed no statistically significant difference [median OS 19.6 vs. 18.1 months, HR 1.09 (95% CI: 0.77–1.56), *P* = 0.65] (Supplementary Fig. 2).

As of August 31, 2022, patients in the toripalimab group received a median of 9 cycles of treatment, while those in the placebo group received a median of 8 cycles (Supplementary Table 2). No new safety concerns emerged following prolonged toripalimab exposure for up to two years. The incidence of Grade ≥3 adverse events (AEs) (78.9% vs. 82.1%) was comparable between the two groups (78.9% vs. 82.1%), as was the rate of serious adverse events (SAEs) (46.4% vs. 35.3%). AEs leading to treatment discontinuation were more frequent in the toripalimab group (15.3% vs. 3.2%), as were investigator-assessed immune-related adverse events (irAEs) (50.6% vs. 21.2%) and Grade ≥3 irAEs (16.9% vs. 3.2%) (Supplementary Table 3). Supplementary Table 4 lists the most common AEs observed. Increased alanine aminotransferase (ALT), hypo- and hyperthyroidism, diarrhea, and rash were more frequently reported in the toripalimab group. The irAEs were consistent with those seen in patients treated with checkpoint inhibitors (Supplementary Table 5).

Among the 465 eligible enrolled patients, whole exome sequencing was successfully performed on tumor tissue samples from 394 patients as previously described.^15^ Following the updated OS analysis, patients with mutations in the *FA-PI3K-Akt* pathway and *IL-7* signaling pathway were associated with improved OS in the toripalimab group (interaction *P* = 0.006, and 0.001 respectively) compared to the interim analysis results (Supplementary Fig. 3). To further validate the previous findings from tumor tissue profiling, ctDNA were extracted from 460 pretreatment plasma samples and subjected to deep panel-based sequencing (520-gene panel). Among them, 387 patients (84.1%) tested positive for somatic alterations (ctDNA positive). The absence of baseline ctDNA correlated with improved PFS and OS but did not predict a response to toripalimab treatment (Supplementary Fig. 4).

**Fig. 3:**
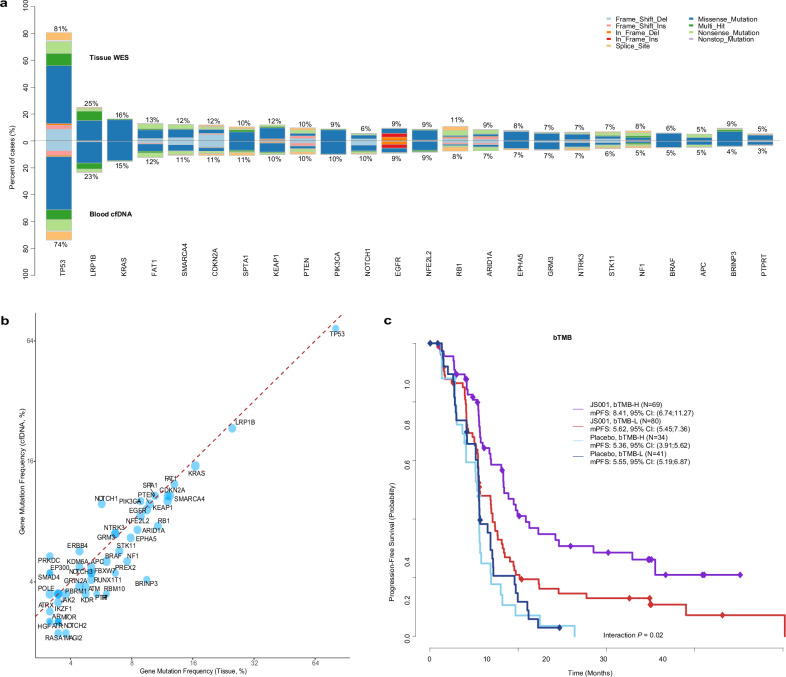
The concordance of mutational landscape between ctDNA and tissue WES data. **a** The matched frequency of top mutated genes derived from blood-based ctDNA and tumor WES data, ordered by the prevalence rate in ctDNA. **b** The mutations of top mutated genes between ctDNA profiling and matched tissue were strongly concordant. **c**The Kaplan-Meier estimates of PFS stratified by bTMB status in ITT patients. Low ctDNA shedders (<5% tumor fraction) were removed to ensure the specimens had subclone mutations adequately captured by the sequencing assay. ctDNA, circulating tumor DNA; WES, whole exome sequencing; TMB, tumor mutational burden; bTMB, blood tumor mutational burden; PFS, progression-free survival; ITT, intention-to-trea;JS001: toripalimab

Notably, the overall mutational landscape of ctDNA greatly exhibited a striking resemblance to the results obtained from tissue-based analysis. The mutation prevalence of the top mutated genes from ctDNA profiling was highly correlated with that from the tissue profiling (rho=0.81, *P* < 3.7e-13, Fig. 3a) in matched tissue and pre-treatment blood samples. Mutations of individual genes were also strongly concordant between ctDNA and tissue samples (median Cohen’s Kappa: 0.71, median PPV: 0.82, Fig. 3b) although the mutation frequencies of the majority of genes were slightly lower in ctDNA likely due to a lower amount of tumor DNA present in the blood compared to the tumor tissue. Similarly, bTMB also exhibited a strong correlation with tissue-based TMB (Supplementary Fig. 5). Patients with high bTMB showed significantly better PFS in the toripalimab group (interaction *P* = 0.02) (Fig. 3c). High TMB—whether measured in tissue or blood—was associated with significantly longer PFS in the toripalimab group only among non-squamous NSCLC patients (Supplementary Fig. 6), but did not have predictive value for OS (Supplementary Fig. 7)

Gene Set Enrichment Analysis on the ctDNA data was also performed independently on significantly interacted genes, as was done in tissue WES data,^15^ to identify over-represented biologic pathways. The *FA*-*PI3K-Ak* and *IL-7* signaling pathways remained in the top enriched pathways despite the number of genes being substantially limited by the designed panel (520 genes in total) (Supplementary Fig. 8). Altered *FA*-*PI3K-Akt* and *IL-7* signaling pathways by ctDNA analysis were also predictive of PFS (interaction *P* = 0.008 and 0.002 respectively) (Supplementary Fig. 8b, c), even though some genes were absent in the NGS panel compared with WES. Notably, mutations in the FA-PI3K-Akt and IL-7 signaling pathways displayed minimal predictive value for SCC (Supplementary Fig. 3), and we observed a distinct biomarker profile in SCC (Supplementary Fig. 9).

**Fig. 4:**
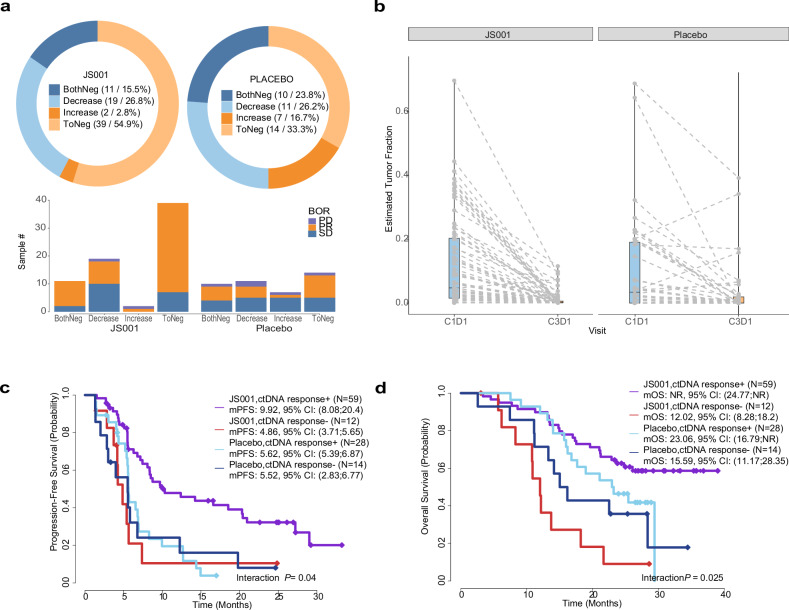
Analyses of ctDNA dynamics at C3D1. **a** Distribution of tumor load changes in the two arms and association with the best overall response. Tumor load dynamics were categorized into four groups: Negative in both baseline and C3D1 timepoints(BothNeg), Decrease at C3D1(Decrease), Increase at C3D1(Increase), and Reduction to undetectable levels at C3D1(ToNeg). The decrease in the toripalimab arm was more prominent. A higher percentage of patients exhibiting partial response was also observed in the toripalimab group. **b** Tumor load, as estimated by tumor fraction in blood, decreased at C3D1 for both toripalimab and placebo arms. Response of ctDNA at C3D1 is predictive of PFS (**c**) and OS (**d**) in ITT patients. The ctDNA response was defined as >85% reduction of tumor load from C1D1 or no detection of ctDNA at C3D1. ctDNA, circulating tumor DNA, ITT, intention-to-treat. JS001: toripalimab

To explore the potential of dynamic ctDNA profiling, we conducted ctDNA analysis on blood samples collected at cycle 3, day 1 (C3D1) from 113 patients (71 in the toripalimab group and 42 in the placebo group). Interestingly, 82.1% (32/39) of the ctDNA-negative patients in the toripalimab group showed a clinical response, while only 57.1% (8/14) in the placebo group responded (Fig. 4a). Among the 32 responders in the toripalimab group, 56.3% (18/32) demonstrated a response at the initial RECIST evaluation, suggesting that ctDNA changes occurred prior to radiographic identification of clinical responses. Furthermore, among the 32 responders in the toripalimab arm, only 56.3% (18/32) displayed a response at the initial RECIST evaluation, suggesting that ctDNA changes preceded radiographic identification of clinical responses in some cases. Significantly, tumor burden, as assessed by the tumor fraction in blood, notably decreased at C3D1 in both toripalimab and placebo arms (nominal *P* < 0.001), with a more pronounced reduction observed in the toripalimab arm(Fig. 4b). Moreover, a significant reduction in tumor burden (defined as either a six-fold decrease or reaching an undetectable level) predicted improved PFS and OS for toripalimab-treated patients in both the ITT and non-SCC populations (Fig. 4c, d and Supplementary Fig. 10a–e).

To further explore the mechanism possible leads to the survival different between SCC and non-SCC, we analyzed the genomic profiling and ctDNA dynamics. We observed distinct mutational patterns between SCC and non-SCC for the top mutated genes (Supplementary Fig. 11a, b). More patients in SCC harbored PIK3CA, PTEN, and NFE2L2. By contrast, non-SCC patients carried more mutations of KRAS, SMARCA4, and STK11. In addition, patients with SCC had higher ctDNA+ rate at the baseline comparing to ones with non-SCC. Furthermore, reduction of ctDNA at C3D1 was more frequent in SCC placebo group (79.0%, 15 out of 19) than in non-squamous placebo group (56.5%, 13 out of 23) (Supplementary Fig. 11c).

## Discussion

In the final OS analysis from the CHOICE-01 trial, incorporating toripalimab into standard first-line chemotherapy dramatically enhanced OS outcomes of NSCLC patients who do not possess EGFR/ALK driver mutations, aligning with the results from the interim OS analysis. Furthermore, this study offers the most extensive dataset to date, examining a broad spectrum of exploratory endpoints to assess the impacts of treatment.

Subgroup analyses revealed that the survival advantage was markedly more substantial in the non-squamous cell carcinoma (non-SCC) subgroup. In contrast, there was no observed benefit in the squamous cell carcinoma (SCC) subgroup, diverging from findings in previous research.^3^ Importantly, some phase III randomized trials investigating first-line immuno-chemotherapy in SCC have not yet released their final OS analysis.^20–23^ The lack of OS benefits in SCC in the current study may partly stem from immediate active crossover implementation. The median OS for SCC in the placebo arm was 18.1 months, surpassing the 17.2 months observed in the experimental arm in the KEYNOTE-407 study and substantially exceeding the historical control of 11.6 months,^3^ indicating an OS crossover effect. Furthermore, despite the high rate of active crossover, non-SCC patients still experienced marginal OS benefits from first-line immuno-chemotherapy compared to crossover patients in the placebo arm. Additionally, certain markers demonstrating favorable predictive value for survival in the non-SCC population did not exhibit corresponding predictive value in SCC individuals, further underscoring the distinct molecular and immune characteristics between these two histological types of lung cancer.^11,12,24^ From the perspective of genomic profiling and ctDNA dynamics, we did observe distinct mutational patterns between SCC and non-SCC for the top mutated genes. More patients in SCC harbored PIK3CA, PTEN, and NFE2L2. By contrast, non-SCC patients carried more mutations of KRAS, SMARCA4, and STK11. These differences, combined with the benefits of second-line PD-1 inhibitors and a high crossover rate in SCC, together may have contributed to the fact that SCC patients did not show sufficient survival benefits. Therefore, we propose, that first-line treatment strategies may need to be tailored differently for patients with these two histological subtypes. In the subgroup analysis of PD-L1, we unexpectedly founded that for the population with PD-L1 > = 50%, OS did not demonstrate a significant improvement in the toripalimab group. For the stratification factor is PD-L1 TC ≥ 1% instead of TC ≥ 50%, there is an imbalance in sample size for the two groups in PD-L1 high expression subgroup, which could potentially lead to bias in the analysis. In this study, patients with stage III derived limited benefit from toripalimab, which may due to the sample size. The Neotorch trial indicated that resectable stage III patients significantly benefited from toripalimab treatment.^25^ Therefore, chemotherapy combined with immunotherapy remains the preferred treatment option for stage III NSCLC.

Analyzing ctDNA through liquid biopsy holds promise as a minimally invasive approach to detect tumor mutations and track disease progression. Previous studies have explored ctDNA’s potential as a predictive biomarker for immunotherapy.^26^ Our study, with a prespecified biomarker analysis, marks the first attempt to integrate and align WES of tumor biopsies with blood ctDNA sequencing to forecast clinical response within a randomized phase III framework. We investigated baseline bTMB, dynamic ctDNA response, ctDNA-derived genetic patterns, directly comparing them with tissue-based WES results and correlating them with clinical outcomes. Previous investigations on bTMB have yielded inconsistent findings, hindering its clinical application as a predictor for PFS or OS.^27^ Recent studies have introduced refined bTMB calculations, such as low allele frequency (LAF)-bTMB, showing promise in predicting survival benefit^s^.^28^ The B-F1RST and BFAST studies prospectively assessed bTMB’s predictive value in NSCLC patients receiving ICI monotherapy, indicating its potential to predict ORR, though not PFS and OS. In our study, profiling ctDNA from baseline blood samples revealed a consistent mutational landscape and TMB compared to tissue-based WES profiling. High bTMB was predictive of better PFS, but not OS in the combination group. A potential challenge in estimating blood TMB lies in the low shedding of ctDNA in certain patients with cancer, which may hinder the detection of low-frequency mutations, resulting in reduced TMB. Indeed, our analysis observed an improved correlation between bTMB and tissue-based TMB when raising the threshold of tumor fraction from 0.5 to 5% or higher to ensure adequate capture of subclone mutations by the NGS assay.

Furthermore, ctDNA-based genetic analysis confirmed the findings derived from tissue-based WES. Genetic alterations in the *FA-PI3K-Akt* and *IL-7* signaling pathways as identified through ctDNA profiling, were found to be predictive of improved PFS and OS, aligning with the initial observations from WES. The consistency between tissue-based WES and blood-based ctDNA sequencing highlights ctDNA’s potential as a predictive biomarker for the immuno-chemotherapy.

Conventional imaging for initial response assessment often fails to identify patients who will achieve durable clinical benefit (DCB). Early clearance of ctDNA has already been demonstrated as a predictor for targeted therapies and immunotherapy in the studies such as AURA3, FLAURA, Imvigor 010 and IMpower 150.^25,29–32^ Our study also leveraged dynamic changes in ctDNA to early predict DCB and ultimate outcomes for immuno-chemotherapy, revealing that patients with reduced ctDNA levels at C3D1 in the combination group exhibited significantly better PFS and OS. However, further validation through prospective studies is warranted to ensure robustness of these findings.

The limitation of the study includes that although we have suggested the potential of biomarker exploration based on liquid biopsy, the optimal panel selection and the efficacy of adjusting treatment plans based on biomarkers still need further exploration. Additionally, while toripalimab demonstrated a favorable impact on PFS for SCC patients, this benefit did not translate into a significant improvement in OS. The disparity in immunotherapy responses observed between SCC and non-SCC patients necessitates further exploration to elucidate the underlying mechanisms.

In summary, the combination regimen of toripalimab with chemotherapy for treatment-naïve patients with advanced NSCLC lacking *EGFR/ALK* driver mutations led to better OS compared to chemotherapy alone. Analyzing both tumor tissues and dynamic blood samples through genomic sequencing could offer valuable insights into identifying the patient population that benefits most from first-line immuno-chemotherapy in advanced NSCLC. The differential survival benefits from ICIs and varied predictive biomarkers between SCC and non-SCC highlight a potential focus for further investigation.

## Methods

### Study design and participants

#### Study strategy and participant enrollment

Our study was carried out at 59 hospitals across China, enrolling previously untreated patients who were suffering from locally advanced or metastatic NSCLC. The full eligibility criteria are detailed in Supplement 2.

The primary endpoint was PFS as assessed by investigators. A comprehensive list of endpoints is outlined in the study protocol.

The present study received approval from the institutional review boards of all participating sites. It adhered to local legal and regulatory standards, as well as international ethical guidelines for biomedical research, the International Conference on Harmonization Good Clinical Practice standards, and the Declaration of Helsinki. Written informed consent was signed by every participant.

#### Randomization and masking

Patients (random ratio of 2:1)were subjected to toripalimab or placebo combined with chemotherapy. The randomization was stratified by baseline PD-L1 expression (tumor cell [TC] < 1% vs. TC ≥ 1%), histological type (SCC vs. non-SCC), and smoking status (frequent smoker [≥20 pack years] vs. infrequent or non-smoker), as detailed earlier.^5^ An independent and unblinded statistician from a third-party vendor with perform randomization of both patients and investigational drugs, while the rest of the research team remained blinded to treatment allocation.

#### Procedures

After randomization, participants were assigned to receive either toripalimab or placebo in combination with chemotherapy. Modifications to the toripalimab dose were not permitted, but adjustments to chemotherapy doses were allowed in accordance with the study protocol. For squamous-NSCLC-patients, the treatment regimen consisted of nab-paclitaxel, carboplatin, and either toripalimab or placebo. For nonsquamous NSCLC, the regimen included pemetrexed, cisplatin or carboplatin, and either toripalimab or placebo. Treatment would not stop unless following cases occur (no matter which happened first), disease progression, death, intolerable toxicity, investigator decision, withdrawal, or the completion of a two-year treatment period. After progression was confirmed by the investigator, placebo patients were transitioned to toripalimab monotherapy.

Tumor assessments were conducted at baseline, every 6 weeks for the 1st year, and every 9 weeks thereafter. Clinical responses were assessed by researchers through blinded independent central review (BICR) using RECIST criteria (v1.1). Safety and tolerability were monitored in each patient received any treatment, continuing 3 months after the last dose or beginning new anticancer therapy. Adverse events were classified and graded based on the National Cancer Institute Common Terminology Criteria for Adverse Events (NCI-CTCAE, v5.0). An independent Data Monitoring Committee (iDMC) reviewed safety and efficacy data at predetermined analysis points, with its results were conducted by an independent third-party organization.

Samples (archival or fresh tumor biopsy) were collected from patients before treatment. The expression level of PD-L1 was measured using immunohistochemistry via the JS311 assay in MEDx (China), and positive PD-L1 was defined as ≥1% expression of tumor cells.

Whole-exome sequencing (WES) was performed on tumor biopsy samples containing at least 20% tumor content, in conjunction with matched peripheral blood mononuclear cell samples, at a central laboratory (China). Additionally, blood samples were obtained from patients at baseline and post-treatment visits, and were processed for sequencing at another central laboratory (Burning Rock, Guangzhou, China). Somatic mutations and copy number aberrations were reported by the vendors as previously described.^15,33,34^

After excluding known oncogenic driver mutations and germline single nucleotide polymorphisms (SNPs), tumor mutational burden (TMB) based on tumor tissue was calculated through the analysis of somatic mutations, which encompassed coding base substitutions and INDELs, expressed per megabase (Mb). A TMB-high cutoff was defined as ≥10 mutations/Mb, aligning with the criterion employed by the FoundationOne CDx assay, which is FDA-approved as a companion diagnostic for pembrolizumab. Similarly, blood-based TMB (bTMB) was determined by counting somatic single-nucleotide variants (SNVs) and indels detected at an allele frequency of ≥0.2% within the coding regions of the targeted genes per megabase. To align with tumor tissue TMB, a bTMB-high (bTMB-H) cutoff of ≥10 mutations/Mb was also applied. The ctDNA fraction was estimated based on the allele frequencies of autosomal somatic mutations, as previously described. Pathway analysis was conducted for genes with significant interaction with clinical outcomes (PFS/OS) using Gene Set Enrichment Analysis (GSEA), with gene sets derived from the MSigDB database.^35^ The concordance of mutation prevalence between tissue profiling and ctDNA sequencing was evaluated using Spearman’ s rank correlation. The agreement of mutation calls for individual genes was assessed by Cohen’ s kappa statistic and positive predictive values. Associations between individual genomic features and PFS/ OS were analyzed using Cox regression to assess the significance of the interaction effects.

#### End Points

The primary end point was investigator-assessed PFS. Secondary end points included PFS by BIRC, OS, ORR, and safety. Exploratory endpoints aimed to examine the potential relationship between toripalimab’s immunogenic response and its safety and efficacy, as well as to identify subpopulations with the best efficacy predictions through biomarker analysis (including but not limited to PBMCs, PD-L1, WES, etc.).

### Statistical analysis

The required sample size for the study was determined according to the following assumptions: 450 patients were necessary to observe 356 progression-free survival (PFS) events, enabling the detection of a hazard ratio of 0.7 with 85% power at a two sided significant level of 0.05. The overall type I error rate was controlled through the implementation of the Pocock boundary, which was approximated by the Lan-DeMets alpha spending function. PFS, ORR, and OS were tested in a hierarchical manner. The study was designed with 83% power to identify a 0.68 hazard ratio for OS. Stopping boundaries for OS analysis were calculated using the O’Brien Fleming boundary, again approximated by the Lan-DeMets alpha spending function. Further details of the statistical analyses are provided in Supplement 3.

Data in this study were analyzed using SAS version 9.4 by SAS Institute and R software.

## Supplementary information

Supplement 1

Supplement 2

Supplement 3

## Data Availability

The data utilized in this study are presented within the article or its Supplementary appendix. Additional data in a de-identified form could contact the corresponding author.
