# Supplementary material for: Toripalimab plus chemotherapy for first line treatment of advanced non-small cell lung cancer (CHOICE-01): final OS and biomarker exploration of a randomized, double-blind, phase 3 trial
Source: Signal Transduct Target Ther. 2024 Dec 24;9:369. doi: 10.1038/s41392-024-02087-6 (PMC11666711; doi:10.1038/s41392-024-02087-6)
Supplement: Supplementary file 1 — Supplement 1 [file 41392_2024_2087_MOESM1_ESM.docx]

Supplementary Materials for

Toripalimab plus Chemotherapy for First Line Treatment of Advanced Non-Small Cell Lung Cancer (CHOICE-01): Final OS and biomarker exploration of a randomized, double-blind, phase 3 trial

**Authors:** Jia Zhong, MD^1^*; Kailun Fei, MD^1^*; Lin Wu, MD^2^; Baolan Li, MD^3^; Zhijie Wang, MD^1^; Ying Cheng, MB^4^; Xiaoling Li, MD^5^; Xicheng Wang, MM^6^; Liang Han, MM^7^; Xiaohong Wu, MB^8^; Yun Fan, MD^9^; Yan Yu, MD^10^; Dongqing Lv, MM^11^; Jianhua Shi, MM^12^; Jianjin Huang, MB^13^; Shaozhang Zhou, MD^14^; Baohui Han, MD^15^; Guogui Sun, MD^16^; Qisen Guo, MM^17^; Youxin Ji, MD^18^; Xiaoli Zhu, MD^19^; Sheng Hu, MD^20^; Wei Zhang, MD^21^; Qiming Wang, MD^22^; Yuming Jia, MB^23^; Ziping Wang, MD^24^; Yong Song, MD^25^; Jingxun Wu, MD^26^; Meiqi Shi, MB^27^; Xingya Li, MD^28^; Zhigang Han, MM^29^; Yunpeng Liu, MD^30^; Zhuang Yu, MD^31^; An-Wen Liu, MD^32^; Xiuwen Wang, MD^33^; Caicun Zhou, MD^34^; Diansheng Zhong, MD^35^; Liyun Miao, MD^36^; Zhihong Zhang, MD^37^; Hui Zhao, MD38; Jun Yang, MD^39^; Dong Wang, MD^40^; Yingyi Wang, MD^41^; Qiang Li, MM^42^; Xiaodong Zhang, MB^43^; Mei Ji, MD^44^; Zhenzhou Yang, MD^45^; Jiuwei Cui, MD^46^; Beili Gao, MM^47^; Buhai Wang, MD^48^; Hu Liu, MD^37^; Lei Nie, MB^49^; Mei He, MB^50^; Shi Jin, MD^51^; Wei Gu, MM^52^; Yongqian Shu, MD^53^; Tong Zhou, MM^54^; Jian Feng, MM^55^; Xinmei Yang, MB^56^; Cheng Huang, MM^57^; Bo Zhu, MD^58^; Yu Yao, MD^59^; Sheng Yao, PhD^60,61^，Jianjun Yu, PhD^60,61^; Shang li Cai, PhD^62^; Yiran Cai, PhD^62^; Jiachen Xu, MD^1^;Wei Zhuang^1^; Xianmin Luo^61^；Jianchun Duan, MD^1#^ and Jie Wang, MD^1#^

Correspondence to: [zlhuxi@163.com](mailto:zlhuxi@163.com); [duanjianchun79@163.com](mailto:duanjianchun79@163.com).

**This PDF file includes:**

Supplementary table 1 to 5

Supplementary figure 1 to 11

Supplemental Methods

**Supplemental Tables**

| **Supplementary table 1.** New anti-Cancer therapy in Intent-to-Treat Population after the study New anti-cancer treatments included active crossover to toripalimab in the placebo group. | | | |
| --- | --- | --- | --- |
|  | Toripalimab + Chemo  N = 309 (%) | Placebo + Chemo  N= 156 (%) | Total  N=465 (%) |
| New anti-cancer therapy | 181 (58.6) | 132(84.6) | 313(67.3) |
| Systemic treatment | 152 (49.2) | 121(77.6) | 273(58.7) |
| Surgical treatment | 5 (1.6) | 1 (0.6) | 6 (1.3) |
| Radiotherapy | 42 (13.6) | 34(21.8) | 76(16.3) |
| Other | 54 (17.5) | 37(23.7) | 91(19.6) |
|  | | | |
| Systemic treatment | 152 (49.2) | 121(77.6) | 273(58.7) |
| PD-1/L1 Inhibitors | 53 (17.2) | 102 (65.4) | 155 (33.3) |
| TORIPALIMAB | 10 (3.2) | 84 (53.8) | 94(20.2) |
| SINTILIMAB | 15 (4.9) | 17 (10.9) | 32 (6.9) |
| CAMRELIZUMAB | 10 (3.2) | 8 (5.1) | 18 (3.9) |
| TISLELIZUMAB | 11 (3.6) | 5(3.2) | 16(3.4) |
| ATEZOLIZUMAB | 4 (1.3) | 0 | 4 (0.9) |
| PEMBROLIZUMAB | 2 (0.6) | 2 (1.3) | 4 (0.9) |
| NIVOLUMAB | 2 (0.6) | 1 (0.6) | 3 (0.6) |
| PM-8001 | 0 | 3 (1.9) | 3 (0.6) |
| Cadonilimab | 0 | 1 (0.6) | 1(0.2) |
| PENPULIMAB | 1 (0.3) | 0 | 1 (0.2) |
| RC 98 | 1 (0.3) | 0 | 1 (0.2) |
| TQ B2450 | 1 (0.3) | 0 | 1 (0.2) |
| Cytotoxic Therapy | 119(38.5) | 80(51.3) | 199(42.8) |
| Tyrosine Kinase Inhibitor | 73 (23.6) | 48(30.8) | 121(26.0) |
| Anti-angiogenic Agent (non-TKI) | 48 (15.5) | 24 (15.4) | 72 (15.5) |
| Traditional Chinese Medicine | 1 (0.3) | 1 (0.6) | 2 (0.4) |
| Other | 9(2.9) | 4(2.6) | 13(2.8) |
| Unknown | 3 (1.0) | 1 (0.6) | 4 (0.9) |
| New anti-cancer therapy | 181 (58.6) | 132(84.6) | 313(67.3) |
| Data Cutoff: August 31, 2022 | | | |

| **Supplementary table 2.** Toripalimab/Placebo Exposure. | | |
| --- | --- | --- |
|  | **Toripalimab + Chemotherapy N=308** | **Placebo + Chemotherapy N=156** |
| **Actual number of doses** | | |
| Median | 9.0 | 8.0 |
| Min-Max | 1 - 35 | 1 - 35 |
| **Actual duration of medication (weeks)** | | |
| Median | 28.7 | 21.6 |
| Min-Max | 0.1 – 114.0 | 0.1 – 119.3 |
| **Actual cumulative dose (mg)** | | |
| Median | 2160.0 | 1920.0 |
| Min-Max | 240 - 8400 | 240 - 8400 |
| **Actual dose intensity (mg/week)** | | |
| Median | 75.5 | 75.2 |
| Min - Max | 26.0 - 80.6 | 46.5 - 80.0 |
| *Compliance classification;Data Cutoff: August 31, 2022 | | |

| **Supplementary table 3.** Summary of Treatment Emergent Adverse Events (Safety Population) | | |
| --- | --- | --- |
| **Adverse Event Category** | **Toripalimab + Chemotherapy  N=308** | **Placebo + Chemotherapy N=156** |
| Any TEAE | 305 (99.0) | 156 (100) |
| Any TEAE Grade ≥3 | 243 (78.9) | 128 (82.1) |
| Any SAE | 143 (46.4) | 55 (35.3) |
| Any TEAEs resulting in death | 17 (5.5) | 4 (2.6) |
| Any TEAE results in permanent discontinuation of toripalimab/placebo | 47 (15.3) | 5 (3.2) |
| Any TEAE leading to interruption of toripalimab/placebo | 198 (64.3) | 86 (55.1) |
| Any Investigator-determined irAE | 156 (50.6) | 33 (21.2) |
| Any Investigator-determined irAE with Grade ≥3 | 52 (16.9) | 5 (3.2) |
| Any infusion-related reaction | 8 (2.6) | 2 (1.3) |
| TEAE: treatment-emergent adverse event, SAE: serious adverse event, irAE: immune-related adverse event.  Note: TEAEs are adverse events that either start or worsen in severity on or after the date/time of first dose of study treatment and on or before 90 days after the date/time of last dose of study treatment or before starting a new anti-cancer therapy, whichever occurs first.  Data Cutoff: August 31, 2022 | | |

| **Supplementary table 4.** Treatment Emergent Adverse Events Reported in At Least 10% of the Patients in either treatment group | | | | |
| --- | --- | --- | --- | --- |
| **System Organ Class**  Preferred Term | **Toripalimab+Chemotherapy N=308, N (%)** | | **Placebo + Chemotherapy  N=156, N (%)** | |
|  | **All Grades** | **Grades ≥3** | **All Grades** | **Grade ≥3** |
| Any TEAE (%) | 305(99.0) | 243(78.9) | 156(100) | 128(82.1) |
| Blood and lymphatic system disorders | | | | |
| Anemia | 275(89.3) | 93(30.2) | 148(94.9) | 56(35.9) |
| Neutropenia | 259(84.1) | 171(55.5) | 137(87.8) | 85(54.5) |
| Leukopenia | 258(83.8) | 110(35.7) | 131(84.0) | 65(41.7) |
| Thrombocytopenia | 215(69.8) | 53(17.2) | 114(73.1) | 28(17.9) |
| Lymphopenia | 35(11.4) | 7(2.3) | 22(14.1) | 2(1.3) |
| Investigations | | | | |
| Alanine aminotransferase increased | 121(39.3) | 5(1.6) | 49(31.4) | 3(1.9) |
| Aspartate aminotransferase increased | 120(39.0) | 1(0.3) | 41(26.3) | 2(1.3) |
| Weight decreased | 50(16.2) | 0 | 27(17.3) | 0 |
| Hyperbilirubinemia | 36(11.7) | 1(0.3) | 12(7.7) | 1(0.6) |
| Metabolism and nutrition disorders | | | | |
| Decreased appetite | 109(35.4) | 3(1.0) | 57(36.5) | 2(1.3) |
| Hypoproteinemia | 83(26.9) | 0 | 30(19.2) | 0 |
| Hyperlipidemia | 63(20.5) | 6(1.9) | 34(21.8) | 2(1.3) |
| Hyperglycemia | 57(18.5) | 6(1.9) | 21(13.5) | 1(0.6) |
| Hypokalemia | 54(17.5) | 9(2.9) | 29(18.6) | 5(3.2) |
| Hyponatremia | 36(11.7) | 9(2.9) | 15(9.6) | 2(1.3) |
| Hyperuricemia | 31(10.1) | 0 | 20(12.8) | 0 |
| Endocrine disorders | | | | |
| Hypothyroidism | 50(16.2) | 1(0.3) | 5(3.2) | 0 |
| Hyperthyroidism | 37(12.0) | 0 | 3(1.9) | 0 |
| Gastrointestinal disorders | | | | |
| Nausea | 102(33.1) | 1(0.3) | 57(36.5) | 0 |
| Constipation | 62(20.1) | 0 | 44(28.2) | 0 |
| Vomiting | 61(19.8) | 1(0.3) | 33(21.2) | 1(0.6) |
| Diarrhea | 45(14.6) | 6(1.9) | 13(8.3) | 0 |
| General disorders and administration site conditions | | | | |
| Fatigue | 113(36.7) | 5(1.6) | 57(36.5) | 3(1.9) |
| Pyrexia | 74(24.0) | 0 | 31(19.9) | 0 |
| Edema | 41(13.3) | 1(0.3) | 20(12.8) | 0 |
| Cardiac disorders | | | | |
| Arrhythmia | 67(21.8) | 2(0.6) | 38(24.4) | 1(0.6) |
| Musculoskeletal and connective tissue disorders | | | | |
| Musculoskeletal pain | 43(14.0) | 1(0.3) | 29(18.6) | 0 |
| Respiratory, thoracic and mediastinal disorders | | | | |
| Cough | 52(16.9) | 2(0.6) | 27(17.3) | 0 |
| Hemoptysis | 37(12.0) | 1(0.3) | 20(12.8) | 2(1.3) |
| Skin and subcutaneous tissue disorders | | | | |
| Rash | 73(23.7) | 5(1.6) | 25(16.0) | 3(1.9) |
| Infections | | | | |
| Pneumonia | 32(10.4) | 16(5.2) | 20(12.8) | 10(6.4) |
| TEAE = treatment-emergent adverse event. Note: Adverse event coding version: MedDRA 24.0.  Note: Adverse events that occurred during treatment are all adverse events that occurred between the first dose and 90 days after the last dose or before starting a new antineoplastic therapy, whichever occurred first.  For the same patient if the same adverse event occurs multiple times, that patient is counted only once in the same system organ classification and preferred terminology when calculating the number of adverse events.  Data Cutoff: August 31, 2022 | | | | |

| **Supplementary table 5.** Immune related adverse events (irAE) reported in more than one patient in either group as assessed by the investigator. | | | | |
| --- | --- | --- | --- | --- |
| **No. of patients (%)** | **Toripalimab + chemo N=308 (%)** | | **Placebo + chemo N=156 (%)** | |
|  | **All Grades** | **Grades ≥3** | **All Grades** | **Grade ≥3** |
| **Any irAE** | 50.6 | 16.9 | 21.2 | 3.2 |
| Hypothyroidism | 11.4 | 0.3 | 1.3 | 0 |
| Pneumonitis | 11.7 | 3.6 | 3.2 | 0 |
| Rash | 10.7 | 1.3 | 6.4 | 1.3 |
| Hyperthyroidism | 8.1 | 0 | 1.9 | 0 |
| Alanine aminotransferase increased | 6.5 | 0.3 | 1.9 | 0.6 |
| Aspartate aminotransferase increased | 6.5 | 0 | 1.3 | 0 |
| Thrombocytopenia | 5.2 | 3.2 | 1.3 | 0 |
| Thyroid function test abnormal | 3.6 | 0 | 3.2 | 0 |
| Amylase increased | 3.6 | 0 | 0.6 | 0 |
| Pyrexia | 3.2 | 0 | 0.6 | 0 |
| Hyperbilirubinemia | 2.9 | 0 | 0 | 0 |
| Anemia | 2.3 | 0.6 | 1.9 | 0 |
| Edema | 2.3 | 0 | 1.3 | 0 |
| Hyperglycemia | 3.2 | 1.0 | 1.3 | 0.6 |
| Leukopenia | 2.3 | 0.6 | 0.6 | 0 |
| Fatigue | 2.6 | 0.3 | 3.8 | 0 |
| Renal function test abnormal2.600.60Neutropenia | 1.9 | 1.3 | 0.6 | 0 |
| Arrhythmia | 3.9 | 0 | 1.3 | 0 |
| Pruritus | 1.6 | 0 | 0.6 | 0 |
| Hepatitis | 1.3 | 1.0 | 0 | 0 |
| Hypoproteinemia | 1.3 | 0 | 0 | 0 |
| Hyponatremia | 1.3 | 0.3 | 0 | 0 |
| Diarrhea | 1.3 | 0.3 | 0 | 0 |
| Hepatic function abnormal | 1.3 | 0.6 | 0 | 0 |
| Proteinuria | 1.3 | 0 | 0 | 0 |
| Renal injury | 1.6 | 0.3 | 0 | 0 |
| Gamma-glutamyl transferase increased | 1.0 | 0.6 | 0.6 | 0 |
| Decreased appetite | 1.3 | 0 | 0.6 | 0 |
| C-reactive protein increased | 1.0 | 0 | 0 | 0 |
| Blood creatine phosphokinase increased | 1.0 | 0 | 0 | 0 |
| Hypokalemia | 1.0 | 0 | 0 | 0 |
| Hyperlipidemia | 1.0 | 0 | 0 | 0 |
| Stomatitis | 1.0 | 0.3 | 0 | 0 |
| Pancreatitis | 1.0 | 0.3 | 0 | 0 |
| myocarditis | 1.0 | 0.6 | 0 | 0 |
| Arthritis | 1.0 | 0 | 0 | 0 |
| Pneumonia | 0.6 | 0.6 | 0 | 0 |
| Enteritis | 0.6 | 0.3 | 0 | 0 |
| Constipation | 0.6 | 0 | 0 | 0 |
| Vomiting | 0.6 | 0 | 0 | 0 |
| Note: For the same patient if the same adverse event occurs multiple times, that patient is counted only once in the same system organ classification and preferred terminology when calculating the number of adverse events.  Data Cutoff: August 31, 2022 | | | | |

**Supplemental Figures**


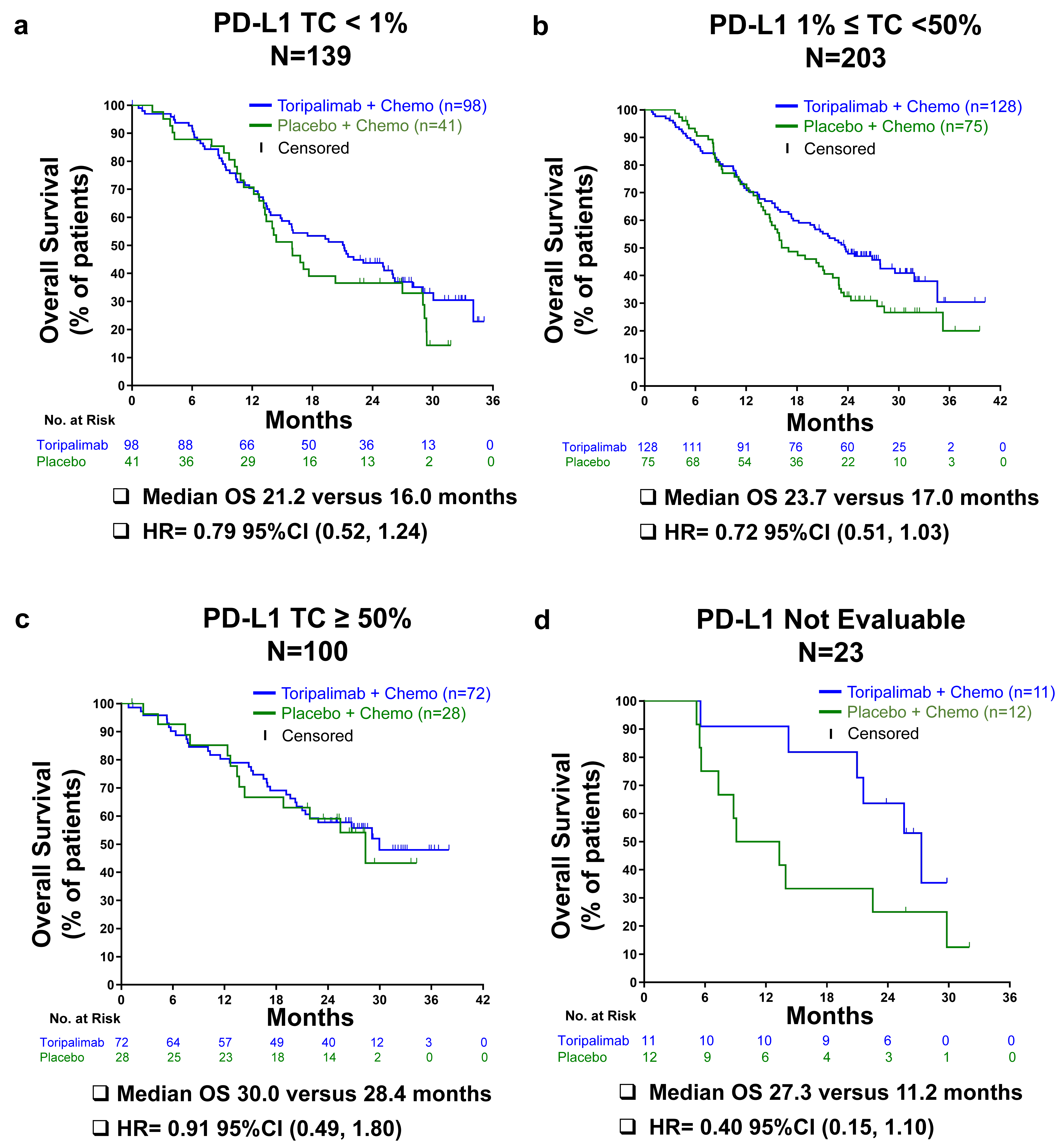


**Supplementary figure 1.** **Overall survival (OS) in the PD-L1 expression subgroups at the final OS analysis (Data Cutoff Date August 31, 2022).** Archival or fresh tumor biopsy samples were obtained from patients prior to treatment. PD-L1 expression was evaluated by immunohistochemistry (IHC) staining with JS311 antibody using a validated assay in a central lab. Kaplan-Meier-estimated OS curves are shown to compare the toripalimab plus chemotherapy group with the placebo plus chemotherapy group in PD-L1 tumor cell (TC) expression subgroups: TC<1% **(Panel a)**, 1% ≤ TC <50% **(Panel b)**, TC ≥ 50% **(Panel c)**, and PD-L1 not evaluable **(Panel d)**. Censored patients are marked with “┃” in the graph. Numbers of patients at risk at indicated time points are shown below x-axis. The median OS and stratified hazard ratio for OS are shown under the Kaplan-Meier curves.


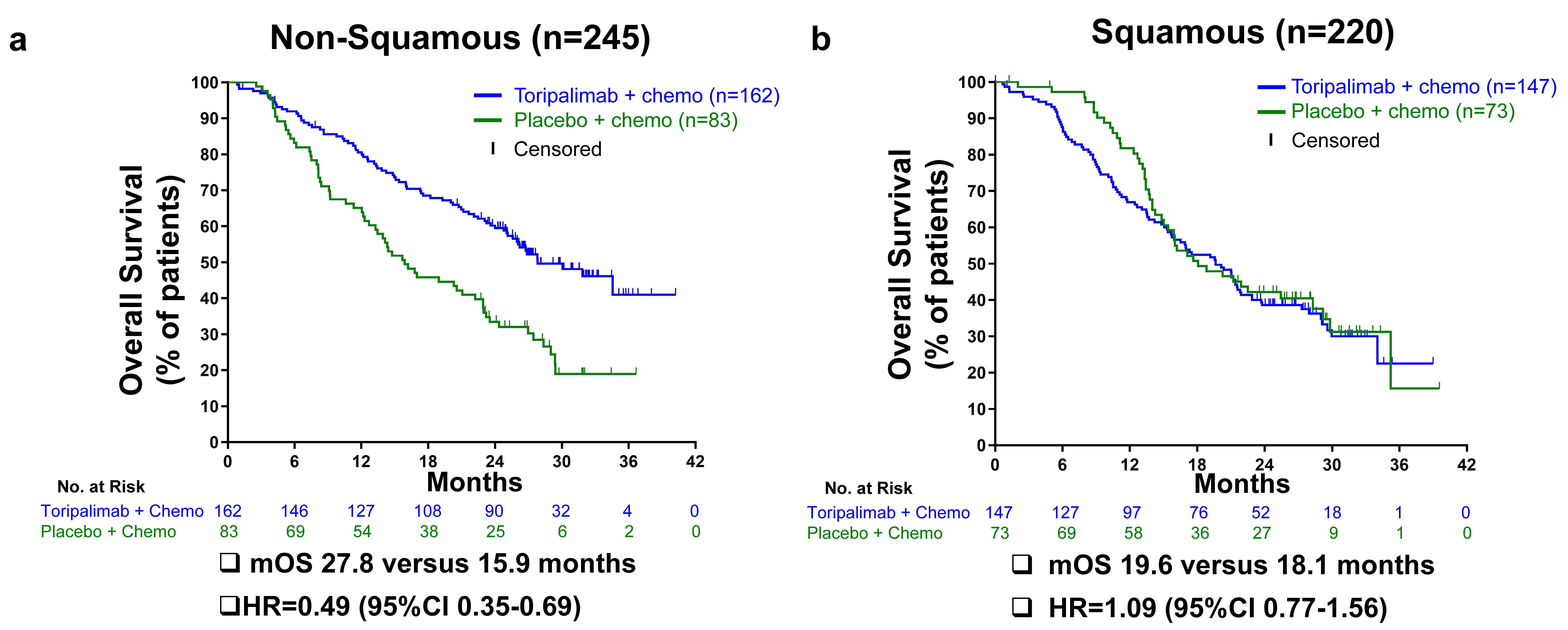


**Supplementary figure 2. Overall survival (OS) in the histology subgroups at the final OS analysis (Data Cutoff Date August 31, 2022).** Kaplan-Meier-estimated OS curves are shown to compare the toripalimab plus chemotherapy group with the placebo plus chemotherapy group in patients with non-squamous NSCLC **(Panel a)** and squamous NSCLC **(Panel b)**. Censored patients are marked with “┃” in the graph. Numbers of patients at risk at indicated time points are shown below x-axis. The median OS and stratified hazard ratio for OS are shown under the Kaplan-Meier curves.





**Supplementary figure 3.** **Associations of FA-PI3K-Akt and IL-7 pathway alterations with the treatment effects of OS.** The Kaplan-Meier estimates of OS stratified by *FA-PI3K-Akt* pathway alterations in ITT patients **(Panel a)**, non-squamous NSCLC **(Panel c)** and squamous NSCLC **(Panel e)**. The Kaplan-Meier estimates of OS stratified by *IL-7* pathway alterations in ITT patients **(Panel b)**, non-squamous NSCLC **(Panel d)** and squamous NSCLC **(Panel f)**. OS, overall survival; ITT, intention-to-treat; non-SCC, non-squamous; SCC, squamous; *FA-PI3K-Akt*, focal adhesion-phosphatidylinositol 3-kinase-Akt signaling pathway (COL3A1/ COL6A3/ FLT1/ FLINC/ HGF/ IRS1/ IRS2/ ITGA4/ ITGA8/ KDR genes); *IL-7*, interleukin-7 signaling pathways (HGF/ IRS1/ IRS2/ SMARCA4).

**
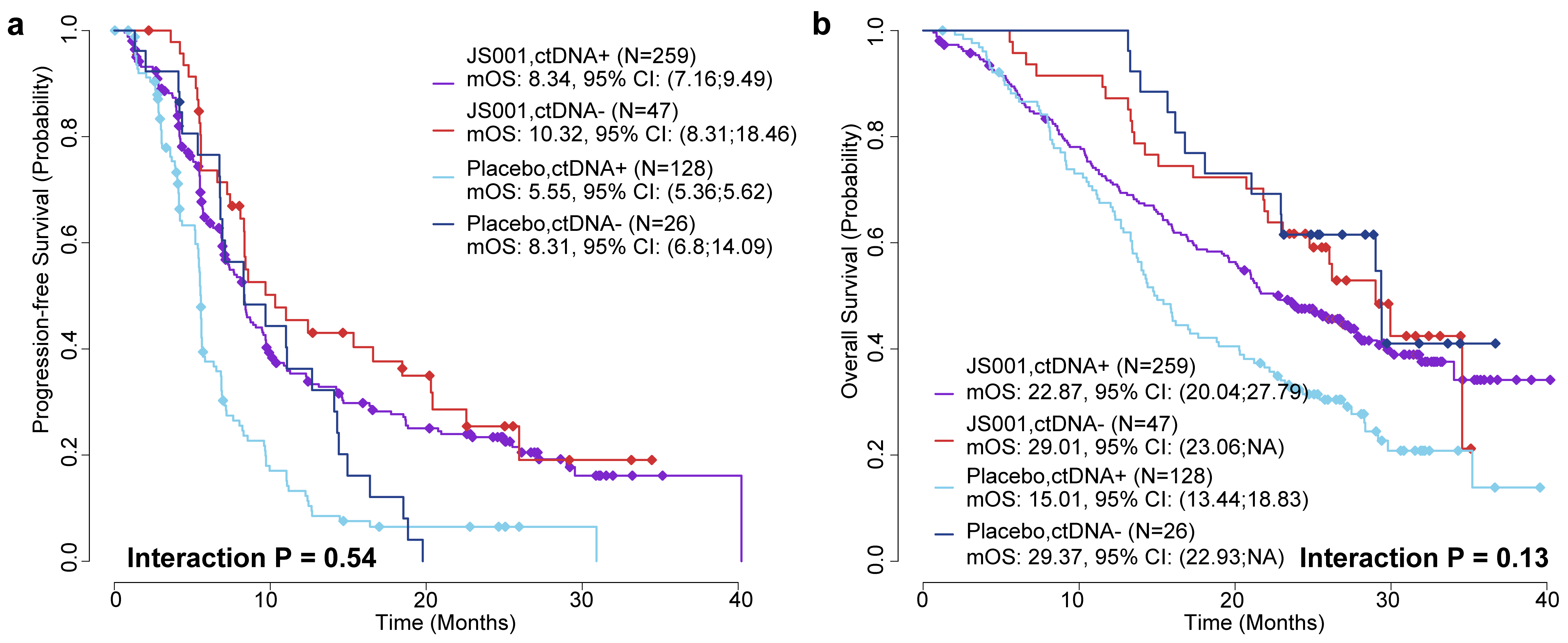
**

**Supplementary figure 4.** **The analyses of ctDNA status at baseline.** The absence of ctDNA at baseline is associated with better PFS **(Panel a)** and OS **(Panel b)**, but not predictive to toripalimab treatment. ctDNA, circulating tumor DNA, ITT, intention-to-treat.


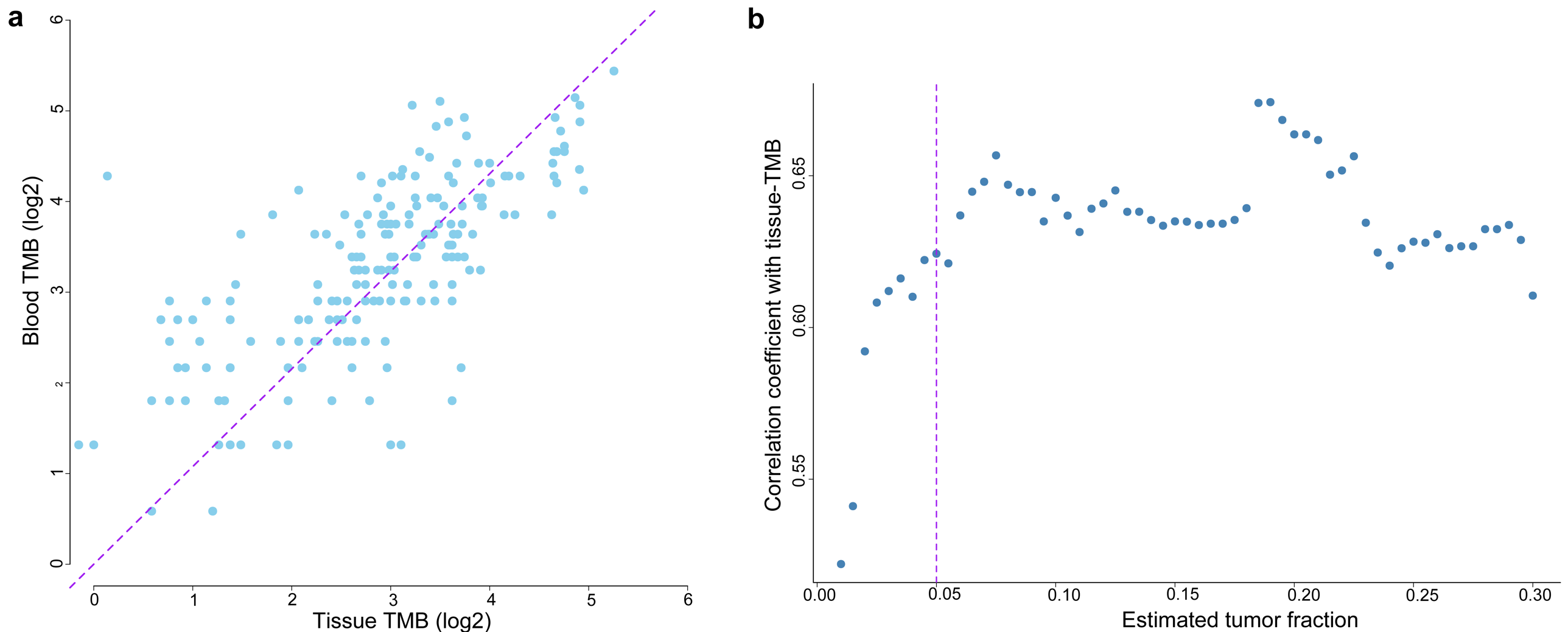


**Supplementary figure 5. The concordance between bTMB** **and tTMB**. The correlations between bTMB and tTMB with thresholds of ctDNA fractions 5% **(Panel a).** Samples with low ctDNA fraction often resulted in under-estimation of bTMB. When the tumor fraction increased from 0.5% to 5%, the correlation coefficients between bTMB and tTMB improved from 0.52 to 0.63. For the current study, the threshold of 5% tumor fraction was used to evaluate the significance of bTMB as a predictive biomarker for toripalimab treatment**(Panel b)**. bTMB, blood based tumor mutational burden; tTMB, tissue based tumor mutational burden.

**
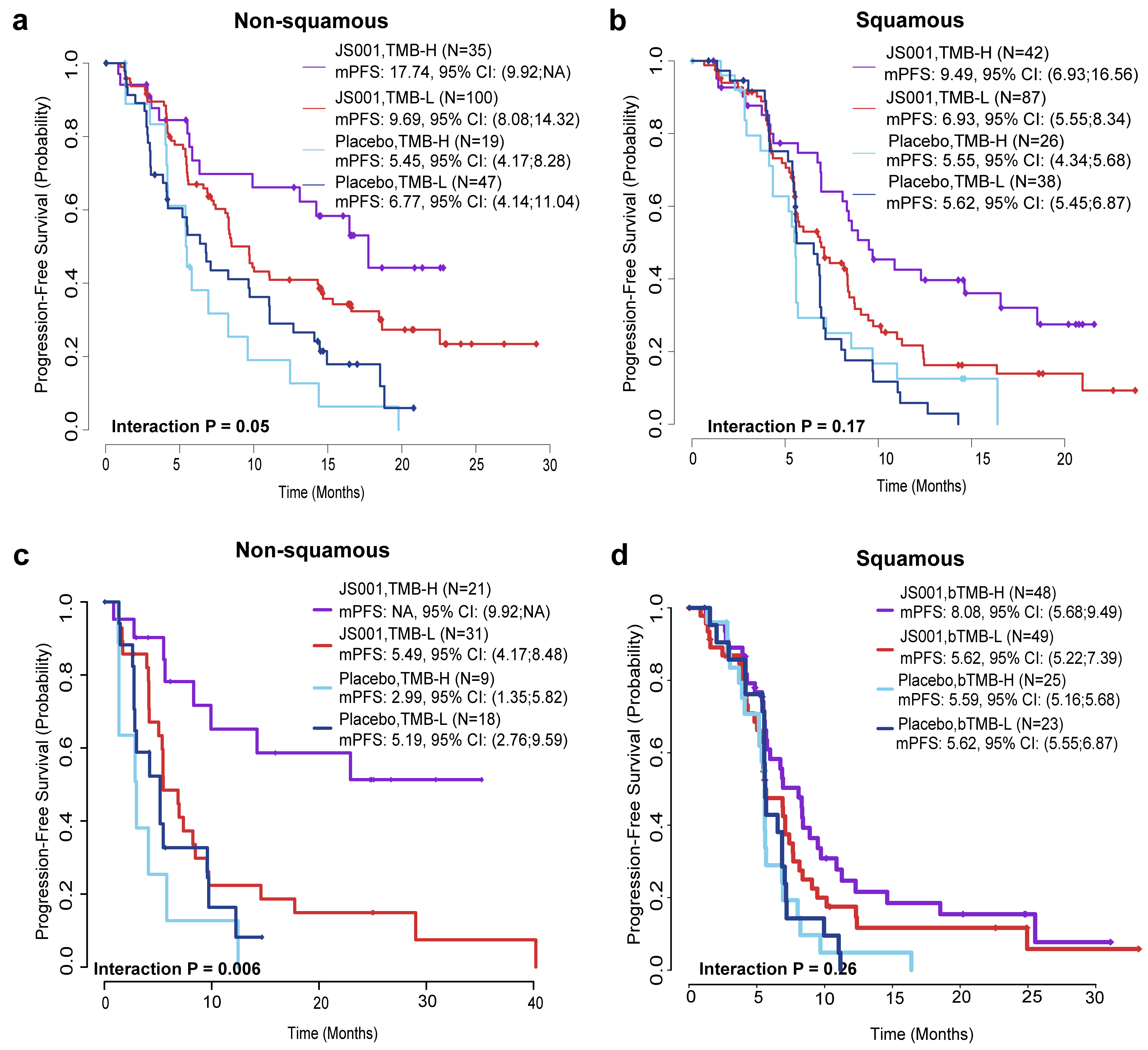
**

**Supplementary figure 6. Progression survival (PFS) between tissue-based or blood-based TMB in the histology subgroups** High tissue-based or blood-based TMB is associated with significantly longer PFS in the toripalimab group for patients with non-squamous NSCLC **(Panel a and c)**. The same threshold of 10muts/Mb was used to classify patients into TMB-High or TMB-low group. For patients with squamous NSCLC, the interactions between TMB and the treatment effect were not significant **(Panel b and d)**.

**
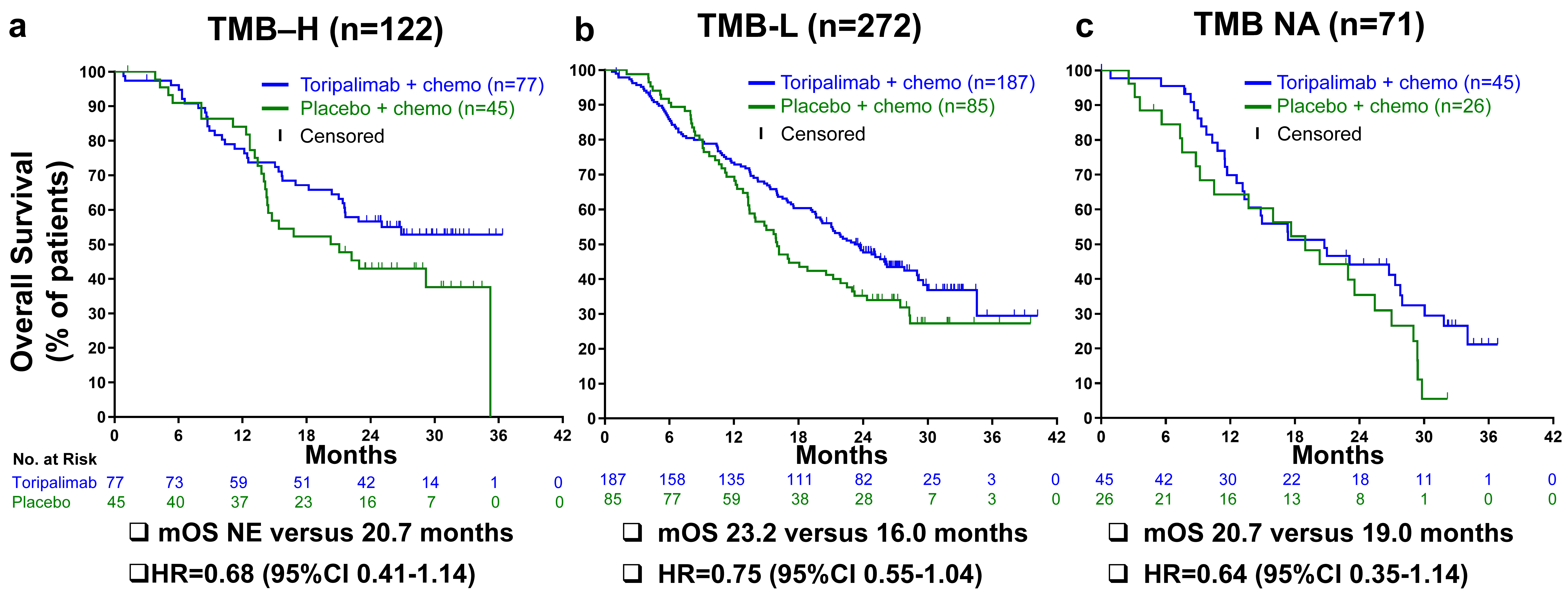
**

**Supplementary figure 7. Overall survival (OS) in TMB subgroups at the final OS analysis (Data Cutoff Date August 31, 2022).** Kaplan-Meier-estimated OS curves are shown to compare the toripalimab plus chemotherapy group with the placebo plus chemotherapy group in patients with TMB-H **(Panel a)** , TMB-L **(Panel b)** and TMB-NA**(Panel c)**. The threshold of high-TMB was 10 muts/Mbp. Censored patients are marked with “┃” in the graph. Numbers of patients at risk at indicated time points are shown below x-axis. The median OS and hazard ratio for OS are shown under the Kaplan-Meier curves. NA, Not applicable

**Supplementary figure 8.** **The predictive potency of blood-based ctDNA.** Gene Set Enrichment Analysis on the ctDNA data indicated the focal adhesion and IL-7 signaling pathways remained in the top enriched pathways **(Panel a)**. The Kaplan-Meier estimates of PFS stratified by ctDNA detected FA-PI3K-Akt pathway alterations**(Panel b)**. The Kaplan-Meier estimates of PFS stratified by ctDNA detected IL-7 pathway alterations**(Panel c)**. ctDNA, circulating tumor DNA; PFS, progression-free survival; ITT, intention-to-treat; FA-PI3K-Akt, focal adhesion-phosphatidylinositol 3-kinase-Akt signaling pathway (COL3A1/ COL6A3/ FLT1/ FLINC/ HGF/ IRS1/ IRS2/ ITGA4/ ITGA8/ KDR genes); IL-7, interleukin-7 signaling pathways (HGF/ IRS1/ IRS2/ SMARCA4).





**Supplementary figure 9.** **Associations of DNAH5/DSCAM alterations and TCB status with the treatment effects of PFS and OS.** The Kaplan-Meier estimates of PFS **(Panel a)** and OS **(Panel b)** stratified by DNAH5/DSCAM alterations in squamous NSCLC patients. The Kaplan-Meier estimates of PFS **(Panel c)** and OS **(Panel d)** stratified by TCB status in squamous NSCLC patients. Gene+: DNAH5 or DSCAM mutation; Gene-: DNAH5 and DSCAM wildtype; TCB+: Tumor copy-num burden ≥65% percentile; TCB-: Tumor copy-num burden <65% percentile.





**Supplementary figure 10**. **The analyses of ctDNA dynamics at C3D1 in the histology subgroups**. Reduction of ctDNA at C3D1 was associated with significantly better PFS and OS for patients with non-squamous NSCLC in the toripalimab group **(Panel a and Panel b)**. The interactions were not significant in patients with squamous NSCLC **(Panel c and Panel d).** Progression free survivals in ctDNA reduction subgroup stratified by treatment **(Panel e)**.


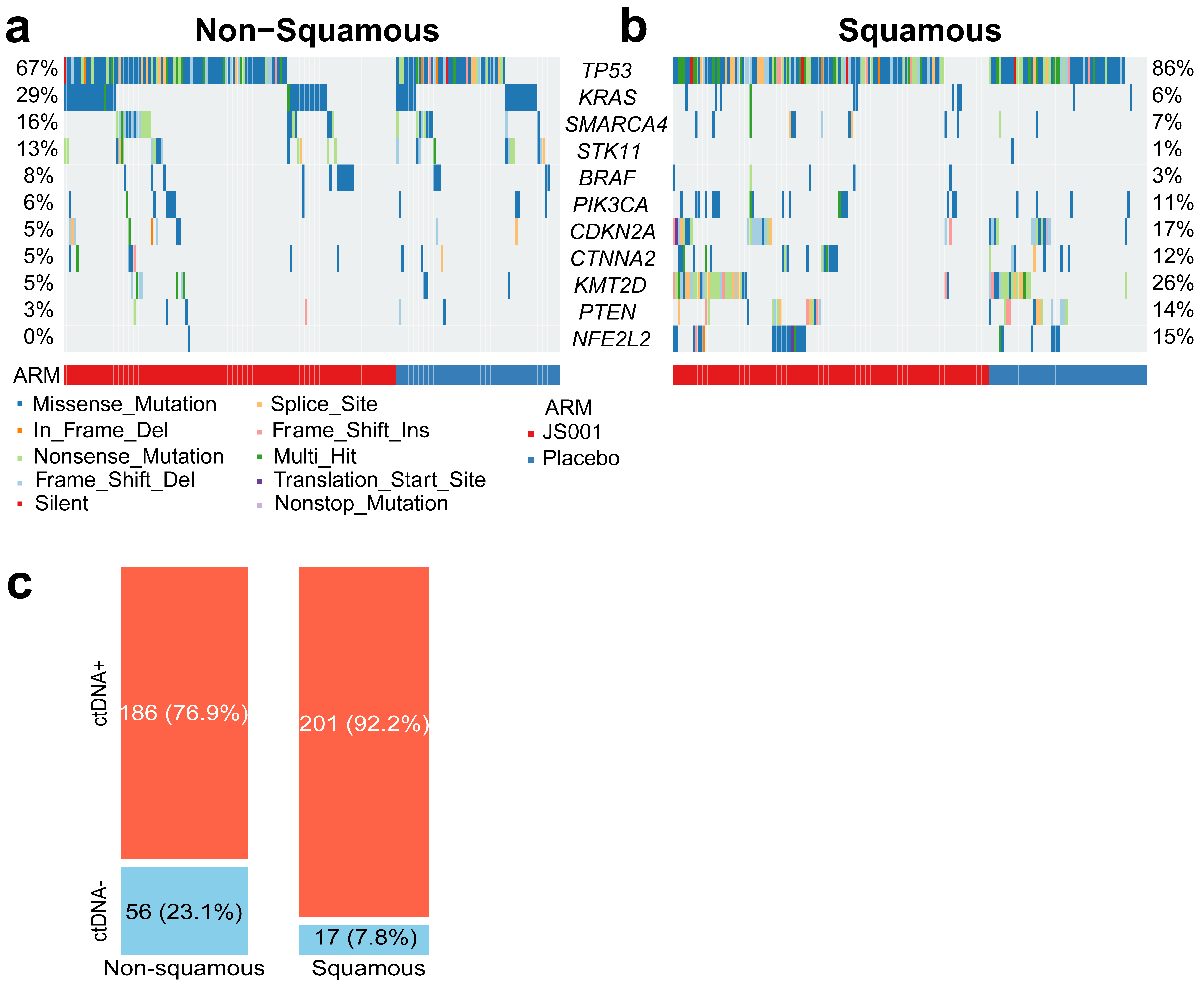


**Supplementary figure 11**. **Different genomic profile for squamous and non-squamous NSCLC.** The mutational patterns between non-SCC **(Panel a)** and SCC **(Panel b)** for the top mutated genes and baseline ctDNA positive rate in non-SCC and SCC **(Panel c).**

**Supplemental Methods**

**Treatment Procedures**

Patients with squamous NSCLC would receive nab-paclitaxel 100 mg/m^2^ IV on days 1, 8, 15 Q3W and carboplatin AUC 5 IV Q3W plus toripalimab 240 mg or placebo IV Q3W for 4-6 cycles during the chemotherapy phase. During the maintenance phase, patient will receive toripalimab 240 mg or placebo IV Q3W up to 2-year treatment.

Patients with non-squamous NSCLC would receive pemetrexed 500 mg/m^2^ IV and cisplatin 75 mg/m^2^ IV or carboplatin AUC 5 IV Q3W plus toripalimab 240 mg or placebo IV Q3W for 4-6 cycles during the chemotherapy phase. During the maintenance phase, patient will receive pemetrexed plus toripalimab 240 mg or placebo IV Q3W up to 2-year treatment.

**Blood ctDNA Processing and Sequencing**

Blood samples were centrifuged at 2000 g for 10 min at 4 °C at the lab and the supernatants were centrifuged at 16000g for 10 min at 4 °C, collected as plasma samples, and stored at -80 ℃. DNA was extracted from the plasma using QIAamp Circulating Nucleic Acid Kit (Qiagen, CA, USA) according to the manufacturer’s protocol. DNA quantification, library construction, sequencing, and data processing were performed. Briefly, for each sample, at least 50 ng of DNA was subjected to fragmentation, end repair, phosphorylation, dA addition, adaptor ligation, and purification. Capture-based targeted sequencing was performed with a panel of 520 cancer-related genes (OncoScreen Plus, covering a 1.86 MB region of the human genome, Burning Rock Biotech, Guangzhou, China) on the NextSeq 500 System (Illumina, Inc., San Diego, CA, USA). Genomic DNA from the paired peripheral blood mononuclear cells was also sequenced accordingly to identify germline variants. The raw sequencing data were trimmed and mapped to the human genome (hg19). After variant calling and annotation, mutations were filtered against common single nucleotide polymorphisms from the matched PBMC genomic DNA as well as in commonly used databases.

The ctDNA fraction was estimated as follows. The mutant allele fraction (MAF) and the ctDNA fraction were related, as MAF=(ctDNA*1)/([1-ctDNA]*2+ctDNA*1); therefore, ctDNA=2/([1/MAF]+1). Somatic mutations in genes with a detectable copy number amplification were omitted from ctDNA fraction estimates.
