# Supplementary material for: Toripalimab plus chemotherapy for first line treatment of advanced non-small cell lung cancer (CHOICE-01): final OS and biomarker exploration of a randomized, double-blind, phase 3 trial
Source: Signal Transduct Target Ther. 2024 Dec 24;9:369. doi: 10.1038/s41392-024-02087-6 (PMC11666711; doi:10.1038/s41392-024-02087-6)
Supplement: Supplementary file 2 — Supplement 2 [file 41392_2024_2087_MOESM2_ESM.docx]

| **Clinical Study Protocol** | |
| --- | --- |
| **Protocol title:** | A phase III, randomized, double-blind, placebo-controlled, multi-center clinical study to evaluate the efficacy and safety of Toripalimab Injection (JS001) or placebo combined with first-line standard chemotherapy in treatment-naive advanced non-small cell lung cancer (NSCLC) |
| **Study Protocol No.:** | JS001-019-III-NSCLC |
| **Investigational product:** | Toripalimab Injection (JS001) |
| **English name of investigational product:** | Toripalimab Injection |
|  |  |
| **Sponsor:** | Shanghai Junshi Biosciences Co., Ltd.  Floor 13, Building 2, Nos 36 and 58, Haiqu Road, Pudong New Area, Shanghai  P.C 201203 |
|  |  |
| **Protocol version:** | Version 4.0 |
| **Version date:** | June 24, 2020 |
|  |  |

Statement of Confidentiality

| This document contains confidential information of Shanghai Junshi Biosciences Co., Ltd. and is for use in this clinical study only. This document must not be disclosed to anyone other than the study staff and members of the institutional review board. Without the written approval by Shanghai Junshi Biosciences Co., Ltd., this information can not be used for any objective except evaluation or conduction of the clinical study. |
| --- |

Sponsor’s signature page

| Protocol title: | A phase III, randomized, double-blind, placebo-controlled, multi-center clinical study to evaluate the efficacy and safety of Toripalimab Injection (JS001) or placebo combined with first-line standard chemotherapy in treatment-naive advanced non-small cell lung cancer (NSCLC) |
| --- | --- |
| Protocol Number: | JS001-019-III-NSCLC |
| Version number and version date： | Version 4.0, June 24, 2020 |

| Sponsor | Shanghai Junshi Biosciences Co., Ltd.  Address: Floor 13, Building 2, Nos 36 and 58, Haiqu Road, Pudong New Area, Shanghai  Telephone: 021- 20248288 |
| --- | --- |
| Representative of clinical study team | Name: Yongyong SHI  Address: Room 1705, Floor 17, Shijihui Office Building 2, No 1196, Century Avenue, Pudong New District, Shanghai  Telephone: (021)80164690-65109  Email: yongyong_shi@junshipharma.com |
| Medical supervisor(s) | Name: Yanyan WANG  Address: Room 1705, Floor 17, Shijihui Office Building 2, No 1196, Century Avenue, Pudong New District, Shanghai  Telephone: (021) 80164690-65203  Mail:yanyan_wang@ junshipharma.com |

Shanghai Junshi Biosciences Co., Ltd.

Medical Executive Director, Clinical Study Principal Signature Date

Xiaoyan KANG

Investigator’s Statement

I have read the clinical study protocol entitled “A phase III, randomized, double-blind, placebo-controlled, multi-center clinical study to evaluate the efficacy and safety of Toripalimab Injection (JS001) or placebo combined with first-line standard chemotherapy in treatment-naive advanced non-small cell lung cancer (NSCLC)” (version number: 4.0, dated June 24, 2020) and agree to conduct this clinical study in accordance with all the clauses specified in the study protocol, current regulations and the ethical principle in the Declaration of Helsinki.

I agree to comply with the guideline of Good Clinical Practice and other applicable NMPA regulations/guidelines.

I agree that the confidential information contained in this document shall not be used for other purposes except the evaluation or conduction of the clinical study, without prior written approval by Shanghai Junshi Biosciences Co., Ltd.

| **Principal Investigator Name:** | **(in Chinese calligraphy of regular script)** | |  |
| --- | --- | --- | --- |
| **Signature:** |  | **Date: DD MM YYYY** | |

Contract Research Organisation (CRO) Signature Page

| Protocol title: | A phase III, randomized, double-blind, placebo-controlled, multi-center clinical study to evaluate the efficacy and safety of Toripalimab Injection (JS001) or placebo combined with first-line standard chemotherapy in treatment-naive advanced non-small cell lung cancer (NSCLC) |
| --- | --- |
| Protocol Number: | JS001-019-III-NSCLC |
| Version number and version date： | 4.0, June 24, 2020 |

I have read the clinical study protocol entitled “A phase III, randomized, double-blind, placebo-controlled, multi-center clinical study to evaluate the efficacy and safety of Toripalimab Injection (JS001) or placebo combined with first-line standard chemotherapy in treatment-naive advanced non-small cell lung cancer (NSCLC)” (version number: 4.0, dated June 24, 2020) and agree to perform the relevant responsibilities in accordance with all the clauses specified in the study protocol, Chinese GCP regulations, and current laws & regulations.

**Project leader**

|  |  |  |
| --- | --- | --- |
| Printed name |  | Position |
|  |  |  |
| Signature |  | Date |

Organization of the clinical trial

| **Sponsor** | Shanghai Junshi Biosciences Co., Ltd.  Suzhou Zhonghe Biosciences Co., Ltd.  Telephone: 021-61040009 Fax:021-20423282 |
| --- | --- |
| **Sponsor Contact** | Xiaoyan KANG  Contact number: 021-80164690/18616892591 |
| **Clinical Study Leading Unit** | Cancer Hospital Chinese Academy of Medical Sciences |
| **Leading Principal Investigator** | Professor Jie WANG  Contact number:010-87788495/13910704669 |
| **Contract Research Organization (CRO)** | Beijing Halma Orient Medicine Technology Co. Ltd  Telephone: 010-82255819 Fax: 010-82255829 |
| **Data Management and Statistical Unit** | Nanjing Ackerman Information Technology Co., Ltd. |
| **Pharmacokinetics Analysis Unit** | United-Power Pharma Tech Co., Ltd. |
| **Clinical Central Laboratory biological sample analyst (blood/tissue)** | Maijie Translational Medicine (Suzhou) Co., Ltd.  OrigiMed (Shanghai) Co., Ltd. |

Protocol Synopsis

| **Title** | A phase III, randomized, double-blind, placebo-controlled, multi-center clinical study to evaluate the efficacy and safety of Toripalimab Injection (JS001) or placebo combined with first-line standard chemotherapy in treatment-naive advanced non-small cell lung cancer (NSCLC) |
| --- | --- |
| **Protocol No.** | JS001-019-III-NSCLC |
| **Study phase** | Phase III |
| **Trial duration** | March 2019 to June 2021 |
| **Study objectives** | **Primary Objective**   - To evaluate progression-free survival (PFS) of Toripalimab injection (JS001) versus placebo combined with standard 1st-line chemotherapy for treatment-naive advanced non-small cell lung cancer.   **Secondary Objectives**   - To evaluate the overall survival (OS), objective response rate (ORR) by RECIST1.1 criteria, duration of response (DOR), disease control rate (DCR) and time to response(TTR) of Toripalimab injection (JS001) versus placebo combined with standard 1st-line chemotherapy for treatment-naive advanced non-small cell lung cancer; - To evaluate the safety and tolerability of JS001 versus placebo combined with standard first-line chemotherapy for treatment-naive advanced non-small cell lung cancer;   **Exploratory Objectives**   - To evaluate PFS, ORR, DOR, DCR and TTR of Toripalimab injection (JS001) versus placebo combined with standard 1st-line chemotherapy for treatment-naive advanced non-small cell lung cancer based on iRECIST criteria; - To evaluate the immunogenicity of JS001, and explore the potential relationship between its immunogenic response and the safety and efficacy; - To explore the population with the best predictive efficacy through biomarker analysis (including but not limited to PBMC, PD-L1 IHC, WES, RNASeq and others). |
| **Study Endpoints** | **Primary endpoint**   - Progression free survival (PFS) evaluated by investigators according to the response evaluation criteria in solid tumors (RECIST 1.1).   **Secondary endpoints**   - Overall survival (OS); - PFS evaluated by the Blinded Individual Review Committee (BIRC) based on RECIST1.1 criteria; - Objective response rate (ORR) and duration of response (DOR) evaluated by investigators and BIRC based on RECIST1.1; - Disease control rate (DCR) evaluated by investigators and BIRC based on RECIST1.1; - Time to response (TTR) evaluated by investigators and BIRC based on RECIST1.1; - Safety: overall incidence of adverse events (AEs); incidence of grade 3 and above AEs; incidence of serious adverse events (SAEs); incidence of AEs leading to termination of the investigational drug; incidence of AEs leading to interruption of the investigational drug;   **Exploratory endpoints:**   - PFS, ORR, DOR, DCR and TTR based on irRECIST criteria; - To evaluate the immunogenicity of JS001, and explore the potential relationship between its immunogenic response and the safety and efficacy; - To analyze potential biomarkers related to the efficacy on a exploratory basis. |
| **Study Design** | **Overall design:**  This is one randomized, double-blind, placebo-controlled, multi-center, phase III clinical study to evaluate the efficacy and safety of Toripalimab injection (JS001) or placebo combined with standard 1st-line chemotherapy in treatment-naïve advanced non-small cell lung cancer (NSCLC); and evaluate the population with the best predictive biomarkers, i.e., positive diagnosis population.  About 450 subjects with advanced non-small cell lung cancer without activated EGFR mutation and ALK fusion will be 2:1 randomized into two groups, JS001 combined with the standard 1st-line chemotherapy will be given in the study group whereas placebo combined with standard 1st-line chemotherapy will be given in the control group.  The stratification will be based on the following factors:   - PD-L1 expression (TC ≥ 1% vs TC < 1%);   Note: The patients not evaluable for PD-L1 will be included in TC < 1% group.   - Smoking state (often smoking vs no smoking or infrequent smoking);   Note: Smoking state is expressed by smoking index, smoking index = number of cigarettes smoked per day × number of years of smoking, often smoking is defined as ≥ 400 cigarettes per year.   - Pathological type (squamous cell carcinoma vs non-squamous cell carcinoma).   Note: The patients with adenosquamous cell carcinoma will be stratified by squamous cell carcinoma and treated with a chemotherapy regimen of nab-paclitaxel combined with carboplatin. 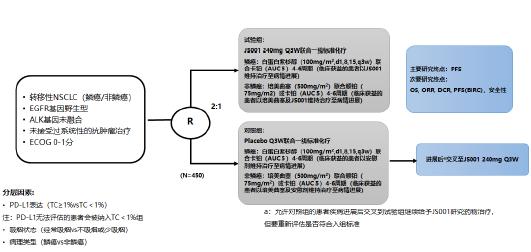  - Metastatic NSCLC, squamous or non-squamous - EGFR wild-type - No ALK fusion - No prior systemic anti-tumor therapy - ECOG score 0-1   **Stratification factors:**   - PD-L1 expression (TC ≥ 1% vs TC < 1%)   Note: The patients not evaluable for PD-L1 will be included in TC < 1% group.   - Smoking state (often smoking vs no smoking or infrequent smoking) - Pathological type (squamous cell carcinoma vs non-squamous cell carcinoma)   **Experimental group:**  **JS001 240mg Q3W plus 1st standard chemotherapy**  **Squamous carcinoma: Albumin-bound paclitaxel (100mg/m^2^, d1, 8, 15, q3w) + carboplatin (AUC 5) for 4-6 cycles, the patients with clinical benefit will receive maintenance treatment of JS001 until progressive disease.**  **Non-squamous carcinoma: Pemetrexed (500 mg/m^2^) + cisplatin (75 mg/m^2^) or carboplatin (AUC 5) for 4-6 cycles, the patients with clinical benefit will receive maintenance treatment of pemetrexed + JS001 until progressive disease.**  **Control group:**  **Placebo Q3W plus 1st standard chemotherapy**  **Squamous carcinoma: Albumin-bound paclitaxel (100mg/m^2^, d1, 8, 15, q3w) + carboplatin (AUC 5) for 4-6 cycles, the patients with clinical benefit will receive maintenance treatment of placebo until progressive disease.**  **Non-squamous carcinoma: Pemetrexed (500 mg/m^2^) + cisplatin (75 mg/m^2^) or carboplatin (AUC 5) for 4-6 cycles, the patients with clinical benefit will receive maintenance treatment of pemetrexed + placebo until progressive disease.**  **Primary study endpoint: PFS**  **Secondary study endpoints: OS, ORR, DCR, PFS (BIRC), safety**  **Cross over to JS001 240mg Q3W after progressive disease ^a^**  a: The subjects in the control group are allowed to cross over to experimental group to receive JS001 study treatment after progressive disease, but their eligibility shall be re-assessed.  Patients will receive the following treatment:   - **JS001/Placebo**   JS001 240mg/placebo is given intravenously in a cycle of 21 days, the treatment will continue until the subject reaches the criteria on discontinuation of the drug, i.e., documented progressive disease, unacceptable adverse events (AEs), not suitable to continue treatment as considered by investigators, withdrawal of informed consent by the subject, the subject has received JS001 treatment for an accumulative period of 2 years or other reasons as specified in the protocol.  When progressive disease occurs based on RECIST1.1 criteria, unblinding may be performed according to investigator’s judgment:   - Subjects who have received placebo + chemotherapy combination therapy (control group), if meeting the crossover treatment criteria, can receive JS001 crossover treatment after obtaining informed consent for crossover treatment, i.e., intravenous infusion of JS001 240 mg, 21 days as a cycle until they reach the criteria on discontinuation of the drug, for up to 2 years. At that time, the tumor image will be re-baselined. After receiving JS001 alone crossover therapy, imaging examinations will be performed every 9 weeks (±7 days). After occurrence of progressive disease defined by the RECIST v1.1 criteria, if the investigator believes that the clinical benefit can still be obtained from the treatment, after obtaining the subject’s signed informed consent on continuation of medication after progressive disease, the clinically stable patients can continue treatment until progressive disease confirmed by iRECIST (iCPD); - Subjects who have received JS001 + chemotherapy combination therapy (experimental group), if the investigator believes that the clinical benefit can still be obtained from the treatment, and after obtaining the subject’s signed informed consent on continuation of medication after progressive disease, can continue to be given JS001 monotherapy (terminate chemotherapy at the same time). Within 4-8 weeks, the investigator will confirm PD according to iRECIST criteria. If there is confirmed progressive disease (iCPD), it is recommended to discontinue JS001 treatment. The subjects will receive JS001 treatment for an accumulative period of up to 2 years (including combined chemotherapy and monotherapy).   Or, when the investigator confirms progressive disease according to the RECIST1.1 criteria, if the subjects remain clinically stable, they can still remain blinded to the assigned treatment, and after obtaining the subject’s signed informed consent to continue medication after progressive disease, they may continue to receive the trial treatment according to randomly assigned treatment. Within 4-8 weeks, the investigator will confirm PD according to the iRECIST criteria. Upon confirmed iCPD, the patients can be unblinded. The control group will undergo JS001 cross-treatment according to the above procedure (in line with the cross-treatment criteria), and the experimental group is recommended to terminate the current treatment.   - **First-line standard chemotherapy**   **Patients with squamous cell carcinoma will receive the following chemotherapy regimen:**  Albumin-bound paclitaxel + carboplatin: albumin-bound paclitaxel 100mg/m^2^ iv, d1, 8, 15 (dosing of albumin-bound paclitaxel injection on day 15 will be at the discretion of the investigator), carboplatin AUC 5 iv on day 1 of each 21-day cycle, for up to 4-6 cycles.  **Patients with non-squamous cell carcinoma will receive the following induction chemotherapeutic regimen for treatment:**  Pemetrexed + cisplatin: Pemetrexed 500mg/m^2^ and cisplatin 75mg/m^2^ via IV infusion on Day 1 of each 21-day cycle, for up to 4-6 cycles.  Or, Pemetrexed + carboplatin: Pemetrexed 500mg/m^2^ and carboplatin AUC 5 via IV infusion on Day 1 of each 21-day cycle, for up to 4-6 cycles.  The patients without progressive disease after 4-6 cycles of therapy can continue to receive Pemetrexed alone for maintenance therapy.  Cisplatin or carboplatin will be given at the discretion of the investigator.   - **Tumor assessments:**   Regardless of delayed dose, the tumor will be evaluated using RECIST 1.1 and iRECIST criteria at baseline and once every 6 weeks in the first 12 months after cycle 1 day 1. Twelve (12) months later, tumor evaluation shall be performed once every 9 weeks. The patients who have received JS001 alone crossover therapy after unblinding (patients in the original control group) will undergo tumor evaluation every 9 weeks. The patients will receive tumor evaluation until radiological progressive disease defined by RECIST 1.1 or iRECIST criteria, patient death, withdrawal of informed consent form, sponsor’s termination of the study, or other reasons as specified in the protocol, whichever comes first. The patients who terminate treatment for the reasons (e.g., toxicity) other than radiological progressive disease will continue to undergo tumor evaluation as scheduled, until radiological progressive disease defined by RECIST 1.1 or iRECIST criteria, initiation of new anti-tumor treatment, death, withdrawal of informed consent form, sponsor’s termination of the study, or other reasons as specified in the protocol, whichever comes first.  All the radiological data for tumor evaluation will be collected by the sponsor or its designated CRO, and reviewed independently by the Blinded Individual Review Committee (BIRC) using RECIST 1.1, including blinded, central and independent review of computed tomography (CT) scan findings. The decision of the above review will be made prior to the analysis of primary efficacy endpoint in the main study.   - **Acquisition of tumor tissue specimen:**   During the screening period, formalin-fixed fresh tissue samples, paraffin-embedded tumor tissue blocks, or at least 12 unstained tumor specimen sections must be provided (if approved by the sponsor’s medical monitor, less than 12 unstained sections can be submitted) for biomarker evaluation and detection.  If the tumor lesion can be obtained when the efficacy is evaluated as partial response (PR) and/or progressive disease (PD), it is encouraging for the subjects to participate in the optional biomarker study in this study. If pseudo-progression is suspected, it is advisable to conduct biopsy of the lesion for confirmation of diagnosis if the condition allows.  Biomaker analyses will be conducted in this study, which will be performed for archived tissue specimens (requirement at enrollment), and prospectively collected blood samples and freshly acquired tumor samples. The samples collected will be analyzed for PD-L1 expression, TMB, DDR (by NGS/WES) and other potential markers in the central laboratory designated by the sponsor, as for evaluation of the expression of biomarkers and patient’s outcome, including but not limited to the correlation between response and progressive disease.   - **Immunogenicity**   3 ml blood sample will be collected to evaluate the anti-drug antibody (ADA) and corresponding trough concentration of JS001. |
| **End of study** | The end time of the study is defined as the date of the last patient last visit (LPLV), or the date of collection of the last data required for statistical analysis, whichever comes first. The last data required for statistical analysis will be described detailedly in the Statistic Analysis Plan. The sponsor has the right to decide to end this study at any time due to specific reasons (such as major safety issues, force majeure, etc.). Duration of the study is estimated to be about 27 months from randomization of the first subject. |
| **Inclusion criteria** | **Only the patients meeting all the following criteria can be eligible to participate in the trial:**  1. Having sufficient understanding of this study and being willing to sign the informed consent form (ICF);  2. Histologically and/or cytologically confirmed, unresectable locally advanced (phase IIIB or IIIC) and stage IV non-small cell lung cancer (in accordance with AJCC 8th edition) not suitable for radical concurrent radio-chemotherapy, without activated EGFR mutation and ALK fusion; this test is not mandatory for the patients with squamous cell carcinoma;  3. At least one measurable lesion (in accordance with RECIST 1.1);  Note: the lesion previously irradiated cannot be regarded as a target lesion, unless definite progressive disease occurs after radiotherapy.  4. No history of any systemic anti-tumor therapy. If the adjuvant/neoadjuvant therapy is completed at least 6 months prior to the occurrence of metastasis, the subject receiving the adjuvant/neoadjuvant therapy meets the condition;  5. Agreement on providing formalin fixed tumor tissue specimen or fresh biopsy tissue from tumor lesions after diagnosis of metastasis, for at least 12 sections (if approved by the sponsor’s medical monitor, less than 12 unstained sections can be submitted); if recent biopsy is not feasible, the biopsy sample obtained before adjuvant/neoadjuvant chemotherapy can be accepted (i.e., archived specimen);  6. Age 18-75 years, male or female;  7. ECOG Scores 0-1;  8. Expected survival ≥ 3 months;  9. Laboratory test values within 7 days before the first dose must meet the following criteria:  a) Neutrophils ≥ 1.5 × 10^9^/L;  b) Platelets ≥ 100 × 10^9^/L;  c) Hemoglobin ≥ 90g/L (no transfusion of concentrated red blood cell within 4 weeks);  d) Serum creatinine ≤ 1.5 × upper limit of normal (ULN) and creatinine clearance ≥ 50 mL/min;  e) Total serum bilirubin ≥ 1.5 × ULN;  f) Aspartate aminotransferase (AST) and alanine aminotransferase (ALT) ≤ 2.5 × ULN; ALT and AST ≤ 5 × ULN for patients with hepatic metastasis; alkaline phosphatase (ALP) ≤ 2.5 × ULN, or ≤ 5 × ULN in patients combined with tumor bone metastasis;  g) International normalized ratio (INR) or prothrombin time (PT) ≤ 1.5 × ULN, unless the subject is receiving anticoagulation therapy; activated partial thrombin time (aPTT) ≤ 1.5 × ULN, unless the subject is receiving anticoagulation therapy;  10. Women of childbearing potential must have a confirmed negative result of serum pregnancy test and agree to use effective contraceptive measures during the use of study drug and within 90 days after the last dose. Women of childbearing potential in this protocol are defined as sexually mature women: 1) no history of hysterectomy or bilateral oophorectomy, 2) natural menopause for not more than 12 consecutive months (fertility cannot be excluded in case of amenorrhoea following cancer therapy) (i.e., menses occurred at any time within the previous consecutive 12 months); if male subjects' female parters are of childbearing potential, the subjects must agree to use adequate contraceptive measures from start of the first dose of study treatment to 90 days after the last dose of study treatment. |
| **Exclusion criteria** | **Patients who fulfill any of the following criteria must be excluded from the study:**  1. Known allergy to recombinant humanized anti-PD-1 monoclonal antibody drug and its components;  2. Histologically or cytopathologically confirmed combination with small cell lung cancer component or sarcomatoid lesion;  3. Current participation in and receiving other study treatment, or participation in treatment of one study drug within 4 weeks prior to administration of JS001;  4. Previous use of systematic chemotherapy for advanced NSCLC; targeted therapy for advanced NSCLC (including but not limited to erlotinib, crizotinib, cetuximab);  5. Previous use of anti-PD-1 antibody, anti- PD-L1 antibody, anti- PD-L2 antibody or anti- CTLA-4 antibody (or any other antibody acting on T cells synergetic stimulation or checkpoint pathway, such as IDO, IL-2R, GITR);  6. Chest (lung) radiotherapy > 30 Gy within 6 months prior to the start of study treatment. However, the locally palliative radiotherapy for bone metastasis is excluded;  7. Use of traditional Chinese medicine with antitumor indication within 2 weeks prior to the first dose, or use of the drugs with immunomodulatory effect (including thymosin, interferon and interleukin, etc.), or have undergone major surgery within 3 weeks prior to the first dose or have not fully recovered from previous surgery;  8. Patient with active tuberculosis (TB), receiving anti-tuberculosis therapy currently or within 1 year prior to screening;  9. Subjects with active or untreated central nervous system (CNS) metastasis;  Note: If the subjects’ CNS tumor metastasis is limited to the supratentorial area and/or cerebellum, has received adequate treatment, and clinical stability (enhanced MRI or CT is preferred for imaging testing) has been maintained for at least 4 weeks, and the subjects’ clinical symptoms of nervous system and other parts can recover to NCI-CTC AE ≤ grade 1 at least 2 weeks prior to the first dose, they can participate in the study. If a new asymptomatic CNS metastasis is detected on scans during the screening period, the subjects must receive radiotherapy and/or surgery for CNS metastases. After the end of treatment in the above patients, there is no need to receive the additional brain scanning before enrollment if all the other criteria are met.  10. Spinal cord compression for which operation and/or radical radiotherapy has not been given, or no clinical evidence of stable disease for ≥ 4 weeks prior to enrollment after treatment for previously diagnosed spinal cord compression;  11. Uncontrolled pleural effusion, pericardial effusion, or ascites requiring repeated drainage (once per month or more frequently); subjects with stable symptoms for at least two weeks upon drainage can be enrolled;  12. Uncontrollable or symptomatic hypercalcemia (ionized calcium > 1.5mmol/L or calcium > 12mg/dL or corrected serum calcium > ULN);  13. Clinically uncontrolled active infection, including but not limited to acute pneumonia;  14. Uncontrollable major epileptic seizure or superior vena cava syndrome;  15. Previous or current combination with other malignancies (except non-melanoma radically treated and of no recurrence evidence, including skin basal cell carcinoma or squamous cell carcinoma, breast/cervical carcinoma in situ, superficial bladder cancer and other carcinomas in situ);  16. History of interstitial pneumonia, idiopathic pulmonary fibrosis, organized pneumonia (e.g., obliterating bronchiolitis), drug induced pneumonia, idiopathic pneumonia or evidence of active pneumonia during chest CT scanning for screening, or other moderate to severe lung diseases that severely affect lung function;  17. Known hepatic diseases of clinical significance, including untreated active viral hepatitis, alcoholic hepatitis or other hepatitis, liver cirrhosis, hereditary liver disease;  Note 1: Active viral hepatitis is defined as hepatitis B virus (HBV) infection with hepatitis B virus deoxyribonucleic acid (HBV DNA) higher than the lower limit of detection; or hepatitis C virus (HCV) infection (positive anti-HCV antibody and the quantitative detection results of HCV RNA higher than the lower limit of detection);  Note 2: Patients with recovered previous hepatitis B virus (HBV) infection or HBV infection (defined as positive hepatitis B core antibody [HBcAb] and negative HBsAg) can participate in this study. Prior to enrollment, HBV DNA detection must be performed for such patients (patients can be enrolled only when HBV DNA is lower than the lower limit of detection);  Note 3: For patients with HBsAg positive and HBV DNA less than the lower limit of detection, the investigator will comprehensively assess the risk. If necessary, the patients should be regularly assessed and received anti-HBV virus treatment throughout the study treatment period;  Note 4: Patients with positive HCV antibody test results can be enrolled in this study only when the test of polymerase chain reaction of HCV RNA is negative;  18. Known human immunodeficiency virus (HIV) infection (known positive HIV antibody);  19. Serious cardiovascular disease, for example, New York Heart Association (NYHA) > grade 2 heart failure, unstable angina pectoris, unstable arrhythmia, myocardial infarction or cerebrovascular accident within 6 months prior to randomization;  20. Use of systemic immunosuppressive therapy (i.e., use of corticosteroid or immunosuppressant) for any active autoimmune disease within 2 years prior to Day 1 of the 1st cycle;   1. The autoimmune diseases include but are not limited to interstitial pneumonia, uveitis, enteritis, hepatitis, hypophysitis, nephritis, hyperthyroidism; 2. Patients with leukoderma or childhood asthma that has been completely relieved and does not need any intervention in adulthood can be enrolled; 3. Patients receiving replacement therapy (e.g., thyroxine, insulin or physiological corticosteroid replacement therapy for adrenal/pituitary insufficiency) are eligible to participate in the study; 4. Patients who need to use bronchodilator, inhaled steroid or local steroid injection intermittently for asthma will not be excluded from the study.   21. Being unable or unwilling to use folic acid or vitamin B_12_ injection (applicable for patients with non-squamous cell carcinoma who plan to receive pemetrexed treatment);  22. Vaccination of live-virus vaccine within 30 days after the start of planned treatment; use of seasonal influenza vaccine free of live virus is allowed;  23. Patients with previous allogeneic stem cell or solid organ transplantation.  24. Women who are pregnant or at lactation or have the potential of pregnancy show positive pregnancy test prior to the first dosing; patients who have childing-bearing potential but are not willing to receive contraception measures or whose sex partners are not willing to receive contraception measures;  25. Any other disease or condition of clinical significance that can affect the compliance with the protocol (e.g., history of psychosis or drug abuse), cannot benefit from this clinical study, or affect the signature of the informed consent form (e.g., drug addiction and drug abuse), or is unsuitable to be involved in this clinical trial, as considered by investigators (including but not limited to: abnormal laboratory results, clinically active diverticulitis, intra-abdominal abscess, intestinal obstruction, peritoneal metastatic cancer);  **Eligibility criteria on crossover to JS001 treatment period for control group:**  1. As evaluated by investigators, the subjects must have clear radiological progressive disease documented during placebo combined with standard 1st-line chemotherapy or during maintenance treatment;  2. The subjects have received no other systemic anticancer therapy other than the chemotherapy drugs in this study, and the previous anticancer therapy, including chemotherapy and palliative radiotherapy, must be completed at least 3 weeks prior to the administration of JS001;  3. The adverse events related to chemotherapy or pallative radiotherapy must have been relieved to grade 1 or baseline at the entry in crossover treatment;  4. Any major surgery must be completed at least 28 days prior to the first dose of JS001;  5. No previous anti-PD-1 or anti- PD-L1 treatment;  6. ECOG PS 0-1;  7. The laboratory data on the eligibility of crossover therapy must meet the following criteria and should be obtained within 14 days prior the start of JS001 treatment:  Peripheral hemogram: white blood cell (WBC) ≥ 3.0 × 10^9^/L, neutrophil (ANC) ≥ 1.5 × 10^9^/L, platelet (PLT) ≥ 100 × 10^9^/L, hemoglobin (Hgb) ≥ 90g/L;  Renal function: serum creatinine (Cr) level ≤ 1.5 × ULN or calculated creatinine clearance (CrCl) > 40 mL/min (using Cockcroft Gault formula);  Hepatic function: AST/ALT ≤ 2.5 × ULN in subjects without hepatic meatastasis, AST/ALT ≤ 5 × ULN in subjects with hepatic metastasis; Total bilirubin ≤ 1.5 × ULN (except subjects with Gilbert's syndrome for whom total bilirubin must be < 3.0 mg/dL).  8. Men of reproductive ability or women of pregnant potential must use highly effective contraceptive methods (e.g. oral contraceptives, intrauterine contraceptive device, abstinence of sexual intercourse or barrier contraception in combination with spermatocide) during the trial, and continue contraception for 90 months after the end of treatment;  9. Being voluntary to participate in the study, sign the informed consent form, with good compliance and willingness to cooperate with follow-up. |
| **Investigational product, dosage and administration method** | **Induction period**  **Drug A:** Toripalimab injection (JS001) or placebo  Specifications: JS001: 240mg/6ml/vial; placebo: 6ml/vial  Administration method: JS001 fixed-dose 240 mg or placebo 6ml intravenous drip, every 3 weeks, on day 1 of each cycle. The chemotherapy may be initiated after the end of close monitoring of each vital sign, 1 hour after the end of intravenous drip of JS001.  **Drug B:** Pemetrexed freeze-dried powder  Administration method: the dose administered will be calculated based on body surface area, 500mg/m^2^ iv drip, once every three weeks, on Day 1 of each course of therapy.  **Drug C:** Albumin-bound Paclitaxel Injection  Administration method: the dose administered will be calculated based on body surface area, 100mg/m^2^ iv drip, d1, 8, 15, once every week (dosing of albumin-bound paclitaxel injection on day 15 will be at the discretion of the investigator).  **Drug D:** Carboplatin Injection  Administration method: AUC 5, iv drip, once every three weeks in induction therapy period, on Day 1 of each course of therapy.  **Drug E:** Cisplatin Injection  Administration method: the dose administered will be calculated based on body surface area, 75mg/m^2^ iv drip, once every three weeks, on Day 1 of each course of therapy.  **Maintenance period**  **Drug A:** Toripalimab injection (JS001) or placebo  Specifications: JS001: 240mg/6ml/vial; placebo: 6ml/vial  Administration method: JS001 fixed-dose 240 mg or placebo 6ml intravenous drip, every 3 weeks, on day 1 of each cycle. The chemotherapy may be initiated after the end of close monitoring of each vital sign, 1 hour after the end of intravenous drip of JS001. The subjects will receive JS001 treatment for an accumulative period of up to 2 years.  **Drug B:** Pemetrexed freeze-dried powder  Administration method: the dose administered will be calculated based on body surface area, 500mg/m^2^ iv drip, once every three weeks, on Day 1 of each course of therapy.  **Appendix: body surface area (BSA) using Dubois formula:**  BSA (m^2^) = 0.20247 x height (m)^0.725^ x weight (kg)^0.425^ |
| **Concomitant medication and concurrent therapy** | During the study treatment, investigators are allowed to provide corresponding supportive treatment according to subject’s clinical need. The specific medications can be seen in the following narration on concomitant medications. Use of the antitumor therapy unspecified in the study protocol is prohibited during treatment, however, the medical measures taken for life-threatening tumor emergencies are not included.  **Permitted Concomitant Medications**  Use of local, intra-ocular, intra-articular, intranasal and inhaled corticosteroids (very little systemic absorption) is allowed. ≤ 10 mg/day prednisone steroid dose of adrenal replacement is permitted. It is allowed to use a short-course (less than 3 weeks) corticosteroid for prevention of allergy (e.g., allergy to contrast agent dye) or pretreatment of chemotherapy or treatment of non-autoimmune disease (e.g., delayed type hypersensitivity caused by contact allergens), or treatment of adverse events caused by study drug.  If necessary, the subject might be administered with sufficient supportive treatment, including whole blood and blood product infusion, antibiotics treatment, antiallergic treatment, and antidiarrheal treatment, etc.  During the study, after discussion of the case with the medical monitor from the sponsor, the subjects can receive palliative radiotherapy for symptomatic bone metastases or isolated brain metastases upon agreement by the sponsor. The investigator needs to clearly determine that the use of palliative topical treatment does not mean progressive disease. Radiotherapy is not allowed for the target lesion.  **Prohibited Concomitant Medication**  In the screening and treatment periods of the trial, subjects are forbidden to receive the following treatments:   - Systematic antitumor chemotherapy, biotherapy, or proprietary Chinese medicines with clear anti-tumor indications; - Immunotherapy unspecified in the protocol; - Investigational product other than JS001, albumin-bound paclitaxel, pemetrexed, cisplatin and carboplatin; - Vaccination within 4 weeks prior to administration and during the administration. The vaccine includes but is not limited to: measles, epidemic parotiditis, rubella, varicella, yellow fever, rabies, Bacille Calmette-Guerin and typhoid (oral) vaccines; - Use of any systemic glucocorticoid other than treatment for the adverse events induced by immunotherapy (note: use of steroid at physiological dose is allowed after communication with the sponsor; pretreatment of chemotherapeutic drugs in accordance with the instructions or clinical diagnosis and treatment practice is allowed during treatment); - Generally, use of traditional Chinese medicine (TCM) is not recommended in the protocol. |
| **Study Visit** | The study consists of three periods: screening, treatment, and follow-up:  **Screening period:**  1. To start with the confirmation of subjects' initial inclusion eligibility and signature of informed consent form (ICF);  2. To collect tumor tissues for biomarker analysis;  3. To determine whether the patient is eligible;  4. To provide characteristics of the disease, including baseline disease status for evaluation of response.  **Treatment period:**   - Subjects will be randomized into JS001 combined with chemotherapy group (ARM1, combined therapy group) or placebo combined with chemotherapy group (ARM2, control group); - Subjects must receive the first dose of study drug within 3 days after randomization (the date of first dose is Cycle 1 Day 1, i.e., C1D1); - Evaluation of adverse events should be recorded at each clinical visit; - Regardless of delayed dose, the tumor will be evaluated using RECIST 1.1 and iRECIST criteria at baseline and once every 6 weeks in the first 12 months after C1D1. After 12 months, the tumor will be required to be evaluated once every 9 weeks, until progressive disease or termination of treatment, whichever comes later; if the investigator considers addition of tumor radiological evaluation to identify progressive disease or response based on the results of clinical evaluations, unscheduled radiological evaluation can be arranged. - This period will end when the subjects are withdrawn from the study treatment.   **For the subjects who are randomized into placebo combined with chemotherapy group (ARM2, control group):**   - The subjects in the control group are allowed to cross over to JS001 treatment after progressive disease.   Investigator judged radiological record must be available for progressive disease;   - Chemotherapy must be completed at least 3 weeks prior to administration of JS001, and chemotherapy related toxicity must be recovered to grade 1 or baseline; - In case the radiological record meets RECIST 1.1 criteria, the radiological evaluation of progressive disease can be used as the baseline radiology for subsequent evaluations following start of JS001 treatment, and the tumor will be evaluated once every 9 weeks since first dose of crossover treatment. Until progressive disease or termination of treatment, whichever comes later; if the investigator considers addition of tumor radiological evaluation to identify progressive disease or response based on the results of clinical evaluations, unscheduled radiological evaluation can be arranged.   Therapeutic regimen for the subjects who are crossed over to JS001 treatment following randomization to control group: 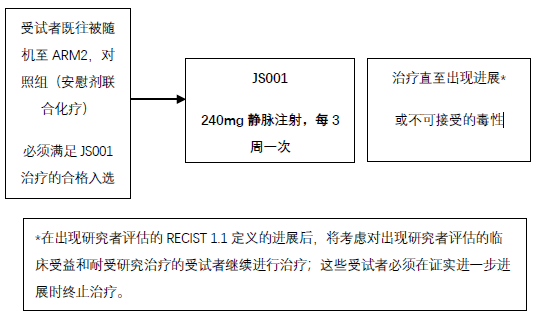 Subjects who have been randomized into ARM2 control group (placebo combined with chemotherapy) previously must meet the eligible inclusion criteria for JS001 treatment  **JS001**  **240mg iv, once every 3 weeks**  The treatment will be given until progression*  or unacceptable toxicity  * After investigator evaluated progression defined using RECIST 1.1, the treatment will be considered to be continued for the subjects with investigator evaluated clinical benefit and tolerability to study treatment; these subjects must terminate their treatment when further progression is confirmed.  **Follow-up period:**   - Start from the decision made on withdrawal of subjects from study treatment (no further study treatment given); - The patients who have terminated treatment for reasons other than tumor progression will continue to receive tumor evaluation, just like the patients are still receiving study drug, until radiological progressive disease determined using RECIST v1.1 or iRECIST criteria, death, initiation of new anti-tumor therapy, death, withdrawal of informed consent form, termination of study by the sponsor, whichever comes first; if the investigator considers addition of tumor radiological evaluation to identify progressive disease or response based on the results of clinical evaluations, unscheduled radiological evaluation can be arranged. - After completion of end-of-treatment (EOT) visit, survival follow-up will be performed for the subjects every 3 months throughout the study; - The subjects will be followed up for drug related toxicity, until these toxicities are relieved, recovered to baseline or indeed irreversible. |
| **Efficacy evaluation** | The tumor will be evaluated once every 6 weeks in the first 12 months in the study period using RECIST 1.1 and iRECIST criteria. After 12 months, the tumor will be required to be evaluated once every 9 weeks, and the patients who have received JS001 alone crossover therapy after unblinding (patients in the original control group) will undergo tumor evaluation every 9 weeks. Until progressive disease, initiation of a new anti-tumor therapy, intolerable toxicity, withdrawal of informed consent by the subject or death, the subject has received JS001 treatment for an accumulative period of 2 years or other reasons as specified in the protocol, whichever comes first. |
| **Safety evaluation** | The safety evaluation population includes the subjects who have received at least one dose of study drug, and the laboratory examinations (including hematology, blood biochemistry, blood coagulation, thyroid function and urinalysis), 12-lead ECG and vital signs as specified in the protocol will be performed. Adverse events (AEs) and serious adverse events (SAEs) will be monitored and recorded.  Adverse events will be graded in accordance with national cancer institute common terminology criteria for adverse events Version 5.0 (NCI CTCAE V5.0). |
| **Sample size** | The sample size will be calculated based on the primary efficacy endpoint, PFS evaluated by investigators using RECIST 1.1. The patients will be 2:1 randomized to receive treatment, about 450 patients need to be enrolled (about 300 in JS001 group and 150 in placebo group), expecting to conduct primary efficacy analysis when 356 PFS events are observed about 27 months after randomization of the first subject, thereby there is 85% statistical power to detect improvement of PFS in the treatment of metastatic non-small cell lung cancer by JS001 combined with standard therapy versus placebo combined with standard therapy at the one-sided significance level of 0.025 (corresponding hazard ratio HR=0.7). The interim analysis of efficacy is planned to be conducted when 214 PFS events are observed (information ratio 60%). The sample size is calculated based on the following assumptions and using EAST 6.5:   - PFS appears exponential distribution. - Median PFS is 6 months for the chemotherapy group. - α-consumption function of Pocock type (approximation using Lan-DeMets method) will be used for interim analysis and primary analysis, as to control the overall type I error rate. - 450 subjects will be enrolled within 15 months. - The drop-out rate is 5% in each treatment group in the observational period of PFS event in the first 12 months. |
| **Analysis set** | The intent-to-treat (ITT) analysis set includes all the randomized subjects and will be used as the main analysis set for efficacy analysis.  Per protocol set (PPS) includes all the ITT population with no any major deviation from the protocol that may impact efficacy analysis and valid baseline variables. Prior to unblinding in the study, the subjects included in PPS will be determined in accordance with the actual deviation from the protocol. PPS population will be used for the sensitivity analysis of primary efficacy endpoint and partial secondary endpoints.  Safety analysis set (SS) includes all subjects who have received at least dose of study drug, and will be used for safety analysis. |
| **Efficacy Analyses** | The primary efficacy endpoint is PFS evaluated by investigators using RECIST 1.1 criteria in this study. Secondary efficacy endpoints include OS, ORR, DOR, DCR, and TTR.  Stratified log rank test will be used for the comparison of efficacy based on the primary efficacy endpoint PFS. The stratification factors include smoking state (often smoking vs no smoking or infrequent smoking), pathological type (squamous cell carcinoma vs non-squamous cell carcinoma) and PD-L1 status (TC ≥ 1% vs TC < 1%, Note: The patients not evaluable for PD-L1 will be included in TC < 1% group), the significance level is one-sided 0.025. Kaplan-Meier (KM) method will be used to estimate the median PFS in each treatment group, the 95% confidential interval of median PFS will be estimated through Brookmeyer-Crowley method using log-log function conversion to reach normal approximation. The hazard ratio (HR) of PFS and its 95% confidential interval will be estimated using the stratified Cox proportional hazard model, the stratification factors are as the same as above.  The above method used for the primary efficacy endpoint PFS will be used for the secondary efficacy endpoints OS, DOR and TTR. The percentage of subjects in each treatment group will be calculated for ORR and DCR, Clopper-Pearson method will be used to calculate its 95% confidential interval, and the 95% confidential interval for the percentage difference between groups will be estimated using normal approximation. |
| **Safety analysis** | The safety analysis set will be used for safety analysis. The safety of JS001 combined with first-line standard chemotherapy in treatment-naive advanced non-small cell lung cancer (NSCLC) will be evaluated through summary of drug exposure, AEs, change in laboratory examinations and change in vital signs. |

**List of Abbreviations and Relevant Terms**

|  | **Initials/Abbreviations** | **Terms** |  |
| --- | --- | --- | --- |
|  | λ_z_ | Terminal elimination rate constant |  |
|  | ADA | Anti-drug antibody |  |
|  | ADR | Adverse Drug Reaction |  |
|  | AE | Adverse event |  |
|  | ALK | Anaplastic lymphoma kinase |  |
|  | ALP | Alkaline phosphatase |  |
|  | ALT(SGPT) | Alanine Aminotransferase |  |
|  | AST(SGOT) | Aspartate Aminotransferase |  |
|  | ANC | Absolute Neutrophil Count |  |
|  | AR | Accumulation ratios |  |
|  | AUC_0-t_ | Area under serum drug concentration-time curve |  |
|  | AUC_0-t_ /D | Dose normalized AUC_0-t_ |  |
|  | AUC_0-tau_ | Area under concentration-time curve from the first dose to the last dose |  |
|  | AUC_0-tau_/D | Dose normalized AUC_0-tau_ |  |
|  | AUC_0-∞_ | AUC to infinite time |  |
|  | BIRC | Blinded Independent Review Committee |  |
|  | BUN | Blood urea nitrogen |  |
|  | CBC | Complete blood count |  |
|  | CFDA | China Food and Drug Administration |  |
|  | CHO | Chinese Hamster Ovary |  |
|  | Cl^-^ | Chloride |  |
|  | CL | Total clearance |  |
|  | C_max_ | Serum peak concentration |  |
|  | C_max_/D | Dose normalized C_max_ |  |
|  | CRO | Contract Research Organizations |  |
|  | CRP | C-Reactive Protein |  |
|  | CT | Computer-assisted Tomography |  |
|  | CTCAE | Common Terminology Criteria for Adverse Events |  |
|  | CV% | Coefficient of variation |  |
|  | DCR | Disease control rate |  |
|  | DLT | Dose-limiting toxicity |  |
|  | DOR | Duration of response |  |
|  | DSMB | Dataset Safety Monitoring Committee |  |
|  | EBV | Epstein-Barr virus |  |
|  | EC | Ethic Committee |  |
|  | ECG | Electrocardiogram |  |
|  | ECOG | Eastern Cooperative Oncology Group |  |
|  | eCRF | electronic Case Report Form |  |
|  | EDC | Electronic Data Capture |  |
|  | EGFR | Epidermal growth factor receptor |  |
|  | FAS | Full Analysis Set |  |
|  | GCP | Good Clinical Practice |  |
|  | GLP | Good Laboratory Practice |  |
|  | HIV | Human Immunodeficiency Virus |  |
|  | HPV | Human Papilloma Virus |  |
|  | IC | Informed consent |  |
|  | ICF | Informed consent form |  |
|  | ICH | International Conference on Harmonisation |  |
|  | iCPD | iRECIST-confirmed disease progression |  |
|  | iDMC | Independent data monitoring committee |  |
|  | INR | International Normalized Ratio |  |
|  | irAE | Immune-related adverse events |  |
|  | iRECIST | Immune-related Response Evaluation Criteria in Solid Tumors |  |
|  | irRECIST | Immune-related Response Evaluation Criteria in Solid Tumors |  |
|  | IRB/IEC | Institutional Review Board/ Ethics Committee |  |
|  | iUPD | iRECIST-unconfirmed progressive disease (iUPD) |  |
| IV | | Intravenous injection | |
| K^+^ | | Potassium | |
| Kd | | Dissociation Constant | |
| Kg | | Kilogram | |
| LDH | | Lactate dehydrogenase | |
| LI | | Linear index | |
| Mab | | Monoclonal Antibody | |
| MedDRA | | Medical dictionary for Regulatory Activities | |
| Mg | | Milligrams | |
| mL | | Milliliters | |
| MRI | | Nuclear magnetic resonance imaging | |
| MRT | | Mean retention time | |
| MSI | | Microsatellite instability | |
| MTD | | Maximum tolerated dose | |
| Na^+^ | | Sodium | |
| NMPA | | National Medical Products Administration | |
| NK | | Natural killer cell | |
| NSCLC | | Non-small cell lung cancer | |
| OS | | Overall survival | |
| ORR | | Objective response rate | |
| PBMC | | Peripheral blood mononuclear cell | |
| PBSCT | | Peripheral Blood Stem Cell Transplantion | |
| PD | | Progressive Disease | |
| PD-1 | | Programmed death receptor 1 | |
| PD-L1 | | Programmed death receptor-ligand 1 | |
| PFS | | Progression-free survival | |
| PK | | Pharmacokinetics | |
| PR | | Partial Response | |
| PS | | Performance Status | |
| PLT | | Platelets | |
| RBC | | Red Blood Cells | |
| RECIST | | Response Evaluation Criteria in Solid Tumors | |
| SAE | | Serious Adverse Event | |
| SAP | | Statistical Analysis Plan | |
| SOP | | Standard Operating Procedures | |
| SUSAR | | Suspected unexpected serious adverse reaction | |
| t_1/2_ | | Half-life | |
| TB | | Tuberculosis | |
| TCR | | Tissue Cross-Reactivity | |
| T_max_ | | Time to maximum drug concentration | |
| TTR | | Time to response | |
| V_d_ | | Apparent volume of distribution | |
| V_dss_ | | Volume of distribution at steady state | |
| WBC | | White blood cells | |

# TABLE OF CONTENTS

[Sponsor’s signature page 2](#_Toc50977033)

[Investigator’s Statement 3](#_Toc50977034)

[Contract Research Organisation (CRO) Signature Page 4](#_Toc50977035)

[Organization of the clinical trial 5](#_Toc50977036)

[Protocol Synopsis 6](#_Toc50977037)

[TABLE OF CONTENTS 22](#_Toc50977038)

[Protocol body text 31](#_Toc50977039)

[1 Study Background 31](#_Toc50977040)

[1.1 Background relevant to lung cancer 31](#_Toc50977041)

[1.2 Immunotherapy Background 34](#_Toc50977042)

[1.3 Physical characteristics on Toripalimab Injection (JS001) 37](#_Toc50977043)

[1.4 Preclinical data review 38](#_Toc50977044)

[1.4.1 Pharmacodynamics 38](#_Toc50977045)

[1.4.2 Pharmacokinetics/ pharmacodynamics and immunogenicity 38](#_Toc50977046)

[1.4.3 Toxicology 38](#_Toc50977047)

[1.5 Clinical experience on Toripalimab Injection (JS001) 39](#_Toc50977048)

[1.5.1 Overall summary 39](#_Toc50977049)

[1.5.2 Clinical pharmacokinetics 43](#_Toc50977050)

[1.5.2.1 Pharmacokinetics in CT1 (HMO-JS001-I-CRP-01) 43](#_Toc50977051)

[1.5.2.2 Pooled analysis of pharmacokinetics and dose selection 45](#_Toc50977052)

[1.5.3 Clinical efficacy and safety data 46](#_Toc50977053)

[1.5.3.1 Clinical Effectiveness 46](#_Toc50977054)

[1.5.3.2 CLINICAL SAFETY 58](#_Toc50977055)

[1.6 Study rationale and benefit-risk assessment 61](#_Toc50977056)

[1.6.1 Rationale for evaluation of JS001 as monotherapy in patients with solid tumors 61](#_Toc50977057)

[1.6.2 Rationale for evaluation of JS001 combined with the 1st-line standard of care in patients with solid tumors 61](#_Toc50977058)

[1.6.3 Rationale for allowing patients to continue receiving JS001 treatment after being evaluated as progressive disease in accordance with RECIST v1.1 criteria and irRECIST criteria 62](#_Toc50977059)

[1.6.4 Rationale for collection of archived and fresh tumor samples and blood sample for biomarkers 63](#_Toc50977060)

[2 Study Objectives and Endpoints 64](#_Toc50977061)

[2.1 Study Objectives and Endpoints 65](#_Toc50977062)

[2.1.1 Primary Objectives 65](#_Toc50977063)

[2.1.2 Secondary Objectives 65](#_Toc50977064)

[2.1.3 Exploratory Objectives 65](#_Toc50977065)

[2.1.4 Primary Endpoints 65](#_Toc50977066)

[2.1.5 Secondary Endpoints 65](#_Toc50977067)

[2.1.6 Exploratory Endpoints 66](#_Toc50977068)

[3 Study Design 66](#_Toc50977069)

[3.1 Overall Design 66](#_Toc50977070)

[3.1.1 Bases for Continued Treatment allowed in the Selected Patients with Progressive Disease 68](#_Toc50977071)

[3.1.2 Rationale for Dose Selection of JS001 69](#_Toc50977072)

[3.2 Test Method 70](#_Toc50977073)

[3.2.1 Patient Screening 70](#_Toc50977074)

[3.2.2 Randomization and Treatment Assignment 70](#_Toc50977075)

[3.2.3 Stratification 71](#_Toc50977076)

[3.2.4 End of Study 71](#_Toc50977077)

[4 Study Population 71](#_Toc50977078)

[4.1 Patient Inclusion Criteria 72](#_Toc50977079)

[4.2 Patient Exclusion Criteria 73](#_Toc50977080)

[4.3 Eligibility criteria for crossover treatment to JS001 treatment period - inclusion criteria for subjects previously randomly enrolled to AMR2 (placebo plus chemotherapy group, control group) : 76](#_Toc50977081)

[4.4 Criteria for the discontinuation of investigational treatment 77](#_Toc50977082)

[4.5 Criteria for Study Withdrawal by Subjects 78](#_Toc50977083)

[4.6 Management for Subjects Who Terminate the Treatment 79](#_Toc50977084)

[4.7 Randomization, Blinding and Unblinding 79](#_Toc50977085)

[5 Study Visits and Assessments 80](#_Toc50977086)

[5.1 Informed Consent Forms 80](#_Toc50977087)

[5.2 Eligibility Criteria 80](#_Toc50977088)

[5.3 Assignment of Screening Number 80](#_Toc50977089)

[5.4 Assignment of Treatment/ Randomization Number 80](#_Toc50977090)

[5.5 Medical History 81](#_Toc50977091)

[5.6 Demography 81](#_Toc50977092)

[5.7 Physical Examination 81](#_Toc50977093)

[5.8 ECOG Score 81](#_Toc50977094)

[5.9 Electrocardiogram 81](#_Toc50977095)

[5.10 Vital Signs 82](#_Toc50977096)

[5.11 Laboratory Tests 82](#_Toc50977097)

[5.12 Screening/Baseline Period (Day -28 to Day -1) 83](#_Toc50977098)

[5.13 Visits during Treatment Period 85](#_Toc50977099)

[5.14 Treatment after Disease Progression 87](#_Toc50977100)

[5.15 Crossover-treatment Period 90](#_Toc50977101)

[5.16 End of Treatment Visit 91](#_Toc50977102)

[5.17 Safety Follow-up Visit 92](#_Toc50977103)

[5.18 Follow-up Visit 92](#_Toc50977104)

[5.19 Survival Follow-up 92](#_Toc50977105)

[6 Iinvestigational Product and Research Method 93](#_Toc50977106)

[6.1 Clinical Drug Supplies 93](#_Toc50977107)

[6.1.1 Toripalimab Injection (JS001) and Placebo 93](#_Toc50977108)

[6.1.2 Pemetrexed 93](#_Toc50977109)

[6.1.3 Nab-paclitaxel 94](#_Toc50977110)

[6.1.4 Carboplatina 94](#_Toc50977111)

[6.1.5 Cisplatin 95](#_Toc50977112)

[6.2 Management of Investigational Product 95](#_Toc50977113)

[6.2.1 Reception and Storage 95](#_Toc50977114)

[6.2.2 Disposal 95](#_Toc50977115)

[6.2.3 Preparation Method and Records 96](#_Toc50977116)

[6.2.3.1 JS001 Injection 96](#_Toc50977117)

[6.2.3.2 Pemetrexed 96](#_Toc50977118)

[6.2.3.3 Nab-paclitaxel 97](#_Toc50977119)

[6.2.3.4 Carboplatin 98](#_Toc50977120)

[6.2.3.5 Cisplatin 99](#_Toc50977121)

[6.3 Method of Administration 99](#_Toc50977122)

[6.3.1 JS001/Placebo 99](#_Toc50977123)

[6.3.2 First-line Standard Chemotherapy 100](#_Toc50977124)

[6.3.2.1 Patients with squamous cell carcinoma will receive the following chemotherapy regimen: 100](#_Toc50977125)

[6.3.2.2 Patients with non-squamous cell carcinoma will receive the following chemotherapy regimen: 100](#_Toc50977126)

[6.3.3 Continue the treatment with JS001 after disease progression 100](#_Toc50977127)

[6.4 Precautions for use 100](#_Toc50977128)

[6.4.1 Precautions for administration of JS001 101](#_Toc50977129)

[6.4.2 Precautions for use of Pemetrexed + Carboplatin or Cisplatin 102](#_Toc50977130)

[6.4.3 Precautions for nab-paclitaxel 102](#_Toc50977131)

[6.5 Concomitant medication and concurrent therapy 103](#_Toc50977132)

[6.5.1 Permitted Concomitant Medications 103](#_Toc50977133)

[6.5.2 Disallowed Concomitant Medication 103](#_Toc50977134)

[6.6 Drug compliance 104](#_Toc50977135)

[7 Test for Biomarkers 104](#_Toc50977136)

[8 Efficacy assessments 105](#_Toc50977137)

[8.1 Tumour Evaluation 105](#_Toc50977138)

[8.2 ECOG PS 106](#_Toc50977139)

[9 Safety evaluation 106](#_Toc50977140)

[9.1 Overall plan for safety issue management 106](#_Toc50977141)

[9.1.1 Monitoring 106](#_Toc50977142)

[9.1.2 Dose adjustment 107](#_Toc50977143)

[9.1.2.1 Adjustment of JS001 treatment 107](#_Toc50977144)

[9.1.2.2 Management of special adverse events for JS001 108](#_Toc50977145)

[9.1.2.3 Dose adjustment of chemotherapeutics 112](#_Toc50977146)

[9.1.2.3.1 Dose adjustment and management of specific adverse events for Pemetrexed and Cisplatin 113](#_Toc50977147)

[9.1.2.3.2 Dose adjustment and management of specific adverse events for nab-paclitaxel 114](#_Toc50977148)

[9.1.2.3.3 Dose adjustment and management of specific adverse events for carboplatin 116](#_Toc50977149)

[9.2 Safety parameters and definitions 118](#_Toc50977150)

[9.2.1 Definition of Adverse Events (AEs) 118](#_Toc50977151)

[9.2.2 Serious adverse events (SAEs) (reported to the sponsor immediately) 118](#_Toc50977152)

[9.2.3 Adverse events of special interest (AESI) (reported to the sponsor immediately) 119](#_Toc50977153)

[9.3 Causality assessment 120](#_Toc50977154)

[9.4 Assessment of severity 120](#_Toc50977155)

[9.5 Recording and reporting of adverse events/serious adverse events 121](#_Toc50977156)

[9.5.1 Recording and reporting of adverse events 122](#_Toc50977157)

[9.5.2 Reporting of Serious Adverse Events 122](#_Toc50977158)

[9.5.3 Reporting and follow-up of pregnancy 124](#_Toc50977159)

[9.5.3.1 Pregnancy of female subjects 124](#_Toc50977160)

[9.5.3.2 Pregnancy in the Female Partner of a Male Subject 124](#_Toc50977161)

[9.5.3.3 Misscarriage 124](#_Toc50977162)

[9.5.3.4 Congenital anomaly/birth defect 124](#_Toc50977163)

[10 Data management 125](#_Toc50977164)

[10.1 Data entry 125](#_Toc50977165)

[10.2 Database Lock 125](#_Toc50977166)

[10.3 Data archiving 126](#_Toc50977167)

[11 Statistical analyses 126](#_Toc50977168)

[11.1 Determination of sample size 127](#_Toc50977169)

[11.2 Data analysis sets 127](#_Toc50977170)

[11.3 Analytical method 128](#_Toc50977171)

[11.3.1 Demographic characteristics, baseline disease characteristics, and trial progress 128](#_Toc50977172)

[11.3.2 Effectiveness 128](#_Toc50977173)

[11.3.3 Safety 130](#_Toc50977174)

[11.3.4 Biomarkers Analysis 130](#_Toc50977175)

[11.4 Interim analysis 130](#_Toc50977176)

[12 Study Management 131](#_Toc50977177)

[12.1 Ethical considerations 131](#_Toc50977178)

[12.2 Informed consent 131](#_Toc50977179)

[12.3 Institutional Ethics Committee (IEC) 132](#_Toc50977180)

[12.4 Protection of patient's rights and interests 132](#_Toc50977181)

[12.5 Compensation for health damage of subjects 133](#_Toc50977182)

[12.6 Recording and storage of study data 133](#_Toc50977183)

[12.7 Original data management 134](#_Toc50977184)

[12.7.1 Types of source data 134](#_Toc50977185)

[12.7.2 Direct entry in the source data/record 134](#_Toc50977186)

[12.8 Return or Destruction of the investigational product/Treatment Supplies 135](#_Toc50977187)

[12.9 Quality control and quality assurance 135](#_Toc50977188)

[12.10 Supervision and Auditing 135](#_Toc50977189)

[12.11 Amendments tostudy protocol 136](#_Toc50977190)

[12.12 Violation to the protocol 136](#_Toc50977191)

[12.13 Study summary report 136](#_Toc50977192)

[12.14 Confidentiality, and publication of study results 136](#_Toc50977193)

[13 Quality control of trial 137](#_Toc50977194)

[14 Amendments of and deviation from trial protocol 138](#_Toc50977195)

[14.1 Amendment of Study Protocol 138](#_Toc50977196)

[14.2 Deviation from Study Protocol 138](#_Toc50977197)

[15 Trial discontinuation and closure 139](#_Toc50977198)

[15.1 Trial discontinuation required by the sponsor 139](#_Toc50977199)

[15.2 End of trial 139](#_Toc50977200)

[16 References 140](#_Toc50977201)

[17 Attachments 147](#_Toc50977202)

[Attachment 1： Study schedule 147](#_Toc50977203)

[Attachment 2： Eastern Cooperative Oncology Group (ECOG) - Performance Status Scale 155](#_Toc50977204)

[Attachment 3： Response Evaluation Criteria in Solid Tumors (RECIST1.1) 156](#_Toc50977205)

[Attachment 4： Introduction to assessment process of iRECIST for evaluating disease progression 174](#_Toc50977206)

[Attachment 5： Recommendations for alternative therapy of hypothyroidism 178](#_Toc50977207)

[Attachment 6： Precautions for allergies 180](#_Toc50977208)

**LIST OF TABLES**

[Table 1 Comparison of the structure and physicochemical property between JS001 and agents with the same target 37](#_Toc50975625)

[Table2 Overview of ongoing clinical studies of JS001 39](#_Toc50975626)

[Table 3 Summary of pharmacokinetic parameters after multiple doses of JS001 iv drip 45](#_Toc50975627)

[Table 4 Response evaluation in study CT1 (RECIST1.1) 47](#_Toc50975628)

[Table 5 Response evaluation per RECIST 1.1 in Study CT4 (HMO-JS001-II-CRP-01) (RES) 49](#_Toc50975629)

[Table 6 Response evaluation in study CT5(JS001-Ib-CRP-1.0) (RECIST1.1) 55](#_Toc50975630)

[Table 7 Summary of immune-related adverse events of Toripalimab 59](#_Toc50975631)

[Table 8 Toripalimab Injection (JS001) and placebo 93](#_Toc50975632)

[Table 9 Infusion of the First and Subsequent Doses of JS001 101](#_Toc50975633)

[Table 10 Recommended Pretreatment Medications for the Chemotherapy of Pemetrexed + Carboplatin/ Cisplatin 102](#_Toc50975634)

[Table 11 Treatment adjustment guideline for infusion reactions 109](#_Toc50975635)

[Table 12 - Adjustment plan of JS001 treatment 111](#_Toc50975636)

[Table 13 - Dose adjustment of chemotherapeutics 113](#_Toc50975637)

[Table 14 Dose adjustment of Pemetrexed and Cisplatin for hematological toxicity 113](#_Toc50975638)

[Table 15 Dose adjustment of Pemetrexed and Cisplatin for non-haematological toxicity 114](#_Toc50975639)

[Table 16 Dose Adjustment of Pemetrexed and Cisplatin Caused by Neurotoxicity 114](#_Toc50975640)

[Table 17 Dose adjustment of nab-paclitaxel for hematological and neurological adverse reactions 115](#_Toc50975641)

[Table 18 Recommendation on the starting dose in patients with hepatic impairment 115](#_Toc50975642)

[Table 19 Dose adjustment of carboplatin for hematological toxicity 116](#_Toc50975643)

[Table 20 Dose adjustment of carboplatin based on the non-hematological toxicity in the previous ccycle. 117](#_Toc50975644)

[Table 21 Severity rating scale for adverse events not specifically listed in NCI CTCAE5.0 121](#_Toc50975645)

[Table 22 Definitions of the reporting interval for adverse events/serious adverse events 121](#_Toc50975646)

[Table 23 Efficacy boundary conditions for PFS analysis by α - consumption function of Pocock type (LAN DeMets method approximation) 131](#_Toc50975647)

**LIST OF FIGURES**

[Figure1 Mean blood concentration-time curve after single dose of 1, 3 and 10 mg/kg JS001 iv drip 44](#_Toc50975732)

[Figure 2 Mean blood concentration-time curve curve after multiple doses of 1, 3 and 10 mg/kg JS001 iv drip 44](#_Toc50975733)

[Figure 3 Study Flow Chart 67](#_Toc50975734)

[Figure 4 Subsequent imaging evaluation and treatment of the subject after the first imaging evaluation on PD by the investigator 89](#_Toc50975735)

Protocol body text

# Study Background

## Background relevant to lung cancer

Lung cancer is still the main cause of cancer death globally; it is one of the most common cancers in men and women, and estimated to account for 13% of all the new cancers in 2008^[1]^. 226, 160 new cases of lung cancer and 160, 340 new cases of death from lung cancer is expected in US, in 2012^[2]^. 288, 000 new cases of lung cancer and 253, 000 new cases of death is expected in Europe, in 2008^[3]^.

Non-small cell lung cancer (NSCLC) is the main subtype of lung cancers, and accounts for about 85% of all cases ^[4, 5]^. There are two main histological types for NSCLC: adenocarcinoma and squamous cell cancer^[6]^. The histological type of adenocarcinoma accounts for more than half of all the NSCLC, whereas the squamous cell cancer accounts for about 25%^[7]^. The other NSCLC types include large cell carcinoma, neuroendocrine tumor, sarcomatoid and poorly differentiated carcinoma.

The 5-year overall survival is 2%-4% for advanced diseases and varies in different regions. The poor prognostic factors for the survival in patients with NSCLC include late stage of disease at the initial diagnosis, poor physical performance and history of weight loss of unknown origin. More than half of the NSCLC patients have distant metastasis at the diagnosis, which will aggravate the poor survival prognosis directly.

There is an obvious difference in the characteristics between adenocarcinoma and squamous cell NSCLC. Firstly, squamous cell cancer is commonly seen in central respiratory tract and usually localized in bronchial epithelial cells^[9]^, whereas the non-squamous cell cancer is commonly seen in pulmonary parenchymal area far away from central respiratory tract. The evaluation of NSCLC tumor tissue shows a cytological difference between squamous cell cancer (keratinization, intracellular bridging and central necrosis) and adenocarcinoma (glandular structure). When poorly differentiated tumor sample is present or limited tissue is available, immunohistochemical markers can be used to assist histological diagnosis. Thyroid transcription factor -1 (TTF-1) is rarely expressed in squamous cell carcinoma, however, it is actively expressed in adenocarcinoma cells. On the contrary, p63, CK5/6 and 34βE12 are actively expressed in squamous cell carcinoma, and rarely expressed in adenocarcinoma cells^[6]^.

The gene changes with significant prognostic and/or predictive effect on NSCLC include epidermal growth factor receptor (EGFR) mutation, anaplastic lymphoma kinase (ALK) gene rearrangement and GTP enzyme-Kras (KRAS) mutation. The incidence of these mutations varies in squamous cell carcinoma and adenocarcinoma. For example, it is reported that 10%-40% patients with adenocarcinoma NSCLC had EGFR kinase domain mutation, however, such mutation is rarely seen in patients with squamous cell NSCLC^[10]^. Similarly, ALK fusion oncogene (identified as the promoter of lung tumor) is observed in about 7% patients with adenocarcinoma; however, it is very rare in squamous cell carcinoma^[10, 7]^. In addition, KRAS mutation is very rare in squamous cell NSCLC, however, it can be observed in about 30% patients with adenocarcinoma NSCLC^[6]^.

**Treatments for advanced non-small cell lung cancer without EGFR mutation and ALK rearrangement**

In the main markets worldwide, the conventional first-line treatment for patients with driver gene negative advanced NSCLC is platinum based dual-therapy, including Carboplatin and Paclitaxel (or nab-paclitaxel [Abraxane®]), Carboplatin or Cisplatin combined with Gemcitabine (for squamous cell cancer only), Carboplatin or Cisplatin combined with Pemetrexed (for non-squamous cell cancer only). The maintenance therapy is also recommended for some histologic subtypes of NSCLC, i.e. continuation of original therapy or change of drugs; e.g., pemetrexed maintenance therapy has shown that OS and PFS could be improved, especially for non squamous histological subtypes ^[11,12]^.

Pemetrexed combined with Cisplatin may significantly prolong the survival of patients with non-squamous NSCLC as compared to gemcitabine combined with chemotherapy, the median survival time of patients in the adenocarcinoma subgroup receiving the combined therapy and monotherapy respectively was 12.6 months vs. 10.9 months, and that of patients with large cell subgroup receiving the combined therapy and monotherapy was 10.4 months vs. 6.7 months respectively, and the tolerance was better ^[13]^.

A large Phase III clinical study PARAMOUNT confirms that Pemetrexed maintenance therapy for progression-free patients after 4 cycles of Pemetrexed combined with Cisplatin until progressive disease or intolerable toxicity prolonged PFS (4.1 months vs. 2.8 months) and OS (13.9 months vs. 11.0 months) ^[14-15]^of patients with PS score of 0~1, compared to placebo.

Nab-paclitaxel is a new type of chemotherapy drug in which advanced nanotechnology is used to wrap Paclitaxel with albumin, as the castor oil isn't required to be used as the cosolvent, the incidence of allergic reaction is significantly reduced, and it is unnecessary to pretreat piror to medication, nab-Paclitaxel can be infused within a short duration (30 minutes) compared to conventional Paclitaxel drugs, significantly improving the safety and enhancing the convenience of usage. Meanwhile, tumor tissue had a higher uptake ^[16-17]^ of albumin through high expression of acid secretory protein (SPARC) rich in cysteine, and the drug concentration reaching tumor cells would be higher, consequently, better clinical anti-tumor effect and relatively low side effects would be achieved, and a number of clinical studies have confirmed the efficacy of nab-Paclitaxel in solid tumors ^[18-20]^. CA-031 Study ^[21]^ showed ORR of nab-paclitaxel was significantly higher than that of paclitaxel in patients with Squamous-cell carcinoma (SCC) NSCLC (41% vs 24%; response rate ratio [RRR] 1.680; P < 0.001). The ORR with nab-paclitaxel and Paclitaxel was 26% and 27% in adenocarcinoma patients (RRR 0.966; P = 0.814), 33% vs 15% (RRR 2.167; P = 0.323) in patients with large cell carcinoma (LC), and 24% vs 15% (RRR 1.593; P = 0.372) in patients with other pathological types. In patients with squamous cell carcinoma, the median overall survival with nab-paclitaxel and Paclitaxel was 10.7 and 9.5 months (HR 0.890; P = 0.310) respectively, and the median overall survival of general population was 12.4 and 10.6 months (HR 1.208; P = 0.721) respectively. The nab-paclitaxel significantly reduced the incidence of Grade 3/4 neuropathy and joint pain (P < 0.05), while Paclitaxel showed lower incidence of thrombocytopenia and anemia. In conclusion, the first-line use of nab-paclitaxel in NSCLC patients showed good risk-benefit profile regardless of histology. In 2012, the US Food and Drug Administration (FDA) approved the nab-paclitaxel for use in combination with carboplatin for the first-line treatment of patients with advanced or metastatic NSCLC who could not be treated radically by surgery or radiotherapy.

The estimated 5-year overall survival (OS) of patients with advanced NSCLC was still low (11% and 17% ^[22,23]^ respectively) despite of advancements in the diagnosis, imaging, staging, and treatment of NSCLC. Median OS was 10-12 months in patients with advanced NSCLC ^[24]^. The patients with non-target mutation (i.e., epidermal growth factor receptor [EGFR] or anaplastic lymphoma kinase [ALK] mutations) had the response rate of about 20% to 30% and progression free survival (PFS) of 4-5 months ^[25,26,27]^. The duration of response (DOR) was also limited, and the drug resistance might be the main limiting factor. The 1-year survival rate of patients with good physical status was 30% - 40%. Therefore, the first-line treatment of driver gene negative advanced NSCLC was an unmet major clinical need.

Pembrolizumab, based on the data of KEYNOTE-021 G in 2017, had been approved by the Food and Drug Administration (FDA) for first-line treatment of patients with metastatic non squamous NSCLC without EGFR or ALK gene mutation. It was fully approved in August 2018 based on the confirmatory outcomes of KEYNOTE-189.

KEYNOTE-021G Cohort is an extended cohort of Phase I / II studies that compared the efficacy and safety of Pembrolizumab + Carboplatin and Pemetrexed versus Carboplatin and Pemetrexed alone as first-line treatment in patients with advanced NSCLC with non squamous histological subtypes. Compared to patients receiving the chemotherapy alone (29%), the patients receiving Pembrolizumab combined with chemotherapy as the first-line therapy (55%) had higher ORR ^[28]^.

Pembrolizumab combined with Pemetrexed and platinum-based chemotherapy versus placebo combined with Pemetrexed and platinum-based chemotherapy for the treatment of previously untreated locally advanced or metastatic non-squamous NSCLC was compared in KEYNOTE-189 ^[29]^ Study. A total of 616 patients were enrolled, the 12-month overall survival rate of patients receiving Pembrolizumab combined with chemotherapy was 69.2% (95% confidence interval [CI], 64.1- 73.8), and 49.4% (95% CI, 42.1-56.2) in the placebo combined with chemotherapy group (death risk factor 0.49; 95% CI 0.38-0.64; P < 0.001). The progression free survival of patients was 8.8 months (95% CI, 7.6-9.2) in the pembrolizumab combined with chemotherapy group and 4.9 months (95% CI, 4.7-5.5) in the placebo combined with chemotherapy group (hazard ratio of progressive disease or death, 0.52; 95% CI, 0.43-0.64; P < 0.001). The overall tolerability of combined therapy was good. The incidence of adverse events ≥ Grade 3 in Pembrolizumab combined with chemotherapy group was 67.2% and 65.8% in the placebo combined with chemotherapy group. The efficacy was irrelevant to the expression of PD-L1, and even patients with negative PD-L1 could benefit from the combined therapy.

KEYNOTE-407 ^[30]^ Study compared Pembrolizumab combined with Carboplatin +Paclitaxel or nab-Paclitaxel versus Placebo +Carboplatin +Paclitaxel or nab-Paclitaxel in the treatment of previously untreated locally advanced or metastatic lung squamous cell carcinoma, a total of 204 patients were enrolled as of the first interim analysis, of whom 101 received chemotherapy + Pembrolizumab, and 103 received chemotherapy; the median follow-up duration was 7.7 months (range: 0.4-13.9 months). 78% of the patients were male, 48% of the patients were under 65 years old, and 28% of the patients had ECOG PS score of 0. 35% patients had TPS of PD-L1 < 1%. 32% patients used nab-paclitaxel. ORR with Pembrolizumab + chemotherapy and chemotherapy alone respectively was 58.4% and 35.0%, P = 0.0004. 65.8% patients receiving Pembrolizumab + chemotherapy and 45.6% patients receiving chemotherapy alone had a median duration of response ≥ 6 months. The incidence of Grade 3-4 AEs in Pembrolizumab + chemotherapy group and chemotherapy alone group was 64.4% and 74.5% respectively. The OS HR in the nab-Paclitaxel subgroup was 0.59 (95% CI 0.36-0.98).

These three studies suggest that the immunotherapy combined with chemotherapy is an effective first-line therapeutic regimen for patients with advanced NSCLC.

## Immunotherapy Background

From the exploration of antibody therapy and cytokine therapy in the 1970s and 1980s, to the cellular immunotherapy firstly approved by FDA for prostate cancer in 2010, as well as Bristol-Myers Squibb’s CTLA-4 monoclonal antibody Yervoy against immunological checkpoint approved in 2011 (CLTA-4 target by Bristol-Myers Squibb) and the launch of Opdivo (jointly developed by Onokazu Pharma and Bristol-Myers Squibb) and Keytruda (MSD) for PD-1 in 2014, the immunotherapy for tumors has gone through more than a hundred years of development. Currently, the immunotherapy has become one of the regimens in clinical treatment for tumors and mainly includes cytokine therapy, therapeutic vaccine, adoptive cell therapy (ACT) and immune checkpoint block or immune costimulatory pathway activator, including CTLA-4/PD-1 antibody. Tumor cells can inhibit the activity of T cells and escape from immune killing through expression of the ligand of immune checkpoint. The proliferation, survival and killing activity of T cells can be enhanced through blockage of such immune checkpoint, thereby achieving the immunotherapeutic effect. In recent years, a good effect has been shown for the checkpoint-specific targeted monoclonal antibody in monotherapy or combination with chemotherapy, making its position increase continuously in treatment of a variety of tumors.

The CTLA-4 targeted drug Ipilimumab (trade name: Yervoy) developed by Bristol-Myers Squibb is the first immune checkpoint antagonistic antibody in the world. In 2014, the PD-1 inhibitor Nivolumab (trade name: Opdivo) jointly developed by Bristol-Myers Squibb and Onokazu Pharma is firstly launched in Japan for treatment of advanced melanoma. The Pembrolizumab (trade name: Keytruda) developed by MSD is the first PD-1 inhibitor in US, for treatment of advanced melanoma with BRAF V600E mutation after failure of Ipilimumab therapy, or dual resistance to Ipilimumab and BRAF inhibitor. Compared with the traditional chemotherapeutic agent and targeted therapeutic agent, these immune checkpoint antibodies have the following characteristics^[31]^:

Broad-spectrum: the development and growth of tumor are dependent on immunosuppression and immunologic escape, PD-1 and CTLA-4 antibodies target on human immune cells and kill cancer cells indirectly through activation of in vivo immune system, its broad-spectrum antitumor effect is shown in clinical trials.

Specificity and low toxicity: the traditional chemotherapy kills rapid-growing cells in a non-specific manner, although the small molecule and antibody for targeted therapy reduce this non-specific phenomenon based on that, there is still great toxicity as corresponding targets are also more or less expressed in normal cells, for example, EGFR antigen. Immunotherapy activates the killer T cells against tumor cells specifically through antigen memory response, therefore, it has enhanced specificity and reduced toxicity.

Metastasis: it has been demonstrated in lung, liver and small intestinal tumors that when T cell is activated, it will be greatly proliferated and metastatic to the targeted cancer tissues, effectively increase the number of activated T cells in target organs, thereby killing tumor cells^[32]^.

Adjustability^[33]^: tumor cells have high mutation rate, its surface antigen will change based on that. Correspondingly, after mature T cells are activated, the body can respond to more epitopes (including hidden epitopes) in succession through epitope spreading with the continuation of immune response, adjust the immune response to new tumor antigen and achieve immune reaction to the original and metastatic lesions, making systemic tumor subside. At the same time, the immune reaction against multiple antigens makes it difficult for the tumor to produce escape variant.

Durability: for specific tumor antigen, the immune cells have memory ability, can inhibit the development of tumor cells for a long term and achieve curative effect on tumors. The phase III clinical study of Ipilimumab showed 25% patients with advanced melanoma could survive for more than two years, 43% patients receiving Nivolumab for treatment of melanoma were still alive two years later^[34]^.

Due to the above characteristics of immune checkpoint-blocking antibody, its therapeutic effect can remold human immune system, enhance the capability of immune system to recognize tumor cells, and achieve the objective of inhibition and killing of cancer fundamentally. This will not only reduce the pain from treatment, improving the quality of life substantially, but also regain the suppressed immunological memory of immune cells in the counterbalance with tumor cells, forming permanent anticancer effect and eradicating recurrence and metastasis, and prolonged patient’s survival while the quality of life is guaranteed.

PD-1 is one important inhibitor receptor on the surface of T cells, expressed on activated T cells, B cells, macrophages, dendritic cells and monocytes. The ligand of PD-1 is B7 homologous protein PD-L1 (also known as B7-H1) and PD-L2 (also known as B7-DC). The PD-1 intracellular domain contains one ITIM motif and one immuno receptor tyrosine-based switch motif (ITSM). The ITSM motif mediates the recruitment of SHP family phosphatase and inhibition of T cell activation signal. PD-L1 ligand is highly expressed on the surface of various tumor cells, and PD-1/PD-L1 binding plays an important role in the down-regulation of T cell activation and the maintenance of peripheral immune tolerance, thus tumor cells can inhibit T cell activation and escape from the killing by immune cells through expressing PD-L1 and thereby interacting with PD-1^[35]^.

Currently, the clinical trials conducted for PD-1 targeted agent at home and overseas include advanced melanoma, non-small cell lung cancer, renal cell carcinoma, gastric cancer, ovarian cancer, triple-negative breast cancer, colorectal cancer, pancreatic adenocarcinoma, hepatocellular carcinoma, prostate cancer, transitional cell carcinoma, head and neck squamous cell carcinoma, head and neck cancer, adult acute myeloid leukemia, chronic granulocytic leukemia, multiple myeloma, myelodysplastic syndrome, cervical carcinoma and glioblastoma. Results of the clinical trials that have ended and the interim results of partial trials show PD-1 antibody is significantly superior to the previous therapeutie approaches in terms of better efficacy and higher safety.

## Physical characteristics on Toripalimab Injection (JS001)

Toripalimab Injection, hereinafter referred to as JS001, targets on human PD-1 and is a neutralizing blocking antibody. It binds to PD-1 with high affinity, selectively blocks the binding of PD-1 to its ligand PD-L1 and PD-L2, thereby activating T lymphocytes, improving proliferation of lymphocytes and secretion of cytokines, in particular IFN-γ.

The preclinical pharmacodynamic study showed JS001 significantly stimulated the proliferation of CD4+ and CD8+ T cells and enhanced the activation of human effector/memory T cells in the graft-versus-host diease (GVHD) animal model induced by adoptive transfer of human PBMC. At the same time, the study in 624 MEL animal model transplanted with human melanoma showed JS001 combined with cytotoxic lymphocyte (CLT) could eliminate immunosuppressive effect, enhance the killing effect of CLT on tumor cells and achieve the expected good therapeutic effect.

Compared with Nivolumab and Pembrolizumab, the CDR sequence and structure at the binding site of JS001 with antigen mainly differ in 6 CDR sequences, which determines the different physiochemical and biological characteristics among them. The results of affinity test based on SPR or ELISA show JS001 has higher affinity than Pembrolizumab and Nivolumab. See Table 1.

Table 1 Comparison of the structure and physicochemical property between JS001 and agents with the same target

| **Sample** | **Antibody subtype** | | **Binding affinity**  **(SPR)** | **Binding EC50 (Elisa)** | **Source** |
| --- | --- | --- | --- | --- | --- |
|  | **Heavy chain** | **Light chain** |  |  |  |
| JS001 | IgG4 | Kappa | 0.92nM | 64pM | Humanized |
| Pembrolizumab | IgG4 | Kappa | Not reported | 70pM | Humanized |
| Nivolumab | IgG4 | Kappa | 2.64nM | Not reported | Fully humanized |

JS001 preparation is developed as injection in a specification of 240mg/6ml/vial. CFDA clinical trial approval was obtained on December 27, 2015, the approval number was: 2015L05752.

## Preclinical data review

### Pharmacodynamics

The in vitro pharmacodynamic study showed antigen memory response to JS001 could significantly stimulate massive proliferation of T cells and enhance release of IFN-γ. The in vivo experiment in NSG mice showed that JS001 could effectively increase the proliferation of CD4+ and CD8+ T cells, whilst enhancing better activation of human effector/memory T cells, which was obviously potent than the same-target drug Nivolumab that had been approved overseas. JS001 could eliminate the immunosuppression of tumor on T cells and the killing effect of cytotoxic T lymphocyte (CLT) on tumor cells. In addition, JS001 would not induce antibody-dependent cell mediated cytotoxicity and cytokine storm.

The receptor occupancy test in Cynomolgus monkeys showed a dose-effect relationship between JS001 and PD-1 binding on cell surface, the effective dose started from serum antibody concentration 0.3 μg/mL and became saturated at 3 μg/mL, when the optimal biological effect was exerted. These preclinical data provided evidence for dose inference in further clinical studies for JS001.

### Pharmacokinetics/ pharmacodynamics and immunogenicity

After iv drip administration of single dose of test article JS001 at different doses (1, 10 and 75 mg/kg) in Cynomolgus monkeys, the serum exposure level was linearly increased with dose increase in the dose range of 1-10 mg/kg. The Cmax was 27.70 ± 12.29 µg/mL, 216.11 ± 34.52 µg/mL and 1891.72 ± 270.16 µg/mL in each dose group, respectively. The in vivo t1/2 was 134-194 hours, which was similar with the pharmacokinetic (PK) data reported for Nivolumab and Pembrolizumab, and no obvious immunogenicity was found.

### Toxicology

As the binding of JS001 to mouse-derived PD-1 is non-specific, cynomolgus monkey is selected as the main animal tested for toxicological study in accordance with the *Gguideline on Non-Clinical Safety Evaluation for Therapeutic Biologics*. The systematic evaluation of preclinical safety and toxicology was commissioned to National (Shanghai) Center for New Drug Safety Evaluation, and conducted strictly in accordance with Good Laboratory Practice. General pharmacology, acute toxicity, chronic toxicity studies, hemolysis and local stimulation as well as other toxicity studies have been conducted.

It is found in the study on evaluation of safety pharmacology of recombinant humanized anti-PD-1 antibody that 10, 30 and 100 mg/kg JS001 have no obvious effect on the cardiovascular system and respiratory frequency in cynomolgus monkey after intravenous administration. At the same time, JS001 has no effect on the motor coordination and behavioral activity in rats.

The acute toxicity study showed no obvious acute toxicity after single intravenous dose of JS001 to cynomolgus monkeys, and the No Observed Adverse Effect Level (NOAEL) was 406 mg/kg.

In the subacute toxicity study after repeated intravenous administration of JS001 once per week for consecutive 4 weeks, no obvious toxicity was seen for JS001. No drug-related abnormal reaction is seen in all the animals. No toxicologically significant regular change was seen in the weight, temperature, electrocardiogram (ECG) parameters, coagulation function, ophthalmologic examination, routine urinalysis, serum cytokines, each parameter in serum chemistry in each dose group at each testing time point. JS001 basically shows a linear pharmacokinetic profile in cynomolgus monkeys, and there is accumulation after repeated doses.

During the chronic toxicity study in cynomolgus monkeys after intravenous administration of 10, 30 and 100 mg/kg JS001 for consecutive 26 weeks, neither abnormal changes related with JS001 nor obvious toxic reaction was seen in weight, food consumption, clinical observations, temperature, ECG, ophthalmologic examination, complete blood cell count and coagulation function, serum chemistry, immune function, urinary and fecal analysis, macroscopic pathology, organ weight and histopathology. Thus, the NOAEL was 100 mg/kg for JS001.

Please refer to JS001 investigator’s brochure for the preclinical study data on JS001.

## Clinical experience on Toripalimab Injection (JS001)

### Overall summary

Currently, the clinical study of Toripalimab Injection (JS001) is conducted only in China, including several ongoing and planned studies, as to evaluate the efficacy and safety of monotherapy or combined therapy with other drugs. By August 31, 2018, a total of 18 phase I-III programs were ongoing, and more than 900 patients have been enrolled in JS001 clinical studies in total. Please see Table2 for the complete list of the ongoing studies.

Table2 Overview of ongoing clinical studies of JS001

| **No.** | **Study code** | **Trial name** | **Progress** |
| --- | --- | --- | --- |
| CT1 | HMO-JS001-I-CRP-01 | A phase I, open, mono-center, dose-escalation study to investigate the tolerability and pharmacokinetics of single dose and multiple doses of recombinant humanized anti-PD-1 monoclonal antibody injection in patients with advanced tumors | Enrollment closed |
| CT2 | JS001-I-CRP-1.4 | A phase Ia clinical study on the safety, tolerability, pharmacokinetics and pharmacodynamics of single dose combined with multiple doses of Toripalimab Injection in patients with advanced solid tumors | Enrollment closed |
| CT3 | JS001-I-CRP-1.3 | A phase I clinical study on the safety, tolerability, pharmacokinetics and pharmacodynamics of single dose and multiple doses of recombinant humanized anti-PD-1 monoclonal antibody injection in patients with advanced malignant tumors | Enrollment closed |
| CT4 | HMO-JS001-II-CRP-01 | One open, multi-center, single-arm, phase II clinical study to investigate the efficacy and safety of recombinant humanized anti-PD-1 monoclonal antibody injection in patients with locally advanced or metastatic melanoma after failure of standard of care | Enrollment closed |
| CT5 | JS001-Ib-CRP-1.0 | A multi-center, open, phase Ib/II clinical study to evaluate JS001 in the treatment of advanced gastric adenocarcinoma, esophageal squamous cell carcinoma, nasopharyngeal carcinoma and squamous cell carcinoma of head and neck | Enrollment ongoing |
| CT6 | JS001-I | A phase I clinical study on the safety, tolerability, pharmacokinetics and pharmacodynamics of multiple doses of recombinant humanized anti-PD-1 monoclonal antibody injection in patients with recurrent refractory malignant lymphoma | Enrollment closed |
| CT7 | HMO-JS001-I-PK -01 | One phase I study to investigate the similarity in pharmacokinetics and safety of single-dose and parallel comparison of recombinant humanized anti-PD-1 monoclonal antibody injection in patients with advanced NSCLC before and after process change | Enrollment ongoing |
| CT8 | HMO-JS001-II-MM-02 | One randomized, controlled, multi-center, phase II clinical study to investigate recombinant humanized anti-PD-1 monoclonal antibody injection versus high-dose interferon in adjuvant therapy of completely resected mucosal melanoma | Enrollment ongoing |
| CT9 | HMO-JS001-I-CRP-03 | An open-label, monocenter, dose-escalation, phase I clinical study to investigate the tolerability and pharmacokinetics of single and multiple doses of recombinant humanized anti-PD-1 monoclonal antibody injection in patients with triple negative breast cancer. | Enrollment closed |
| CT10 | HMO-JS001-I-CRP-1.4 | A prospective, phase I clinical study on radiotherapy combined with recombinant humanized anti-PD-1 monoclonal antibody injection (JS001) in treatment of advanced triple negative breast cancer | Enrollment ongoing |
| CT11 | HMO-JS001-I-CRP-1.5 | A phase I clinical trial on recombinant humanized anti-PD-1 monoclonal antibody injection (JS001) in combination with Gemcitabine + Cisplatin (GP) as the first-line therapy for advanced triple negative breast cancer | Not initiated but closed |
| CT12 | HMO-JS001-II-CRP-02 | One open, multi-center, single-arm, phase II clinical study to investigate the efficacy and safety of recombinant humanized anti-PD-1 monoclonal antibody injection in patients with locally advanced or metastatic bladder urothelial carcinoma after failure of standard of care | Enrollment ongoing |
| CT13 | HMO-JS001-Ib-CRP-01 | One open, mono-center, dose-escalation, phase Ib clinical study to investigate the tolerability and pharmacokinetics of recombinant humanized anti-PD-1 monoclonal antibody injection combined with Axitinib in patients with advanced renal carcinoma and melanoma after failure of standard of care | Enrollment ongoing |
| CT14 | HMO-JS001-Ib-NEC-02 | A phase Ib clinical study to investigate the safety and effectiveness of recombinant humanized anti-PD-1 monoclonal antibody injection in patients with advanced neuroendocrine tumor after failure of standard of care | Enrollment ongoing |
| CT15 | JS001-NPC-III | One randomized, double-blind, international, multi-center, phase III study to evaluate the efficacy and safety of JS001/placebo combined with GP regimen (Gemcitabine and cisplatin) in treatment of advanced nasopharyngeal carcinoma | Initiating |
| CT16 | JS001-016-II-HCC | One randomized, double-blind, multi-center, phase II study to evaluate the efficacy and safety of recombinant humanized anti-PD-1 monoclonal antibody (JS001) as the postoperative adjuvant therapy for patients receiving radical resection of high-risk recurrent liver cancer | Initiating |
| CT17 | JS001-017-III-MM | One randomized, controlled, multi-center, phase III clinical study to investigate recombinant humanized anti-PD-1 monoclonal antibody injection (JS001) versus dacarbazine as the 1st-line therapy for unresectable or metastatic melanoma | Enrollment ongoing |
| CT18 | JS001-PII-LC-001 | A multi-center, single-arm phase II clinical study to evaluate JS001 combined with Pemetrexed and carboplatin in treatment of advanced or recurrent non-small cell lung cancer with EGFR sensitive mutation and negative T790M after failure of EGFR-TKI therapy | Enrollment ongoing |

Current clinical data on the safety and effectiveness of Toripalimab Injection (JS001) mainly comes from the following 5 clinical trials in patients with solid tumors. Other studies included:

Study HMO-JS001-I-CRP-01(CT1) was a phase I, open, mono-center, dose-escalation study to investigate the tolerability and pharmacokinetics of single dose and multiple doses of JS001 in patients with advanced tumors. The dose-escalation group included 1 mg/kg Q2W, 3 mg/kg Q2W and 10 mg/kg Q2W in the study. A total of 36 patients with solid tumors after failure of previous treatment were enrolled, mainly including those with advanced melanoma, renal carcinoma and urothelial carcinoma. No dose-limiting toxicity was observed in each dose group, the maximum exposure dose was 10 mg/kg, and valid case of clinical response was observed in each dose group.

Study JS001-I-CRP-1.4(CT2) was a phase Ia clinical study on the safety, tolerability, pharmacokinetics and pharmacodynamics of single dose combined with multiple doses of JS001 in patients with advanced solid tumors. The dose-escalation group included 0.3 mg/kg, 1 mg/kg Q2W, 3 mg/kg Q2W, 10 mg/kg Q2W and 240 mg Q2W in the study. A total of 25 patients with solid tumors after failure of previous treatment were enrolled, mainly including those with gastric cancer, esophageal cancer and nasopharyngeal carcinoma. No dose-limiting toxicity was observed in each dose group, the maximum exposure dose was 10 mg/kg, and valid case of clinical response was observed in multiple dose groups.

Study HMO-JS001-II-CRP(CT4) was one open, multi-center, single-arm, phase II clinical study to investigate the effectiveness and safety of JS001 3mg/kg Q2W in treatment of patients with locally advanced or metastatic melanoma after failure of standard of care. The primary endpoint was the objective response rate (ORR) evaluated by independent radiological data review committee in accordance with the response evaluation criteria in solid tumors (RECIST1.1). By January 4, 2018, a total of 128 patients with locally advanced or metastatic melanoma after failure of previous treatment had been enrolled, a total of 128 patients had been included in the safety analysis set. JS001 has good safety and tolerability, the adverse reactions are mainly grade 1-2, and no new, unexpected safety signal is found compared with similar products that have been approved overseas.

Study JS001-Ib-CRP-1.0 (CT5) was a multi-cohort, phase Ib/II clinical study (basket trial) intended to preliminarily evaluate the anti-tumor activity and tolerability of JS001 in the treatment of advanced gastric adenocarcinoma, esophageal squamous cell carcinoma, nasopharyngeal carcinoma and squamous cell carcinoma of head and neck, and provide a basis for the subsequent phase III clinical study. The primary endpoint was the objective response rate (ORR) evaluated by investigators based on RECIST 1.1. It is shown in the interim analysis by November 30, 2017 that a total of 161 patients have been imcluded in the efficacy evaluation analysis set and the objective response rate is 22.4% according to the preliminary data. A total of 201 patients have been included in the safety evaluation analysis set, the overall safety and tolerability is good, the adverse reactions are mainly grade 1-2, and no new, unexpected safety signal is found.

JS001-I (CT6) was one phase I, dose-escalation clinical study on the safety, tolerability, pharmacokinetics and pharmacodynamics of multiple doses of JS001 in patients with recurrent refractory malignant lymphoma. The dose-escalation group included 1 mg/kg Q2W, 3 mg/kg Q2W and 10 mg/kg Q2W in the study. The primary objective was to determine the safety and tolerability of JS001 monotherapy in treated subjects with advanced or recurrent malignant lymphoma. By February 23, 2018, the analysis showed a total of 13 patients with lymphoma after failure of previous therapy were enrolled, no dose-limiting toxicity was observed in each dose group, the maximum exposure dose was 10 mg/kg, and valid case of clinical response was observed in multiple dose groups.

### Clinical pharmacokinetics

Currently, there have been no clinical pharmacological studies conducted specifically for Toripalimab Injection (JS001). The pharmacokinetic data on JS001 are mainly sourced from the following studies on JS001 monotherapy: CT1, CT2 and CT3. Currently, no data have been acquired on the pharmacokinetics of JS001 combined with other anticancer drugs. The population pharmacokinetic model is planned to be established and validated at appropriate time points in the future, as to evaluate the effect of clinical factors on drug exposure, for example, age, sex, weight, hepatic and renal function, etc.

#### Pharmacokinetics in CT1 (HMO-JS001-I-CRP-01)

The concentration of JS001 in human serum samples from CT1 (from the enrolled patients with the dose level of 1-10 mg / kg) was quantified using methodologically validated electrochemiluminescence (MSD), the lowest limit of quantitation (LLOQ) was 2.560 ng/mL.

Watson LIMSTM v.7.3.0.01 (Thermo Scientific Inc.) software was used to manage all the samples determined in the analysis batch and data incurred. The standard curve fitting and calculation of blood concentration were completed using the computing function of the software. The pharmacokinetic parameters were analyzed using the non-compartment model in WinNonLin v 6.4 (Certara Corp.) software. Linear Mixed Effects Wizard was used to evaluate the dose linearity of drug exposure level in each dose group. Dixon Q-test statistical method was used for identification and elimination of outlier values.

The mean blood concentration – time curve in Cycle 1 after administration of 1, 3 and 10 mg/kg JS001 iv drip was shown in Figure 1 below (single-dose phase), the mean blood concentration – time curve in the full course of therapy was shown in Figure 2 (multi-dose phase).

Figure1 Mean blood concentration-time curve after single dose of 1, 3 and 10 mg/kg JS001 iv drip


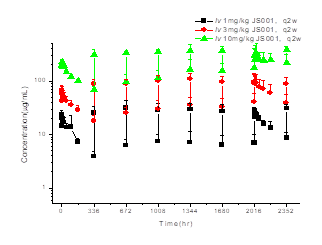


Figure 2 Mean blood concentration-time curve curve after multiple doses of 1, 3 and 10 mg/kg JS001 iv drip

After single dose of JS001 iv drip at different doses (1, 3 and 10 mg/kg), the preliminary pharmacokinetic data in Cycle 1 showed the serum exposure level was increased with dose increase and basically appeared a linear pharmacokinetic profile, the mean Cmax increased proportionally with dose increase, the peak concentration Cmax was 22.13±3.94, 65.59±14.44 and 182.70±30.68 μg/mL, respectively. Similarly, AUC(0-t) was 3584.64±588.88, 12924.87±3666.69 and 40118.28±14113.95 hr*μg/mL, respectively, which was approximately proportionally with dose; CL was similar in each dose group, i.e., 0.27, 0.22 and 0.24 mL/hr/kg, respectively, which also further validated the linear pharmacokinetic profile. The actual measurements of CL and Vd were consistent with that in human typical IgG4 antibody in the range ≥1 mg/kg for JS001.

After multiple doses of JS001 iv drip at different doses (1, 3 and 10 mg/kg, once every two weeks), the blood concentration basically reached steady state after consecutive three doses, the average trough concentration was 6.23±2.05, 25.15±7.42 and 97.39±36.25 μg/mL, respectively. After the 7th dose, the peak concentration Cmax was 47.05±16.97, 105.19±32.67 and 323.77±41.96 μg/mL, respectively, AUC(0-inf) was 8815.79±2382.45, 44022.34±6105.77 and 126194.03±72330.76 hr*μg/mL, respectively; the accumulation factor was 1.57±0.19, 2.25±0.77 and 2.00±0.54 in each dose group, respectively. A certain accumulation of JS001 was shown in humans after multiple doses. This conclusion needed further data support due to little data available.

#### Pooled analysis of pharmacokinetics and dose selection

Data on the samples in each dose group of JS001 (dose level 0.3-10mg/kg once every two weeks and 240 mg fixed dose) from three phase I dose-escalation clinical studies (CT1, CT2, CT3) were summarized for pooled analysis of pharmacokinetics. See Table 3 for the detailed parameters. The study results showed a basically linear pharmacokinetics after administration of 1, 3 and 10 mg/kg JS001 iv drip for clinical subjects; and a certain non-linear pharmacokinetic profile when the dose was increased to 10 mg/kg. The study results showed an obvious “long-acting” feature of antibody drugs for JS001, the blood concentration basically reached a steady state in clinical subjects after the administration of JS001 iv drip for consecutive 3-4 doses, the mean half-life was 222.98±61.92 hours (9.29±2.58 days), Cmax was 77.17±47.3 and AUC(0-t) was 11635.89±7204.64 in 3 mg/kg (n=37) dose group after multiple doses.

Table 3 Summary of pharmacokinetic parameters after multiple doses of JS001 iv drip

| **PK variables** | **Unit** | **JS001** | | | | | | | | | |
| --- | --- | --- | --- | --- | --- | --- | --- | --- | --- | --- | --- |
|  |  | **0.3 mg/kg** | | **1 mg/kg** | | **3 mg/kg** | | **10 mg/kg** |  | **240 mg/subject** | |
| Kel | 1/hr | 0.004±0.000 | n=2 | 0.003  ±0.001 | n=14 | 0.002  ±0.001 | n=27 | 0.002  ±0.001 | n=10 | 0.001 | n=1 |
| t_1/2_ | hr | 188.322  ±4.934 | n=2 | 242.52  ±42.69 | n=14 | 302.91  ±88.48 | n=27 | 430.3  ±193.82 | n=10 | 525.106 | n=1 |
| T_max_ | hr | 0-2 | n=2 | 2  (0.5，12) | n=14 | 2(0.5，24) | n=27 | 2(0，24) | n=10 | 0 | n=1 |
| C_max_ | μg/mL | 6.577  ±2.193 | n=2 | 30.42  ±11.03 | n=14 | 93.31  ±30.55 | n=27 | 305.97  ±97.01 | n=10 | 152.628 | n=1 |
| AUC_(0-t)_ | hr*μg/mL | 1099.367  ±448.421 | n=2 | 4538.23  ±1541.22 | n=14 | 18180.89  ±6101.07 | n=27 | 62833.57  ±24694.62 | n=10 | 34212.001 | n=1 |
| AUC_(0-inf)_ | hr*μg/mL | 1583.594  ±717.532 | n=2 | 7895.14  ±2051.06 | n=14 | 34529.42  ±12671.64 | n=27 | 157946.58  ±81099.1 | n=10 | 95199.868 | n=1 |
| AUC_(t-inf)%_ | % | 29.785  ±3.498 | n=2 | 37.87  ±6.88 | n=14 | 46.09±9.07 | n=27 | 54.72  ±13.45 | n=10 | 64.063 | n=1 |
| Vd | mL/kg | 57.017  ±24.486 | n=2 | 66.58  ±18.73 | n=14 | 54.1  ±27.26 | n=27 | 69.64  ±54.56 | n=10 | 1909.836 | n=1 |
| Cl | mL/hr/kg | 0.211  ±0.096 | n=2 | 0.19  ±0.05 | n=14 | 0.13  ±0.06 | n=27 | 0.12  ±0.08 | n=10 | 2.521 | n=1 |
| MRTinf | hr | 264.836  ±11.921 | n=2 | 341.12  ±58.95 | n=14 | 435.8  ±125.05 | n=27 | 609.1  ±259.74 | n=10 | 755.415 | n=1 |

The steady-state lowest blood concentration was ranged from 20-40μg/ml at the dose of 3 mg/kg once every two weeks. The in vitro study showed the PD-1 receptor on the surface of T cells could be saturated when the concentration of JS001 was >20nM or 3μg/ml, the concentration of JS001 was usually maintained at 25μg/ml in peripheral blood, in consideration of the restricted entry of antibody macromolecules in tumor microenvironment, as to ensure complete occupancy of PD-1 in the lymphocytes in tumor microenvironment. Each phase I study showed complete occupancy of PD-1 receptor could be maintained throughout the treatment in different dose groups (0.3, 1, 3 and 10 mg/kg, once every two weeks). In accordance with the above pharmacokinetic results and data on receptor occupancy, the dose of 3 mg/kg once every two weeks was supported to be selected as the dose for CT4 pivotal study. 360mg fixed dose once every three weeks was explored in two early studies (CT5 and CT7), the preliminary pharmacokinetic results from 9 patients showed that, compared with 3 mg/kg once every two weeks (from the 11 patients in CT1), the steady-state peak concentration was increased to 153% (166.53 ug/mL: 95% CI 121.0 to 212.0 vs 108.83 ug/mL: 95% CI 87.5 to 130.0); the steady-state peak concentration was increased to 133% (47.92 ug/mL: 95% CI 31.8 to 64.0 vs 36.09 ug/mL: 95% CI 27.7 to 44.5), AUC 0-Day85 was increased to 138% (164993 ug/mL*hr vs 119814 ug/mL*hr). The PK model predicted a similar trough concentration (~32 ug/mL) and exposure level for 240mg once every three weeks and 3 mg/kg once every two weeks. In comprehensive analysis of the above results of pharmacokinetics, receptor occupancy of JS001 and the results from the phase II pivotal study for melanoma, JS001 240 mg fixed dose once every three weeks was supported as the recommended dose for phase III clinical study.

### Clinical efficacy and safety data

#### Clinical Effectiveness

**Study CT1 (HMO-JS001-I-CRP-01)**

Study CT1, a phase I study initiated in Beijing Cancer Hospital, is designed to investigate the tolerability and safety of single and multiple doses of Toripalimab Injection (JS001) in patients with malignant melanoma and advanced tumors of urinary system, and determine the dose-limiting toxicity (DLT) .

The study included two parts, phase I dose-escalation period and extension period. A total of 10 subjects were recruited to receive 1 mg/kg, 3mg/kg and 10mg/kg doses sequentially in dose-escalation period, there were 3, 4 and 3 subjects in the above dose groups, respectively. The number of subjects was expanded to 12, 11 and 3 in the three dose groups (1, 3 and 10 mg/kg Q2W) in dose-extension period, respectively. The enrollment started on March 24, 2016 and ended on December 19, 2016. In the statistical analysis of clinical data by July 31, 2017, 36 patients were included in the safety and efficacy evaluable population, there were 15, 15 and 6 patients in 1 mg/kg, 3 mg/kg and 10 mg/kg dose groups, respectively. There were 22 patients with melanoma (including acral melanoma in 11 patients, mucosal melanoma in 4 patients, sunlight-injured melanoma in 4 patients and non-sunlight-injured melanoma in 3 patients), 5 patients with renal cancer and 8 patients with urothelial carcinoma. The median age of patients was 51 years (range: 28-70 years), the male patients accounted for 58%, and there were 47%, 50% and 3% patients with ECOG PS score of 0, 1 and 2, respectively.

The results evaluated by investigators in accordance with the response evaluation criteria in solid tumors 1.1 (RECIST1.1) showed partial response (PR) was observed in each dose group, in a total of 6 patients, including melanoma in 3 patients and renal cancer in 3 patients, one patient (2.78%) with malignant acral melanoma was observed to reach complete response (CR) in 3 mg/kg dose group, the objective response rate (ORR) was 19.44%, 10 patients reached stable disease (SD) and the disease control rate (DCR) reached 50%. In addition, one patient (bladder cancer) in 1 mg/kg group reached partial response, which has not been confirmed yet. See Table 4 for details. The result based on irRECIST is similar to that based on RECIST 1.1.

Table 4 Response evaluation in study CT1 (RECIST1.1)

|  |  | **1 mg/kg group** | **3 mg/kg group** | **10 mg/kg group** | **Total** |
| --- | --- | --- | --- | --- | --- |
| RECIST | CR | 0(0.00) | 1(6.67) | 0(0.00) | 1(2.78%) |
|  | PR | 1(6.67) | 3(20.00) | 2(33.33) | 6(16.67%) |
|  | uPR | 1(6.67) | 0(0.00) | 0(0.00) | 1(2.78%) |
|  | SD | 7(46.67) | 3(20.00) | 0(0.00) | 10(27.78%) |
|  | PD | 5(33.33) | 8(53.33) | 4(66.67) | 17(47.22%) |
|  | NE | 1(6.67) | 0(0.00) | 0(0.00) | 1(2.78%) |
|  | Total | 15 | 15 | 6 | 36 |
|  |  |  |  |  |  |
|  | ORR(%) | 6.67 | 26.67 | 33.33 | 19.44 |
|  | 95% CI | (0.17,31.95) | (7.79,55.10) | (4.33,77.72) | (8.19,36.02) |
|  |  |  |  |  |  |
|  | ORR(%) not confirmed | 13.33 | 26.67 | 33.33 | 22.22 |
|  | 95% CI | (1.66,40.46) | (7.79,55.10) | (4.33,77.72) | (10.12,39.15) |
|  |  |  |  |  |  |
|  | DCR(%) | 60.00 | 46.67 | 33.33 | 50.00 |
|  | 95% CI | (32.29,83.66) | (21.27,73.41) | (4.33,77.72) | (32.92,67.08) |

By July 31, 2017, all the patients had been followed up for more than 7 months after drug administration, no DLT or infusion reactions occurred in the 36 subjects enrolled; by the lock of database, a total of 6 events of death had been reported, including progressive tumor in 4 subjects, and serious adverse event of decreased appetite (due to progressive disease) in 1 subject whose prognosis was death. One subject died accidentally that was judged by investigators as positively unrelated with the investigational drug; two deaths were judged to be related with the investigational drug, i.e., decreased appetite (due to progression of disease) and progressive tumor, respectively.

The AEs related with the investigational drug were mostly grade 1-2, the most frequently reported (>10%) adverse events related with the investigational drug included rash, fever, anemia, sinus tachycardia, pruritus, hypochloraemia, weakness, decreased appetite, hypoproteinemia, cough, hyponatremia, sinus bradycardia, sinus arrhythmia, nausea, vomiting and hypocalcemia. The serious adverse events related with the investigational drug included decreased appetite, arrhythmic attack each in 2 patients, and fever, pulmonary infection and progressive tumor each in 1 patient.

The grade 3 or above adverse events related with the investigational drug included anemia, hypokalemia, abnormal heart rate, elevated blood uric acid, decreased blood pressure and chronic renal disease; the grade 3 or above laboratory abnormalities related with the investigational drug included elevated lipase, bilirubin conjugated increased, blood glucose increased, urine protein present, elevated alanine aminotransferase, blood creatine phosphokinase increased, blood creatinine increased.

There was immune-related hypothyroidism in 2 patients and immune-related hyperthyroidism in 1 patient, which were grade 1-2 and did not lead to discontinuation or interruption of doses.

It was found through preliminary analysis that there was no dose-response relationship between the investigational drug-related toxicity and drug dose.

The tumor tissue specimen was acquired from 28 patients, the expression level of PD-L1 on tumor cells (TC) and immune cells (IC), as well as CD8 positive cells (i.e., TIL, tumor-infiltration lymphocyte) were evaluated using the PD-L1 immunohistochemical double staining method independently developed by the Laboraotry of Shanghai Junshi Biosciences Co., Ltd. The results showed the clinical response was associated with tumor-infiltration lymphocyte (TIL) and PD-L1 expression. In the 15 patients from TIL high-expression subgroup, ORR reached 47% and DCR was 73%; whereas in the 16 patients from PD-L1 positive (defined as positive PD-L1 >1%) subgroup, ORR reached 44% and DCR was 69%.

**Study CT4 (HMO-JS001-II-CRP-01)**

CT4 is an open, single-arm, multi-center, phase II clinical study to evaluate the safety and effectiveness of Toripalimab Injection (JS001) (3 mg/kg, once every two weeks) monotherapy in patients with locally advanced or metastatic melanoma after failure of standard of care. The primary objective of this study is the objective response rate (ORR) evaluated by the Independent radiological data review committee in accordance with RECIST 1.1.

The enrollment started on December 28, 2016 and ended on September 15, 2017. A total of 128 subjects were enrolled. By the cutoff date of data on March 15, 2018, there had been 127, 128, 121 and 121 patients in the full analysis set (FAS), safety set (SS), response evaluable set (RES) and per protocol set (PPS), respectively. Median follow-up for the 127 patients included in FAS was 5.79 months.

The median age was 52.49 years (range: 21-76 years) in the subjects in FAS; 57 subjects were male and accounted for 44.88%, 70 subjects were female and accounted for 55.12%; 73 (57.48%) and 54 (42.52%) subjects had ECOG performance status score of 0 and 1, respectively; all the patients had received systematic treatment previously, including 87 (68.5%) patients who had received ≥2 lines of systematic treatment and 56 (44.1%) patients who had received ≥3 lines of systematic treatment. The clinical pathological type included cutaneous type (acral, 50 patients, 39.37%), cutaneous type (non-acral, 29 patients, 22.83%), mucosal type (22 patients, 17.32%), unknown primary foci type (26 patients, 20.47%).

The results of evaluation by the independent 3rd-party evaluation committee in accordance with the response evaluation criteria in solid tumors 1.1 (RECIST1.1) suggested that, based on the overall response-evaluable population, there was 1 (0.83%) patient with complete response (CR), 21 (16.54%) patients with partial response (PR), 51 (40.16%) patients with stable disease (SD), and 48 (37.80%) patients with progressive disease (PD); the ORR was 17.32% (95%CI: 11.19, 25.04%) and the disease control rate (DCR) was 57.48% (95%CI: 48.40, 66.20%). See Table 5 below for details.

Table 5 Response evaluation per RECIST 1.1 in Study CT4 (HMO-JS001-II-CRP-01) (RES)

|  | **Evaluation by independent third party (N=127)** | |
| --- | --- | --- |
|  | **Evaluation by RECIST 1.1** | **Evaluation by irRECIST** |
| BOR |  |  |
| CR | 1(0.79) | 1(0.79) |
| PR | 21(16.54) | 22(17.32) |
| SD | 51(40.16) | 53(41.73) |
| PD | 48(37.80) | 43(33.86) |
| NE | 6(4.72) | 8(6.30) |
|  |  |  |
| ORR |  |  |
| n | 22 | 23 |
| % | 17.32 | 18.11 |
| 95% CI^*^ | (11.19，25.04) | (11.84，25.92) |
|  |  |  |
| DCR |  |  |
| n | 73 | 76 |
| % | 57.48 | 59.84 |
| 95% CI^*^ | (48.40，66.20) | (50.78，68.44) |

Note: objective response rate (ORR) =(CR+ PR)/total number of cases ×100%

Disease control rate (DCR) = (CR + PR + SD)/ total number of cases ×100%

The subgroup analysis was conducted in the FAS population for objective response rate (ORR) according to the evaluation results provided by the independent radiological data review committee per RECIST 1.1 criteria. The following parameters were included: sex (male VS female), age (<65 years VS ≥65 years), ECOG PS score (0 VS 1), number of lines of previous systematic treatment (1 line VS 2 lines VS 3 lines VS ≥4 lines), previous treatment history (chemotherapy, interferon, IL2 and Ipilimumab), different subtypes of melanoma (cutaneous type (non-acral) VS cutaneous type (acral) VS mucosal type VS unknown primary foci type), LDH level (LDH<140U/L VS 140≤LDH≤280U/L VS LDH >280 U/L), brain metastasis (yes VS no), number of baseline foci (<3 VS ≥3), BRAF test result (mutant type VS wild type), PD-L1 test result (negative VS positive) and ADA test result (positive VS negative).

Clinical benefits were noted in different pathological subtypes, and ORR and DCR for different subtypes are shown as follows, 14.00% (7/50, 95% CI: 5.82, 26.74%) and 52.00% (26/50, 95% CI: 37.42, 66.34%) for cutaneous type(acral); 0% and 40.91% (9/22, 95%CI: 20.71, 63.65%) for mucosal type; 31.03% (9/29, 95%CI: 15.28, 50.83%) and 65.52% (19/29, 95%CI: 45.67, 82.06%) for cutaneous type (non-acral); 23.08% (6/26, 95%CI; 8.97, 43.65%) and 73.08% (19/26, 95%CI: 52.21, 88.43%）for unknown primary foci type.

ORR value of patients with postive PD-L1 is higher than that of patient with PD-L1, ORR and DCR of 26 PD-L1 positive patients are 38.46% (10/26, 95% CI: 20.23, 59.43%) and 80.77% (21/26, 95% CI: 60.65, 93.45%); ORR and DCR of 84 PD-L1 negative patients were 11.90% (10 / 84, 95% CI: 5.86, 20.81%) and 48.81% (41/84, 95% CI: 37.74, 59.96%) respectively.

ORR value of patients with BRAF mutation is higher than that of patients with wild-type BRAF, where ORR and DCR of 34 patients with BRAF mutations are 32.35% (11 / 34, 95% CI: 17.39, 50.53%) and 70.59% (24/34, 95% CI: 52.52, 84.90%); while ORR and DCR of 86 patients with wild-type BRAF are 9.30% (8 / 86, 95% CI: 4.10, 17.51%) and 52.33% (45/86, 95% CI: 41.27, 63.21%) respectively.

The ORR was numerically higher in the patients who had received Ipilimumab previously than that in those who had not received Ipilimumab previously, and in the 9 patients who had received Ipilimumab previously, the ORR and DCR were 22.22% (2/9, 95%CI: 2.81, 60.01%) and 77.78% (7/9, 95%CI: 39.99, 97.19%); and 16.95% (20/118, 95%CI: 10.67, 24.96%) and 55.93%(66/118, 95%CI: 46.50, 65.06%) in the 118 patients who had not received Ipilimumab previously.

Analysis of the remaining subgroup showed that in the patients with advanced melanoma after failure of standard of care, the ORR was not significantly correlated with age (<65 VS ≥65 years), gender (male VS female), ECOG score (0 VS 1), ADA test results (positive VS negative), LDH level (LDH<140 U/L VS 140≤LDH≤280U/L VS LDH >280 U/L), previous chemotherapy (yes VS no), previous use of interferon (yes VS no) and previous use of IL2 (yes VS no).

Based on the evaluation results by the independent radiographic data review board per RECIST 1.1 criteria, the analysis of duration of response (DOR) showed CR or PR in 22/127 patients; by the cutoff date of data, the response was ongoing in 21 patients, 21 patients were censored, and the median duration of response had not been reached yet; DOR was ranged from 1.87 months to 10 months, which were all censored data. It was supposed that that the end data of response of 21 subjects with persistent response was the cutoff date of data in current analysis , namely March 15, 2018, and the deletion or loss was not considered, actually average estimatable was no less than 6.02 months, and the median DOR was not less than 4.99 months. irRECIST evaluation results were similar.

Time to response (TTR) was analyzed and showed CR or PR in 22/127 patients, the median time to response was 3.45 months (95%CI: 1.74, 3.57), and the time to response was ranged from 1.61 month to 7.34 months.

The median progression-free survival (PFS) was 3.61 months (95%CI: 2.72, 5.48 months). The longest progression-free survival was 13.61 months, and the 6-month progression-free survival rate was 34.89%.

The OS data were far from mature (107 out of 127 patients were censored) and had not yet reached the median overall survival, with the longest survival of 14.52 months, the 6-month survival rate of 88.87%, and the 12-month survival rate of 78.97%.

The cutoff date for safety data was March 15, 2018, and only 3 out of 128 patients undergoing the safety analysis were reported to have SAE with death outcomes, 2 (1.56%) of whom were found to have disease progressive disease, 1 (0.78%) were found to suffer from infectious pneumonia, and the investigators assessed to be possibly unrelated.

2/128(1.56%) patients had infusion reaction (mainly rash), leading to dose interruption for 1-2 hours. The dose was continued after the rash was resolved, the infusion was prolonged to 120 minutes. The previous duration of infusion was 60 minutes and anti-allergic drug was given for pretreatment.

The most frequently reported adverse events (≥ 10%) related with the investigational drug in the treatment of advanced melanoma include elevated alanine aminotransferase (27.34%), elevated thyroid stimulating hormone (25.78%) and urine protein present (25.78%), blood creatine phosphokinase increased (22.66%), rash (22.66%), elevated blood glucose (20.31%), decreased white blood cell count (19.53%), and positive urinary leukocytes (19.53%), elevated aspartate aminotransferase (19.53%), increased amylase (18.75%), hematuria (17.19%), skin depigmentation (17.19%), anemia (16.41%), decreased neutrophil count (15.63%), increased conjugated bilirubin (14.84%), pruritus (14.84%), loss of appetite (14.06%), hypothyroidism (14.06%), elevated serum bilirubin (13.28%), decreased free thyroxine (13.28%), serum thyroid stimulating hormone (12.50%), fatigue (12.50%), elevated free thyroxine (10.94%), fever (10.94%), cough (10.94%), and elevated free triiodothyronine (10.16%). Most AEs were grade 1-2 and could be recovered through dose interruption or symptomatic treatment.

Grade 3 or above investigational drug related adverse event occurred in 30 (23.44%) patients. The incidence of all the Grade 3 or above investigational drug related adverse events was lower than 3%, except grade ≥3 increase of amylase and lipase (3.91% each).

14 (10.94%) patients had SAE related with investigational drug, the incidence of various types of SAE by PT was lower than 2%, i.e., pancreatitis, upper gastrointestinal hemorrhage, decreased platelet count and hepatic disease (each in 2 patients (1.56%)), as well as pancreatitis, elevated transaminase, infectious pneumonia, interstitial lung disease, progressive tumors, hypophysitis, venous thrombosis of extremities and uveitis (each in 1 patient (0.78%)).

19 (14.84%) patients terminated the study for investigational drug related adverse events, and the AEs occurring in ≥ 2 patients included elevated alanine aminotransferase, elevated aspartate aminotransferase, and elevated blood creatine phosphokinase separately in 3 (2.34%) patients, as well as increased amylase, decreased platelet count, increased lipase and upper gastrointestinal hemorrhage, acute pancreatitis, and liver disease separately in 2 (1.56%) patients. 9 (7.03%) patients had dose interruption for investigational drug related AEs, and the AEs occurring in ≥2 patients included elevated alanine aminotransferase, hypertriglyceridemia and skin rash separately in 2 (1.56%) patients.

Immune-related adverse reactions are as follows:

2 (1.6%) patients developed immune related interstitial pulmonary disease, including 1 case each of Grade 1 and Grade 3. The median time of onset was 5.2 months (range, 2.7-7.7 months), 1 patient (0.8%) required permanent discontinuation of study drug treatment and received corticosteroids with an initial prednisone dose of 50 mg for 3 days, and the event was relieved with sequelae. Another patient developed interstitial lung disease of Grade 1 in about 20 days after the last dose of the study drug, and the event was ongoing by the cutoff date of data.

18 (14.1%) patients developed immune-related hypothyroidism, including Grade 1 AE in 6 (4.7%) patient, Grade 2 AE in 12 (9.4%) patients and Grade ≥3 AE in 0 patient. The median time to onset was 2.8 months (range: 1.0-9.1 months), and the median duration was 19.3 weeks (range: 3.6-49.3 weeks). No patient required permanent discontinuation or interruption of the investigational therapy. 12 (66.7%) patients received thyroid hormone replacement therapy, and no patient used corticosteroid. 6 (33.3%) patients had a relieved condition, and the median time to response was 8.0 weeks (range: 3.6-28.0 weeks).

7 (5.5%) patients developed immune-related hyperthyroidism, including Grade 1 AE in 5 (3.9%) patients, Grade 2 AE in 2 (1.6%) patients, and Grade ≥3 AE in 0 patient. The median time to onset was 1.0 months (range: 0.9-7.4 months), and the median duration was 4.7 weeks (range: 3.6-15.9 weeks). No patient required permanent discontinuation or interruption of the investigational therapy for hyperthyroidism hyperthyroidism. 2 (28.6%) patients received Methimazole symptomatic therapy, but no patient received corticosteroid therapy. 4 (57.1 %) patients had a relieved condition, and the median time to response was 4.7 weeks (range: 3.9-12.1 weeks).

3 (2.3%) patients developed immune-related pancreatitis (all Grade 2). The median time to onset was 0.5 months (range: 0.4-1.9 months), and the median duration was 5.1 weeks (range: 1.0-7.4 weeks). 3 (2.3%) patients needed to permanently discontinued the study medication. No patient was treated with corticosteroids. 3 (100 %) patients had a relieved condition, and the median time to response was 5.1 weeks (range: 1.0-7.4 weeks).

5 (3.9%) patients had immune-related liver dysfunction, including 3 (2.3%) patients with events of Grade 1 and 2 (1.6%) patients with events of Grade 4. The median time to onset was 1.9 months (range: 0.5-2.9 months), and the median duration was 10.4 weeks (range: 2.1-22.3 weeks). 2 (1.6%) patients needed to be subject to permanent discontinuation of therapy with JS001 No patient was treated with corticosteroids. 4 (80.0 %) patients had a relieved condition, and the median time to response was 8.7 weeks (range: 2.1-13.0 weeks).

1 (0.78%) patient had immune-related adrenocortical insufficiency, which was grade 2. The patient received corticosteroids and prednisone with 7.5mg of initial dose by the time of onset. up to the cut-off date of data, the event was still reported to be continuous, the use of corticosteroids was still reported to be continuous. The study medication was not changed, and the dose was still maintained.

ADA was detected in all the patients, and positive in 23 (18.0%) patients. No significant differences in the incidence of AE of Grade 3 or above, discontinuation of investigational drug due to AE, interruption of investigational drug due to AE and SAE as well as the incidence of adverse events leading to death were not noted in subjects with positive and negative ADA.

Generally, JS001 has good compliance, safety and tolerability in advanced or metastatic melanoma patients with a failure of previous standard treatment, the adverse reactions are mainly grade 1-2, the immune-related adverse reactions have a low incidence and severity, and no new, unexpected safety signal is found as compared with similar products that have been approved overseas.

**Study CT5 (JS001-I-CRP-1.3)**

CT5 is a phase Ib/II study initiated in multiple centers, including Sun Yat-sen University Cancer Center, to evaluate the preliminary efficacy, safety and tolerability of Toripalimab Injection (JS001) 3mg/kg Q2W monotherapy (cohorts 1-4) and 360 mg Q3W combined with the 1st-line standard therapy (cohorts 5-8) for advanced gastric adenocarcinoma, esophageal squamous cell carcinoma, nasopharyngeal carcinoma and squamous cell carcinoma of head and neck.

There were a total of 8 cohorts in this study. By October 10, 2017, 279 patients had been enrolled in cohorts 1-4, i.e., 58, 59, 128 and 34 patients separately with gastric adenocarcinoma, esophageal squamous cell carcinoma, nasopharyngeal carcinoma and squamous cell carcinoma of head and neck after the failure of at least 1st-line therapy received JS001 3mg/kg alone once every two weeks.

The primary efficacy endpoint of this study was ORR evaluated in accordance with RECIST1.1 criteria, the secondary efficacy endpoints included duration of response (DCR), disease control rate (DCR), progression-free survival (PFS), overall survival (OS), progression-free survival at 6 months (6M PFS%), survival rate at one year (1YR OS%), frequency of positive and negative anti-drug antibody (ADA) detection from the baseline, and the exploratory efficacy endpoint included the analysis of the correlation between the expression of PD-L1 in tumor tissues and the antitumor activity of JS001. Safety variables included adverse events (AE), changes in laboratory test, changes in vital signs, electrocardiogram (ECG) , infusion reactions, serious allergic reactions and immune-related AE (irAE).

The interim statistical analysis was based on the data from cohorts 1-4 (subjects receiving JS001 monotherapy). The objects for safety analysis were the subjects who had been screened successfully and received the investigational treatment by October 10, 2017. By the cutoff date of data on April 10, 2018, 279 patients had been included in the safety set (defined as the subjects who had received at least one dose of JS001) for cohorts 1-4; there were 58 patients with gastric adenocarcinoma, 59 patients with esophageal squamous cell carcinoma, 128 patients with nasopharyngeal carcinoma, 34 patients with squamous cell carcinoma of head and neck. The objects for efficacy analysis were the subjects who had been screened successfully and received the investigational treatment by August 31, 2017. By the cutoff date of data on November 30, 2017, 161 patients had been included in the efficacy evaluation set (the subjects who had received at least one dose of JS001 and had at least one post-baseline tumor evaluation in addition to baseline evaluation) for cohorts 1-4; there were 43 patients with gastric adenocarcinoma, 48 patients with esophageal squamous cell carcinoma, 100 patients with nasopharyngeal carcinoma, and 23 patients with squamous cell carcinoma of head and neck.

The analysis on the baseline characteristics of patients showed that the median age was 56 years (range: 24-75 years), the male patients accounted for 80.6%, as well as 22.9% and 77.1% patients had ECOG score 0 and 1, respectively. The median age (minimum, maximum) was 59.5 (28, 75) years, 60.0 (42, 73) years, 58.0 (42, 74) years and 46.0 (24, 71) years in gastric adenocarcinoma, esophageal squamous cell carcinoma and squamous cell carcinoma of head and neck, and nasopharyngeal carcinoma cohorts, respectively. The subjects with ECOG score 0 and 1 accounted for 34.5% vs 65.5%, 10.2% vs 89.8%, 27.37% vs 72.7% and 11.8% vs 88.2% separately in gastric adenocarcinoma, esophageal squamous cell carcinoma, nasopharyngeal carcinoma and squamous cell carcinoma of head and neck cohorts. All included patients previously received the systematic treatment.

By the cutoff date of data, the proportion of subjects who had completed ≥4 cycles of therapy was 34.5% in gastric adenocarcinoma cohort, 49.2% in esophageal squamous cell carcinoma cohort, 36.7% in nasopharyngeal carcinoma cohort and 32.4% in squamous cell carcinoma of head and neck cohort; the proportion of subjects who had completed ≥6 cycles of therapy was 29.3% in gastric adenocarcinoma cohort, 32.2% in esophageal squamous cell carcinoma cohort, 17.2% in nasopharyngeal carcinoma cohort and 17.6% in squamous cell carcinoma of head and neck cohort. The subjects with the longest duration of treatment had received the 1st dose in the 17th cycle.

By August 31, 2017, 161 subjects had reached the time for efficacy evaluation. Overall, the results evaluated by investigators in accordance with the response evaluation criteria in solid tumors 1.1 (RECIST1.1) showed complete response in a total of one patient (esophageal cancer) and partial response in 35 patients, and objective response rate (ORR) of 22.4% based on the overall efficacy evaluable population. A total of 44 (27.3%) patients had stable disease and the disease control rate (DCR) was 49.7% in the overall efficacy evaluable population. There were 8 patients with partial response in the patients with gastric adenocarcinoma, the objective response rate was 20.0%; there was 1 patient with complete response and 9 patients with partial response in the patients with esophageal squamous cell carcinoma, the objective response rate was 20.8%; there were 14 patients with partial response in the patients with nasopharyngeal carcinoma, the objective response rate was 28.0%; there were 4 patients with partial response in the patients with squamous cell carcinoma of head and neck, the objective response rate was 17.4%. See Table 6 for details.

Table 6 Response evaluation in study CT5(JS001-Ib-CRP-1.0) (RECIST1.1)

| **N (%)** | **Gastric adenocarcinoma group** | **Oesophageal squamous cell carcinoma group** | **Nasopharyngeal carcinoma group** | **Squamous carcinoma of head and neck group** | **Total** |
| --- | --- | --- | --- | --- | --- |
| Number of patients | 40 | 48 | 50 | 23 | 161 |
| CR | 0(0.0) | 1(21) | 0(0.0) | 0(0.0) | 0(0.0) |
| PR* | 8 (20.0) | 9 (18.8) | 14 (28.0) | 4 (17.4) | 35 (21.7) |
| SD | 11 (27.5) | 15 (31.3) | 14 (28.0) | 4 (17.4) | 44 (27.3) |
| PD | 19 (47.5) | 20 (41.7) | 20 (40.0) | 14 (60.9) | 73 (45.3) |
| NE | 2 ( 5.0) | 3 ( 6.3) | 2 ( 4.0) | 1 ( 4.3) | 8 ( 5.0) |
|  |  |  |  |  |  |
| ORR(%) | 8 (20.0) | 10 (20.8) | 14 (28.0) | 4 (17.4) | 36 (22.4) |
| 95% CI | 9.1, 35.6 | 10.5, 35.0 | 16.2, 42.5 | 5.0, 38.8 | 16.2, 29.6 |
| DCR(%) | 19 (47.5) | 25 (52.1) | 28 (56.0) | 8 (34.8) | 80 (49.7) |
| 95% CI | 31.5, 63.9 | 37.2, 66.7 | 41.3, 70.0 | 16.4, 57.3 | 41.7, 57.7 |

Note: DCR=disease control rate; ORR=objective response rate; PFS=progression-free survival; PR=partial response; SD=stable disease

*Including unconfirmed partial response.

The response profile evaluated in accordance with immune-related response evaluation criteria in solid tumors (irRECIST) was consistent with that by RECIST1.1, and the objective response rate (ORR) was 22.4% as well.

Safety Analysis: By the cutoff date on April 10, 2018, 93.9% of the patients in cohorts thorough from Cohort 1 and Cohort 4 N =279) had a one AE t least, 40.5% patients suffered from AEs graded at Level 3 or above, 26.5% patients had SAEs leading to delath, 9.7% had fatal SAE, 11.5% patients were subject to the interruption of investigational drug due to AEs, and 20.4% had the discontinuation of investigational drug due to AEs.

90.3% patients reported AEs related with the investigational drug, the incidence of grade ≥3 related with the investigational drug was 32.3%, 18.3% patients had SAEs related with the investigational drug,and 6.8% patienns experiened SAEs causing death. 10.0% patients interrupted the dose of the investigational drug for AEs related with the investigational drug, and 14.3% patients terminated the investigational drug for AEs related with the investigational drug. The most frequently reported adverse events which are related to the study drug (≥ 10%) included the anemia, elevated aspartate aminotransferase, fever, loss of appetite, hypothyroidism, hyponatremia, elevated alanine aminotransferase, constipation, and cough.

In grade ≥3 AEs related with the investigational drug, there was anemia in 21 (7.5%) cases, hyponatremia in 17 (6.1%) cases, pulmonary infection in 6 (2.2%) cases, elevated γ-glutamyl transferase and fatigue separately in 5 (1.8%) cases, as well as elevation of both serum amylase and alkaline phosphatase, decreased platelet count, hypercalcemia and hyperuricemia separately in 4 (1.4%) cases. The most frequently reported serious adverse events (≥ 2%) were 8 cases (2.9%) with pulmonary infection and 6 death cases(2.2%), including 7 (2.5%) cases with pulmonary infection and 6 (2.2%) cases with death relevant to the study drug. The development of underlying diseases in most patients were possibly relevant factors.

5 (1.8%) patients had infusion reactions, all of which were non serious adverse events with maximum level graded at 1-2 and had been improved. Where, one case led to the suspension of investigational drug and improved after symptomatic treatment, and all patients did not lead to the discontinuation of investigational drug.

Occurrence of immune-related adverse reactions

3 (1.1%) patients had immune related interstitial lung disease, and the event with the highest intensity in each patient was Grade 2. The median time to onset was 1.5 months (range: 0.6-1.8 months), and the median duration was 6.0 weeks (range: 4.4 - 29.3+ weeks). Two (0.7%) patients needed to terminate the investigational treatment permanently, and one (0.4%) patient needed to interrupt the investigational treatment. Three patients received corticosteroids, the median equivalent start dose of prednisone was 50 mg (range: 33.0-100.0 mg) and the median duration of administration was 25.0 days (range: 22.0-42.0 days). One patient was responded with the duration of response for 6.0 weeks.

38 (13.6%) patients developed immune-related hypothyroidism, including Grade 1 AEs in 25 (9.0%) patients, Grade 2 AEs in 13 (4.7%) patients, and Grade ≥3 AEs in 0 patient. The median time to onset was 2.7 months (range: 0.5-7.6 months), and the median duration was 26.0 weeks (range: 1.1 - 57.1+ weeks). One (0.4 %) patient needed to terminate the investigational treatment permanently, and no patient needed to interrupt the investigational treatment. 23 (60.5%) patient received thyroid hormone replacement therapy, and no patient received corticosteroids. 4 (10.5 %) patients had a relieved condition, and the median time to response was 3.4 weeks (range: 2.1-10.1 weeks).

Of the patients receiving JS001, 6 (2.2%) patients developed immune-related hyperthyroidism, including Grade 1 AEs in 4 (1.4%) patients , Grade 2 AEs in 2 (0.7%) patients, and Grade ≥3 AEs in 0 patient. The median time to onset was 1.9 months (range: 0.9-2.2 months), and the median duration was 7.1 weeks (range: 4.3-32.4+ weeks). No patients terminated the investigational therapy due to hyperthyroidism, while 1 patient (0.4 %) interrupted the investigational therapy due to hyperthyroidism. 1 (16.7%) patient received Methimazole symptomatic therapy, and no patient received corticosteroid therapy. 5 (83.3%) patients had a relieved condition, and the median time to response was 6.3 weeks (range: 4.3-32.4 weeks).

8 (2.9%) patients were found to have hyperglycemia or diabetes, and the highest intensity of the event was Grade 1 in 7 (2.5%) patients, and Grade 2 in 1 (0.4%) patient, and Grade 3 or above in 0. The median time to onset was 4.3 months (range: 0.7-11.8 months), and the median duration was 3.6 weeks (range: 1.9-41.0+ weeks). No patient required the permanent discontinuation or interruption of the investigational therapy. No patient received symptomatic hypoglycemic therapy and corticosteroid treatment, and 5 (62.5%) patients had response, and the median time to response was 2.4 weeks (range, 1.9-12.3 weeks).

Immune-related hepatitis occurred in 12 (4.3%) patients, and the highest intensity of the event was grade 1 in 6 patients (2.2 %), grade 2 in 2 patients (0.7 %), grade 3 in 3 patients (1.1 %), grade 4 in 1 patient (0.2%), and grade 5 in 0 patient.

The median time to onset was 0.9 months (range: 0.4-8.4 months), and the median duration was 28.6 weeks (range: 0.4-52.9+ weeks). Four (1.4 %) patients needed to discontinue the investigational treatment permanently, and 2 (0.7 %) patients needed to interrupt the investigational treatment. No patient was treated with corticosteroids. 4 (33.3 %) patients had a response, and the median time to response was 11.5 weeks (range: 1.9-28.6 weeks). The overall safety data observed in this study was consistent with the available JS001 data. Adverse events are mainly graded 1-2, and most of them are abnormal investigations. A majority of death were found to have underlying disease, and most of the immune related adverse reactions were graded 1-2. After symptomatic treatment, these events were improved without impacts on the continued use of drugs, and incidence of AEs leading to discontinuation or interruption of investigational drug/AEs related to the investigational drug was low. No new, unexpected safety signal occurred as compared with similar products that had been approved overseas. The overall compliance of the investigational drug was good, and AEs were monitorable and controllable.

#### CLINICAL SAFETY

As of December 16, 2019, the summary on the safety of monotherapy in Chinese subjects was originated from 10 single-arm, open-label, single/multi-center clinical studies that have been completed or in which the enrollment was closed (CT1, CT2, CT3, CT4, CT5, CT6, CT7[CT7-1, CT7-2], CT9, CT12, CT14) and 1 randomized controlled study which included the monotherapy group and in which the enrollment was closed(CT8); at the same time, the interim analysis data of one randomized controlled study (CT17) which included the monotherapy group and in which the enrollment was ongoing were also included. There were a total of 985 subjects, including melanoma (N=322), nasopharyngeal cancer (N=200), oesophageal squamous cell carcinoma (N=65), adenocarcinoma gastric (N=63), squamous cell carcinoma of head and neck (N=34), non-small cell lung cancer (N=33), triple negative breast cancer (N=20), malignant lymphoma (N=24), soft tissue sarcoma (N=12), urothelial carcinoma (N=160), renal carcinoma (N=6), neuroendocrine tumour (N=40), pancreatic cancer (N=2) and other types of tumors (N=4). The administrated dose was: 0.3 mg/kg (N=3), 1 mg/kg (N=39), 3 mg/kg (N=851), 10 mg/kg (N=31) and 240 mg (N=61). 109 subjects with solid tumors from the phase I study in US were included in the overall evaluation of ADR.

In the 985 subjects, 964 subjects (97.9%) had at least one AE, 928 subjects (94.2%) had study related AE (if judged by investigators as definitely related, probably related, possibly related or likely unrelated, the causal relationship with the investigational drug would be classified as related). The majority of subjects reported AEs of CTCAE grade 1-2. The investigational drug related AEs with the reporting rate≥10% were anemia, elevated ALT, elevated AST, fever, hypothyroidism, weakness, elevated TSH, cough, rash, decreased appetite, pruritus, increased blood glucose, decreased white blood cell count and urine protein present. The reporting rate of the AE leading to discontinuation of investigational drug was 13.6% (134 subjects), and that of the AE leading to interruption of investigational drug was 15.6% (154 subjects). The reporting rate of CTCAE≥ grade 3 AE was 40.3%; 302 subjects (30.7%) had CTCAE≥ grade 3 AE related to the investigational drug, where the reporting rate ≥1% was seen in anemia, hyponatremia, lung infection and abnormal hepatic function measures. The reporting rate of SAE was 22.9%; 161 subjects (16.3%) had investigational drug related SAE, where the reporting rate ≥1% was seen in lung infection and abnormal liver function tests.

Systematic irAE adjudication had been made in 741/985 subjects receiving the recommended therapeutic dose (3 mg/kg or 240 mg) (not including CT8, CT14 and CT17). In the 741 subjects, 236 subjects (31.8%) had at least one category of irAE, the majority of which were grade 1-2. The most common irAE was hypothyroidism that occurred in 13.6% subjects. 1 subject died of interstitial pneumonia. Statistical summary of the incidence of various irAE was seen in Table 7.

Table 7 Summary of immune-related adverse events of Toripalimab

|  | **CTCAE**  **（N=741）** | | | | | |
| --- | --- | --- | --- | --- | --- | --- |
| **irAE classification**  **PT or classification term** | **1** | **2** | **3** | **4** | **5** | **Total** |
| Number of subjects with at least one irAE | 105 (14.2) | 89 (12.0) | 27 (3.6) | 14 (1.9) | 1 (0.1) | 236 (31.8) |
| **Immune-related endocrine disorders** | 58 (7.8) | 60 (8.1) | 2 (0.3) | 0 | 0 | 120 (16.2) |
| Hypothyroidism* | 47 (6.5) | 54 (7.3) | 0 | 0 | 0 | 101 (13.6) |
| Hyperthyroidism | 24 (3.2) | 11 (1.5) | 0 | 0 | 0 | 35 (4.7) |
| Insufficiency adrenal | 1 (0.1) | 2 (0.3) | 1 (0.1) | 0 | 0 | 4 (0.5) |
| Diabetes mellitus* | 0 | 1 (0.1) | 1 (0.1) | 0 | 0 | 2 (0.3) |
| Hypophysitis* | 1 (0.1) | 1 (0.1) | 0 | 0 | 0 | 2 (0.3) |
| **Immune-related cutaneous adverse reactions** | 42 (5.7) | 16 (2.2) | 2 (0.3) | 0 | 0 | 60 (8.1) |
| Rash* | 18 (2.4) | 7 (0.9) | 1 (0.1) | 0 | 0 | 26 (3.5) |
| Skin depigmentation* | 22 (3.0) | 1 (0.1) | 0 | 0 | 0 | 23 (3.1) |
| Pruritus | 6 (0.8) | 8 (1.1) | 1 (0.1) | 0 | 0 | 15 (2.0) |
| **Immune-related hepatitis** | 20 (2.7) | 13 (1.8) | 9 (1.2) | 5 (0.7) | 0 | 47 (6.3) |
| Abnormal liver function tests* | 17 (2.3) | 11 (1.5) | 7 (0.9) | 2 (0.3) | 0 | 37 (5.0) |
| Damage liver | 1 (0.1) | 2 (0.3) | 0 | 3 (0.4) | 0 | 6 (0.8) |
| Drug-induced liver injury | 1 (0.1) | 0 | 1 (0.1) | 0 | 0 | 2 (0.3) |
| Hepatitis autoimmune | 1 (0.1) | 0 | 0 | 0 | 0 | 1 (0.1) |
| Hepatic necrosis | 0 | 0 | 1 (0.1) | 0 | 0 | 1 (0.1) |
| **Immune-related colitis** | 4 (0.5) | 2 (0.3) | 1 (0.1) | 0 | 0 | 7 (0.9) |
| Diarrhea | 4 (0.5) | 2 (0.3) | 1 (0.1) | 0 | 0 | 7 (0.9) |
| **Immune-related nephritis** | 2 (0.3) | 0 | 3 (0.4) | 0 | 0 | 5 (0.7) |
| Blood creatinine increased | 1 (0.1) | 0 | 2 (0.3) | 0 | 0 | 3 (0.4) |
| Urea blood elevated | 1 (0.1) | 0 | 1 (0.1) | 0 | 0 | 2 (0.3) |
| Creatinine renal clearance decreased | 0 | 0 | 1 (0.1) | 0 | 0 | 1 (0.1) |
| **Immune-related myocarditis** | 2 (0.1) | 0 | 1 (0.1) | 0 | 0 | 3 (0.4) |
| Myocarditis | 2 (0.1) | 0 | 0 | 0 | 0 | 2 (0.3) |
| Autoimmune myocarditis | 0 | 0 | 1 (0.1) | 0 | 0 | 1 (0.1) |
| **Immune-related myositis** | 13 (1.8) | 9 (1.2) | 3 (0.4) | 3 (0.4) | 0 | 28 (3.8) |
| Blood creatine phosphokinase increased | 13 (1.8) | 9 (1.2) | 3 (0.4) | 3 (0.4) | 0 | 28 (3.8) |
| Myositis | 0 | 0 | 1 (0.1) | 0 | 0 | 1 (0.1) |
| **Immune-related pancreatitis** | 10 (1.3) | 3 (0.4) | 5 (0.7) | 5 (0.7) | 0 | 23 (3.1) |
| Increased amylase | 9 (1.2) | 2 (0.3) | 3 (0.3) | 5 (0.4) | 0 | 18 (2.2) |
| Lipase increased | 1 (0.1) | 0 | 3 (0.4) | 4 (0.3) | 0 | 7 (0.7) |
| Pancreatitis | 1 (0.1) | 1 (0.1) | 0 | 0 | 0 | 2 (0.3) |
| Acute pancreatitis | 0 | 2 (0.3) | 0 | 0 | 0 | 2 (0.3) |
| **Immune-related pneumonia** | 6 (0.8) | 4 (0.5) | 2 (0.3) | 0 | 1 (0.1) | 13 (1.8) |
| Interstitial lung disease | 3 (0.4) | 4 (0.5) | 2 (0.3) | 0 | 1 (0.1) | 10 (1.3) |
| Pulmonitis | 3 (0.4) | 0 | 0 | 0 | 0 | 3 (0.4) |
| **Other immune-related adverse reactions** | 0 | 0 | 1 (0.1) | 2 (0.3) | 0 | 3 (0.4) |
| Iritis | 0 | 1 (0.1) | 0 | 0 | 0 | 1 (0.1) |
| Uveitis | 0 | 0 | 1 (0.1) | 0 | 0 | 1 (0.1) |
| Platelet count decreased | 0 | 0 | 0 | 2 (0.3) | 0 | 2 (0.3) |
| *represents use of categorical terms:  Hypothyroidism: hypothyroidism, autoimmune thyroiditis and thyroid disorder  Diabetes mellitus: diabetes mellitus and hyperglycaemia  Hypophysitis: hypophysitis and hypopituitarism  Rash: rash, rash generalised, rash maculo-papular, rash pruritic and dermatitis acneiform  Skin depigmentation: skin depigmentation, vitiligo, skin hypopigmentation and leukoderma  Abnormal liver function tests: alanine aminotransferase increased, aspartate aminotransferase increased, blood bilirubin increased, hepatic function abnormal and bilirubin conjugated increased | | | | | | |

Overall, the adverse event observed in the pooled safety data was mainly abnormality in various examinations, mostly grade 1-2 in severity or consistent with the characteristics of underlying disease. The immune-related adverse reactions were consistent with that reported for products in the same class and showed a good tolerability. Overall, the adverse event was controllable.

Please see the Investigator’s Brochure for the details.

## Study rationale and benefit-risk assessment

### Rationale for evaluation of JS001 as monotherapy in patients with solid tumors

The clinical data emerged in the field of tumor immunotherapy have demonstrated that a focus on treatment improving the response of T cell to the tumor may bring significant survival benefit for patients with advanced malignancies. Therefore, immunomodulation is a new therapeutic strategy with a good prospect for cancers and may improve the anticancer activity. Several anti-PD-1/PD-L1 monoclonal antibodies have been approved for the treatment of a variety of advanced solid tumors in Europe and America.

The results of preclinical pharmacodynamic study show that recombinant humanized anti-PD-1 monoclonal antibody (JS001) can activate T lymphocyte and is significantly active in inhibition of tumor growth in NSG mice when combined with CTL. In the head-to-head in vitro and in vivo pharmacodynamic trials with Nivolumab, a better druggability is shown, and an excellent safety profile is seen in the chronic toxicity study in primates. Based on the results in the above studies, the first human study of JS001 in patients with advanced solid tumors after failure of treatment is initiated.

According to the data from the first human study of JS001 at present, no DLT is observed, the existing data on safety and pharmacokinetics demonstrate the safety of JS001 is acceptable. Based on the results of safety and pharmacokinetics in the previous phase I study of JS001, 3mg/kg (administrated once every two weeks) or 240mg fixed dose (administrated once every three weeks) is determined as the recommended dose for monotherapy.

### Rationale for evaluation of JS001 combined with the 1st-line standard of care in patients with solid tumors

For patients with locally advanced or metastatic non-small cell lung cancer, platinum-based two-drug chemotherapeutic regimen is still the standard 1st-line chemotherapeutic regimen. However, the survival benefit from cytotoxic chemotherapy reaches plateau and there is a large room left for improving the outcome. Through the exposure of immune system to high-level tumor antigens, the killing effect of cytotoxic chemotherapy on tumor cells (TC) can be reasonably predicted; by the inhibition of PD-L1/PD-1 signaling pathway, tumor-specific T cell immunity may be recovered by cytotoxic chemotherapy in this environment, producing a deeper and more persistent response than standard chemotherapy alone^[36,37]^.

As mentioned above, on the basis of the data of KEYNOTE-021 G in 2017, Pembrolizumab in combination with chemotherapy has been approved by the Food and Drug Administration (FDA) for the first-line therapy of metastatic non squamous NSCLC patients without EGFR or ALK gene mutation. It was fully approved in August 2018 based on the confirmatory results of KEYNOTE-189.

Keynote-407 Study compared Pembrolizumab combined with Carboplatin +Paclitaxel or nab-paclitaxel versus placebo combined with Carboplatin+Paclitaxel or nab-paclitaxel in the treatment of previously untreated locally advanced or metastatic lung squamous cell carcinoma. ORR of Pabolizumab + Chemotherapy versus chemotherapy alone was 58.4% and 35.0% respectively, P = 0.0004. 65.8% patients receiving Pembrolizumab + chemotherapy and 45.6% patients receiving chemotherapy alone had a median duration of response ≥ 6 months.

The positive results were obtained in the phase PIII studies of similar immunotherapy combined with 1L standard chemotherapy (IMPOWER150 and IMPOWER131). Thus, immunotherapy combined with 1L standard chemotherapy will become a new standard treatment for NSCLC in the future.

### Rationale for allowing patients to continue receiving JS001 treatment after being evaluated as progressive disease in accordance with RECIST v1.1 criteria and irRECIST criteria

As progression of disease (judged by radiological evaluation for the first time) does not necessarily indicate failure of immunotherapy, the evaluation criteria on routine remission may be unable to evaluate the effect of immunotherapeutic agent adequately. Due to delayed T cell response and potential immunomodulatory activity, an early transient increase is seen in tumor burden, which is called pseudoprogression^[38]^. Kurra et al. Reported that 356 patients with lung cancer were enrolled in a retrospective cohort study, including 103 patients with non-small cell lung cancer and 6 patients with small-cell lung cancer, 6% of these patients showed evidence of decrease of target lesion after early increase of tumor burden or emergence of new lesions according to RECIST criteria, i.e. where the condition of pseudo-progression or delayed response are likely to occur ^[39]^. In addition, in some NSCLC patients whose condition is relieved, biopsy is performed when enlarged lesion or radiologically new lesion appears, the result of biopsy shows immunological cells contained in the lesions with no active tumor cells^[40]^. Due to the possibility of pseudo-progression/tumor immunological infiltration, the patients receiving JS001 are permitted to consider the continuation of the investigational treatment after obvious radiological progression in this study, if a favourable benefit/risk ratio is considered after judgement, when the investigators judge the subject can still benefit from the investigational treatment.

Moreover, irRECIST will be used to evaluate the secondary efficacy endpoints for JS001 (ORR, PFS). New lesion is allowed to be included in the total tumor burden after baseline in accordance with irRECIST criteria. In addition, it is also strongly recommended: the patients with condition remission should undergo biopsy for the enlarged or new lesion after the evidence of progression of disease appears, if appropriate, as to confirm the progression of disease and better instruct the subsequent treatment.

### Rationale for collection of archived and fresh tumor samples and blood sample for biomarkers

Development of predictive diagnosis and analysis method (can prospectively identify patients who may respond to JS001 treatment) can help screen patients who are more likely to benefit from this treatment. Analysis of tumor tissue available prior to treatment, at tumor response and/or progression of disease (PD) will help elucidate the potential predictive parameters related with the reponse and efficacy related resistance of JS001. The resulting conclusion will be helpful for development of a method to improve the antitumor immunotherapy for cancer patients.

Presently, the treatment of lung cancer has entered the era of immunotherapy, programmed death receptor 1 (PD-1) and programmed death ligand 1 (PD-L1) inhibitors show excellent superiority in efficacy and quality of life compared to traditional chemotherapy. The effective rate of these drugs in unselected patients with lung cancer is approximately 20%, which has become the recommended choice for the first-line and second-line treatments of certain patients with lung cancer ^[41-43]^. However, there are no specific markers for predicting the efficacy of immunotherapy, and definite markers can be used to screen patients who will be responsive to immunotherapy and benefit patients to the greatest extent. PD-L1 expression, tumor mutational burden and gene mutation repair ability have become the hotspots of researches on predictive markers of efficacy of immunotherapy.

The expression level of PD-L1 in tumor cells, as an important part of PD-1 / PD-L1 signaling pathway, is a potential biomarker of immune checkpoint inhibition therapy. In the KEYNOTE series of studies on PD-1 inhibitor Pembrolizumab in the treatment of patients with non-small cell lung cancer ^[44-66]^, the objective response rate (ORR), median progression-free survival (PFS) and overall survival (OS) of patients receiving Pembrolizumab were significantly improved with the increase of expression level of PD-L1; especially, ORR of patients with PDL1 expression > 50% was 45.3% in the KEYNOTE-001 clinical study, and that of PD-L1 expression < 1% was only 10.7%. In clinical study CheckMate-017 and CheckMate-057 of PD-1 inhibitor Nivolumab in the treatment of NSCLC patients, there was no significant correlation between the expression of PD-L1 in patients with lung squamous cell carcinoma and efficacy of Nivolumab ^[47]^; in lung adenocarcinoma, ORR and median OS of patients with PD-L1 expression > 5% were 36% and 18.1 month respectively; while ORR and median OS of patients with PD-L1 expression < 5% were only 10% and 9.7 months respectively^[48]^.

The tumor mutational burden (TMB) refers to the total number of somatic gene coding errors, base substitution, gene indels errors detected per million bases. Theoretically, the higher the TMB is, the more new tumor associated antigens are produced, and the more likely it is to stimulate immune response, and with the elimination of inhibitory immune signals, better therapeutic effect will be achieved^[49]^.

Compared to other types of tumors, the lung cancer exhibits higher mutational burden load ^[50]^, and the potential biomarkers widely studied are tumor mutational burden (TMB), microsatellite instability-high (MSI-H) and mismatch-repair deficiency (dMMR). In CheckMate-026 study ^[51]^, median PFS and ORR in high TMB group (> 200 non synonymous mutations) were 9.7 months and 47% respectively, being significantly higher than that in low TMB group (4.2 months and 28%). In PD-L1 unselected population in KETNOTE-001 Study ^[44]^, patients with high TMB who received pembrolizumab also showed more durable clinical benefits. Further studies on lung cancer treatment ^[52-53]^ showed that high PD-L1 expression did not coincide with high TMB group, and there was no correlation between them.

The study data of studies on correlation of blood-tumor mutational burden (bTMB) with improvement of efficacy of Atezolizumab in second-line or above NSCLC therapy (POPLAR and OAK studies) published at the conference of European Society for Medical Oncology (ESMO) recently^[54]^ showed that patients with high bTMB achieved more PFS and OS benefits after being treated with Atezolizumab.

# Study Objectives and Endpoints

This study aims to evaluate the efficacy and safety of Toripalimab Injection (JS001) versus placebo combined with first-line standard chemotherapy in treatment-naive advanced non-small cell lung cancer and thus provide sufficient evidences for supporting the benefits and risks of JS001 combined with 1st-line chemotherapy in the treatment of NSCLC.

## Study Objectives and Endpoints

### Primary Objectives

- To evaluate progression-free survival (PFS) of Toripalimab Injection (JS001) versus placebo combined with first-line standard chemotherapy for treatment-naive advanced non-small cell lung cancer.

### Secondary Objectives

- To evaluate the overall survival (OS), objective response rate (ORR) per RECIST1.1 criteria, duration of response (DOR), disease control rate (DCR) and time to response (TTR) of Toripalimab Injection (JS001) versus placebo combined with first-line standard chemotherapy for treatment-naive advanced non-small cell lung cancer;
- To evaluate the safety and tolerability of JS001 combined with chemotherapy versus placebo combined with first-line standard chemotherapy for treatment-naive advanced non-small cell lung cancer;

### Exploratory Objectives

- To evaluate PFS, ORR, DOR, DCR and TTR of Toripalimab Injection (JS001) versus placebo combined with first-line standard chemotherapy for treatment-naive advanced non-small cell lung cancer according to iRECIST criteria;
- To evaluate the immunogenicity of JS001, and explore the potential relationship between its immunogenic response and the safety and efficacy;
- To explore the population with the best predictive efficacy through biomarker analysis (including but not limited to PBMC, PD-L1, WES, RNASeq and others).

### Primary Endpoints

- Progression free survival (PFS) evaluated by investigators according to the response evaluation criteria in solid tumors (RECIST 1.1).

### Secondary Endpoints

- Overall survival (OS);
- PFS and duration of response (DOR) evaluated by the Blinded Individual Review Committee (BIRC) based on RECIST1.1 criteria;
- Objective response rate (ORR) evaluated by investigators and BIRC based on RECIST1.1 criteria;
- Disease control rate (DCR) evaluated by investigators and BIRC based on RECIST1.1;
- Time to response (TTR) evaluated by investigators and BIRC based on RECIST1.1;
- Safety: overall incidence of adverse events (AEs); incidence of grade 3 and above AEs; incidence of serious adverse events (SAEs); incidence of AEs leading to termination of the investigational drug; incidence of AEs leading to interruption of the investigational drug;

### Exploratory Endpoints

- To evaluate PFS, ORR, DOR, DCR and TTR based on iRECIST criteria.
- To evaluate the immunogenicity of JS001, and explore the potential relationship between its immunogenic response and the safety and efficacy;
- Exploratory analysis of potential efficacy-related biomarkers. PD-L1, WES and RNASeq in tumor tissues, peripheral plasma ctDNA sequencing and characteristics of other biomarkers as well as the analysis of correlations between biomarkers and efficacy or safety.

# Study Design

## Overall Design

This is a randomized, double-blind, placebo-controlled, multi-center, phase III clinical study to evaluate the efficacy and safety of Toripalimab Injection (JS001) or placebo combined with first-line standard chemotherapy in treatment-naïve advanced non-small cell lung cancer (NSCLC), and evaluate the population with the best predictive biomarkers.

About 450 subjects with advanced non-small cell lung cancer without sensitive EGFR mutation and ALK fusion will be randomized into two groups by 2:1, JS001 combined with the first-line standard chemotherapy will be given in the study group whereas placebo combined with the first-line standard chemotherapy will be given in the control group. The stratification will be based on the following factors:

- PD-L1 expression (TC≥1% vs TC＜1%);

Note: the patients whose PD-L1 fails to be be evaluated will be included in the group with TC < 1%

- Smoking status (often smoking vs no smoking or infrequent smoking);

Note: the smoking status is to be denoted with the smoking index, smoking index = number of cigarettes smoked per day × years of smoking. Frequent smoking is defined as ≥ 400 cigarettes per year.

- Pathological type (squamous cell carcinoma vs non-squamous cell carcinoma).

Note: the patients with adenosquamous cell carcinoma will be stratified based on squamous carcinoma and treated with the chemotherapy regimen of nab-paclitaxel combined with carboplatin.


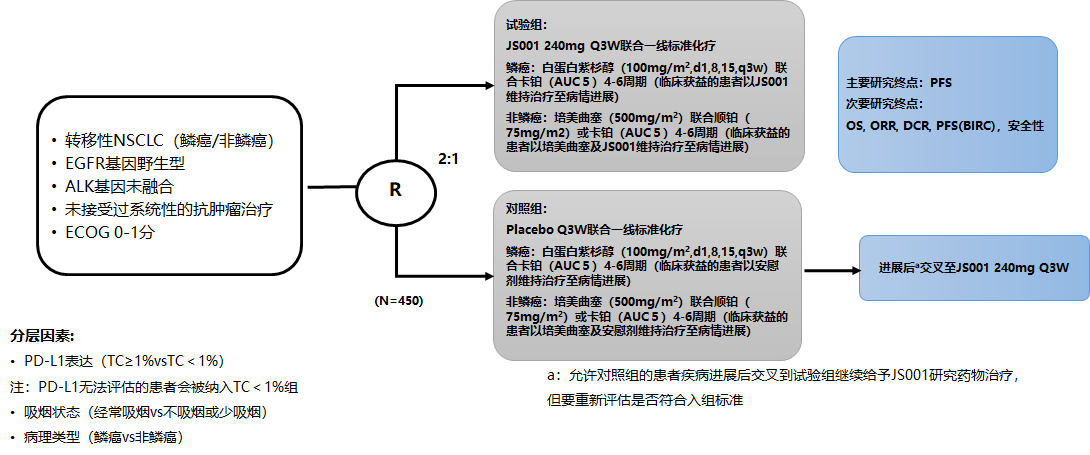


Figure 3 Study Flow Chart

**Patients will receive the following treatment:**

JS001 240 mg/placebo is given intravenously, 21 days as a cycle, the treatment will continue until the subject reaches the criteria of drug discontinuation, i.e., documented progressive disease, unacceptable adverse events (AEs), not suitable to continue treatment as considered by investigators, withdrawal of informed consent by the subject, accumulated 2-year JS001 treatment or other reasons stipulated in the protocol.

The patients with squamous cell carcinoma will receive the following chemotherapy regimen: nab-paclitaxel + carboplatin (4-6 cycles): on Day 1, Day 8 and Day 15, nab-paclitaxel will be given by IV infusion at a dose of 100 mg/m^2^ (the administration of nab-paclitaxel on Day 15 is up to the investigator), and carboplatin AUC 5 (Day 1), 21 days as a cycle.

The patients with non-squamous cell carcinoma will receive the following chemotherapy regimens: pemetrexed + cisplatin (4-6 cycles) : pemetrexed 500mg/m^2^ and cisplatin 75mg/m^2^ via IV infusion on Day 1 of each 21-day cycle.

Pemetrexed + carboplatin (4-6 cycles) : pemetrexed 500 mg/m^2^ and carboplatin AUC 5 via IV infusion on Day 1 of each 21-day cycle. The patients without progression of disease after 4-6 cycles of therapy can continue to receive pemetrexed alone for maintenance therapy.

Cisplatin or carboplatin will be given at the discretion of the investigators.

**The obtaining of tumour tissue specimen:**

The tissue samples will be collected for biomarker evaluation.

If the tumor lesion can be obtained when the efficacy is evaluated as partial response (PR) and/or progression of disease (PD), it is encouraging for the subjects to participate in the optional biomarker study in this study. If pseudo-progression is suspected, it is advisable to conduct biopsy of the lesion for confirmation of diagnosis if the condition allows.

Biomaker analyses will be conducted in this study, which will be performed for archived tissue specimens (requirement at enrollment), and prospectively collected blood samples and freshly acquired tumor samples. The samples collected will be analyzed for PD-L1 expression, TMB (using NGS/WES) and other potential markers in the central laboratory designated by the sponsor, as for the evaluation of the expression of biomarkers and patient’s outcome, including but not limited to the correlation between response and progression of disease.

### Bases for Continued Treatment allowed in the Selected Patients with Progressive Disease

A large amount of clinical evidences showed that some subjects receiving immune system-stimulating drugs might have progression of disease prior to clinically objective response and/or stable disease (in accordance with the traditional response evaluation criteria). This is one rare phenomenon observed in the early JS001 studies. Two hypotheses have been proposed to explain that. Firstly, the exacerbation of inflammation in tumors may enlarge tumors, which is characterized by enlargement of index lesions and new non-index lesion. As time goes on, the inflammatory part of malignancies and masses may reduce later, obvious signs of clinical improvement appear. Or, in some individuals, the kinetics of tumor growth may exceed the rate of development of anti-tumor immune activity initially. In case of sufficient time, the antitumor activity will prevail and become obvious clinically. Therefore, following progression of disease determined by the investigator based on RECIST 1.1, if the investigator determines that the subject can still benefit from JS001 treatment, JS001 study drug treatment may continue to be administered. If a subject is subject to further iRECIST confirmed progressive disease (iCPD) as defined in the iRECIST after continuing the study drug treatment, the subject must withdraw from the study treatment.

### Rationale for Dose Selection of JS001

The data on the samples in each dose group of JS001 (dose level 0.3-10mg/kg once in every two weeks and 240 mg fixed dose) from three phase I dose-escalation clinical studies (CT1, CT2, CT3) were summarized for the pooled analysis of pharmacokinetics. The study results showed a basically linear pharmacokinetics after administration of 1, 3 and 10 mg/kg JS001 iv drip for clinical subjects; and a certain non-linear pharmacokinetic profile when the dose was increased to 10 mg/kg. JS001 shows an obvious “long-acting” characteristic of antibody drugs, and the plasma drug concentration basically reaches a steady state after continuous administration of JS001 iv drip for consecutive 3-4 doses.

The steady-state lowest plasma drug concentration ranged from 20-40 μg/mL at the dose of 3 mg/kg once in every two weeks. The in vitro study showed that PD-1 receptor on the surface of T cells could be saturated when the concentration of JS001 was >20 nM or 3 μg/mL, and the concentration of JS001 was usually maintained at 25 μg/mL in peripheral blood, in consideration of the restricted entry of antibody macromolecules in the tumor microenvironment, so as to ensure the complete occupancy of PD-1 in the lymphocytes in the tumor microenvironment. Each phase I study showed that the complete occupancy of PD-1 receptor could be maintained throughout the treatment in different dose groups (0.3, 1, 3 and 10 mg/kg, once in every two weeks). In accordance with the above pharmacokinetic results and data on receptor occupancy, the dose of 3 mg/kg once in every two weeks was supported to be selected as the dose for CT4 phase II pivotal study.

360 mg fixed dose once in every in every three weeks was explored in two early studies (CT5 and CT7), the preliminary pharmacokinetic results from 9 patients showed that compared with 3 mg/kg once in every two weeks (from 11 patients in CT1), the steady-state peak concentration was increased to 153%; the steady-state peak concentration was increased to 133%, and AUC 0-Day 85 was increased to 138%. The PK model predicted a similar trough concentration (~32 ug/mL) and exposure level for 240 mg once in every three weeks and 3 mg/kg once in every two weeks.

In the comprehensive analysis on the above results of pharmacokinetics and receptor occupancy of JS001 and the results from the phase II pivotal study of melanoma, JS001 240 mg fixed dose once in every three weeks was supported as the recommended dose for the phase III clinical study.

## Test Method

### Patient Screening

Investigators are responsible for keep records of all the screened patients, including those who are enrolled in the trial and excluded from the trial. The above recording should be included in the master test documentation.

Prior to the initiation of screening procedure, patients must sign the informed consent form (ICF). After the signature of ICF, each potential subject will be subject to the inclusion and exclusion criteria evaluation that will be recorded in the electronic case report form (eCRF).

The complete history must be acquired from each patient, the content of record includes all the previous therapies, concurrent medications and all the drugs used within 30 days prior to the first dose.

A full physical examination, including vital signs, body weight and height will be performed for each patient. Demographic information of the patients will also be recorded. One standard 12-lead electrocardiography (ECG) will be performed within 14 days prior to Day 1, as to find out the patients with clinically important abnormalities that have not been diagnosed yet and are considered by investiagtors that may exclude them from participation in the trial. A tissue or blood sample for laboratory testing is required.

At enrollment, the ECOG performance status will be evaluated for each patient (See annex 2: Eastern Cooperative Oncology Group (ECOG) - Performance Status Scale).

Throughout the trial, all the patients enrolled will be evaluated on a regular basis according to the schedule in advance (see Appendix 1: study flow chart).

### Randomization and Treatment Assignment

The subjects will be randomized on a 2:1 ratio to one of the two treatment groups after enrollment.

| ARM 1:  JS001 combined with chemotherapy group | JS001 240 mg + chemotherapy with a cycle of 3 weeks (Q3W) and 4-6 cycles in total, then JS001 240mg Q3W will be given until progression, intolerable toxicity or the subject's withdrawal of informed consent (pemetrexed maintenance treatment is allowed for patients with non squamous cell carcinoma free of progressive disease after 4-6 cycles of treatment) | 300 |
| --- | --- | --- |
| ARM 2:  Placebo combined with chemotherapy group | The 240 mg placebo + chemotherapy with Q3W for 4-6 cycles is administered , followed by placebo 240 mg Q3W until progression, intolerable toxicity, or subject 's withdrawal of informed consent (the patients with non squamous cell cancer free of progressive disease after 4-6 cycles are allowed to receive pemetrexed maintenance treatment), PD patients are allowed to take the crossover administration to JS001 240 mg until progression, intolerable toxicity or the subject's withdrawal of informed consent. | 150 |

### Stratification

The stratification of patients will be based on the following factors: PD-L1 expression (TC≥1% vs TC＜1% Note: the patients whose PD-L1 cannot be evaluated will be included in the TC＜1% group), smoking status (frequent smoking vs no smoking or infrequent smoking), pathological type (squamous cell carcinoma vs non-squamous cell carcinoma).

### End of Study

The end time of the study is defined as the date of the last patient's last visit (LPLV), or the date of collection of the last data point required for statistical analysis, whichever comes earlier. The last data point required for statistical analysis will be described in detail in the Statistic Analysis Plan. The sponsor is entitled to terminate the study at any time due to specific reasons (e. g, major safety issue, force majeure, etc.). The duration of the main study is estimated to be about 27 months from the randomization of the first subject.

If there are subjects who are still receiving the investigational drug treatment at the end of study, they will be transferred to an extension study to continue to receive the investigational drug, until inability to continue benefit as judged by investigators, progression of disease, intolerable toxicity, withdrawal of informed consent or death. The sponsor may make the decision on termination of the study at any time.

# Study Population

The patients with histologically and / or cytologicallly confirmed and untreated locally advanced (Stage IIIB or IIIC) and non-small cell lung cancer at Stage IV (according to the staging of eighth edition of AJCC) who aren't allowed to receive radical concurrent chemoradiotherapy will be selected; and patient's tumor must be free from sensitive EGFR mutations or ALK fusion. Specific inclusion/exclusion criteria are as follows:

## Patient Inclusion Criteria

**Only the patients meeting all the following criteria can be eligible to participate in the trial:**

1. Have full knowledge on this study and are willing to sign informed consent form (ICF);
2. The patients with locally advanced (Stage IIIB or IIIC) or Stage IV non-small cell lung cancer (according to the staging of eighth edition of AJCC) confirmed histologically and/or cytologically, inoperable and unable to receive radical concurrent chemoradiotherapy (according to the staging of eighth edition of AJCC); the tumor of patients must be free of sensitive EGFR mutation and ALK fusion, and the test is not mandatory for patients with squamous cell carcinoma.
3. At least one measurable lesion (in accordance with RECIST 1.1);

Note: the lesion previously irradiated can not be regarded as target lesion, unless clear progression of disease after radiotherapy;

1. No history of any systemic anti-tumor therapy. If the adjuvant/neoadjuvant therapy is completed at least 6 months prior to the occurrence of metastasis, the subject receiving the adjuvant/neoadjuvant therapy meets the condition;
2. It is agreed to provide the formalin-fixed tumor tissue specimens or fresh biopsy tissues from tumor lesions after diagnosis of metastasis (at least 12 sections; if approved by the sponsor medical monitor, less than 12 unstained sections are acceptable); if the recent biopsy is not feasible, the biopsy sample obtained before adjuvant/neoadjuvant chemotherapy can be accepted (i.e., archived specimen);
3. Age of 18-75 years, male or female;
4. ECOG Scores 0-1;
5. Expected survival ≥ 3 months;
6. Laboratory test values within 7 days before the first dose must meet the following criteria:

a. Neutrophils ≥ 1.5 ×10^9^/L;

b. Platelets ≥ 100×10^9^/L;

c. Hemoglobin ≥ 90 g/L (no packed red blood cell transfusion within 4 weeks);

d. Blood creatinine ≤ 1.5×upper limit of normal (ULN) and creatinine clearance ≥ 50 mL/min;

e. Total serum bilirubin ≤ 1.5×ULN;

f. Aspartate aminotransferase (AST) and alanine aminotransferase (ALT) ≤ 2.5×ULN; ALT and AST ≤ 5×ULN for patients with hepatic metastasis; ALP ≤ 2.5×ULN (or ≤ 5×ULN for patients with bone metastasis);

g. International normalized ratio (INR) or prothrombin time (PT) ≤ 1.5×ULN, unless the subject is receiving anticoagulant therapy; activated partial thrombin time (aPTT) ≤ 1.5×ULN, unless the subject is receiving anticoagulant therapy.

1. Women of childbearing potential must have a confirmed negative result of serum pregnancy test and agree to use effective contraceptive measures during the use of investigational drug and within 90 days after the last dose. The women of childbearing potential in this protocol are defined as sexually mature women: 1) no hysterectomy or bilateral ovariectomy, 2) continuous natural menopause for less than 12 months (fertility can not be excluded in case of amenorrhoea following cancer therapy) (i.e., menses occurred at any time in the previously consecutive 12 months). If the male subjects' female parters are of childbearing potential, the subjects must agree to use adequate contraceptive measures from the start of the first dose of investigational treatment to 90 days after the last dose of investigational treatment.

## Patient Exclusion Criteria

**Patients meeting any one of the following conditions can not be included in this clinical study:**

1. Known allergy to recombinant humanized anti-PD-1 monoclonal antibody drug and its components;
2. Histologically or cytopathologically confirmed combination with small cell lung cancer component or sarcomatoid lesion;
3. Current participation in and receiving other investigational treatment, or participation in treatment of one investigational drug within 4 weeks prior to administration of JS001;
4. Previous use of systematic chemotherapy for advanced NSCLC; targeted therapy for advanced NSCLC (including but not limited to erlotinib, crizotinib, cetuximab);
5. Previous use of anti-PD-1 antibody, anti-PD-L1 antibody, anti-PD-L2 antibody or anti-CTLA-4 antibody (or any other antibody acting on T cells for synergetic stimulation or the checkpoint pathway, such as IDO, IL-2R, GITR);
6. Chest (lung) radiotherapy > 30 Gy within 6 months prior to the start of investigational treatment. However, the locally palliative radiotherapy for bone metastasis is excluded;
7. Use of traditional Chinese medicine with antitumor indication or the drugs with immunomodulatory effect (including thymosin, interferon and interleukin within two weeks prior to the first dose, or performance of major surgery within 3 weeks before the first dose or no complete recovery from the previous surgery.
8. Active tuberculosis (TB), receiving anti-tuberculosis therapy currently or within one year prior to screening;
9. Subjects with active or untreated central nervous system (CNS) metastasis will be excluded;

Note: If the CNS tumor metastasis is limited to supratentorial site and /or cerebellum of the subject, who has received adequate treatment, and matained clinical stability (imaging testing, enhanced MRI or CT preferred) for at least 4 weeks, and clinical symptoms of the subject including the nervous system can recover to NCI-CTC AE ≤ grade 1 at least 2 weeks prior to the first dosing, such subject is allowed to participate in the study, while the radiotherapy and / or surgery for CNS metastases must be performed if new asymptomatic CNS metastasis is detected during the screening period,. After the end of treatment in the above patients, there is no need to receive the additional brain scanning before enrollment if all the other criteria are met.

1. Spinal cord compression for which operation and/or radical radiotherapy has not been given, or no clinical evidence of stable disease for ≥4 weeks prior to enrollment after treatment for previously diagnosed spinal cord compression
2. Uncontrolled pleural effusion, pericardial effusion, or ascites requiring repeated drainage (once per month or more frequently); the subjects with stable symptoms for at least 2 weeks after drainage can be enrolled;
3. Uncontrollable or symptomatic hypercalcemia (ionized calcium > 1.5 mmol/L or calcium > 12 mg/dL or corrected serum calcium > ULN);
4. Clinically uncontrolled active infection, including but not limited to acute pneumonia;
5. Uncontrollable major epileptic seizure or superior vena cava syndrome;
6. Previous or current combination with other malignancies (except radically treated non-melanoma without recurrence evidences, including skin basal cell carcinoma or squamous cell carcinoma, breast/cervical carcinoma in situ, superficial bladder cancer and other carcinomas in situ);
7. History of interstitial pneumonia, idiopathic pulmonary fibrosis, organized pneumonia (e.g., obliterating bronchiolitis), drug induced pneumonia, idiopathic pneumonia or evidence of active pneumonia or other moderate and severe lung diseases seriously influencing the lung function during chest CT scanning for screening;
8. Known hepatic diseases of clinical significance, including untreated active viral hepatitis, alcoholic hepatitis or other hepatitis, liver cirrhosis, hereditary liver disease;

Note 1: Active viral hepatitis is defined as hepatitis B virus (HBV) infection with hepatitis B virus deoxyribonucleic acid (HBV DNA) higher than the lower limit of detection; or hepatitis C virus (HCV) infection (positive anti-HCV antibody and the quantitative detection results of HCV RNA higher than the lower limit of detection);

Note 2: Patients with recovered previous hepatitis B virus (HBV) infection or HBV infection (defined as positive hepatitis B core antibody [HBcAb] and negative HBsAg) can participate in this study. Prior to enrollment, HBV DNA detection must be performed for such patients (patients with HBV DNA lower than the lower limit of detection);

Note 3: The patient with positive HBsAg, and HBV DNA below lower limit of detection is allowed to be enrolled, the risk will be assessed by the investigator, and such patient should receive anti-HBV treatment during the whole study treatment to avoid virus activation;

Note 4: patients with positive HCV antibody test results can be enrolled in this study only when the test of polymerase chain reaction of HCV RNA is negative.

1. Known human immunodeficiency virus (HIV) infection (known positive HIV antibody);
2. Serious cardiovascular disease, for example, New York Heart Association (NYHA) grade 2 or >2 heart failure, unstable angina pectoris, unstable arrhythmia, myocardial infarction or cerebrovascular accident within 6 months prior to randomization;
3. Use of systemic immunosuppressive therapy (i.e., use of corticosteroid or immunosuppressant) for any active autoimmune disease within two years prior to Day 1 of the 1st cycle;

① The autoimmune diseases include but are not limited to interstitial pneumonia, uveitis, enteritis, hepatitis, hypophysitis, nephritis, hyperthyroidism;

② Patients with leukoderma or childhood asthma that has been completely relieved and does not need any intervention in adulthood can be enrolled;

③ Patients receiving replacement therapy (e.g., thyroxine, insulin or physiological corticosteroid replacement therapy for adrenal/pituitary insufficiency) are eligible to participate in the study;

④ Patients who need to use bronchodilator, inhaled steroid or local steroid injection intermittently for asthma will not be excluded from the study.

1. Being unable or unwilling to use folic acid or vitamin B_12_ Injection (applicable to non-squamous cell carcinoma patients who planned to receive pemetrexed therapy);
2. Vaccination of live-virus vaccine within 30 days after the start of planned treatment; use of seasonal influenza vaccine free of live virus is allowed;
3. Previous allogeneic stem cell or solid organ transplantation;
4. Women who are pregnant or at lactation or have the potential of pregnancy show positive pregnancy test prior to the first dosing; patients who have childing-bearing potential but are not willing to receive contraception measures or whose sex partners are not willing to receive contraception measures;
5. Any other disease or condition of clinical significance that can affect the compliance with the protocol (e.g., history of psychosis or drug abuse), cannot benefit from this clinical study, or affect the signature of the informed consent form (e.g., drug addiction and drug abuse), or is unsuitable to be involved in this clinical trial, as considered by investigators (including but not limited to: Abnormal laboratory result, clinically active diverticulitis, intra-abdominal abscess, intestinal obstruction, peritoneal metastasis);

## Eligibility criteria for crossover treatment to JS001 treatment period - inclusion criteria for subjects previously randomly enrolled to AMR2 (placebo plus chemotherapy group, control group) :

1. As evaluated by investigators, the subjects must have clear radiological progression of disease documented during placebo combined with first-line standard chemotherapy or maintenance therapy;
2. The subjects did not receive other systematic anticancer therapy except the chemotherapeutic drugs in this study , and the previous treatment, including chemotherapy and palliative radiotherapy, must be completed at least 3 weeks prior to the administration of JS001;
3. The adverse event related with chemotherapy or pallative radiotherapy must have been relieved to grade 1 or baseline at the entry in crossover treatment;
4. Any major surgery must be completed at least 28 days prior to the first dose of JS001;
5. No previous anti-PD-1 or anti- PD-L1 treatment;
6. ECOG PS score 0-1;
7. The laboratory data on the eligibility of crossover therapy must meet the following criteria and should be obtained within 14 days prior the start of JS001 treatment:

Peripheral hemogram: white blood cell (WBC) ≥ 3.0×10^9^/L, neutrophil (ANC) ≥ 1.5×10^9^/L, platelet (PLT) ≥ 100×10^9^/L, hemoglobin (Hgb) ≥ 90 g/L;

Renal function: serum creatinine ≤ 1.5 × ULN or calculated creatinine clearance > 40 mL/min (using Cockcroft-Gault formula);

Hepatic function: AST/ALT ≤ 2.5 × ULN in subjects without hepatic meatastasis, AST/ALT ≤5 × ULN in subjects with hepatic metastasis; total bilirubin ≤ 1.5 × ULN (total bilirubin < 3.0 mg/dL for subjects with Gilbert syndome).

1. Man of reproductive ability or women of pregnant potential must use highly effective contraceptive methods (e.g. oral contraceptives, intrauterine contraceptive device, abstinence of sexual intercourse or barrier contraception in combination with spermatocide) during the trial, and continue contraception for 90 months after the end of treatment;
2. Being voluntary to participate in the study, sign the informed consent form, with good compliance and willingness to cooperate with follow-up.

## Criteria for the discontinuation of investigational treatment

Subjects were discontinued from investigational drug if any of the following circumstances occurred:

- Progression of disease as judged in accordance with RECIST 1.1 criteria, and inability to benefit from the continuation of JS001 treatment according to the investigator’s judgment;
- Recurrence of progression of disease after continuous administration of JS001 following iRECIST confirmed progression of disease (iCPD);
- Adverse events;
- The subject (or legal representative) requests to terminate the investigational drug;
- Serious violation to the study protocol for which the investigators judge that it is necessary for subjects to withdraw from the study;
- Other conditions for which the investigators judge that it is necessary for subjects to withdraw from the study;
- Suspension of study medication greater than maximum suspension duration specified in the protocol;
- Pregnant subjects (female subjects);
- The subject who receives 2 years of JS001/ placebo treatment (patients in the control group can receive the treatment up to 2 years after crossover therapy with JS001);
- Any of withdrawal criteria specified in the protocol is satisfied.

The investigators should record the main reasons for the termination of investigational treatment in eCRF. The patients who terminated the investigational product will not be replaced.

All patients who terminated the investigational treatment must have a 30-day safety visit after the last dose of investigational drug. Except for the patients who have terminated the therapy with the study drug due to disease progression, the subject will continue to receive the tumor evaluation, follow-up of anti-tumor drugs and survival until the disease progresses, or the subject withdraws from the study, or the subject dies, whichever comes earlier.

## Criteria for Study Withdrawal by Subjects

Subjects can freely discontinue the study participation at any stage during the study. Investigators should inquire the reason for drop-out of the study, and ask subjects to return to the study center for the last visit if possible, and follow up any adverse event that is not relieved as far as possible. If subjects are not able to attend the visit at the scheduled time, investigators need to do everything possible to contact them, and urge them to return to the study center to complete corresponding visit as soon as possible. Investigators also have the right to terminate the subject’s treatment prematurely for the reasons below or other reasons including:

- Death;
- Withdrawal of informed consent by the subjects;
- Loss of follow-up (defined as inability to attend one visit specified in the protocol, and failure of contact with the subject or his/her family members through two or more ways for at least three times within three months after that visit);
- Study terminated by sponsor.

Patients with study withdrawal should always be asked about the reason(s) that will be recorded. Patients will not be followed up after withdrawal of informed consent for any reason, however, the sponsor still has the right to keep and continue to use the data collected prior to withdrawal of the informed consent. Patients who withdraw from the study will not be replaced.

## Management for Subjects Who Terminate the Treatment

The reason for termination of treatment should be recorded in the original medical record and eCRF, and the subsequent anticancer therapy and survival after termination of treatment should be recorded in the original medical record and eCRF.

## Randomization, Blinding and Unblinding

All patients will be assigned a screening number after the written informed consent is obtained. When the work during the screening period and confirmation on the eligibility requirements of all patients are completed, the patients will be assigned with random numbers (a different number different from the screening number) and will be randomly assigned to their treatment groups in a ratio of 2:1 to receive JS001+ chemotherapy as well as placebo + chemotherapy.

During the study, the sponsor, all patients, all staffs in the study centers, the client and its representatives (excluding IxRS suppliers and iDMC members) will remain blinded to treatment allocation until unblinding.

When the number of PFS events of 356 patients required for the primary efficacy analysis is reached, the sort-out and validation of all data are completed and the clinical database is locked, the sponsor will unblind and perform primary analysis on PFS.

The patient may be unblinded after disease progression to determine follow-up treatment. See Section 5.14 treatment after disease progression.

In the case of medical emergency, the investigators may apply for emergency unblinding for patient management needs (for example, when a serious adverse event occurs, and knowing the treatment allocation can affect the management of patient), and implement the emergency unblinding via Rave RTSM system, and they should contact and achieve agreement with the sponsor’s medical monitor(s). If the investigator desires to know the characteristics of the study drug for any other reason, such investigator should contact the medical monitor. The investigator should document and provide explanations for any ahead-of-time unblinding (for example, unexpected unblinding). The subjects with emergency unblinding must discontinue the investigational drug treatment immediately and withdraw from the study.

# Study Visits and Assessments

The patients enrolled in the study will be visited and evaluated on a regular basis according to the specified visit evaluation procedure during the trial. (See Appendix 1: Study Flow chart).

During conduction of the clinical trial, unscheduled evaluation will be performed if clinically indicated. The data of the unscheduled evaluation should be recorded in eCRF.

## Informed Consent Forms

The informed consent forms for this study involves the primary informed consent, informed consent form for optional biomarker subgroup study, informed consent form for JS001 cross therapy for control group after disease progression, and informed consent form for continual medication after disease progression. Patients (or authorized representative) will be requested by the investigators at the study site or their authorized personnel to sign on the informed consent form (ICF) approved by IRB. The patients may keep one signed informed consent form, and the other copy will be kept at the study site. The trial title, version number and date must be provided on each page of ICF. The informed consent must be obtained prior to any study specific procedures.

## Eligibility Criteria

All the inclusion and exclusion criteria will be reviewed by investigators or qualified designated personnel, as to ensure the subject meets the eligibility for enrollment in the trial.

## Assignment of Screening Number

All the subjects who have signed the informed consent will be given one unique screening number, which is used to identify subjects in all the procedures prior to randomization or treatment allocation. Each subject will be assigned only with a screening number. The screening number will be used to identify subjects in treatment allocation and all the subsequent procedures. The screening number must not be re-used for different subjects.

Subjects re-screened in the study should be assigned with a new screening number, and each patient can be re-screened at most once.

## Assignment of Treatment/ Randomization Number

All the subjects meeting the enrollment requirements will be allocated by randomization and given a treatment/randomization number. Once a treatment number had been assigned to a subject it could not be re-assigned to any other subject.

A single subject could not be assigned more than 1 treatment/randomization number.

## Medical History

The medical history includes the status of various concurrent diseases. Investigators need to acquire the overall history of previous and current diseases, as well as the data on currently ongoing medications. The key point of special focus should also be provided for the target tumor type.

## Demography

Demographic data includes the date of birth, ethnicity, age, and sex. After signature of ICF, the demographic data will be recorded at screening.

## Physical Examination

The systematic routine examination of main body systems (including head, eyes, ear, nose, throat, neck, heart, chest (including lungs), abdomen, four limbs, skin, lymph nodes, nervous system, and patient's general condition) will be performed, and the known drug reactions or other allergies of patients will be recorded. In subsequent follow-up, the repeated physical examination of the same system will be performed at screening (1-28 days prior to Day 1), prior to each dose of treatment and at the withdrawal from follow-up, as to observe a variety of clinically significant changes from baseline.

Investigators will perform full physical examination for subjects in screening period. The clinically significant abnormalities should be recorded in the history. A complete physical examination will be conducted as specified in the flow chart of the trial. In accordance with the study flow chart, investigators will perform targeted physical examination prior to administration of the investigational drug according to clinical indications, in the cycles with no need of full physical examination. The clinically significant abnormality newly discovered after the first dose of investigational drug should be recorded as adverse event.

## ECOG Score

ECOG PS score should be provided at screening (1-28 days prior to Day 1), prior to the start of each cycle of therapy and at withdrawal from the follow-up.

## Electrocardiogram

One standard 12-lead electrocardiograhy (ECG) should be performed within 14 days prior to Day 1, as to find out the patients with clinically significant abnormalities that have not been diagnosed yet. ECG examinations will be performed before dosing on Day 1 of each cycle (except cycle 1) and at the withdrawal follow-up visit.

## Vital Signs

Documented vital signs include: blood pressure, heart rate, body temperature, and respiration rate at baseline and subsequent follow-up visits. They will be measured once at screening (1-28 days prior to Day 1), prior to each dose of investigational treatment, at discontinuation of the drug and withdrawal from the follow-up.

## Laboratory Tests

The following laboratory evaluations must be performed throughout the trial.

The following assessment will be conducted in the laboratory where the study center is located:

1) Routine blood test: red blood cell count, hemoglobin, hematocrit, white blood cell count and differentiation (neutrophils, lymphocytes, eosinophils, monocytes, and basophils), and platelet count;

2) Serum pregnancy test for women of childbearing potential;

3) Blood biochemistry: total protein, albumin, globulin, blood glucose, total cholesterol, low density lipoprotein, high density lipoprotein, triglycerides, urea/ urea nitrogen, creatinine, alkaline phosphatase, lactic dehydrogenase, creatine kinase, creatine kinase isoenzyme, total bilirubin, direct bilirubin, indirect bilirubin, AST, ALT, calcium, phosphorus, magnesium, potassium, sodium, chloride, serum amylase, and uric acid;

4) Blood coagulation: international narmalized ratio (INR), prothrombin time (PT), activated partial thromboplastin time (aPTT);

5) Thyroid function test: thyroid-stimulating hormone (TSH), serum free triiodothyronine (FT3), serum free thyroxine (FT4);

6) Virological examination: hepatitis B markers (HBsAg, anti-HBs, HBeAg, anti-HBe, anti-HBc) (HBV DNA copies need to be detected in case of positive HBsAg and/or positive HBcAb), HCV antibody (HCV RNA needs to be detected in case of positive HCV antibody), HIV antibody;

7) Urine analysis: specific gravity, pH, urine glucose, protein, cast, ketone bodies, and blood cells (including urine red blood cells and urine white blood cells); if the urine protein test is ++ or more or the results are abnormal and clinically significant as judged by the doctor, then a 24 hour quantitative measurement of urine protein will be carried out.

8) Stool routine: color and shape of stoool, red blood cells, white blood cells, and occult blood;

Additionally, the biomarker analysis will be also planned, 8 mL peripheral blood will be collected and sent to the central laboratory (excluding the first blood collection, subsequent collections of these samples will be completely dependent on the times of the patient's voluntary provision), and 8 mL peripheral blood collection requires separation of plasma and PBMC. The collection purpose of the plasma involves the measurement of DNA TMB of circulating tumor and inflammatory factors; the collection purpose of PBMC collection, the investigation on the receptor expression and occupation of PD-1, and expression of other T cell receptors. See the laboratory manual for specific handling, transportation and preservation.

In terms of the blood collection and processing method, the laboratory procedure at the research institution should be followed.

In case that the patient is female, the patient must be surgically sterilized or have menopause for at least one year, or use effective contraceptive measures during the conduction of the trial and for consecutive 90 days after the last dose, in order to be enrolled in the trial. Male patients must agree to take effective contraception measures during the trial and for the consecutive 90 days after the last dose.

## Screening/Baseline Period (Day -28 to Day -1)

- All subjects must provide a written and signed ICF prior to any specific study evaluation and operation;
- Demographic data: including the date of birth, sex, and ethnicity.
- History of alcohol consumption and smoking;
- Past history (collection of all previous medical histories prior to the signature of ICF except this indication);
- Past tumor history: including diagnosis date of tumor, start/end date of prior therapy, optimal treatment evaluation, date of progressive disease. Radiotherapy history: including start/end date, total radiotherapeutic dose and radiotherapy site;
- Obtainment of tumor tissue samples: the biopsy needle should be used to collect samples or excised or cut tumor biopsy samples should be collected. The needle aspiration or cytological sample is generally unacceptable; normally the samples of bone metastases are not acceptable unless there are evident soft tissue components; all subjects should provide 12 tumor tissue sections for biomarker detection (less than 12 unstained sections are allowed to be submitted with the approval of the sponsor medical monitor). The requirements of tissue section sampling, sample preservation, transportation and analysis are detailed in the Laboratory Manual;
- Physical examination: including head, eyes, ear, nose, throat, neck, heart, chest (including lungs), abdomen, four limbs, skin, lymph nodes, nervous system, and patient's general condition.
- Vital signs;
- ECOG score: it is advisable to evaluate ECOG score by the same investigator throughout the study, refer to Appendix 2 for details;
- Laboratory examination: the laboratory examination will be completed in local laboratory of the study center and needs to be performed within 14 days prior to the first dose. Specific laboratory examination indexes include hematological examination, blood biochemistry, coagulation function, urine test, stool routine and serum pregnancy tests (within 72 hours prior to the first medication), virology detection and thyroid function test (similarly hereinafter), and the hematological examination, blood biochemistry and coagulation function should be checked within 7 days prior to the first medication.
- Hematological examination: red blood cell count, hemoglobin, hematocrit, white blood cell count and differentiation (neutrophils, lymphocytes, eosinophils, monocytes, and basophils), and platelet count;
- Blood biochemistry test: total protein, albumin, globulin, blood glucose, total cholesterol, low density lipoprotein, high density lipoprotein, triglycerides, urea/urea nitrogen, creatinine, alkaline phosphatase, lactic dehydrogenase, creatine kinase, creatine kinase isoenzyme, total bilirubin, direct bilirubin, indirect bilirubin, AST, ALT, calcium, phosphorus, magnesium, potassium, sodium, chloride, serum amylase, and uric acid;
- Routine urine test: specific gravity, PH, urine glucose, protein, cast, ketone body and hematocyte (including urine red blood cells and urine white blood cells). If the urine protein test is ++ or more or the results are abnormal as judged by the doctor, then a 24-h quantitative measurement of urine protein is required.
- Stool routine tests involve the color and shape of stool, red blood cells, white blood cells, and occult blood;
- Coagulation function: international normalized ratio (INR) or prothrombin time (PT), activated partial thromboplastin time (aPTT);
- Serum pregnancy test (if applicable);
- Virological examination: hepatitis B markers (HBsAg, anti-HBs, HBeAg, anti-HBe, anti-HBc) (HBV DNA copies need to be detected in case of positive HBsAg and/or positive HBcAb), HCV antibody (HCV RNA needs to be detected in case of positive HCV antibody), HIV antibody;
- Thyroid function: thyroid-stimulating hormone (TSH), free triiodothyronine (FT3), free thyroxine (FT4);
- The evaluation of left ventricular ejection function should be specifically noticed in cardiac ultrasonography;
- 12-Lead ECG;
- Pulmonary functions: forced vital capacity, forced mid-expiratory flow (FEF25-75), peak expiratory flow rate (FEF25-75), forced expiratory volume within 1 second and diffusing capacity of the lung for carbon monoxide (DLCO);
- Tumor evaluation: the tumor will be evaluated in accordance with RECIST 1.1 criteria and irRECIST criteria. CT or MRI scan of chest, abdomen, and pelvis should be carried out at screening (within 28 days before the first dose); in case of clinical indications, an appropriate method may be used to examine any other known or suspected disease sites, such as head MRI, bone scan, or neck CT scan; if due to routine diagnosis and treatment prior to signing the informed consent, the subject's tumor imaging examination was carried out within 4 weeks prior to enrollment (bone scan was acceptable) and at our study site, it is not necessary to carry out again.
- The inclusion/exclusion criteria will be reviewed to evaluate the eligibility of subjects;
- Subject enrollment: after the verification of the inclusion/exclusion criteria and confirmation of subject’s eligibility, the investigators should login EDC system to randomize the subjects on the same day of drug administration or within 3 days prior to drug administration;
- Concomitant medication/concurrent therapy: all drug therapies of subjects within 30 days prior to the first dose must be recorded in the case report form, including the generic name and daily dose, reasons for use of the drug, start and end dates;

## Visits during Treatment Period

- Physical examination (only targeted physical examination is to be completed);
- Vital signs;
- ECOG score;
- Laboratory test: except for C1D1, the laboratory test during the treatment period must be completed before each dose, and the blood collection time cannot be earlier than 3 days before the dose for hematologic, blood coagulation and biochemistry tests and 7 days before the dose for other laboratory tests. The dose can be started after the laboratory test results are judged as meeting the drug continuation criteria by investigators; the hematologic examination must be performed within 2 days before the administration of nab-paclitaxel on Day 8 and Day 15 of each cycle. If the dose interruption or adjustment occurs due to hematological toxicity, the routine blood test needs to be repeated every week until recovery to normal, in case of neutrophil ≤1.0×10^9^/L or platelet ≤50×10^9^/L, the frequency of re-examination needs to be increased (once in every 2-3 days); if ALT or AST elevation of three times or elevation of two times from baseline occurs during the trial, the frequency of examination needs to be increased (1-2 times per week recommended);
- Echocardiography: once in every two cycles in the first 12 cycles, and once in every three cycles afterwards;
- 12-lead ECG: investigators must review, sign and date all the ECG graphs for safety monitoring. The paper copy of ECG graphs will be maintained as the permanent study file at the study center. Any change in the shape of waveform or other ECG abnormalities must be recorded in eCRF.
- Thyroid function: it will be tested before administration in each cycle and at the end-of-treatment visit. In case of clinically significant change in thyroid function, it is advisable to request a consultation from the department of endocrinology and exclude pituitary function impairment;
- Tumor evaluation: the same radiological examination method should be used at baseline and subsequent evaluations, and evaluated by the same investigator as far as possible. The tumor evaluation should be performed once in every 6 weeks (window period: ± 7 days) in the first 12 months and thereafter once in every 9 weeks using the cycle calculated based on C1D1 and will not be affected by the discontinuation of the drug. Patients who are suspected with progressive disease prior to the next scheduled tumor evaluation should receive an unscheduled tumor evaluation. The radiological examination should proceed until disease progression confirmed by investigators (RECIST1.1 or iRECIST), start of a new antitumor therapy, withdrawal of informed consent, or death, whichever comes first.
- For the subject assessed by iRECIST, if clinical stability is confirmed, the disease progression should be confirmed by the study center within 1st recording of the progression with images. If the time that the participant who received confirmatory imaging examination is less than 4 weeks, it is not required to perform next scheduled tumor imaging examination, and the tumor imaging examination can be restarted at subsequent time point of imaging examination planned if the clinical condition is stable. If there is suitable tumor lesion for biopsy and the subject agrees on participation in the optional biomarker study in case of PR and/or PD, it is advisable to collect fresh tissue to send to central laboratory;
- Anti-drug antibody (ADA) and corresponding trough concentration: 3 mL whole blood will be collected before the first dose and within 60 min before dosing once in every 4 cycles of the first year during the investigational treatment, and the serum will be separated and sent to the central laboratory for testing; thereafter, the blood will be collected before dosing once in every 8 cycles, until the subjects complete the investigational treatment;
- Blood biomarker test: 8 mL whole blood needs to be collected and sent to the central laboratory within 60 min before the first dose, during treatment (synchronic to the tumor evaluation) and at progressive disease/drug discontinuation (end-of-treatment visit), and the specimen after separation will be used for PBMC detection, TBNK lymphocyte subset and other blood biomarker tests (see Section 7 for the details);
- Treatment with investigational product;
- Concomitant medications/accompanied treatments;
- Adverse events;

## Treatment after Disease Progression

When progression of disease per RECIST1.1 criteria occurs, the study can be unblinded according to the investigator’s judgment:

- If the subject who has received the placebo + chemotherapy (control group) reaches the criterion for crossover therapy, such subject is allowed to receive JS001 crossover therapy after obtaining informed consent for crossover treatment, i.e. intravenous infusion of 240 mg JS001 with 21 days as a cycle and maximum cycle not to be greater than 2 years until reaching the withdrawal standard. The tumor images will be redirected to the baseline that time point. The imaging examination should be conducted every 9 weeks (± 7 days) after receiving JS001 monotherapy and crossover therapy. If the investigator considers that clinical benefits can still be obtained from the therapy after the occurrence of disease progression defined in RECIST1.1, the clinically stable patients may continue therapy until iRECIST confirmed progressive disease (iCPD) upon the obtainment of informed consent from the subject to continue medication after disease progression;
- The subject who has received JS001+ chemotherapy (experimental group) can continue to be administered with JS001 monotherapy (simultaneous discontinuation of chemotherapy) if the investigator considers that clinical benefits can also be obtained from therapy upon the obtainment of informed consent of such subject for medication continuation after disease progression, PD will be confirmed by the above mentioned within 4-8 weeks according to iRECIST, and the discontinuation of js001 treatment is recommended if the iRECIST confirmed progressive disease (iCPD) occurs. Maximum treatment of JS001 should not be greater than 2 years (including the duration for combined chemotherapy and monotherapy) in total.

Whereas, when the investigator confirms the disease progression according to the RECIST1.1 criteria, if the subject remains clinically stable, they can still remain blind to the assigned treatment. After obtaining the informed consent of the subjects to continue medication after disease progression, they can continue to receive the trial treatment according to their randomly assigned treatment plan. The investigator will confirm PD according to iRECIST within 4-8 weeks, the patient may be subject to unblinding when iCPD is confirmed, the patient in the control group will receive JS001 crossover therapy according to the process above mentioned (in conformity with crossover therapy criterion), and the patient in the experimental group is advised to terminate current therapy.

The therapy and imaging evaluation of the subject after disease progression are shown in Figure 4.


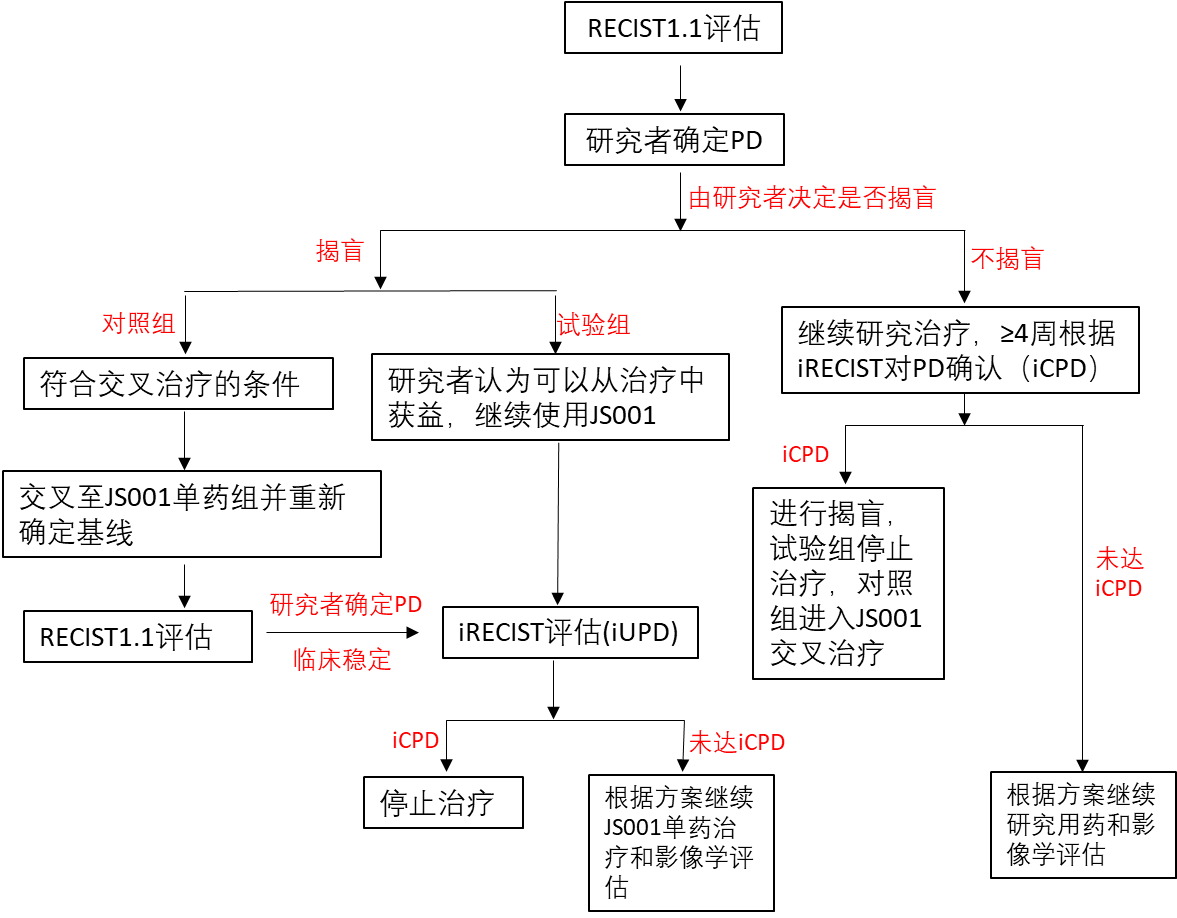


Evaluation by RECIST 1.1

Confirmation of investigator on PD

Determination of the investigation on the feasibility of unblinding

Unblinding

Control group

Trial group

Conditions of crossover treatment compliant

Non-unblinding

The investigator considers the benefits can be made from the therapy and continue to use JS001

Continue the study theray, confirm PD (iCPD) according to iRECIST for the cycle ≥4 weeks

Crossover therapy to JS001 monotherapy group and redefine baseline

Evaluation by RECIST 1.1

Evaluation by iRECIST (iUPD)

Conduct the unblinding, the experimental group discontinue the therapy and the control group enters JS001 crossover therapy

Confirmation of investigator on PD

Clinically stable

iCPD not reached

Discontinuation of therapy

Continuation of JS001 monotherapy and imaging evaluation according to the protocol

Continuation of medication study and imaging evaluation according to the protocol

iCPD not reached

Figure 4 Subsequent imaging evaluation and treatment of the subject after the first imaging evaluation on PD by the investigator

The investigator of the center should make clinical determination on that if the subject suffering from disease progression for the first time (i.e. PD confirmed in RECIST 1.1) or after receiving crossover therapy can continue to benefit from JS001 therapy based on the overall clinical conditions of the subjects including physical status, clinical symptoms and laboratory results. Clinical stabilization is defined by:

- - Evidence of clinical benefit, as considered by investigators;
  - Lack of clear symptoms and signs indicative of disease progression (including exacerbation of the results in laboratory examinations, e.g., new or aggravated hypercalcaemia);
  - No decrease of ECOG physical performance score attributable to progression of disease;
  - No progression of disease at key anatomical sites (e.g., leptomeningeal lesions), as this progression can not be managed in accordance with the medical interventions allowed in the study protocol;

If there are approved therapeutic regimens available, patients should sign the informed consent form at the first progression of disease and confirm their willingness to postpone these therapeutic regimens in order to continue the investigational treatment.

## Crossover-treatment Period

The investigators confirm that subjects in the control group who receiving the unblinding after PD (see Section 5.14 for unblinding conditions) would have the opportunity to receiveJS001 monotherapy in the crossover phase. It isn't mandatory to take crossover therapy, and it is allowed to take JS001monotherapy in crossover manner based on the determination of the investigator and the consent of the sponsor if the subject's condition is stable and meets the criteria for crossover therapy.The subject who permanently discontinues the treatment due to adverse events, withdrawal of informed consent or by reason of any non-progressive disease isn't eligible for crossover therapy. The crossover subject is not allowed to start JS001 therapy 21 days after the last administration upon chemotherapy regardless of the time of progression.

The crossover subject will start the crossover phase as described in the flowchart for crossover therapy in Appendix 1. Maximum duration of the first medication of crossover therapy should not be greater than 56 days after the last treatment with study drug before crossover therapy. All procedures and evaluations completed at the time of withdrawal from main study can be used for baseline assessment during the crossover phase of the study. Of the tumor imaging to determine the disease progression is completed within 28 days before the first administration of JS001, such imaging can be used as a new baseline imaging examination in the crossover phase; or otherwise a new baseline imaging examination must be performed before JS001 monotherapy.

The tumor evaluation will not be baselined again after the the patient in the experimental group receives JS001 monotherapy upon unblinding, iRECIST confirmed PD (iCPD) will be performed within 4-8 weeks after the last imaging examination , subsequent examination will be conducted during the time window of original imaging examination if PD is not confirmed, and the subjects who have been subject to the imaging confirmation within 4 weeks can be exempted.

## End of Treatment Visit

When all the study therapies are discontinued, the patients will be told to return to the study center for the end-of-treatment visit within 3 days after the discontinuation of treatment, regardless of the reason for the discontinuation of treatment. If the decision is made to discontinue the treatment at one visit (e.g., confirmed disappearance of clinical benefit [patients who continue to receive JS001 after progression of disease evaluated in accordance with RECIST1.1] or progression of disease), this visit can also be used as the end-of-treatment visit.

- Physical examination;
- Vital signs;
- ECOG score;
- Laboratory tests;
- Cardiac ultrasonography;
- 12-Lead ECG;
- Thyroid function;
- Tumor evaluation: the same radiological examination method should be used at baseline and subsequent evaluations, and evaluated by the same investigator as far as possible. It can be exempted in case it is completed within 28 days prior to end of treatment (EOT), otherwise it should be completed in the 30-day observational period after the last dose or before the start of a new antitumor therapy (whichever comes first).
- If there is suitable tumor lesion for biopsy and the subject agrees on participation in the optional biomarker study in case of PD, it is advisable to collect fresh tissue specimen to send to central laboratory for testing;
- Anti-drug antibody (ADA) and corresponding trough concentration: 3 mL whole blood will be collected and the serum will be separated (this test is not performed at the end-of-treatment visit for the patients in the control group who receive the crossover-treatment);
- Blood biomarker detection (optional):collection of 8 mL whole blood (the patient in the control group will not be tested at the end-of-treatment visit);
- Concomitant medications/accompanied treatments;
- Collection of AEs/SAEs.

The patients who discontinue the investigational treatment should continue to undergo the tumor evaluation, just as they are still receiving the investigational product, if the discontinuation is not caused by progression of disease, until the start of a new antitumor therapy, radiological progression of disease in accordance with RECIST 1.1 and iRECIST criteria, withdrawal of informed consent, death or termination of study by the sponsor (whichever comes first).

## Safety Follow-up Visit

The necessary safety follow-up visit should be performed 30 days after the last dose of investigational drug or before the start of a new antitumor therapy (whichever comes first). If the end-of-treatment visit falls within the safety follow-up period, the safety visit does not need to be performed again. See annex 1 for details.

## Follow-up Visit

The subject who ends the treatment due to non-progressive disease will be subject to the follow-up visit to collect subsequent anti-tumor treatment, survival status and tumor evaluation, and the follow-up visit requirements will be summarized in the flow chart for investigation in Annex 1.

## Survival Follow-up

For the subjects with progression of disease or starting a new antitumor therapy, the data on the subsequent antitumor therapies and survival of patients will be collected once in every three months using the end-of-treatment visit as the starting point.

# Iinvestigational Product and Research Method

## Clinical Drug Supplies

### Toripalimab Injection (JS001) and Placebo

Table 8 Toripalimab Injection (JS001) and placebo

| Investigational drug/control drug | MFR. | Supply concentration and formulation |
| --- | --- | --- |
| Toripalimab Injection (JS001) | Suzhou Zhonghe Biosciences Co., Ltd. | Sterile liquid containing 240 mg Toripalimab Injection (JS001).  The nominal concentration is 40 mg/mL, containing 20 mM sodium citrate, 2.5% (w/v) mannitol, 50 mM NaCl and 0.02% (w/v) polysorbide 80. The pH is 6.0. Each bottle contains 6 mL of effective volume. |
| Placebo | Suzhou Zhonghe Biosciences Co., Ltd. | Sterile liquid containing 20 mM sodium citrate, 2.5% mannitol, 50 mM NaCl, 0.02% polysorbate 80. The pH is 6.0. Each bottle contains 6 mL of effective volume. |

Toripalimab Injection (JS001) or placebo sterile liquid is placed in a 6 mL glass bottle (West® bottle plug and aluminum plastic combinational cap) and supplied to the study center through a separate multi-patient kit. 1 vial in 1 drug box. A white label is pasted on the drug box and vial of Toripalimab Injection (JS001) or placebo. Other codes are not used.

The label on the vial contains the following information: protocol number, content of Toripalimab Injection (JS001) or placebo, batch number, expiry date, storage conditions and medication instructions. The content of the label is in accordance with Good Manufacturing Practice (GMP) and local regulatory requirements. Label text need to be translated into local language if applicable.

Toripalimab Injection (JS001)/placebo will be provided by the sponsor.

### Pemetrexed

Refer to the requirements in a local label for detailed information for preparation and storage of Pemetrexed for clinical use.

Dosage form: sterile lyophilized powder for injection

Ingredients: Pemetrexed Disodium

Storage conditions: store under room temperature, protected from light

Pemetrexed is provided by the sponsor.

### Nab-paclitaxel

Refer to the requirements in a local label for the detailed information on the preparation and storage of nab-paclitaxel for clinical use.

Dosage form: sterile injection

Ingredients: each bottle contains 100 mg paclitaxel and about 900 mg human serum albumin. Paclitaxel serves as an active component of the drug, and human serum albumin (HSA) plays a role in dispersing, stabilizing particles and carrying the main component. Chemical name of paclitaxel: 5β, 20-epoxy -1, 2α, 4, 7β, 10β, 13α-hexahydroxy taxane-11-ene-9-keto-4, 10-diacetate-2-benzoate-13-(2R, 3S)-N- benzoyl -3- phenyl isoserine ester.

Storage conditions:

The date indicated on the label is considered to be stable when original package of the product is unpacked and stored in the temperature ranging 20 ℃ to 30 ℃. Neither freezing nor refrigeration adversely affects the stability of the product.

Stability of suspension in the bottle after dispersion and dissolution: the product should be immediately used after being dispersed and dissolved, but the medicine bottle containing the suspension should be put back in the original package to avoid light if it is not used immediately, and the suspension should be kept in the refrigerator at 2 ℃-8 ℃ for up to 8 hours.

Stability of suspension in infusion bag after dispersion and dissolution: The suspension prepared according to requirements shall be used immediately after being transferred from the medicine bottle to the infusion bag. The suspension in the infusion bag can be stored for 8 hours at room temperature (20 ℃-25 ℃) and indoor illumination.

Nab-paclitaxel will be provided by the sponsor.

### Carboplatina

Refer to the requirements in a local label for detailed information on preparation and storage of Carboplatin for clinical use.

Dosage form: sterile injection

Ingredients: Cis-I,I-cyclobutane dicarboxylate diamineplatinum, edetate disodium

Storage conditions: store in a cool (not more than 20 °C) and dark place, protected from light

Carboplatin is provided by the sponsor.

### Cisplatin

Refer to the requirements in a local label for the detailed information on preparation and storage of Carboplatin for clinical use.

Dosage form: sterile injection

Ingredients: (cis) dichlorodiamineplatinum, sodium chloride, polyethylene glycol 400

Storage conditions: protected from light, sealed preservation

Carboplatin is provided by the sponsor.

## Management of Investigational Product

### Reception and Storage

The RTSM System will be used for distribution of drugs. The investigational product shall be transported to the research site by the logistics company of a third party with transportation qualification. After receiving the investigational product, a designed receiver of the study center shall check the transportation state and verify the number and state of drug vials, complete the inventory and drug accountability records, and finally fax the signature-confirmed packing list to the sponsor to confirm the receipt of the investigational product.

The investigational product is only for use of the study, and is managed by a designee authorized by the investigator. An inventory record is made at each visit of patients for drug administration, to control completely distribution and use of the investigational product.

### Disposal

All unused Toripalimab Injection (JS001) and chemotherapeutics shall be stored under the specified storage conditions in a storage place designed by the clinical pharmacology facility of the study center. The sponsor shall regularly arrange a designee of a third party to recover any remaining solution and empty vials for used Toripalimab Injection. If the drug is lost or damaged, then a detailed record for such situations are made. At the end of the study, any remaining, unused investigational product, as well as any remaining solution and empty vials of used drug are recovered to the sponsor in a centralized way or destroyed by a designed third party; the remaining solution and empty vials of used chemotherapeutics can be destroyed in the study center, the destroy record documents shall be filled and the destroy procedures shall be assured to abide by the routine medical waste disposal practices of the hospital.

### Preparation Method and Records

#### JS001 Injection

The injection is a colorless, sterile injection, and is slightly opalescent. Prior to use, a careful examination is needed to confirm that each vial of Toripalimab Injection is free of damage, solidification, opacity or precipitation.

Administration dose (mg) = 240 mg

Under sterile conditions, dilute with saline (0.9% Sodium Chloride Solution) to the final concentration of 1-10 mg/mL. It is suggested to draw a corresponding volume of the investigational product and inject into 100 mL of saline solution, and gently invert to mix the dilute solution for 3-5 times, avoiding shaking vigorously; after mixed well, carry out intravenous drip by an online filter (0.2 or 0.22 μm). The first dose is dripped intravenously over at least 60 min. As the injection does not contain any antimicrobial preservative, a solution must be prepared under sterile conditions.

If not used immediately, a prepared dilute solution may be stored in a refrigerator at 2-8°C for not more than 16 h, or under room temperature for not more than 4 h.

At the end of intravenous drip, the saline solution must be used to flush the tube, and meanwhile, the vials and packages of used investigational product shall be recovered by the drug keeper.

If the first dose is tolerated, and no infusion-related adverse event occurs, then JS001 may be infused over 30 min; if infusion reactions occur in the prior infusion, then JS001 may be dripped intravenously over at least 60 min.

The chemotherapy will be started at least one hour after the end of JS001 infusion and various vital signs have no abnormality.

Each vial of JS001 shall be limited for only one subject, and shall not be interchanged. Any remaining solution of JS001 after administration cannot be reused.

#### Pemetrexed

White to pale yellow or yellowish green, lyophilized solid powder.

Administration dose (mg) = 500 mg/m^2^ * body surface area (BSA).

The formula for calculating body surface area is: BSA (m^2^) = 0.20247 x height (m)^0.725^ x weight (kg)^0.425^.

The required quantity of the investigational product is reconstituted with 0.9% Sodium Chloride Injection to produce a clear solution; the solution is colorless to yellow or yellowish green. An adequate volume of the reconstituted Pemetrexed is transferred, and is diluted in 0.9% Sodium Chloride Injection. After dilution, the infusion may be stored for 24 h in a refrigerator or under room temperature.

Pemetrexed shall be dripped intravenously at least 1 hour after the end of JS001 administration. Each vial of pemetrexed shall be limited for only one subject, and shall not be interchanged. Any remaining solution of Pemetrexed after administration cannot be reused. After the end of intravenous drip to the patients, the vials and packages of the drug shall destroyed in a local place according to the relevant procedures.

#### Nab-paclitaxel

This product is white or pale yellow sterile freeze-dried lumps or powder.

Administration dose (mg) = 100 mg/m^2^ * body surface area (BSA) on Day 1, 8 and 15 in a cycle of 21 days.

The formula for calculating body surface area is: BSA (m^2^) = 0.20247 x height (m)^0.725^ x weight (kg)^0.425^.

Preparation of drug prior to intravenous infusion: this agent is a kind of aseptic freeze-dried block or powder before being dispersed and dissolved. Please carefully read the following instructions before dispersing and dissolving the product to avoid mistakes:

1. Disperse and dissolve each bottle with 20 mL of 0.9% sodium chloride injection under aseptic operation.
2. Use the sterile syringe to inject 20 mL 0.9% sodium chloride injection slowly along the inner wall of the bottle, and the duration of the operation should not be less than 1 minute.
3. Don't directly inject 0.9% Sodium Chloride Injection into the freeze-dried block/powder to avoid foaming.
4. Let the vial stand for at least 5 minutes after completing the injection to ensure that the freeze-dried block / powder is completely saturated.
5. Then, gently swirl or invert the vial slowly for at least 2 minutes until the complete dispersion and dissolution of all freeze-dried lumps/powder in the vial to avoid the production of foams.
6. Place still for 15 minutes in case of frothing until the foam subsides.

Nab-paclitaxel on the first day of each cycle should be administered intravenously at least 1 hour after ending the administration of JS001. Each vial of nab-paclitaxel shall be limited for only one subject, and shall not be interchanged. Any remaining solution of nab-paclitaxel after administration cannot be reused. After the end of intravenous drip to the patient, the vials and packages of the drug shall be destroyed in a local place according to the relevant procedures.

#### Carboplatin

The product is a clear, colorless or pale yellow solution.

The dose of Carboplatin for AUC 5 is calculated by Calvert equation (Calvert et al. 1989):

Calvert Equation

Total Dose (mg) = (Target AUC) × (Glomerular Filtration Rate [GFR]+25)

Notes: In Calvert equation, GFR for computation of an AUC-based dose is not more than 125 mL/min. Accordingly, maximum dose of carboplatin = 5 × (125 + 25) = 750 mg.

In this protocol, it is considered that GFR is equivalent to creatinine clearance (CRCL). In accordance with the research institute's guideline or according to Cockcroft and Gault (1976) method, the following equation is used to calculate CRCL:

CRCL= $\frac{（140 - age）（\mathrm{wt}）}{72 \times Scr}$(× 0.85, for females)

Among them: CRCL = creatinine clearance, expressed in mL/min

age = patient's age, expressed in year.

wt = patient's body weight, expressed in kg.

Scr = serum creatinine, expressed in mg/dL.

or CRCL=$\frac{（140 - age）（\mathrm{wt}）}{0.814 \times Scr}$ (× 0.85, for females)

Among them: CRCL = creatinine clearance, expressed in mL/min

age = patient's age, expressed in year.

wt = patient's body weight, expressed in kg.

Scr = serum creatinine, expressed in µmol/L.

The required drug is diluted with 250 mL-500 mL 5% Glucose Injection to produce a clear solution; the solution is colorless to pale yellow.

Carboplatin should be intravenously administered after ending the administration of pemetrexed or albumin-bound paclitaxe, and each bottle of carboplatin is only used for one subject, and shall not be interchanged. Any remaining solution of carboplatin after administration cannot be reused. After the end of intravenous drip to the patient, the vials and packages of the drug shall be destroyed in a local place according to the relevant procedures.

#### Cisplatin

This product is light greenyellow to light yellow and slightly sticky and clear liquid.

Administration dose: the administration dose will be calculated based on body surface area, 75 mg/m^2^ iv drip, once in every three weeks, on Day 1 of each cycle.

Intravenous infusion is usually used for drug administration. Sufficient hydration treatment must be performed 2-16 hours before and within at least 6 hours after drug administration. This product should be intravenously dripped after being diluted with normal saline or 5% glucose solution.

Cisplatin shall be intravenously dripped after the completion of pemetrexed administration. Each vial of cisplatin shall be limited for only one subject, and shall not be interchanged. After the end of intravenous drip to the patient, the vials and packages of the drug shall be destroyed in a local place according to the relevant procedures.

## Method of Administration

### JS001/Placebo

JS001/placebo 240 mg is infused intravenously in a cycle of 21 days, the treatment will continue until the subject reaches the criteria on the discontinuation of the drug, i.e., documented progression of disease, unacceptable adverse events (AEs), not suitable to continue treatment as considered by investigators, withdrawal of informed consent, accumulated 2-year treatment of JS001 received by subjects, or other reasons specified in the protocol.

### First-line Standard Chemotherapy

#### Patients with squamous cell carcinoma will receive the following chemotherapy regimen:

Nab-paclitaxel + carboplatin: albumin paclitaxel in 100 mg/m^2^ (the dosing of nab-paclitaxel on Day 15 depends on the investigator) should be intravenously infused on Day 1, Day 8 and Day 15 ; carboplatin AUC 5 will be dosed on Day 1 of each 21-day cycle, for no more than 4-6 cycles.

#### Patients with non-squamous cell carcinoma will receive the following chemotherapy regimen:

Pemetrexed + cisplatin: Pemetrexed 500 mg/m^2^ and cisplatin 75 mg/m^2^ via IV infusion on Day 1 of each 21-day cycle, for no more than 4-6 cycles.

Pemetrexed + carboplatin: Pemetrexed 500 mg/m^2^and carboplatin AUC 5 via IV infusion on Day 1 of each 21-day cycle, for no more than 4-6 cycles.

The patients without progression of disease after 4-6 cycles of therapy can continue to receive pemetrexed alone for maintenance therapy.

Cisplatin or carboplatin will be give at the discretion of the investigator.

The formula for calculating body surface area (BSA) is calculated with Dubois formula: BSA (m^2^) = 0.20247 x height (m)^0.725^ x weight (kg)^0.425^

### Continue the treatment with JS001 after disease progression

Large amount of evidence shows that few subjects receiving immunotherapy can still have clinical benefit after acquisition of the initial evidence on progression of disease (PD). The treatment and evaluation of with patient with disease progression are detailed in Section 5.14.

## Precautions for use

The subject will receive blind JS001 in 240 mg (D1) or placebo (D1) combined with chemotherapy (the protein-binding paclitaxel100 mg/m^2^(D1, D8,D15) + Carboplatin AUC5 (D1) Q3W with 4-6 cycles in total is to be selected for squamous cell carcinoma, and Pemetrexed in 500 mg/m^2^ for maintenance therapy with Pemetrexed was allowed, followed by blind JS001 240 mg (D1) or placebo (D1) Q3W is to be received until progression / completion.

JS001 or placebo will be administered before chemotherapy.

### Precautions for administration of JS001

1. Prophylaxis Medication

JS001 cannot be administered intravenously or injected rapidly; a peripheral or central intravenous access should be established. Prior to infusion, adequate Epinephrine, Intravenous Diphenhydramine Hydrochloride, or other antiallergic drugs, as well as rehabilitation devices shall be well prepared for use against occurrence of serious allergic reactions. After intravenous infusion, the intravenous access shall be maintained open for drug administration if necessary. If no complication occur, then the intravenous access may be removed through 1-h observation after the end of infusion.

1. Care during Intravenous Care

The first dose of the drug shall be carried out by an experienced doctor. Before and during intravenous drip as well as at least 1 h after the end of intravenous drip, the subject shall be closely monitored for various vital signs (a ECG monitor is suggested to be used to monitor the body temperature, respiration, blood pressure, and heart rate), as well as complexion, sweating or headache to early discover any signs of infusion reactions.

Some infusion reactions may occur in the subsequent phase of drug use, even though no grade of infusion reactions occurs after use of the first dose, and subsequent course of drug use shall also be carried out under care by a doctor.

1. Management of Infusion Reactions

If infusion reactions occur in a subject, an antiallergic drug shall be used preventively prior to administration of each dose of the investigational product from then on. In such case, infusion reactions may occur possibly during intravenous drip of the antibody injection, despite the drugs for prevention of allergic reactions, such as antihistamine drug and glucocorticoid, are used prior to administration of investigational product.Refer to Section 9.1.2.2 for specific treatment.

Table 9 Infusion of the First and Subsequent Doses of JS001

| **Infusion of the first dose** | **Infusion of the subsequent doses** |
| --- | --- |
| - No pretreatment drugs are allowed. - Within 60 min prior to infusion and 30min (± 5min) after infusion, the patient's vital signs (heart rate, respiration rate, blood pressure, and body pressure) shall be recorded. - JS001 shall be dripped intravenous over at least 60 min. - If clinical indications occur, the subject's vital signs must be monitored closely during infusion, and also monitored at 15, 30, 45, and 60 min (± 5 min) after infusion. - The patients will be informed of possible delayed symptoms post transfusion and are required to contact the doctor after the symptoms appear. | - If the patient develops infusion reactions in the previous infusion, then an appropriate pretreatment shall be adopted in subsequent infusions as directed by the investigator. - In 60 min prior to infusion, the patient's vital signs shall be recorded. - If the first dose is tolerated, and no infusion-related adverse event occurs, then JS001 may be infused over 30 min; if infusion reactions occur in the prior infusion, then JS001 may be dripped intravenously over at least 60 min. - If infusion reactions occur in the prior infusion or if clinical indications occur in 30 min (± 5 min) after infusion, the patient's vital signs should also be monitored. |

### Precautions for use of Pemetrexed + Carboplatin or Cisplatin

While receiving the chemotherapy of Pemetrexed + Carboplatin or Cisplatin in accordance with the local standard therapy and the manufacturer's instruction of the product, the patient should receive antemetic drugs and intravenous fluid infusion. It is allowed to pretreat the chemotherapeutic drug according to the instructions during the treatment. Moreover, if Pemetrexed-related rashes occur, a local steroid is recommended as first-tier therapy if clinically feasible. Table 10 Recommended pretreatment medications during treatment are listed.

Table 10 Recommended Pretreatment Medications for the Chemotherapy of Pemetrexed + Carboplatin/ Cisplatin

| **Pretreatment medications** | **Dose/route of administration** | **Time** |
| --- | --- | --- |
| Folate | 350–1000 μg po | In Cycle 1, the drug is administered qd from Day 1 to at least Days 5-7, and persistently until 3 week after Pemetrexed is discontinued |
| Vitamin B12 | 1000 μg IM | In Cycle 1, the drug is administered within one week before Day 1 for once, then every 3 weeks, and persistently until 3 week after Pemetrexed is discontinued |

IM = intramuscular injection; PO = per os (oral administration).

Notes: According to local clinical practices, an antemetic drug may be used preventively.

### Precautions for nab-paclitaxel

Pretreatment: This product isn't required to be pretreated with anti allergic drug.

Note to preparation: This product is a cytotoxic anticancer drug, and it should be handled with care and is advised to be operated with gloves like other potentially toxic paclitaxel compounds. Wash thoroughly with soapy water and water immediately in case of skin contact with this product (lyophilized powder or dissolved suspension). Local exposure may cause stabbing pain, burning sensation, redness and swelling. Rinse thoroughly with flowing water in case this product is in contact with the mucosa.

Note to administration: it is advised to closely observe the injection site during intravenous dripping to guard against any possible vascular leakage. The infusion duration should be controlled within 30 minutes to reduce the infusion-related local reaction.

## Concomitant medication and concurrent therapy

Accompanied therapies include all drugs (such as prescription drugs and over-the-counter drugs) taken by the patient from 30 days before the first dose of investigational drug to the safety follow-up visit. All of those drugs should be reported to the investigator and recorded in eCRF. During the investigational treatment, investigators are allowed to provide corresponding supportive treatment according to subject’s clinical need. The specific medications can be seen in the following narration on concomitant medications. Use of the antitumor therapy unspecified in the study protocol is prohibited during treatment, however, the medical measures taken for life-threatening tumor emergencies are not included.

### Permitted Concomitant Medications

Use of local, intra-ocular, intra-articular, intranasal and inhaled corticosteroids (very little systemic absorption) is allowed. ≤10 mg/day prednisone steroid dose of adrenal replacement is permitted. It is allowed to use a short-course (less than 3 weeks) corticosteroid for the prevention of allergy (e.g., allergy to contrast agent dye) or the pretreatment of chemotherapy or the treatment of non-autoimmune disease (e.g., delayed hypersensitivity caused by contact allergens) or the treatment of adverse events caused by the investigational drug.

If necessary, the subject might be administered with sufficient supportive treatment, including whole blood and blood product infusion, antibiotics treatment, antiallergic treatment, and antidiarrheal treatment, etc.

During study, after the discussion of the case with the medical supervisor from the sponsor, the subjects can receive palliative radiotherapy (e.g., symptomatic bone metastasis or solitary brain metastasis) upon agreement by the sponsor, and radiotherapy is not allowed for target lesions.

### Disallowed Concomitant Medication

In the screening and treatment periods of the trial, subjects are forbidden to receive the following treatments:

- - Systematic antitumor chemotherapy, biotherapy or chinese patent medicines with definite antitumor indications.
  - Immunotherapy unspecified in the protocol.
  - Study drugs other than JS001, Pemetrexed, nab-paclitaxel, Cisplatin and Carboplatin.
  - Vaccination within 4 weeks prior to administration and during study medication. Examples of vaccines include but are not limited to measles, epidemic parotiditis, rubella, varicella, yellow fever, rabies, Bacille-Calmette-Guerin and typhoid (oral) vaccine.
  - Systematic use of any glucocorticoid other than treatment for the adverse events induced by immunotherapy (note: use of steroids at a physiological dose can be allowed after communication with the sponsor; the pretreatment of chemotherapeutics is allowed according to the drug instruction or clinical routine practices during treatment).
  - Generally, use of traditional Chinese medicine (TCM) is not recommended in the protocol.

## Drug compliance

The volume of the investigational product to be infused to the subject should be accurately recorded during the trial.

Calculation of compliance: after the end of the trial, the subject’s compliance should be evaluated in accordance with the dose actually infused and that should be infused, using the following formula: compliance = [dose actually infused / dose that should be infused] ×100%, range of 80%-120% will be considered as good compliance.

# Test for Biomarkers

In addition to the routine test for PD-L1 expression in tumor tissues at screening, this study is planned to test the following biomarkers and carry out a retrospective analysis:

- - Screening period: TMB and DDR of tumor tissue will be detected by whole exome sequencing (WES) of next generation sequencing (NGS)
  - Screening period and treatment period: Each subject is required to provide 8 ml whole blood for biomarker detection; 8 ml of peripheral blood is required to be collected before the first medication, and optional biomarker studies are encouraged in subsequent participation

If tumor lesion tissues may be obtained in subjects whose response is evaluated as PR and/or PD, they are encouraged to participate an optional biomarker study (Sub Study, Paired Biopsy); tumor tissue samples are collected to observe response of tumor tissues to the investigational product. For each subject, at least 12 unstained sections of primary or metastatic tumor tissues shall be provided (<12 unstained sections can be submitted after the approval of the sponsor’s medical supervisor). Prior to any relevant procedures, subjects participating the Sub Study should sign separately an independent informed consent for the Sub Study. If pseudo-progression is suspected, it is advisable to conduct biopsy of the lesion for confirmation of diagnosis if the condition allows.

Tumor tissue and/or hematology samples collected in the study and the optional biomarker study are restricted for this study and the optional biomarker study. It must be approved by the ethics committee of the research site prior to collection of tumor tissue and blood samples for PD-L1 and based on exploratory research in the research site.

# Efficacy assessments

## Tumour Evaluation

Study evaluation will be performed according to the flow chart. CT/MRI should be used for screening/baseline evaluation within 28 days prior to the first dose. Except chest, abdomen and pelvis, all the known sites of lesion should be evaluated at screening/baseline. The subsequent evaluations should included thorax, abdomen, pelvis and all the known sites of disease, and the same radiological examination method with that used at screening/baseline should be used. The tumor will be evaluated once in every 6 weeks in the first 12 months in the study period using RECIST 1.1 and iRECIST criteria. Twelve months later,it will be required to perform tumor evaluation once in every 9 weeks until progression of disease, intolerable toxicity, inability to continue to benefit from the investigational treatment as judged by investigators, withdrawal of informed consent or death, whichever comes first.

If more than one method is used at screening, the most accurate method should be used for recording the data according to RECIST v1.1, and this method should be used again in all subsequent evaluations. If , then the assessment frequency of such non-target lesions can be reduced. For example, repeated bone scan are required only when a complete response (CR) is identified for target lesions or when progressive bone lesion is suspected.

Changes in the tumor measurement and response will be evaluated by investigators in accordance with RECIST 1.1 (response evaluation criteria in solid tumors). All the imaging data for the study objectives will be submitted to BIRC for the judgment of secondary efficacy endpoints.

## ECOG PS

ECOG PS evaluation will be performed at screening and at each cycle of therapy subsequently, as to evaluate how the disease affects patient’s activity of daily living. (See annex 2: ECOG performance status scoring table).

# Safety evaluation

The drug is currently under clinical research, its overall safety profile is not yet clear. The following information is based on non-clinical and clinical study findings, as well as published data of similar molecules.

The investigators and site staffs have the duty to monitor, record and report the events that conform to the definitions of AE or SAE.

## Overall plan for safety issue management

JS001 should be administered in the presence of emergency medical facility and staff who have received training on emergency monitoring and management. During the study period, all adverse events and serious adverse events will be recorded until 30 days after the last dose of investigational drug (90 days from the collection of SAEs to the last dose) or the initiation of new anticancer treatment, whichever comes first. Following this time point, investigators will be required to report all serious adverse events related to the investigational drug (JS001/placebo) used in this study to the sponsor.

### Monitoring

Safety assessment (including laboratory test results) will be conduct according to the schedule in Attachment 1. Laboratory test results must be reviewed before each infusion of investigational drug.

General safety assessment items will include: consecutive history during the study period, physical examination, and specific laboratory examinations including serum biochemistry and blood cell count (for the list of study assessment items and schedule, please see appendix 1).

During the study period, the patients will be closely monitored for any symptom and sign of autoimmune disease and infection.

The reporting of all serious adverse events can be found in Section 9.2.2.

For the patients with ongoing investigational drug-related adverse events after the end of the study or at the termination of investigational treatment, follow-up should be continued until the following situations occur: the events return to baseline levels; the events are assessed as stable by the investigators; the patients start a new anticancer therapy; the patients lose to follow-up; the patients withdraw the informed consent form.

### Dose adjustment

It is not allowed to increase or decrease the dose of JS001/placebo. Dose delay is allowed, and the dose is allowed to be delayed for up to 8 weeks (in case of delayed administration for >8 weeks, communication with the medical supervisor of the sponsor is required, and the drug is continuously used after approval), as calculated from the last dose, otherwise the treatment will be terminated. The cumulative duration of JS001 is up to 2 years.

If the administration delay of this cycle is greater than 3 days due to adverse events, that is, JS001/placebo and chemotherapy drugs are not administered within the window period, then the administration can be resumed according to the judgment of the investigator, when the conditions for drug resumption are satisfied, and the time window of subsequent administration will be recalculated based on actual time of current administration.

If the chemotherapy drug is suspended due to chemotherapy toxicity, but JS001/placebo has been administered within the visit window period, then chemotherapy drug is allowed to be delayed for up to 7 days in this cycle, and the next cycle of administration will be recalculated based on the administration time of current chemotherapy if the chemotherapy drug is administered beyond the window period,. If the chemotherapy drug is delayed for greater than 7 days,no additional dose is advisable within this cycle (including the administration of nab-paclitaxel on Day 8 and Day 15 of the patient with squamous cell carcinoma).

Dose adjustment will be based on drug-related adverse reactions judged by investigators. According to the judgment of the investigators, if the toxicity of the subjects is related to some drugs but unrelated to other drugs, only the dose of some drugs related to toxicity can be adjusted, and only the treatment of some drugs related to toxicity can be interrupted/delayed/terminated. If it is not possible to judge whether it is only related to some drugs, it is necessary to adjust the drug use of all the investigational drugs as a whole.

#### Adjustment of JS001 treatment

Dose adjustment of JS001 are not permitted in this study. If an adverse event requiring dose interruption occurs, the investigational treatment can be interrupted. If the subjects have discontinued JS001 for more than 8 weeks due to adverse reactions and the investigator judges that the risk from continuing to give JS001 outweighs the benefit, the termination of the investigational treatment will be considered.

If the patients need to reduce the dose gradually after treatment with steroids because of adverse events, the patients can discontinue JS001 for a longer time until the completion of dose reduction for steroids or reduction to the dose of prednisone ≤10 mg/day (or equivalent dose). If the drug has been discontinued for more than 8 weeks, the investigators can consult with the medical supervisor, and then the investigational drug can be restarted based on the overall risk benefit assessment after the approval of the medical supervisor.

After the approval of the medical supervisor, the drug can be interrupted for other reasons (e.g., surgery) beyond toxicity. If the drug has been discontinued for more than 8 weeks, the investigators can consult with the medical supervisor, and then the drug can be restarted based on the overall risk benefit assessment after the approval of the medical supervisor.

The management of specific adverse events for JS001 is shown in Section 9.1.2.2. For more details, see the Investigator's Brochure for JS001.

#### Management of special adverse events for JS001

**Infusion reaction**

Clinical symptoms of transfusion reactions include fever, chills, nausea, pruritus, vasogenic edema, hypotension, headache, tracheospasm, urticaria, skin rashes, vomiting, muscle pain, somnolence or high blood pressure. Serious reactions may include acute respiratory distress syndrome (ARDS), myocardial infarction, and cardiogenic shock. Therefore, the subjects in this study must be closely observed for relevant clinical symptoms. See Attachment 6 for the treatment of allergic reactions.

If there are severe reactions, ECG monitoring and rescue medications (including, but not limited to, Adrenaline and glucocorticoids, antihistamines, bronchodilator and oxygen) are needed for emergency treatment. When the infusion reactions of CTCAE5.0 grade 2 or above or suspected events occur, the infusion of JS001 must be stopped immediately. The chemotherapy will be restarted one hour after the end of administration of JS001 while the close monitoring of each vital sign has been completed and confirmed to have no abnormality. The patients will be informed of possible delayed symptoms post transfusion and are required to contact the doctor after the symptoms appear.

If severe allergic reactions occur, the patients must be treated according to the local best routine care.

If the infusion reactions of CTCAE5.0 Grade 3 or above occur again, the patients must discontinue the drug immediately and permanently and withdraw from the study.

Treatment adjustment guideline for infusion reactions is presented in XXX Table 11.

Table 11 Treatment adjustment guideline for infusion reactions

| **CTCAE5.0 grade** | **Adjusting measures** |
| --- | --- |
| **Grade 1 (Mild)** | The interruption of infusion or clinical interventions are not recommended for transient mild reactions. Slow down the dripping speed by 50% and observe for any worsening symptoms closely. Clinical intervention, as required. |
| **Grade 2 (moderate)** | Discontinue JS001 treatment and give immediate systemic treatment (e.g. antihistamines, NSAID, anesthetics, intravenous fluid infusion); when the infusion reaction is relieved to grade 0-1, the drug is re-introduced and the dripping speed is reduced by 50%. Observe any worsening symptoms closely. Take appropriate interventions according to local routine care. |
| **Grade 3 - severe** | Stop the infusion immediately and remove the infusion tube. The investigators discussed whether to restart the drug with the study sponsor medical supervisor based on the patients' actual condition.  If the drug is restarted, the infusion lasts for at least 2 hours, the relevant preventive medications (such as Diphenhydramine and NSAID) are needed, and the relevant clinical symptoms of infusion reactions are needed to be closely observed in subsequent treatment.  Take appropriate interventions according to local routine care. |
| **Grade 4 - life-threatening and requiring urgent clinical intervention.** | Patients with Grade 4 infusion reactions must stop the drug immediately and permanently and withdraw from the study. Take appropriate interventions according to local routine care. |

**Severe allergic reaction**

When the severe allergic reactions occur, the subjects must be given emergency treatment immediately according to the local routine care. The investigators must be informed of such symptoms immediately when they occur.

Systemic allergic reactions (usually defined as reactions occurred within minutes after the drug administration: respiratory distress, laryngeal edema, and/or severe tracheospasm, with concomitant hemorrhagic shock without respiratory systems; The skin reactions are usually characterized by pruritus and urticaria with or without concomitant edema; the reactions of digestive tract system are characterized by nausea, vomiting, abdominal distension and diarrhea) occur, and the patients must immediately stop the drug permanently and withdraw from the study.

The patients must be given Adrenaline, Dexamethasone and ECG monitoring immediately.

**Immune-related adverse events (irAEs)**

JS001 may be related to the following potentially important immune-related adverse events: immune-related hepatitis, pneumonitis, colitis, pancreatitis and endocrine system diseases (hypothyroidism, hyperthyroidism, adrenal cortex insufficiency, and diabetes mellitus). Please refer to the investigator's brochure for the detailed introduction of JS001 safety risk management.

Moreover, immune-related adverse reactions include all kinds of the following events of clinical significance: exfoliative dermatitis, uveitis, arthritis, myocarditis, pancreatitis, hemolytic anemia, possibility of partial seizures in patients with hemispheric inflammation in brain, adrenal insufficiency, myasthenia gravis, optic neuritis and rhabdomyolysis.

It is necessary to closely observe the function of the related systems for the suspected immune associated adverse reactions and ensure that adequate assessment is made to determine the cause and to exclude other causes. In general, JS001 should be discontinued or permanently stopped depending on the severity of the event and/or symptomatic treatment such as glucocorticoids is administrated. When the patients are treated with Prednisone 1-2mg/kg/day or glucocorticoids of the same efficacy, a gradual dose reduction (at least 4 weeks) will be initiated after the events are relieved to grade 0-1. The drug reuse can be considered after completing the dose reduction of steroids or the dose reduction of Prednisone to ≤10mg/day (or an equivalent dose). Please refer to the investigator's brochure for the detailed introduction of risk management. At this point, if irAEs are still at grade 0-1, it will be treated with JS001 again. When an irAE of grade 3 or above recurs (except for endocrine system diseases), the patient needs to discontinue the drug immediately and permanently and withdraw from the study.

Refer to。Table 12 in detail for adjustments on JS001 therapeutic regimen caused by immune related adverse reactions If the investigator's manual is updated during the study, the js001 dosing adjustment protocol of JS001 qill be subject to the guidelines for management of immune related adverse reactions recorded in the latest version of the investigator's manual.

Table 12 - Adjustment plan of JS001 treatment

| **Immune-related adverse reactions** | **Severity**  **(NCI CTC v5.0)** | **Adjustment of regimen** |
| --- | --- | --- |
| Pneumonia | Grade 2 | Dose interruption, until recovery to grade 0-1 |
|  | Grade 3-4 or recurrent grade 2 | Permanently discontinued |
| Diarrhea and Colitis | Grades 2-3 | Dose interruption, until recovery to grade 0-1 |
|  | Grade 4 | Permanently discontinued |
| Hepatitis | Grade 2, AST or ALT at 3-5 x ULN or bilirubin total at 1.5-3 x ULN | Dose interruption, until recovery to grade 0-1 |
|  | Grade 3-4, AST or ALT > 5 x ULN, or total bilirubin > 3 x ULN | Permanently discontinued |
| Nephritis | Grade 2-3 elevated blood creatinine | Dose interruption, until recovery to grade 0-1 |
|  | Grade 4 elevated blood creatinine | Permanently discontinued |
| ENDOCRINE DISORDERS | Grade 2-3 symptomatic thyroid function decreased,  Grade 2-3 hyperthyroidism,  Grade 2-3 hypophysitis,  Grade 2 adrenal insufficiency  Grade 3 hyperglycaemia or type I diabetes mellitus | Dose interruption, until recovery to grade 0-1^c^ |
|  | Grade 4 thyroid function decreased,  Grade 4 hyperthyroidism,  Grade 4 hypophysitis,  Grade 3-4 adrenal insufficiency,  Grade 4 hyperglycaemia or type I Diabetes mellitus | Permanently discontinued |
| Cutaneous adverse reaction | Grade 3 rash | Dose interruption, until recovery to grade 0-1 |
|  | Grade 4 skin rash, Stevens-Johnson syndrome (SJS) or toxic epidermal necrolysis (TEN) | Permanently discontinued |
| Thrombopenia | Grade 3 | Dose interruption, until recovery to grade 0-1 |
|  | Grade 4^b^ | Permanently discontinued |
| Others | Grade 3-4 increased blood amylase and lipase  Grade 2-3 pancreatitis  Grade 2 myocarditis^a^  Grade 2-3 other immune-associated adverse events of first occurrence | Dose interruption, until recovery to grade 0-1 |
|  | Grade 4 or other grade recurrent pancreatitis  Grade 3-4 myocarditis  Grade 3-4 encephalitis  Grade 4 other immune-associated adverse events of first occurrence | Permanently discontinued |
| Recurrent or persistent adverse reaction | Recurrent grade 3-4 (except endocrine disease)  Grade 2-3 adverse reaction not improved to grade 0-1 within 8 weeks after the last dose (except endocrine disease)  Corticosteroid unable to be reduced to ≤10mg/day prednisone equivalent dose within 8 weeks after the last dose | Permanently discontinued |

1. The safety is still unclear whether this product can be resumed after myocarditis is improved to grade 0-1 after treatment.
2. For Grade 4 thrombocytopenia, it is necessary to decide whether permanent drug withdrawal according to the a associated symptoms / signs and clinical judgement of the investigator ; should be considered；
3. The re-medication can be considered if the hypophysitis, hypoadrenocorticism, hypothyroidism and hyperglycemia / Type I diabetes mellitus are fully controlled and only physiological hormone replacement therapy is needed.

#### Dose adjustment of chemotherapeutics

The dose adjustment of chemotherapy drugs must be subjec to the peak toxicity occurring in a cycle. The maximum administration interval of chemotherapy drug isn't to be greater than 42 days (to be calculated based on the last administration time).

Ifthe dose of any drug is reduced due to the toxicity, no further dose escalation is allowed. If the subject suffers from some toxic reactions, and the recommended dose adjustment protocol is contradictory, the most conservative dose adjustment (i.e. the dose reduction suitable for the severests toxicity) should be adopted.

Different dose levels of chemotherapeutic drugs are listed in Table 13, and the chemotherapy drug will be discontinued if the dose still needs to be reduced after two dose levels are reduced.

Relevant regulations for recommended dose adjustment are shown in the following sections. If the investigators consider the benefit/risk ratio of the subjects during the clinical operation, and believe that the operations listed below cannot be carried out or that the situation not listed occurs, then the clinical operation can be done according to the drug instruction and the clinical diagnosis and treatment specifications.

Table 13 - Dose adjustment of chemotherapeutics

|  | **Initial dosage** | **reduce one dose level** | **Reduce two dose levels** |
| --- | --- | --- | --- |
| Pemetrexed | 500 mg/m^2^ | 375 mg/m^2^ | 250 mg/m^2^ |
| Cisplatin | 75 mg/m^2^ | 56 mg/m^2^ | 38 mg/m^2^ |
| Carboplatina | AUC 5  Maximum dose: 750mg; | AUC 3.75  Maximum dose: 562.5mg; | AUC 2.5  Maximum dose: 375mg; |
| Nab-paclitaxela | 100 mg/m^2^ | 75 mg/m^2^ | 50 mg/m^2^ |

##### Dose adjustment and management of specific adverse events for Pemetrexed and Cisplatin

At the beginning of the subsequent treatment cycle, the dose adjustment should be based on the minimum hematological count in the previous treatment cycle or the maximum non-blood system toxicity. If the relevant examination does not meet the requirements, the treatment may be postponed to allow the sufficient recovery time, and the treatment can be delayed for up to 42 days.

While being combined with Carboplatin or during maintenance treatment, refer to Table 14 - Table 16。

Table 14 Dose adjustment of Pemetrexed and Cisplatin for hematological toxicity

| **Toxicity^a^** | **Dose of Pemetrexed and Cisplatin** |
| --- | --- |
| ANC <500/mm^3^ and platelet ≥50，000/mm^3^ | reduce one dose level |
| Platelet <50，000/mm^3^, irrespective of ANC | Reduce two dose levels |

^a^ Nadir in previous courses of therapy.

If the patient suffers from non hematologic toxicity (excluding neurotoxicity) ≥ 3 degree , the therapy with this product should be discontinued until it the level prior to treatment is returned or slightly lower than the level prior to treatment. The treatment should be repeated according to Table 15.

Table 15 Dose adjustment of Pemetrexed and Cisplatin for non-haematological toxicity

| **Toxicity ^a,b^** | **Pemetrexed dose** | **Dose of cisplatin** |
| --- | --- | --- |
| Any toxicity of grade 3^c^ or 4 except mucositis and alopecia. | reduce one dose level | reduce one dose level |
| Any diarrhoea that requires hospitalization (regardless of event grading) or diarrhea of grade 3 or 4. | reduce one dose level | reduce one dose level |
| Grade 3 or 4 mucositis | Reduue two dose levels | Initial dosage |

1. CTCAE grading
2. Excluding neurotoxicity.
3. Excluding grade 3 transaminases increase

The dose adjustment of the investigational product and cisplatin for neurotoxicity is presented in Table 16. If grade 3 or 4 neurotoxicity is observed, the treatment should be terminated.

Table 16 Dose Adjustment of Pemetrexed and Cisplatin Caused by Neurotoxicity

| **CTCAE grading** | **Pemetrexed dose** | **Dose of cisplatin** |
| --- | --- | --- |
| 0-1 | Initial dosage | Initial dosage |
| 2 | Initial dosage | Reduce two dose levels |

If the patients have any haematological or non-haematological toxicity of grade 3 or 4 (excluding grade 3 transaminases increase) after the reduction of two dose levels, Pemetrexed treatment should be discontinued immediately; or if the neurotoxicity of grade 3 or 4 is observed, Pemetrexed treatment should be discontinued immediately.

For more details on the dose adjustment of Pemetrexed, please refer to the package insert.

##### Dose adjustment and management of specific adverse events for nab-paclitaxel

For patients with non-small cell lung cancer, dose of nab-paclitaxel can be resumed only when the neutrophil is at least 1500/mm^3^ and platelet count is at least 100000/mm^3^.

The treatment should be interrupted when serious neutropenia or thrombocytopenia occurs during the treatment of nab-paclitaxel, until the above parameters are recovered (neutrophil count ≥ 1,500/mm^3^ or platelet count ≥ 100,000/mm^3^, on Day 1 of each cycle; or neutrophil count ≥500/mm^3^ or platelet count ≥50,000/mm^3^, on Day 8 or 15 of each cycle). In the subsequent treatment, the dose adjustment of nab-paclitaxel should be performed according to XXX Table 17.

In case that grade 3-4 neurotoxicity occurs, the dose should be reduced in accordance with XXX when it is recovered to grade 1 or completely recovered Table 17.

Table 17 Dose adjustment of nab-paclitaxel for hematological and neurological adverse reactions

| **Adverse Drug Reaction** | **Number of Occurrences** | **Weekly dose of nab-paclitaxel (mg/m^2^)** |
| --- | --- | --- |
| Febrile neutropenia (absolute neutrophil count <500/mm^3^ with fever > 38°C) or  Delay of the next cycle of therapy by more than 7 days due to absolute neutrophil count <1500/mm^3^, or  Absolute neutrophil count <500/mm^3^ for more than 7 days | First | reduce one dose level |
|  | Second | Reduce two dose levels |
|  | Third | Treatment discontinued |
| Platelet count <50,000/mm^3^ | First | reduce one dose level |
|  | Second | Treatment discontinued |
| Serious sensory neuropathy - grade 3 or 4 | First | reduce one dose level |
|  | Second | Reduce two dose levels |
|  | Third | Treatment discontinued |

No dose adjustment is required for patients with mild hepatic impairment. Treatment with nab-paclitaxel may increase the risk of its known toxicity in patients with moderate and severe hepatic impairment. Nab-paclitaxel cannot be used in case of AST >10 x ULN or bilirubin >5 x ULN. The recommendation on the dose adjustment for the first cycle is presented in XXX Table 18.

Table 18 Recommendation on the starting dose in patients with hepatic impairment

|  | **Glutamic-oxaloacetic transferase** |  | **Bilirubin** | **Dose of nab-paclitaxel^a^** |
| --- | --- | --- | --- | --- |
| Mild | <10XULN | and; | >ULN≤1.25 X ULN | 100mg/m^2^ |
| Moderate | <10XULN |  | 1.26 to 2 X ULN | 75mg/m^2^ |
| Severe | <10XULN |  | 2.01 to 5 X ULN | 50mg/m^2b^ |
|  | >10XULN | OR | >5 X ULN | Not permitted |

- 1. This dose is recommended for the treatment-naïve patients, and should be adjusted in accordance with individual tolerability during subsequent treatment.
  2. In case it can be tolerated, the dose can be increased to 75 mg/m^2^ in subsequent cycles of therapy.

##### Dose adjustment and management of specific adverse events for carboplatin

**Hematological Toxicity**

At the start of each cycle, neutrophil count must be ≥ 1500/mm^3^and platelet count must be ≥100,000/mm^3^. If the requirement is not met, the treatment should be delayed, so as to provide the sufficient time for recovery, and the treatment can be delayed for up to 42 days. Growth factor can be used in accordance with the guidelines of American Society of Clinical Oncology (ASCO) and National Comprehensive Cancer Network (NCCN). After recovery, the dose will be adjusted based on the minimum platelet and neutrophil counts in the previous cycle when the subsequent cycle starts (see Table 19).

Table 19 Dose adjustment of carboplatin for hematological toxicity

| **Toxicity^a^** | **Dose of carboplatin** |
| --- | --- |
| ANC <500/mm^3^ and platelet ≥50，000/mm^3^ | reduce one dose level |
| Platelet <50，000/mm^3^, irrespective of ANC | reduce one dose level |
| Platelet <50，000/mm^3^ with ≥grade 2 hemorrhage, irrespective of ANC | Reduce two dose levels |
| ANC<1000/mm^3^ and fever with temperature ≥38.5°C | reduce one dose level |

^a^ Nadir in previous courses of therapy.

All the dose adjustments for the first attack of febrile neutropenia or thrombocytopenia (platelet count <25，000 or <50，000 with hemorrhage or requiring blood transfusion) are permanent. If febrile neutropenia or thrombocytopenia occurs for the second time and reduction of the dose is needed, the dose of carboplatin will be reduced according to doctor’s judgment and local practice of standard treatment. When febrile neutropenia or grade 4 neutropenia occurs, colony stimulating factor (e.g., granulocyte colony-stimulating factor) can be used to substitute dose reduction in accordance with the local practice of standard treatment and ASCO guideline. Chemotherapy should be discontinued immediately for the patients who need the dose reduction for the 3rd time.

If dose adjustment is required simultaneously for ANC and platelet, the patient will receive the lower dose in accordance with the grade of the most serious adverse reaction.

The treatment can be delayed for up to three weeks, until neutrophil count ≥ 1500/mm^3^ and platelet count ≥ 100，000/mm^3^on the 1st day. However, if the counts are not recovered within three weeks, the dose of chemotherapy should be reduced or interrupted according to the doctor’s judgment and the local practice of standard treatment, until the neutrophil count is recovered, otherwise the treatment will be discontinued. If the chemotherapy is interrupted for more than 42 days, all the chemotherapies should be discontinued.

Investigators should pay attention to and be on the alert for the early and obvious bone marrow suppression, infection or signs of febrile neutropenia, thereby rapidly and appropriately managing these complications. The patients must be reminded of the above possible signs, and encouraged to go to see a doctor as early as possible.

If the chemotherapy must be interrupted for hematological toxicity, the complete blood cell count (including WBC differential count) should be performed once per week, until the count reaches the lower limit specified for the treatment. The treatment plan shall be completed in accordance with the conventional order afterwards.

Dose reduction is not needed for anemia. The patients should be supported in accordance with the guideline at the institution where the attending doctor is located.

**Non-hematological toxicity**

If grade 3 or 4 gastrointestinal toxicity occurs, the treatment should be delayed until the testing value returns to ≤baseline value. The dose will be reduced based on the gastrointestinal toxicity of the dose for the last course of therapy, when subsequent course of therapy starts. Table 20 Dose adjustment for non-hematological toxicity is provided.

Table 20 Dose adjustment of carboplatin based on the non-hematological toxicity in the previous ccycle.

| **toxicity** | | **Dose of carboplatin** |
| --- | --- | --- |
| Diarrhea | Grade 3 or 4^a^ | Initial dosage |
| Stomatitis | Grade 3 or 4 | reduce one dose level |
| Nausea/vomiting | Grade 3 or 4 | reduce one dose level |
| Neurotoxicity (motor or sensory) | Grade 2 | Initial dosage |
|  | Grade 3 or 4 | reduce one dose level |
| Transaminases increased | Grade 3 | reduce one dose level |
|  | Grade 4 | Treatment withdrawal |
| Others | Grade 3 or 4 | reduce one dose level |

^a^ Or any grade of diarrhoea requiring hospitalization.

When nausea and/or vomiting occur, appropriate antiemetics should be used for control. If grade 3 or 4 nausea/vomiting occurs despite of the use of antiemetics, the dose should be reduced by 25% in the next cycle.

If oral mucositis occurs on Day 1 in any course of therapy, the dose should be interrupted, until it is resolved. If oral mucositis/stomatitis is not resolved within three weeks, the chemotherapy will be terminated. If grade 3 acute oral mucositis occurs at any time, 75% dose should be given after it is completely resolved. This is a permanent reduction of dose.

## Safety parameters and definitions

The items of safety evaluation include: monitoring and recording of adverse events, including serious adverse events and adverse events of special interest, laboratory safety evaluations specified in the study protocol, measurement of vital signs specified in the study protocol, and other testing parameters critical for safety evaluation specified in the study protocol.

### Definition of Adverse Events (AEs)

Adverse events (AEs) are all the undesirable medical events occurring after the administration of one pharmaceutical therapy during a clinical study, regardless of the casual association between the events and the treatment.

The names of adverse events should be recorded as the names of diagnoses or diseases in the CRF. If the name of adverse event can not be determined or the investigator considers the diagnostic name or disease name can not be used as the name of adverse event, the clinical condition or symptom will be recorded on CRF as the name of adverse event.

### Serious adverse events (SAEs) (reported to the sponsor immediately)

A serious adverse event is defined as an undesirable medical event meeting one or more of the following criteria:

1. Leading to death;

Note: The death due to progressive disease is the expected endpoint of efficacy, it is not necessary to be reported as SAE, but it should be recorded in the death description page of eCRF, and the main cause of death should be noted in the report.

Unexplained deaths during the follow-up period of SAE at the study treatment phase or after the administration in last study treatment should always be reported as SAE and recorded on the death description page in ECRF. The investigators should follow up as much as possible to know and evaluate the cause of death.

1. Life threatening (i.e., the AE, in the view of the investigator, places the patient at an immediate risk of death)
2. Requiring hospitalization or prolonged hospital stay; note: the following hospitalization does not belong to SAE in this study:

- Seeing a doctor in the emergency room or other departments of the hospital for <24 hours, the subject is not admitted (unless important medical event or life-threatening event).
- Surgery on an elective date, scheduled operation prior to signature of the informed consent form.
- Admitted for originally scheduled medical procedure/surgical operation according to the study protocol.
- Routine medical examination requiring hospitalization for evaluation of baseline/tendency of health status (e.g., routine colonoscopy).
- Admission to the department of medicine/surgery that has been scheduled not for rescue or treatment of disease prior to entry in the study; however, these conditions need to be well documented.
- Hospitalization for other living conditions that is unrelated with health status and does not need pharmacological/surgical intervention (e.g., no housing, finance constraints, temporary absence of caregivers, family environment and management reasons).
- Hospitalization to receive anticancer therapy without any other SAE.

1. Leading to permanent or significant disability/ loss of ability;
2. Congenital anomaly/birth defect;
3. Other important medical events.

In other cases, expedited reporting should be decided after medical and scientific judgment if the important medical event may not threaten life immediately or lead to death or hospitalization but may endanger patients or need interventional measures to prevent any one of the other consequences listed in the above definition.

### Adverse events of special interest (AESI) (reported to the sponsor immediately)

The investigator will be required to report non-serious AESI to the sponsor immediately (i.e., within 24 hours after awareness of the event) (without reporting to the regulatory authority). AESI of JS001, including

- Suspected immune-related myocarditis: elevated myocardial enzymes, with change of ECG or clinical symptom
- Abnormal liver function that meets Hy’s Law criteria:

Increased ALT or AST (>3 * ULN) complicated with increased total bilirubin (>2 * ULN), or clinical jaundice, or increased bilirubin excluding obstructive jaundice or other causes.

## Causality assessment

The investigator should judge whether the adverse event is related to the investigational drug based on their understanding of the subjects, the pre- and post-event conditions and evaluation of all potential reasons. The following points should be taken into consideration in the judgment:

- Temporal relationship between the occurrence of the event and the start of the investigational drug;
- The course of events, in particular, the impact of dose reduction, discontinuation of investigational drug, or rechallenge of investigational drug (if applicable) should be considered;
- Known events are related to investigational drug or similar therapy;
- Known events are related to study diseases;
- Subjects have risk factors or receive concomitant medication that increase the probability of occurrence of events;
- There are non-therapeutic related factors known to be related with the occurrence of the event.

The relationship between the adverse event and the investigational drug will be determined as 2 results by the investigator based on its clinical judgment and the following definitions: related/ unrelated.

## Assessment of severity

The severity of AE will be evaluated with reference to CTCAE version 5.0. If the adverse events occured do not fall within the scope of this criteria, the Table 21 below will be used to assess the severity:

Table 21 Severity rating scale for adverse events not specifically listed in NCI CTCAE5.0

| **Grade** | **Severity** |
| --- | --- |
| 1 | Mild; asymptomatic or mild symptoms; clinical or diagnostic observations only; or no treatment required |
| 2 | Moderate; minimal, local or non-invasive intervention indicated; limited age appropriate instrumental ADL (Activities of Daily Living) ^a^ |
| 3 | Severe or medically significant, but not life‑threatening immediately; requiring hospitalization or prolonging hospital stay; disability; or limited self‑care of daily living ^b^, |
| 4 | Life-threatening outcomes, or requiring emergent treatment |
| 5 | Deaths caused by AEs |
| NCI CTCAE= (US)National Cancer Institute Common Terminology Criteria for Adverse Events.  Note: Based on the latest version of NCI CTCAE (v5.0), full-text URL: http://ctep.cancer.gov/protocolDevelopment/electronic_applications/ctc.htm   1. Instrumental activities of daily living refer to cooking, shopping for groceries or clothes, using telephones, and managing money etc. 2. Examples of self-care activities in daily life include bathing, dressing, eating, going to the toilet, and taking medicine, which are activities that ambulatory patients can engage in. | |

## Recording and reporting of adverse events/serious adverse events

At every follow-up visit as scheduled during the trial, all adverse events that have occurred since the previous visit must be recorded. Progressive disease (including fatal progressive disease) expected to occur in the study population should be measured as an endpoint of efficacy and shall not be reported in terms of AEs. Moreover, the symptoms, signs or clinical sequelae definitely caused by disease progression are also not required to be reported in terms of AEs. If it is uncertain whether an event is due to disease progression, the event should be reported as an adverse event.

The definitions of the reporting interval for adverse events/serious adverse events are presented in Table 22. Investigators must determine the severity of each adverse event and the relationship between each adverse event and investigational drug (see Sections 9.3 and 9.4).

Table 22 Definitions of the reporting interval for adverse events/serious adverse events

| **Time period** | **Reporting requirements** |
| --- | --- |
| From signing the ICF until receiving the first dose of investigational drug | Only serious adverse events are reported. |
| From the initiation of first dose of investigational drug through 30 days after the last dose of investigational drug or initiation of a new anti-tumor therapy (whichever occurs first) | All AEs should be recorded (including non-serious adverse events of special interest specified in the protocol) and reported. |
| 30 to 90 days after the last dose of investigational drug or the initiation of a new anti-tumor therapy (whichever occurs first) | All SAEs (including non-serious adverse events specially interest specified in the protocol) should be reported. |
| At any time after the ending of investigational treatment | The investigator should report any SAEs judged as related to JS001. |

### Recording and reporting of adverse events

All AEs that occur during the clinical study, regardless of whether they are suspected of being related to investigational drugs, should be treated with the following measures:

1. Investigators should immediately take appropriate protective measures for the subjects to ensure their safety.
2. If the investigational treatment is terminated, investigators should also periodically follow up and perform corresponding diagnosis and treatment for the subjects and fill in the eCRF with the date of termination of the investigational treatment (the date when the investigational drug is discontinued) and the reason for termination.
3. The investigators should follow up all AEs until any of the following:

- AEs are relieved or improved to baseline levels;
- Subject death;
- Subjects are lost to follow-up;
- Subjects start a new anti-cancer treatment;
- End of study.

Any adverse event should be recorded in detail on the case report form and reported in the clinical study report.

### Reporting of Serious Adverse Events

The care physician should immediately take appropriate protective measures for all serious adverse events during the treatment or within 90 days after the last treatment or until the start of new anti-tumor treatment (whichever occurs first). These adverse events will be followed up until remission or stability above the acceptable level by investigators.

Standard SAE report form provided by the sponsor should be completed according to the completion guide within 24 hours after being informed of SAE and sent to the email address and or fax number provided by the sponsor. Other reporting requirements can be reported according to the requirements of the organization office of the center.

After the end of follow-up period, investigators are not responsible for active collection of new adverse events. However, after the trial period including the follow-up period, if investigators are aware of any SAE that is considered to be related with the investigational drug (JS001/placebo), they should report to Junshi Safety Department within 24 hours after awareness.

Death

All deaths that occur during the study, or within the protocol-defined safety follow up period after the administration of the last dose of investigational drug must be reported as follows:

- Death definitely caused by progressive disease is defined as an efficacy endpoint and does not need to be reported as SAE. The death should be recorded on the death description page in eCRF.
- If the death is not caused by the progress of studied disease (or unknown cause) , the AE causing the death must be reported as SAE. It should also be documented in the Statement of Death page in the eCRF. The report should include a description of possible disease progression and complications (if applicable) and indicate the primary and secondary causes of death.
- A death without any known cause is reported as SAE. It should also be documented in the Statement of Death page in the eCRF. The investigator should follow up as much as possible to know and evaluate the cause of death. The autopsy making for assessing the cause of death, and a copy of the autopsy results should be sent to the sponsor within the normal time limit if an autopsy is performed.

Deaths occurring after the protocol-defined safety follow-up period after the administration of the last dose of investigational drug should be documented in the Statement of Death page. If the death occurs as a result of an event that starts after the defined safety follow-up period and the event is considered to be caused by the delayed toxicity of investigational drug, then it should also be reported as an SAE to the sponsor.

### Reporting and follow-up of pregnancy

#### Pregnancy of female subjects

The female patients of childbearing potential will be informed to notify investigators immediately if they are pregnant during the study or within 90 days after the last dose of investigational drug. Investigators should fill and submit the pregnancy report to the sponsor immediately (i.e., within 24 hours after awareness of pregnancy). Investigators should discontinue the investigational drug and discuss with the subjects about the risk of pregnancy and possible effects on the fetus. The investigators must continue monitoring the subjects until the end of the pregnancy. Any SAE associated with pregnancy (e.g. fetal event, maternal event during or after pregnancy, or congenital malformation/birth defect of child) should be reported in AE page of eCRF.

#### Pregnancy in the Female Partner of a Male Subject

When the investigator is informed that the spouse of male subject is pregnant during the study period or within 90 days after last administration of the study drug, the investigator must be immediately informed, and the investigator shall immediately (i.e. within 24 hours after knowing the pregnancy) complete the pregnancy report form and submit it to the sponsor, the investigator will provide information about the pregnancy risk and potential impact on the fetus, and the pregnancy conditions must be followed up to the maximum extent.

#### Misscarriage

All spontaneous abortion should be classified as SAE (as spontaneous abortion is an event of medical significance), recorded on AE page of eCRF and reported to the sponsor immediately (i.e. within 24 hours after being informed of the event).

#### Congenital anomaly/birth defect

Any congenital malformation / birth defect of the infant delivered by female patient or female spouse of male subject who have been exposed to the study drug should be classified as SAE, recorded on AE page of eCRF and reported to the sponsor immediately (i.e. within 24 hours after being informed of the event).

# Data management

## Data entry

In this study, subjects' data shall be input into the electronic case report form (eCRF), and transferred to the data system confirmed by the sponsor, to incorporate with the data of other sources.

The management of clinical data shall be carried out in accordance with applicable CDISC criteria and the data clearance procedure to ensure integrity of the data; for example, incorrect and inconsistent data are removed. Adverse events will be encoded by Medical Dictionary for Regulatory Activities (MedDRA), and the concomitant medication name will be encoded by the World Health Drug Dictionary (WHO Drug). eCRF shall be kept by the sponsor; a copy shall be mailed to the investigator as Investigator's Copy.

The staff at study site are responsible for filling in eCRF. For all subjects signing ICF, the investigator or an authorized person shall record carefully and detailedly any items in eCRF, without any blank or missed items (an item without any record shall be filled with UK/NA/ND as is the case); all data in eCRF must be checked with the patient's original data to ensure that they are correct.

The investigator must attach the original laboratory sheet or a copy to the subject's investigational medical records; the investigator shall check any abnormal laboratory test or examination data, and explain whether they are clinically significant; the investigator shall fill in strict accordance with the instruction for eCRF filling.

Authorized representatives (supervisors, auditors, etc.) from Junshi must be allowed to visit all study center sites on a regular basis to evaluate the quality of the data and whether the study is complete and reliable. They will review the study records on-site, compare these records directly with the original documents, discuss the implementation of the study with the investigators, and confirm whether the study facility still meets the requirements.

Content: whether the protocol is followed; whether all CRFs are filled correctly and completely, and whether they are consistent with the original documents such as the study medical records and laboratory test reports, and whether the data is wrong or missing. Monitors needed to check the content in eCRF with the original document one-by-one, as to ensure the consistency between the data in eCRF and original data, this process was also called source data verification (SDV).

## Database Lock

The data may be locked when the following conditions are met:

1. All data have been input the database;
2. All questions have been resolved;
3. Investigators have finished the signature;
4. The analysis population have been defined and judged.

After database locked, the database may be re-unlocked when the project team considers unlock necessary after assessment. The process of unlocking the database should include notifying the project team to clearly define which data errors will be changed, the reason for the change, and the date of the change, and be signed by principal investigator, data managers, and statistical analysts, etc. The relocking of the database should follow the same notification/approval process as the database first lock.

## Data archiving

After completion of the study, subject’s eCRF in a format of PDF needs to be generated in EDC system, and saved in one CD-ROM that will be submitted to the sponsor and each institution for archival, in order for audit and / or inspection. The storage and management of the study data shall be carried out in accordance with the requirements of the GCP. The necessary documents for the clinical trial shall be kept until 2 years after the approval of the investigational drug for marketing or 5 years after the termination of the clinical trial.

# Statistical analyses

The statistical analysis plan shall be formulated after finalization of the protocol, and finally determined prior to locking of the database. Statistical Analysis Plan: The contents of all scheduled statistical analyses would be specified and described in details, based on the main characteristics of the Protocol. All statistical analyses are calculated with SAS9.4 and above versions of statistical analysis software by the clinical data analysis department.

In this study, all variables obtained at the various observation timepoints are described statistically by random treatment group, unless they are not described statistically at the specific timepoints as specified in the Protocol. Overall, continuous variables (such as age) will be described statistically with N, mean, median, standard deviation, minimum, and maximum, and categorical variables will be described statistically with frequency and percentage of each category. The final analysis of the study would be based on the data collected throughout the study. The statistical method would be described detailedly in the Statistic Analysis Plan.

## Determination of sample size

The sample size will be calculated based on the primary efficacy endpoint and with PFS evaluated by investigators using RECIST 1.1. The patients will berandomized by 2:1 to receive treatment, about 450 patients need to be enrolled (about 300 in JS001 group and 150 in placebo group), expecting to conduct the primary efficacy analysis when 356 cases of PFS events are observed at about 27 months after the randomization of the first subject, thereby there is 85% power to detect the improvement of PFS in the treatment of advanced or recurrent non-small cell lung cancer by JS001 combined with pemetrexed + platinum-based drugs versus placebo combined with pemetrexed + platinum-based drugs at the one-sided significance level of 0.025 (corresponding hazard ratio (HR)=0.7). The interim analysis of efficacy is planned to be conducted when 214 cases of PFS events (60% information ratio) are observed. The sample size will be calculated using EAST 6.5 based on the following assumptions:

- PFS appears exponential distribution.
- Median PFS is 6 months for the chemotherapy group.
- α-consumption function of Pocock type (approximation using Lan-DeMets method) will be used for interim analysis and primary analysis so as to control the overall type I error rate.
- 450 subjects will be enrolled within 15 months.
- The drop-out rate is 5% in each treatment group in the observational period of PFS event in the first 12 months.

## Data analysis sets

The intent-to-treat (ITT) analysis set includes all the randomized subjects during the main study phase and will be used as the main analysis set for efficacy analysis.

Per protocol set (PPS) includes all the ITT population without any major deviation influencing the efficacy analysis from the protocol and with the valid baseline. Prior to unblinding in the study, the subjects included in PPS will be determined in accordance with the actual deviation from the protocol. PPS population will be used for the sensitivity analysis of primary efficacy endpoint and partial secondary endpoints.

Safety analysis set includes all subjects who have received at least one dose of investigational drug, and will be used for safety analysis.

## Analytical method

Unless otherwise specified, all efficacy endpoints will be analyzed with ITT set as main analysis set and analyzed according to the treatment plan assigned to the subject at the time of randomization. The analysis on the duration of response (DOR) and time to first response (TTR) will be based on the subject who has achieved response (including CR and PR). PPS will be used as a sensitivity analysis set.

The safety analysis will be performed based on the safety analysis data set and grouped according to the therapeutic regimen actually accepted by the subject.

### Demographic characteristics, baseline disease characteristics, and trial progress

The demographic variables (e. g, age, gender, height, weight, etc.) and baseline disease characteristics (e.g., ECOG status) will be based on the intention-to-treat population will be descriptively summarized based on the treatment group.

The completion of the trial, reason for termination of trial medication and early withdrawal from the trial, as well as major protocol violations (e. g, violation of the inclusion criteria) of intention-to-treat population will be summarized based on the treatment group.

### Effectiveness

**Primary efficacy endpoints:**

The primary efficacy endpoint in this study is progression free survival (PFS) assessed by the investigator as per RECIST 1.1. PFS is defined as the time from the randomization to the first record of progressive disease, or to death for any cause (whichever comes first). Subjects with no progression of disease or death will be censored on the day of the last valid tumor evaluation. Subjects who have not received any tumor evaluation during the study and are not dead will be censored one day after randomization. Subjects who do not report any progression of disease nor start any anticancer therapy unspecified in the protocol will be censored on the day of the last evaluable tumor evaluation prior to the start of subsequent anticancer therapy.

Stratified log rank test will be used for the analysis of the primary efficacy endpoint PFS. The stratification factors include smoking status (often smoking vs no smoking or infrequent smoking), pathological type (squamous cell carcinoma vs non-squamous cell carcinoma) and PD-L1 status (TC≥1% vs TC＜1%, note: the patients not evaluable for PD-L1 will be included into TC＜1% group), the significance level is one-sided 0.025. Kaplan-Meier (KM) method will be used to estimate the median PFS in each treatment group, the 95% confidential interval of median PFS will be estimated through Brookmeyer-Crowley method using log-log function conversion to reach normal approximation. The hazard ratio (HR) of PFS and its 95% confidential interval will be estimated using the stratified Cox proportional hazard model and the same stratification factors as the randomization factors.

**Secondary efficacy endpoints:**

Secondary efficacy endpoints are as follows:

- Overall survival (OS) is defined as the time from randomization to death for any cause. Patients without any death record will be censored on the last known survival date. Patients without any follow-up information will be censored on the date of randomization.
- The overall response rate (ORR) is defined as the proportion of subjects with the best overall response (BOR) of complete response (CR) or partial response (PR). Where, BOR refers to the best response recorded between the date of randomization to the date of objectively recording progression according to RECIST 1.1 criteria or the date of starting the subsequent anti-tumor therapy (whichever comes first). ORR will be calculated separately based on assessment results by BIRC or the investigator according to RECIST 1.1.
- Disease control rate (DCR) is defined as the proportion of subjects with BOR of CR, PR or SD. DCR will be calculated separately based on assessment results by BIRC or the investigator according to RECIST 1.1.
- Duration of response (DOR) is defined as the time from first recorded response (CR or PR) to first recorded disease progression, or to death, whichever is earlier. DOR is analyzed only for the patients with BOR of CR or PR. Patients without progression or death after achieving response will be censored on the date of the last tumor evaluation; if no tumor evaluation is performed after achieving response, the patients will be censored on the day of achieving response. DOR will be calculated separately based on assessment results by BIRC or the investigator according to RECIST 1.1.
- Time to response (TTR) is defined as the time from randomization to first recorded response (CR or PR). TTR will be calculated only for the patients with BOR of CR or PR, without censoring. TTR will be calculated separately based on assessment results by BIRC or the investigator according to RECIST 1.1.

The above method used for the primary efficacy endpoint PFS will be used for the secondary efficacy endpoints OS, DOR and TTR. The percentage of subjects in each treatment group will be calculated for ORR and DCR, Clopper-Pearson method will be used to calculate its 95% confidential interval, and the 95% confidential interval for the percentage difference between groups will be estimated using normal approximation. Kaplan-Meier method will be used to estimate the 1-year OS rate, 6-month and 1-year PFS rate, and 95% confidence interval will be estimated by Greenwood Formula.

The analysis methods for more efficacy endpoints are described detailedly in the Statistic Analysis Plan.

### Safety

The safety analysis of subjects will be performed by the actual treatment group. Adverse events will be coded using the Medical Dictionary for Regulatory Activities (MedDRA). All AEs, SAEs, death causing AEs, AEs leading to discontinuation or termination of trial medication reported during the study period will be analyzed based on systematic organ classification and preferred terminology, severity, relations with study drug, etc.

The clinical laboratory test data at each visit will be summarized. The values of laboratory data collected during treatment will be classified as low, normal or high level based on normal range of the laboratories used in this study. The number and proportion of patients undergoing the change in clinical laboratory value after baseline during treatment will be summarized.

The vital signs and 12 lead ECG measured in each visit will be summarized and described.

### Biomarkers Analysis

The following endpoints should be summarized, based on the efficacy-evaluable analysis set and per-protocol set. Include (but not limited to):

- To analyze the correlation between PD-L1 expression in tumor tissue and the antitumor activity of JS001 combined with chemotherapy
- Correlation analysis between expression of other potential pharmacodynamic indicators and antitumor activities of the combination of JS001 with chemotherapy
- To evaluate the change in TBNK lymphocyte subset during treatment with Pemetrexed and carboplatin combined with JS001 and analyze its correlation with antitumor activity

## Interim analysis

The interim analysis of efficacy is planned to be conducted when 214 cases of PFS events are observed. The analysis will be performedand the summary report will be reported by the independent statistical support group (iSSG), and the results of the interim analysis will be reviewed by independent data monitoring committee (iDMC) to provide the sponsor with suggestions. Please see iDMC Regulations & Rules for the establishment, responsibilities and data monitoring plan of iDMC.

Table 23 Efficacy boundary conditions for PFS analysis by α - consumption function of Pocock type (LAN DeMets method approximation)

| **PFS Analysis** | **Information ratio ^a^** | **Number of PFS events** | **Efficacy margin (one-sided p-value)** |
| --- | --- | --- | --- |
| Interim analysis | 60% | 214 | 0.0177 |
| Primary Analysis | 100% | 356 | 0.0128 |
| 1. The information ratio refers to the proportion of PFS events required for analysis to the total number of PFS events required for scheduled primary analysis. | | | |

# Study Management

This study shall be carried out in strict accordance with the laws and regulations for clinical studies in China, including GCP, Declaration of Helsinki (edition 2008), and other regulations, as well as this study protocol. The specific procedures for implementation of the study shall be subject to the standard operation procedures of the parties participating the study.

## Ethical considerations

Study protocol, ICF, eCRF, and some other materials must be submitted to IRB/IEC for approval prior to initiation of the study. IRB/IEC shall review and approve those materials in strict accordance the requirements of relevant laws and regulations. The study shall not be initiated until receipt of the approval document by IRB/IEC.

During the study, any change to the protocol shall be reviewed and approved by IRB/IEC prior to any specific implementation.

## Informed consent

The investigator or a designee shall be responsible for explaining, to each patient and the legal representative of each patient, study background, pharmacological characteristics of the investigational product, study protocol, and benefits and risk from participation of the study, and shall obtain the written informed consent signed by the patient or his/her legal representative and the investigator doctor prior to enrollment of the patient.

The final ICF document shall include the following contents: Study objective, study procedure, patients' obligations, and predictable benefits and predictable risk & inconveniences for patients participating the study; in case of any study-related impairment, treatment and appropriate insurance compensation that the patient may acquire; consultation of the study data, and confidentiality of patients' information, etc. A written approval opinions for ICF from the relevant regulatory authority should be obtained, and the ICF is written in a readable language by patients.

The patient or his/her legal representative, and the investigator or his/her designee should sign and date ICF; the informed consent process shall be recorded in the medical history or clinical records for each patient shall record, and a written informed consent shall be obtained prior to participation of the trial. The original ICF document shall be kept by investigator and the patient (one copy by the investigator and one copy by the patient). If important new data involved with the investigational product are found, then ICF shall be revised in writing and submit to the relevant regulatory authority for approval and the informed consent shall be obtained again.

The informed consent shall also contain the following attached signature page:

- If a patient develops the first imaging progression that complies with the criteria as specified in Section 3.1, and wants to continue treatment, then the page shall be signed after approval by the attending doctor. If a patient develops the first imaging progression, then the informed consent may be signed after the patient must be discussed for available optional therapies in advance, as well as any potential risk from continued treatment.

## Institutional Ethics Committee (IEC)

The sponsor and investigators will prepare all the relevant materials, including the protocol, informed consent form, copy of investigator’s brochure, approval letter and drug inspection report from relevant authorities and any advertisement for recruiting subjects, which will be provided to IRB/EC by investigators. The ethical written approval document shall be submitted by the researcher to Shanghai Junshi Biosciences Co., Ltd. The trial can not be initiated before acquisition of the protocol and informed consent form approved by IRB/EC by investigators and receipt of the copy of letter of ethical approval by the sponsor. In accordance with IRB/EC rules and regulations at the study center, all the amendments, regular progress reports and report of serious adverse events must be submitted to IRB/EC in real time.

## Protection of patient's rights and interests

In order to ensure conduction of this clinical trial according to ethical requirement, investigators should comply with internationally recognized guideline, the study is intended to acquire the scientific knowledge derived from this trial whilst minimizing the risk of participants, and will be helpful for better investigation of the disease.

The participants will provide the written informed consent form as to demonstrate that they are voluntary to participate in this trial. The updated safety information will be provided to investigators, institutional review board and patients, so that the patients can consider the relevant and updating information that may affect their voluntariness in continuous participation in the trial.

## Compensation for health damage of subjects

Patients will not receive any compensation for participation in the trial. However, various reasonable temporary advances resulted from participation in the trial (e.g., travel expenses) can be compensated by the sponsor.

The sponsor has purchased the clinical trial insurance for subjects and shall be liable for treatment cost for any damage that occurs in a subject from participation in the study and that is causally related with the study, and shall give corresponding economic compensation in accordance with the national relevant legal regulations. However, the sponsor shall not be liable for any damage caused by medical accident or due to non-compliance to the study protocol.

## Recording and storage of study data

The data in this trial will be captured using electronic case report form (EDC). Filling of CRF will be completed and signed by the principal investigator or his/her authorized representative at each base.

The initial entry and record of subsequent changes should be maintained and include the following information: time and date of the entry, signature of the person doing the entry or change.

If the patient can not complete the trial, i.e., withdrawal from the trial, the reason for the withdrawal must be recorded in EDC. If the patient withdraws from the trial due to an adverse event, try to clearly document the final result.

Investigators should ensure the accuracy, completeness and instantaneity of the data reported to the sponsor in EDC and all the reports required to be provided.

To ensure tte evaluation and supervision by NMPA and the sponsor, the Investigator shall agree to keep all study data, including confirmation records of all subjects (which may be used to check efficiently all recording data, such as eCRF and hospital original records), all original signed ICFs of all subjects, all eCRFs, the detailed records for drug distribution, etc. They shall be kept for 5 years after the end of study or until the sponsor's notice for destruction.

All the materials of this clinical study are the sponsor's properties. Unless required by NMPA, the investigators shall not provide them to any third party in any form without a written permission by the sponsor.

## Original data management

### Types of source data

The source data include all the information in the original records, clinical findings, observations or copy of the clinical record of other activities in the clinical trial required for trial organization and evaluation. Such as:

1. Admission/outpatient note;
2. Doctor’s and nurse’s medical order;
3. Notepad;
4. Original laboratory reports;
5. ECG, X-ray films;
6. Pathology and special evaluation report;
7. Signed informed consent form;
8. Inquiry letter;
9. Screening and enrollment record.

### Direct entry in the source data/record

Once trial related monitoring and audit, IRB/EC evaluation and/or inspection requirement by regulatory authorities occur, investigators/institutions should agree the sponsor or their authorized representative to look up the original material/document.

Monitor is responsible to ensure:

1. All the materials recorded in CRF are valid.
2. Patient’s safety and rights are protected.
3. The trial is conducted in accordance with currently approved protocol, GCP and all the regulatory requirements in operation.

## Return or Destruction of the investigational product/Treatment Supplies

At the end of the study, any remaining unused investigational product, as well as any remaining solution of used drugs and empty vials of Toripalimab Monoclonal Antibody Injection are recovered to the sponsor in a centralized way or destroyed by a designed third party; any remaining solution and empty vials of chemotherapeutics can be destroyed in the study center, and it shall be assured to record the destroy procedures or fill the destroy documents.

## Quality control and quality assurance

In order to ensure the quality of study, the clinical study plan is discussed and established jointly by the sponsor, CRO and the investigators prior to the initiation of the formal study. Relevant investigators participating the study are trained for GCP.

Each study center must manage the investigational product according to SOP, including receipt, storage, dispensation and recovery.

## Supervision and Auditing

**Monitoring:**

The clinical monitor authorized by the sponsor shall have the right to consult eCRF, ICF, and all original data.

The clinical monitor shall be responsible for formulating the plan and procedures that must be followed during the study. An on-site visit shall be carried out prior to initiation of the study. A regular visit shall be paid during performance of the study. If required, the subject shall be contacted by telephone, fax, or mail, as supplement to the on-site visit.

Prior to initiation of the study, the investigator shall be notified of the expected frequency of monitoring visits . Additionally during the study, the investigator shall be notified in advance prior to each monitoring visit. The visit is intended to ensure that the clinical study shall be carried out in strict accordance with the study protocol; integrity and accuracy of the case report form, that may be verified from the original documents.

The clinical monitor shall check that all eCRFs are filled in correctly and completely and are consistent with the original data; all the errors and missing data have been modified or noted, signed and dated by investigators. At each visit, the investigator and the clinical monitor shall cooperate closely, to check and verify the case report form, drug supply and inventory records, drug distribution and recovery records, and other scheduled additional records.

The sponsor or a person authorized by the sponsor may check quality of the study; the checker have the right to examine all study-related medical records, investigator's files and correspondences, and ICF.

**Auditing:**

In due course, the auditor shall audit the study in accordance with relevant SOPs, to ensure that the study are carried out in accordance with the national relevant laws and regulations, SOPs, and the protocol. The audit range include office documents and the research site's documents. After the end of auditing, the auditor shall submit a written report to describe any problem found and propose suggestions; relevant persons (investigator and auditor) shall adopt corresponding corrective measures and record in writing.

## Amendments tostudy protocol

For any significant amendment to the protocol, a written amendment shall be obtained that is agreed by the sponsor and the investigators, and submitted to IRB/IEC for approval and sent to NMPA for filing.

## Violation to the protocol

If enrolled by mistake, any subject who is deviated obviously from the predefined criteria of the protocol should withdraw from the study.

At each return visit, the investigator shall inquire the subject for whether there is any concurrent medications since the last visit, to judge whether the treatment violates the protocol, and meanwhile to record such information.

## Study summary report

After the end of the study, the investigators will make an objective summary based on the study results, carry out a statistical analysis on the study data using proper statistical methods, and finish an objective evaluation on the drug safety according to the study results, and after reviewed and agreed by the sponsor, they will make a written summary report of the clinical study.

## Confidentiality, and publication of study results

Objective, content and results of this clinical trial as well as all the future information must be strictly confidential. All materials and results are copyrighted by the sponsor.

After the end of the trial or when the data have been adequate (through sponsor’s rational judgment), investigators can prepare the data sourced from the trial for publication. Prior to public release, such data should be submitted to the sponsor to review and issue opinions. In order to ensure the sponsor can issue opinions and propose relevant suggestions, the materials for public communication should be submitted to the sponsor for review at least 60 days prior to submission for publication, public communication or review by the public release committee.

Investigators must agree that all the reasonable opinions issued by the sponsor and related with the article to be published by investigators will be added in the article by investigators.

During review of the article to be published, the sponsor has the right to postpone publication of the article in order for the sponsor to take measures to protect their patent information. All the materials related with the trial can not be published without the sponsor’s written approval. Except legal cause, the sponsor or the principal investigator can not reveal the trial results to any third party before bilateral agreement on data analysis and interpretation is reached.

# Quality control of trial

In order to ensure the accuracy, consistency, completeness and reliability of the trial data generated in this protocol, the study should be conducted in accordance with the standard operating procedures (SOPs) of the investigator site, GCP guideline and relevant regular requirements of NMPA.

This trial will be monitored by the clinical monitors from the sponsor or their designated unit. The on-site follow-up will be performed prior to initiation of the trial at the center and at appropriate time points during conduction of the trial. The communication record will also include teleconference and correspondence.

In accordance with the requirements in GCP guideline, the supervisors must be allowed to look up the original documentation of investigators, so as to check: consistency of the data recorded in eCRFs; patient’s safety and rights protection; whether the trial is conducted in accordance with the currently approved protocol and the requirements of all effective regulations.

Investigators agree to cooperate with relevant inspections fully in accordance with the requirement in the Protocol in a written form, and allow authorized personnel to look up all the documentations directly. Various materials directly recorded in eCRFs and regarded as the original materials will be locked prior to the conduction of the trial.

Each study base may be audited by the auditors sent by the sponsor or inspected by the regulatory authorities. In case of such audit/inspection, investigators should agree to allow inspectors to look up the original documents directly and schedule time to discuss various findings with relevant staff.

# Amendments of and deviation from trial protocol

## Amendment of Study Protocol

In case the protocol needs to be changed following approval, the written amendment signed by the same personnel needs to be provided. The amendment should be informed to the ethics committee and NMPA involved for filing. The changes that have a significant effect on the patient’s safety in the trial need to be approved by the responsible ethics committee and submitted to NMPA for filing.

The amendment should be distributed to all the personnel involved in conduction of the trial. Change to all the procedures required in the amendment should be informed to all the personnel.

## Deviation from Study Protocol

Major deviation from the protocol is defined as various events or behaviors of patients or investigators leading to inability to evaluate the primary objective of this protocol or unreliable evaluation results.

The ethics committee should establish, record in a written form and follow up the procedures, the established procedures should include: any deviation from the protocol or any change to the protocol can not be performed prior to written approval of corresponding amendment, except the necessary deviation as to eliminate immediate risk, or changes only involving the logistics or administration of the trial.

Without the sponsor’s consent and prior review and written approval of the amendment by the ethics committee, investigators can not make any deviation from the protocol or any change to the protocol, except the necessary deviation as to eliminate the immediate risk or changes only involving logistics or administration of the trial (e.g., changing monitors or telephone number). In order to eliminate the immediate risk, investigators can make any deviation from the protocol or any change to the protocol without prior approval by the ethics committee. Investigators should submit deviations or changes conducted, reasons for deviation or change, and planned amendment to the protocol where possible, if appropriate:

- - Submit to the ethics committee for review and approval;
  - Submit to the sponsor for consent;
  - Submit to the regulatory authorities.

Investigators or their designated personnel should record of and make explanations for various conditions that have deviated from the approved protocol.

# Trial discontinuation and closure

## Trial discontinuation required by the sponsor

The sponsor retains the right to interrupt or permanently discontinue conduction of this trial at one study base or all the study bases for various reasons at any time (including but not limited to safety or ethical issue or serious violation from relevant requirement).

In special circumstances, the trial can be terminated in one separate trial base, if the sponsor has reasonable reason, for example, trial fraud is suspected or the trial does not comply with the guideline in Good Clinical Practice.

If the sponsor makes a decision that such action is necessary, the sponsor will discuss this question with investigators (including the reason for taking such action). The sponsor will inform investigators of interruption of this trial in advance, prior to its conduction.

If the trial is interrupted or terminated for safety reason, the sponsor will inform all the other investigators and/or institutional organizations conducting the trial immediately, and the interruption or termination of the trial as well as the reason will also be informed to the regulatory authorities. If required by corresponding regulations, investigators must inform the ethics committee immediately and provide the reason for the interruption or termination.

## End of trial

The sponsor reserves the right to stop the study at any time due to medical reasons or any other reasons. If the study is terminated prematurely or discontinued, then the sponsor should notify immediately the investigator that the study is terminated or discontinued, and explain the reasons for termination or discontinuation of the study. According to the requirements of relevant laws and regulations, the sponsor or investigator should also notify IRB/IEC that the study is terminated or discontinued and explain the reasons.

The investigator reserve the right to judge whether the study should be stopped. If the investigator terminates or discontinues the study without any prior approval by the sponsor, the investigator should notify immediately the sponsor and IRB/IEC, and provide a detailed written explanation for termination or discontinuation of the study to the sponsor and IRB/IEC. Study records must be stored.

If there are subjects who are still receiving the investigational product at that time, they will be transferred to one extension study to continue to receive the drug therapy in this study, until inability to continue benefit as judged by investigators, progression of disease, intolerable toxicity, investigator’s decision, withdrawal of informed consent or death.

Once the trial ends, the monitors will conduct the following work together with investigators or staff at the base:

- Return all the trial materials (except the original materials where the names are not hidden) to the sponsor.
- Data inquiries;
- Traceability, harmonization and disposition of unused investigational product;
- Check the integrity of the trial record in the site;
- The biological samples are sent to the analytical laboratory.

If the trial is permanently discontinued, all the trial materials must be returned to the sponsor. In addition, all the unused investigational products will be disposed in accordance with corresponding procedure. The economic compensation for investigators and/or institutional organizations will refer to the agreement between investigators and the sponsor.

The sponsor will inform the ethics committee that the trial has ended within 90 days after end of the clinical trial. If the trial needs to be terminated prematurely, this time period will be reduced to 15 days and an unambiguous interpretation will be made for its reason.

# References

1. Jemal A， Bray F， Center MM， et al. Global Cancer Statistics. CA Cancer J Clin 2011; 61:69-90.
2. Siegel R， Naishadham D， Jemal A. Cancer Statistics， 2012. CA Cancer J Clin. 2012; 62:10-29.
3. CLOBOCAN 2008. Estimated cancer incidence: mortality， prevalence and disability-adjusted life years(DALYs) Worldwide in 2008. Available at: http://globocan.iarc.fr/factsheets/cancers/lung.asp.
4. Molina JR， Yang P， Cassivi SD， et al. Non-small cell lung cancer: epidemiology， risk factors， treatment， and survivorship. Mayo Clin Proc 2008; 83:584-94.
5. Howlader N， Noone AM， Krapcho M， et al. SEER Cancer Statistics Review， 1975-2011， National Cancer Institute. Bethesda， MD， http://seer，cancer.gov/csr/1975_2011/， based on November 2013 SEER data submission， posted to the SEER web site， April 2014.
6. Travis WD， Brambilla E， Noguchi M， et al. International association for the study of lung cancer/American thoracic society/European respiratory society international multidisciplinary classification of lung adenocarcinoma. J Thorac Oncol 2011; 6:244-285.
7. Langer CJ， Besse B， Gualberto A， et al. The evolving role of histology in the management of advanced non-small-cell lung cancer. J Clin Oncol 2010;28:5311-20.
8. Cetin K， Ettinger DA， Hei YJ， et al. Survival by histologic subtype in stage IV non-small cell lung cancer based on data from the Surveilance， Epidemiology and End Results Program. Clin Epidemiol 2011;3:139-48.
9. Hirsch FR， Spreafico A， Novello S， et al. The prognostic and predictive role of histology in advanced non-small cell lung cancer: a literature review. J Thorac Oncol 2008;3:1468-81.
10. Herbst RS， JV Heymach JV， Lippman SM. Lung cencer. N Engl J Med 2008;359:1367-80.
11. Ciuleanu T， Brodowicz T， Zielinski C， Kim JH， Krzakowski M， Laack E， et al. Maintenance pemetrexed plus best supportive care versus placebo plus best supportive care for non-smallcell lung cancer: a randomized， double-blind， phase 3 study. Lancet 2009;374:1432-40.
12. Paz-Ares LG， de Marinis F， Dediu M， Thomas M， Pujol JL， Bidoli P， et al. PARAMOUNT: Final overall survival results of the phase III study of maintenance pemetrexed versus placebo immediately after induction treatment with pemetrexed plus cisplatin for advanced nonsquamous non-small-cell lung cancer. J Clin Oncol 2013;31:2895-902.
13. Scagliotti GV， Parikh P， von Pawel J et al. Phase Ⅲ study comparing cisplatin plus gemcitabine with cisplatin plus pemetrexed in chemotherapy-naive patients with advanced-stage non-small-cell lung cancer. J Clin Oncol， 2008，26(21):3543-3551.
14. Paz-Ares LG， de Marinis F， Dediu M， et al. Maintenance therapy with pemetrexed plus best supportive care versus placebo plus best supportive care after induction therapy with pemetrexed plus cisplatin for advanced non-squamous non-small-cell lung cancer (PARAMOUNT): a double-blind， phase 3， randomised controlled trial.Lancet Oncol， 2012， 13(3):247-255.
15. Paz-Ares LG， de Marinis F， Dediu M， et al. PARAMOUNT: final overall survival results of the phase Ⅲ study of maintenance pemetrexed versus placebo immediately after induction treatment with pemetrexed plus cisplatin for advanced nonsquamous non-small-cell lung cancer. J Clin Oncol， 2013， 31(23):2895-2902.
16. Huang Y，Zhang J，Zhao YY，et a1．SPARC expression and prognostic value in non-small cell lung cancer [J]．Chin j Cancer，2012．31(1 1)：541—548．DOI：10．5732／cjc．012．10212.
17. Yared JA．Tkaczak KH．Update on taxane development：new analogs and new formulations[J]．Drug Des Devel Therapy，2012，6：371-384．DOI：10．2147／13DDT．S28997.
18. Vishnu P， Roy V．Safety and efficacy of nab-paclitaxel in the treatment of patients with breast cancer l J—Breast Cancer(Auckl)，2011，5：53-65．DOI：10．4137／BCBCRS5857.
19. Von Hoff DD，Ervin T，Arena FP，el a1．Increased survival in pancreatic cancer with nab-paclitaxel plus gemcitabine[J]．New Engl J Med， 2013， 369(18)： 169l-1703 DOI：10.1056／NEJMoal304369.
20. Zhang C，Awasthi N，Schwarz MA，et a1．Superior antitumnr activity of nanopartiele albumin-bound paclitaxel in experimental gastric cancer[J]．PLoS One，2013，8(2)：e58037．DOI：10.1371，joumal．pone．0058037.
21. ouchi M，Okamoto I，Sakai H，et a1．Efficacy and safety of weekly nab-paclitaxel plus carboplatin in patients with advanced non-small cell lung cancer[J]．LungCancer，2013，81(1)：97-101．DOI：10．1016／j．1ungean．2013．02．020.
22. D’Addario G， Fruh M， Reck M， Baumann P， Klepetko W， Felip E， et al. Metastatic non-small cell lung cancer: ESMO clinical practice guidelines for diagnosis， treatment and follow-up. Ann Oncol 2010;21(Suppl 5):116-9.
23. Howlander N， Noone AM， Krapcho M， Garshell J， Neyman N， Altekruse SF， et al. SEER cancer statistics review， 1975-2011. Bethesda (MD): National Cancer Institute. Available from:URL:http://seer.cancer.gov/csr/1975_2011/. Based on November 2013 SEER data submission(posted to the SEER web site， April 2014).
24. Bonomi PD. Implications of key trials in advanced nonsmall cell lung cancer. Cancer 2010;116:1155-64.
25. Sandler A， Gray R， Perry MC， Brahmer J， Schiller JH， Dowlati A， et al. Paclitaxel-carboplatin alone or with bevacizumab for non-small-cell lung cancer. N Engl J Med 2006;355:2524-50.
26. Scagliotti GV， Parikh P， von Pawel J， Biesma B， Vansteenkiste J， Manegold C， et al. Phase III study comparing cisplatin plus gemcitabine with cisplatin plus pemetrexed in chemotherapy naive patients with advanced-stage non-small-cell lung cancer. J Clin Oncol 2008;26:3543-51.
27. Schiller JH， Harringdon D， Belani CP， Langer C， Sandler A， Krook J， et al. Comparison of four chemotherapy regimens for advanced non-small-cell lung cancer. N Engl J Med 2002;10:92-8.
28. Langer CJ， Gadgeel SM， Borghaei H， Papadimitrakopoulou VA， Patnaik A， Powell SF， et al.Carboplatin and pemetrexed with or without pembrolizumab for advanced， non-squamous nonsmall-cell lung cancer: a randomised， phase 2 cohort of the open-label KEYNOTE-021 study.Lancet Oncol. 2016 Nov;17(11):1497-1508.
29. Gandhi L， Rodríguez-Abreu D， Gadgeel S，et al.Pembrolizumab plus Chemotherapy in Metastatic Non-Small-Cell Lung Cancer.N Engl J Med. 2018 May 31;378(22):2078-2092.
30. 2018 ASCO Annual Meeting.Phase 3 study of carboplatin-paclitaxel/nab-paclitaxel (Chemo) with or without pembrolizumab (Pembro) for patients (Pts) with metastatic squamous (Sq) non-small cell lung cancer (NSCLC). J Clin Oncol 36， 2018 (suppl; abstr 105).
31. Arnold M， Soerjomataram I， Ferlay J， et al.Global incidence of oesophageal cancer by histological subtype in 2012.Gut 2015;64:381–7
32. Chan SH， Chew TS， Goh EH， et al.Impaired general cell-mediated immune functions in vivo and in vitro in patients with nasopharyngeal carcinoma.Int J Cancer 1976;18:139−44.
33. Chen BJ， Chapuy B， Ouyang J， et al.PD-L1 expression is characteristic of a subset of aggressive B-cell lymphomas and virus-as标准治疗iated malignancies.Clin Cancer Res 2013;19:3462-73.
34. Cutler， D.M.， Are we finally winning the war on cancer? J Econ Perspect， 2008. 22(4): p. 3-26.
35. Emens LA， Braiteh FS， Cassier P， et al.Inhibition of PD-L1 by MPDL3280A leads to clinical activity in patients with metastatic triple-negative breast cancer [abstract].San Antonio Breast Cancer Symposium 2014:PD1-6.
36. Merritt RE， Mahtabifard A， Yamada RE， et al. Cisplatin augments cytotoxic T-lymphocyte-mediated antitumor immunity in poorly immunogenic murine lung cancer. J Thorac Cardiovasc Surg 2003;126:1609-17.
37. Apetoh L， Ghiringhelli F， Tesniere A， et al. Toll-like receptor 4-dependent contribution of the immune system to anticancer chemotherapy and radiotherapy. Nat Med 2007;13:1050-59.
38. Hales RK， Banchereau J， Ribas A， et al. Assessing oncologic benefit in clinical trials of immunotherapy agents. Ann Oncol 2010;21:1944-51.
39. Kurra V， Sullivan RJ， Gainor JF et al. (2016) Pseudoprogression in cancerimmunotherapy: rates， time course and patient outcomes. J Clin Oncol 34:6580-6580.
40. Gettinger SN， Kowanetz M， Soria JC， et al.Molecular correlates of PD-L1 status and predictive biomarkers in patients with non-small-cell lung cancer (NSCLC) treated with the anti-PDL1 antibody MPDL3280A [abstract].International Association for the Study of Lung Cancer 15th World Conference 2013:MO19.09.
41. HUI R， GARON E B， GOLDMAN J W， et al. Pembrolizumab as first-line therapy for patients with PD-L1-positive advanced non small cell lung cancer: a phase 1 trial[J]. Ann Oncol， 2017， 28(4):874-881. DOI: 10.1093/annonc/mdx008.
42. YKI-JARVINEN H， BERGENSTAL R M， BOLLI G B， et al. Glycaemic control and hypoglycaemia with new insulin glargine 300 U/ml versus insulin glargine 100 U/ml in people with type 2 diabetes2 randomized 12-month trial including 6-month extension[J/OL]. Diabetes Obes Metab， 2015， 17(12): 1142-1149[2017-12-01].https://onlinelibrary.wiley.com/ doi/abs/10.1111/ dom.12532. DOI:10.1111/dom.12532.
43. RITTMEYER A， BARLESI F， WATERKAMP D， et al. Atezolizumab versus docetaxel in patients with previously treated non-smallcelllung cancer (OAK): a phase 3， open-label， multicentre randomized controlled trial[J/OL]. Lancet， 2017， 389(10066): 255-265[2017-12-01]. https://www. sciencedirect. com / science / article / piiS014067361632517X?via%3Dihub. DOI: 10.1016/S0140-6736(16)32517-X.
44. PASSIGLIA F， BRONTE G， BAZAN V， et al. PD-L1 expression as predictive biomarker in patients with NSCLC: a pooled analysis[J].Oncotarget， 2016， 7(15): 19738-19747. DOI: 10.18632 / oncotarget.7582.
45. HIRSCH F R， MCELHINNY A， STANFORTH D， et al. PD-L1 Immunohistochemistryassays for lung cancer: results from phase 1 of the blueprint PD-L1 IHC assay comparison project[J/OL]. J ThoracOncol， 2017， 12(2): 208-222[2017-12-01]. https://www. sciencedirect.com / science / article / pii / S1556086416335365? via% 3Dihub.DOI: 10.1016/j.jtho.2016.11.2228.
46. UBE J M. Unleashing the immune system: PD-1 and PD-Ls in the pre-treatment tumor microenvironment and correlation with response to PD-1/PD-L1 blockade[J/OL]. Oncoimmunology， 2014， 3(11): e963413[2017-12-01]. https://www.tandfonline.com/ doi/ full/10.4161 / 21624011.2014.963413. DOI: 10.4161 / 21624011.2014.963413.
47. BORGHAEI H， BRAHMER J， HORN L， et al. P2.35: nivolumab vs docetaxel in advanced NSCLC: CheckMate 017/057 2-Y update and exploratory cytokine profile analysis: track: immunotherapy[J/OL]. J Thorac Oncol， 2016， 11(Suppl 10): S237-S238[2017-12-01]. https://www.sciencedirect.com/science/article/pii/S1556086416308152?via%3Dihub. DOI: 10.1016/j.jtho. 2016.08.106.
48. BORGHAEI H， PAZ-ARES L， HORN L， et al. Nivolumab versus docetaxel in advanced non-squamous non-small cell lung cancer[J].N Engl J Med， 2015， 373(17): 1627-1639. DOI: 10.1056 / NEJ-Moa1507643.
49. Wungki P， Gilberto L， Deukoo K， et al. Correlating ISEND and tumor mutation burden (TMB) with clinical outcomes of advanced non-small cell lung cancer (ANSCLC) patients on nivolumab. Poster in the 18th World Conference on Lung Cancer.
50. ALEXANDROV L B， NIK-ZAINAL S， WEDGE D C， et al. Signatures of mutational processes in human cancer[J/OL]. Nature， 2013，500(7463): 415-421. DOI: 10.1038/nature12477.
51. KILLOCK D. Lung cancer: frontline nivolumab - CheckMate 026 ends in stalemate[J]. Nat Rev Clin Oncol， 2017， 14(8): 458-459.DOI: 10.1038/nrclinonc.2017.102.
52. ADAMS D L， ADAMS D K， HE J， et al. Sequential tracking of PDL1 expression and RAD50 induction in circulating tumor and stromal cells of lung cancer patients undergoing radiotherapy[J / OL].Clin Cancer Res， 2017， 23(19): 5948-5958[2017-12-01]. https://clincancerres. aacrjournals. org / content / 23 / 19 / 5948. long. DOI:10.1158/1078-0432.CCR-17-0802.
53. NICOLAZZO C， RAIMONDI C， MANCINI M， et al. Monitoring PD-L1 positive circulating tumor cells in non-small cell lung cancer patients treated with the PD-1 inhibitor nivolumab[J/OL]. Sci Rep，2016， 6: 31726[2017-12-01]. https://www.ncbi.nlm.nih.gov/pmc/articles/PMC4995431.DOI: 10.1038/srep31726.
54. KHAGI Y， GOODMAN AM， DANIELS G A， et al. Hypermutated circulating tumor DNA: correlation with response to checkpoint inhibitor-based immunotherapy[J / OL]. Clin Cancer Res， 2017， 23(19): 5729-5736[2017-12-01]. https://clincancerres.aacrjournals.org/content/23/19/5729. DOI: 10.1158/1078-0432.CCR-17-1439.

# Attachments

1. Study schedule

**Initial phase**

|  | Screening period  (baseline) | | Treatment period ^2^  One cycle consisting of 21 d (±3 d) | | | | | | | | | | | | | | | End of treatment  Visit 27 | Subsequent treatment visit ^27^ | | |
| --- | --- | --- | --- | --- | --- | --- | --- | --- | --- | --- | --- | --- | --- | --- | --- | --- | --- | --- | --- | --- | --- |
| DURATION OF TREATMENT |  |  | Cycle 1 | | | Cycle 2 | | | Cycle 3 | | | Cycle 4 | | | Cycle 5 and beyond | | |  | Safety follow-up | Follow-up visit | Survival follow-up |
| Window (days) | Within 28 days prior to randomization | Pre-randomization  14  Within XX days | C1D1 | C1D8±1 | C1D15±1 | C2D1  ±3 | C2D8±1 | C2D15±1 | C3D1  ±3 | C3D8±1 | C3D15±1 | C4D1  ±3 | C4D8±1 | C4D15±1 | CxD1±3 | CxD8±1 | CxD15±1 | Treatment withdrawal  ±3 | 30 ± 7 days after the last dose | Subjects withdrawing from the study due to non-disease progression will be evaluated synchronically with the tumor evaluation. | Every 3 months ±14 d after progressive disease or losing clinical benefits |
| General assessments | | | | | | | | | | | | | | | | | | | | | |
| Informed consent^1^ | × |  |  |  |  |  |  |  |  |  |  |  |  |  |  |  |  |  |  |  |  |
| Sub-study informed consent form for optional biomarkers | × |  |  |  |  |  |  |  |  |  |  |  |  |  |  |  |  |  |  |  |  |
| Demographic information^3^ | × |  |  |  |  |  |  |  |  |  |  |  |  |  |  |  |  |  |  |  |  |
| Inclusion/exclusion criteria | × |  |  |  |  |  |  |  |  |  |  |  |  |  |  |  |  |  |  |  |  |
| Past medical history | × |  |  |  |  |  |  |  |  |  |  |  |  |  |  |  |  |  |  |  |  |
| Concomitant Medications ^4^ | × | × | × |  |  | × |  |  | × |  |  | × |  |  | × |  |  | × | × |  |  |
| Prior tumor history^5^ | × |  |  |  |  |  |  |  |  |  |  |  |  |  |  |  |  |  |  |  |  |
| Subject Enrollment^6^ |  |  | × |  |  |  |  |  |  |  |  |  |  |  |  |  |  |  |  |  |  |
| Subsequent antitumor therapy |  |  |  |  |  |  |  |  |  |  |  |  |  |  |  |  |  | × | × | × | × |
| Survival status follow-up |  |  |  |  |  |  |  |  |  |  |  |  |  |  |  |  |  |  |  | × | × |
| Clinical evaluation | | | | | | | | | | | | | | | | | | | | | |
| Adverse events (AEs) collection ^7^ |  |  | × |  |  | × |  |  | × |  |  |  |  |  | × |  |  | × | × |  |  |
| Complete physical examination ^8^ | × |  |  |  |  |  |  |  |  |  |  |  |  |  |  |  |  |  |  |  |  |
| Targeted physical examination^9^ |  |  | × |  |  | × |  |  | × |  |  | × |  |  | × |  |  | × | × |  |  |
| Height & weight |  | × | × | × | × | × | × | × | × | × | × | × | × | × | × | × | × | × | × |  |  |
| Vital Signs | × |  | × |  |  | × |  |  | × |  |  | × |  |  | × |  |  | × | × |  |  |
| 12-lead ECG |  | × |  |  |  | × |  |  | × |  |  | × |  |  | × |  |  | × | × |  |  |
| Echocardiography ^[10]^ | × |  |  |  |  |  |  |  | × |  |  |  |  |  | × |  |  | × |  |  |  |
| Pulmonary function test ^11^ | × |  |  |  |  |  |  |  |  |  |  |  |  |  |  |  |  |  |  |  |  |
| ECOG score | × |  | × |  |  | × |  |  | × |  |  | × |  |  | × |  |  | × | × |  |  |
| Laboratory test (local laboratory)^12^ | | | | | | | | | | | | | | | | | | | | | |
| Serum pregnancy test (if applicable)^13^ |  | × |  |  |  | × |  |  | × |  |  | × |  |  | × |  |  | × | × |  |  |
| Coagulation function ^14^ |  | × |  |  |  | × |  |  | × |  |  | × |  |  | × |  |  | × | × |  |  |
| Hematological examination^15^ |  | × |  | × | × | × | × | × | × | × | × | × | × | × | × | × | × | × | × |  |  |
| Blood biochemical examination^16^ |  | × |  |  |  | × |  |  | × |  |  | × |  |  | × |  |  | × | × |  |  |
| Creatinine clearance ^17^ |  | × |  |  |  | × |  |  | × |  |  | × |  |  | × |  |  | × | × |  |  |
| Urine routine ^18^ |  | × |  |  |  | × |  |  | × |  |  | × |  |  | × |  |  | × | × |  |  |
| Stool routine + occult blood^18^ |  | × |  |  |  | × |  |  | × |  |  | × |  |  | × |  |  | × | × |  |  |
| Virological test^19^ | × |  |  |  |  |  |  |  |  |  |  |  |  |  |  |  |  |  |  |  |  |
| Thyroid function^20^ |  | × |  |  |  | × |  |  | × |  |  | × |  |  | × |  |  | × | × |  |  |
| Laboratory tests (central laboratory) | | | | | | | | | | | | | | | | | | | | | |
| Anti-drug antibody (ADA) with corresponding trough concentration^21^ |  |  | × |  |  |  |  |  |  |  |  |  |  |  | × |  |  | × |  |  |  |
| Biomarker test in peripheral blood ^22^ |  |  | × |  |  |  |  |  | × |  |  |  |  |  | × |  |  | × |  |  |  |
| Collection of tumor tissues | | | | | | | | | | | | | | | | | | | | | |
| PDL1 and TMB analysis for freshly acquired tissues ^23^ | × |  |  |  |  |  |  |  |  |  |  |  |  |  |  |  |  |  |  |  |  |
| When the lesions show partial response or disease progression occurs, the collection of tissue specimens is only applicable for the patient who has signed on the informed consent of the optional biomarker subgroup study | | | | | | | | | | | | | | | | | | | | | |
| EGFR and ALK test^24^ | × |  |  |  |  |  |  |  |  |  |  |  |  |  |  |  |  |  |  |  |  |
| Tumor evaluations | | | | | | | | | | | | | | | | | | | | | |
| Tumor evaluation ^25^ | × |  |  |  |  |  |  |  | × |  |  |  |  |  | × |  |  | × |  | × |  |
| Dosing of investigational drug ^26^ | | | | | | | | | | | | | | | | | | | | | |
| JS001 or placebo |  |  | × |  |  | × |  |  | × |  |  | × |  |  | × |  |  |  |  |  |  |
| Pemetrexed |  |  | × |  |  | × |  |  | × |  |  | × |  |  | × |  |  |  |  |  |  |
| Cisplatin/carboplatin ^28^ |  |  | × |  |  | × |  |  | × |  |  | × |  |  |  |  |  |  |  |  |  |
| Nab-paclitaxel ^29^ |  |  | × | × | × | × | × | × | × | × | × | × | × | × | × | × | × |  |  |  |  |

Comments:

1. The informed consent form must be obtained prior to the study-specific research procedures;
2. One treatment cycle consists of 3 weeks. Subjects who continue treatment after progression shall still follow the previous treatment plan until a second progression;
3. Demographic data: including date of birth, sex, self-reported ethnicity;
4. All drug treatments of patients must be recorded in the case report form within 30 d prior to the first dose, including generic name of the drug product, daily dose, reasons for treatment with the drug, starting date and ending date;
5. The past tumor history includes diagnosis date of tumor, start/end date of previous therapy, optimal treatment evaluation, date of progressive disease; the radiography history includes start/end date and radiography site.
6. Subject enrollment: After the verification of the subjects’ inclusion/exclusion criteria and the confirmation of the subjects’ eligibility, the investigators should login RTSM system for randomization within 3 days prior to the first dose;
7. Adverse events (AE) should be collected within 30 days from the initiation of the first dose to the last dose or the starting of a new antitumor therapy (whichever comes first). Serious adverse events should be reported from signing the informed consent form to the administration of the first dose;
8. Physical examination: including head, eyes, ear, nose, throat, neck, heart, chest (including lungs), abdomen, four limbs, skin, lymph nodes, nervous system, and patient's general condition;
9. For the dosing cycle where a complete physical examination is not required according to the flow chart for trial, a specialty physical examination will be conducted by the investigator according to clinical application before the trial treatment is implemented. New clinically significant findings should be recorded as AEs.
10. Left ventricular ejection fraction is measured by echocardiography. The examination is carried out once per 2 cycles in the first 12 cycles and thereafter once per 3 cycles since C1D1;
11. Pulmonary function test including the forced vital capacity, peak mid-expiratory flow (FEF25-75), peak expiratory flow (PEF), forced expiratory volume in one second, and diffusion lung capacity for carbon monoxide (DLCO) will be performed during the screening period;
12. Except for C1D1, the laboratory tests during the treatment period should be completed prior to each medication, only the results of hematological examination, coagulation function and blood biochemical test (including creatinine clearance rate) within 3 days prior to administration and results of other laboratory tests within 7 days before administration are acceptable. The results of laboratory examination must be determined by the investigator to meet the criterion for continuous medication before starting medication;
13. Females of childbearing potential must have a serum pregnancy test within 72 hours prior to the first dose of investigational drug. Afterwards, the pregnancy test should be conducted within 7 days before weekly treatment until safety follow-up and visit.
14. Coagulation function: international normalized ratio (INR), prothrombin time (PT), activated partial thromboplastin time (aPTT); test during screening shall be completed within 7 days prior to first dose.
15. Hematological examination: red blood cell count, hemoglobin, hematocrit, white blood cell count with differential (neutrophils, lymphocytes, eosinophils, monocytes, basophils), and platelet count; test during screening shall be completed within 7 days prior to first dose. If the neutrophil is ≤1.0×10^9^/L or platelet count is ≤50×10^9^/L, the frequency of repeated test needs to be increased (once per 2-3 days); if the dose is interrupted or adjusted for hematological toxicity, the complete blood cell count needs to be repeated every week until normalization; the visit procedure of day 8 and day 15 is for those subjects receiving nab-paclitaxel only, and shall be completed within 2 days prior to treatment with nab-paclitaxel.
16. Blood biochemistry test includes total protein, albumin, globulin, blood glucose, total cholesterol, low density lipoprotein, high density lipoprotein, triglycerides, urea / urea nitrogen, creatinine, alkaline phosphatase, lactate dehydrogenase, creatine kinase, creatine kinase isoenzyme, total bilirubin, direct bilirubin, indirect bilirubin, AST, ALT, calcium, phosphorus, magnesium, potassium, sodium, chloride, serum amylase, and uric acid; test during screening shall be completed within 7 days prior to first dose. If ALT or AST increases by 3 times or 2 times higher than abnormal value of baseline during the test, the examination frequency should be increased (recommended to be 1-2 times / week);
17. Creatinine clearance (Ccr) is to be calculated as: Ccr=(140-age) ×weight (kg)/[72 (kg)×Scr(mg/dl)] or Ccr=[(140-age) ×weight (kg)]/[0.814×Scr(µmol/L)], the unit of creatinine should be noted in the calculation of creatinine clearance, the result calculated ×0.85 for women
18. Urine routine: specific gravity, pH, urine glucose, protein, cast, ketone bodies, and blood cells (including white blood cells urine and red blood cells urine); if urine protein test is ++ or more or the results are abnormal and clinically significant as judged by the doctor, then a 24 hour quantitative measurement of protein urine is required; Stool routine includes stool routine examination, including: stool color and shape, red blood cells, white blood cells, and occult blood;
19. Virology test includes two pairs of semi-hepatitis B (if HBsAg positive and / or HBcAb positive, then HBV DNA copy number has to be measured), HCV antibody, and HIV antibody; HBV DNA copy number has to be measured in case of HCV antibody positive
20. Thyroid function test: Thyroid stimulating hormone (TSH), serum free triiodothyronine (FT3), and serum free thyroid hormone (FT4) are measured once prior to dose of each cycle, and at the end-of-treatment visit; if a clinical significant change occurs in thyroid function, then it is suggested that a consultation by the Endocrinology Department is carried out and relevant pituitary function test is carried out;
21. The samples will be collected once prior to the first administration of treatment period of the study, within 60 minutes before each 4 -cycle medication in the first year, and then 60 minutes before each 8-cycle medication;
22. Only the subject from biomarker study participant is required to take peripheral blood biomarker sampling and detection (excluding the first blood collection, voluntary provision frequency of the patient should be completely followed for subsequent collections of samples). The collection will be made once respectively after randomization and before the first administration and at each imaging evaluation until the disease progresses. Approximately 8ml of peripheral venous blood is drawn at every time point for biomarker test.
23. All subjects must provide tumor tissue specimens for PD-L1 test in the central laboratory before enrollment, a retest can be requested if the test results fails to be evaluated due to conditions,; the methods including next-generation sequencing (NGS) and whole exome sequencing (WES) should be adopted for TMB.
24. The patient with unknown EGFR and ALK status will accept local laboratory report (a well validated and NMPA approved kit is to be used), and the patient with squamous cell carcinoma isn't required to subject to the tests of EGFR and ALK status.
25. Tumors are evaluated in accordance with the criteria of RECIST version 1.1 and irRECIST. Tumor evaluation at screening must be carried out within 4 weeks prior to first dose, and enhanced CT scan of chest, abdomen (including liver and adrenal gland), and pelvis should be carried out (unless contraindicated, oral / IV contrast agent should be used); if clinically indicated, an appropriate method may be used to examine any other known or suspected disease sites, such as head MRI, bone scan, or neck CT scan; if patients' tumor imaging examination for routine diagnosis and treatment prior to signing the informed consent are carried out within 4 weeks prior to enrollment and in our site, it is not necessary to repeat it. Baseline and subsequent evaluations should be carried out by the same imaging examination method, and should be carried out by the same investigator as far as possible. The tumor evaluation should be performed once in every 6 weeks in the first 12 months (window period +/7 days) using the cycle calculated based on C1D1, thereafter once in every 9 weeks (window period +/7 days) , and will not be affected by discontinuation of the drug. Patients who are suspected with progressive disease prior to the next scheduled tumor evaluation should receive an unscheduled tumor evaluation;
26. The investigational treatment includes two periods: induction treatment period and maintenance treatment period. The number of courses during the induction therapy will be 4-6. In the induction treatment phase, one course of treatment consists of 21 (± 3 d). On the first day of each course, the enrolled patients shall receive intravenously a fixed dose 240mg of JS001 / placebo, followed by pemetrexed + platinum or nab-paclitaxel + carboplatin chemotherapy, until occurrence of the following events (whichever is first): 4-6 courses of treatment are completed; progressive disease. After induction therapy, the patients with non-squamous NSCLC shall continue the maintenance treatment and one cycle of treatment still consists of 21 days (± 3 d). On the first day of each course, the enrolled patients shall receive intravenously a fixed dose 240mg of JS001 / placebo, followed by pemetrexed alone as chemotherapy, until that the investigator judges that the subject cannot benefit any more, or develops progressive disease or intolerable toxicity, or at the discretion of the investigator, or until the subject withdraws the informed consent or the subject dies. JS001 is administered every 3 weeks.
27. Subsequent treatment visits: If end of treatment visit falls within the safety follow-up period, no repeated safety visit is required. The survival follow-up shall be carried out every 3 months after the last dose (end of treatment), to collect subsequent antitumor therapies and SAEs as judged to be related to investigational drug; subjects terminated the treatment due to any reasons other than progressive disease should also receive an imaging evaluation every 6 weeks in the first 12 months, and every 9 weeks after 12 months until progressive disease, death, or initiation of a new antitumor therapy, or withdrawal of the informed consent (whichever is first). Then a survival follow-up should be carried out every 3 months (if applicable).
28. Drug selection of Cisplatin and Carboplatin: Pemetrexed combined with Cisplatin or Carboplatin can be used in the patients with non-squamous cell carcinoma (the selection on Platinum is to be determined by the investigator), and the use of nab-paclitaxel combined with Carboplatin is the only choice for the patients with squamous cell carcinoma.
29. The feasibility of administration of nab-paclitaxel on Day 15 of each cycle will be determined by the investigator.

**Crossover Phase**

It is only applicable to the subject whose PD is subject to the assessment according to RECIST1.1 or iRECIST and meets the requirements of crossover phrase.

|  | **Treatment period 1** | | | | | | | | | | | | | | |  |  | **Follow-up phase ^2^** | **Survival Follow-up^3^** |
| --- | --- | --- | --- | --- | --- | --- | --- | --- | --- | --- | --- | --- | --- | --- | --- | --- | --- | --- | --- |
| **DURATION OF TREATMENT** | **1^4^** | **2** | **3** | **4** | **5** | **6** | **7** | **8** | **9** | **10** | | **11** | **12** | **13** | **14 and onwards** | **End of treatment visit^[5]^** | **Safety follow-up visit ^6^** | **Follow-up visit and onwards** | **Survival follow-up** |
| **Time window (days):** | **+3** | **± 3** | **± 3** | **± 3** | **± 3** | **± 3** | **± 3** | **± 3** | **± 3** | **± 3** | | **± 3** | **± 3** | **± 3** | **± 3** | **Discontinuation of Investigational drug ±3** | **30 (±7 days) after the last dose** | **Simultaneously with tumor assessment for these subjects who have terminated the treatment due to any reasons other than progressive disease** | **Every 3 months ±7** |
| General assessments | | | | | | | | | | | | | | | | | | | |
| Prior and concomitant medication | X | X | X | X | X | X | X | X | X | | X | X | X | X | X | X | X |  |  |
| Subsequent antitumor therapy |  |  |  |  |  |  |  |  |  | |  |  |  |  |  | X | X | X | X |
| Survival status follow-up |  |  |  |  |  |  |  |  |  | |  |  |  |  |  |  |  | X | X |
| Clinical evaluation | | | | | | | | | | | | | | | | | | | |
| Adverse events (AEs) collection ^7^ | X | X | X | X | X | X | X | X | X | | X | X | X | X | X | X | X | X |  |
| Complete physical exam | X |  |  |  |  |  |  |  |  | |  |  |  |  |  |  |  |  |  |
| Targeted physical examination |  | X | X | X | X | X | X | X | X | | X | X | X | X | X | X | X |  |  |
| Vital signs, weight | X | X | X | X | X | X | X | X | X | | X | X | X | X | X | X | X |  |  |
| ECOG PERFORMANCE STATUS | X | X | X | X | X | X | X | X | X | | X | X | X | X | X | X | X |  |  |
| 12-lead ECG ^8^ | X | X | X | X | X | X | X | X | X | | X | X | X | X | X | X | X |  |  |
| Echocardiography ^[9]^ | X |  |  | X |  |  | X |  |  | | X |  |  | X | X | X |  |  |  |
| Laboratory test (local laboratory) ^10^ | | | | | | | | | | | | | | | | | | | |
| Hematology Panel | X | X | X | X | X | X | X | X | X | | X | X | X | X | X | X | X |  |  |
| Coagulation function | X | X | X | X | X | X | X | X | X | | X | X | X | X | X | X | X |  |  |
| Blood biochemical examination | X | X | X | X | X | X | X | X | X | | X | X | X | X | X | X | X |  |  |
| Routine urine test | X |  |  | X |  |  | X |  |  | | X |  |  | X | X | X | X |  |  |
| Stool routine + occult blood | X |  |  | X |  |  | X |  |  | | X |  |  | X | X | X | X |  |  |
| Thyroid Function | X | X |  | X |  | X |  | X |  | | X |  | X |  | X | X | X |  |  |
| Serum pregnancy test (if applicable) ^11^ | X | | | | | | | | | | | | | | | X | X |  |  |
| **Efficacy evaluation** | | | | | | | | | | | | | | | | | | | |
| Tumor imaging examination ^12^ | X |  |  | X |  |  | X |  |  | | X |  |  | X | X | X |  | X^13^ |  |
| **研究药物给药** | | | | | | | | | | | | | | | | | | | |
| JS001 | X | X | X | X | X | X | X | X | X | | X | X | X | X | X |  |  |  |  |
| 1. Normally, the evaluation /examination will be performed before the first administration of trial therapy on Day 1 of each cycle, unless otherwise indicated. Each treatment cycle is defined as 3 weeks (21 days ±3 days). If the treatment cycle is adjusted, all procedures will be performed based on the cycles rather than the weeks of treatment except for imaging examination, and the imaging examination will be performed every 9 weeks (63 days ± 7 days) starting from the first administration of trial therapy,without considering treatment delay. 2. For the convenience of the subject, all follow-up assessments can be performed at the same visit where the imaging examination is obtained. The subjects with progressive disease or initiatinga new antitumor therapy will enter the survival follow-up period directly. The subjects with AEs grade 1 or above will continue follow-up until AEs recovered to grade 0-1 or until initiation of a new anti-tumor therapy, whichever comes first. 3. Once the subject stops the imaging evaluation of this protocol (e.g. because of PD or commencement of new anti-tumor treatment), the subject enters the survival follow-up period and should be subject to the follow-up every 3 months to assess the survival status. The treatment post study and the subject's response will also be collected. 4. All procedures and assessments completed at the time of withdrawal from primary study can be used to study the startup of the crossover phase if appropriate. 5. The visit should be stopped when the study drug is discontinued for any reason. If the discontinuation visit is performed simultaneously with mandatory safety follow-up visit 30 days after the last dose of the study treatment, there is no need to repeat the procedure. 6. A mandatory safety follow-up visit shall be performed approximately 30 days after the last dose of the investigational drug or prior to the initiation of new anti-tumor treatment, whichever occurs first. The subjects with AEs grade 1 or above will continue follow-up until AEs recovered to grade 0-1 or until initiation of a new anti-tumor therapy, whichever comes first. If end of treatment visit falls within the safety follow-up period, no repeated safety visit is required. 7. During the study, all AEs shall be recorded until 30 days after the last dose of the investigational drug or the initiation of a new anti-tumor therapy (whichever occurs first); those AEs suspected to be related to the investigational drug shall be collected until 90 days after the last dose or the initiation of a new anti-tumor therapy (whichever occurs first). 8. 12-lead ECG shall be performed within 14 days prior to first dose of the investigational drug. ECG examination is allowed to be performed 72 hours before administration after the first cycle. 9. Echocardiography shall be performed within 14 days prior to first dose of the investigational drug. It shall also be performed in Cycle 14 and every 6 cycles thereafter. 10. Laboratory investigations at screening shall be performed within 14 days prior to first dose of the investigational drug. Only the results of hematological test, coagulation function test and blood biochemical test (including creatinine clearance rate) within 3 days before administration and after the first cycle are acceptable, and the results of other laboratory tests within 7 days before administration are acceptable. Laboratory results must be available before dosing. Urine routine and stool routine: performed once every 6 cycles since Cycle 14. T3 or FT3, FT4 and TSH: Once every 2 cycles from Cycle 14; 11. Females of childbearing potential have to have a serum pregnancy test within 72 hours prior to the first dose of the investigational drug. Thereafter, the pregnancy test should be implemented within 7 days before treatment in each cycle till safety follow-up visit 30 days after the last dose. 12. The tumor response assessment is required to be implemented every 9 weeks (63 ± 7 days) until PD or the subject starts other anti-tumor therapy. The investigator is responsible for determination of disease response or progression evaluation. 13. The subject who starts other anti-tumor treatments isn't required to take the tumor imaging examination. | | | | | | | | | | | | | | | | | | | |

1. Eastern Cooperative Oncology Group (ECOG) - Performance Status Scale

| **Grade** | **Criteria** |
| --- | --- |
| 0 | Fully active, able to carry on all pre-disease performance without restriction  (Karnofsky 90-100) |
| 1 | Restricted in physically strenuous activity but ambulatory and able to carry out work of a light or sedentary nature, e.g., light house work, office work  (Karnofsky 70-80) |
| 2 | Ambulatory and capable of all self-care but unable to carry out any work activities. Up and about more than 50% of waking hours  (Karnofsky 50-60) |
| 3 | Capable of only limited self-care, confined to bed or chair more than 50% of waking hours  (Karnofsky 30-40) |
| 4 | Completely disabled. Cannot carry on any self-care. Totally confined to bed or chair  (Karnofsky 10-20) |

1. Response Evaluation Criteria in Solid Tumors (RECIST1.1)

Response Evaluation Criteria in Solid Tumors RECIST Version 1.1

As RICIST version 1.1 is not published in any formal Chinese version, and this version is an internal translation. For more detailed information, please refer to the English version: <http://ctep.cancer.gov/protocolDevelopment/docs/recist_guideline.pdf>.

**Summary
Background information**

To evaluate any change of tumor burden is an important feature in clinical evaluation of cancer treatment. Both tumor volume reduction (objective response) and progressive disease are significant judgment endpoints. Since RECIST was published on 2000, many investigators, associations, enterprises, and governmental authorities have adopted the criteria to evaluate therapeutic efficacy. However, some problems emerged thereupon result in issuance of this amendment (version 1.1). The corrections (please see the topic of the respective chapters) are originated from evaluation of large-scale databases (more than 6500 patients), simulation studies, and literature reviews.

**Important corrections in RECIST version 1.1**

Main revisions include:

Judgment of the number of lesions: for the convenience of analysis, the evaluation of the data from many trials are combined into one database; according to the database, the total number of lesions required for judgment of tumor burden at the reaction endpoint is decreased from at most 10 to now at most 5 (that for each organ is decreased from at most 5 to 2).

Now the judgment for pathological lymph nodes is combined as: Nodes in a short axis value of 15 mm as judged are measurable target lesions for evaluation. For judgment of tumor response, the short axis value (of the nodular lesion) must be within the sum of (the radius of) the lesion. Nodes shrunk to <10 mm of short axis value may be considered as normal.

In clinical trials with response rate as the primary judgment endpoint, it has to confirm efficacy; however, it is not required in randomized, controlled clinical trials, as a control group has become a valid mode to explain data of the trial. progressive disease is clarified in the following aspects: Except the original definition —— the sum of the target lesion (radius) is increased by 20%, if the sum is very small, now the it must have a 5-mm increase of its absolute value (of the short axis of the lesion). Additionally, it has provided a guideline for "definite exacerbation" of unmeasurable or non-target lesion —— i.e. circumstances to be easily confused in the initial version of the RECIST guideline. Finally, one section is used specifically to introduce a test for new lesion, including interpretation of the scan results of FDG-PET. Imaging Guideline: The amended RECIST include new imaging appendix, in which the recommendations for evaluation of the optimal anatomy of lesions are provided.

The following work:

A critical problem considered by the working team in the amendment of RECIST 1.1 is: Evaluate whether tumor load is revised from 1-dimensional anatomy evaluation to 3-dimensional anatomy evaluation or whether the functional evaluation by PET and MRI is appropriate. The current conclusion is there is lacking sufficient criteria or evidences to give up the anatomical evaluation for tumor load. The only one explanation is that FDG-PET imaging is used as an adjuvant mean for judgment of condition. As discussed detailedly in the topic of the final chapter, the use of those uptodatetechnologies with enticing prospect requires corresponding clinical validation studies.

Keywords: Criteria for Response Evaluation; Solid Tumor; Guideline

1. Background

1.1 History of RECIST Criteria

To evaluate any change of tumor burden is an important feature in clinical evaluation of cancer treatment. The times to tumor reduction (objective response) and progressive disease are important judgment endpoints in a clinical trial of cancer. To screen new antitumor drugs, the evidences from studies of many years supports that tumor reduction is used as an endpoint of Phase II trial. Those studies suggest that, for solid tumors, a drug that may promote tumor reduction in some patients may be later proved possibly (imperfect though) to increase a patient's overall survival or allow patients to have an opportunity to enter event evaluation in a Phase III randomized trial. Presently, among the indicators to evaluation treatment effects in Phase II screening trials, objective response is more reliable than any other biomarkers. Moreover, among Phases II and III clinical trials for drug development, the time to progressive disease (or PFS) is gradually used as an endpoint to judge effectiveness in clinical trials in severe disease, and is based on anatomic measurement of tumor size.

However, the two judgment endpoints i.e. objective response and time to progressive disease are valuable only when the widely accepted and easy-to-use criteria and rules are established on the basis of tumor burden anatomy. In 1981, the World Health Organization (WHO) first published the criteria for response in tumors, which are mainly used in trials in which tumor response is a primary endpoint. The WHO criteria introduced the overall evaluation concept of tumor burden by measuring and totaling the 2-dimensional of tumor, and judged treatment response by evaluating a change during treatment since baseline. However, in a dozen years after publication of the criteria, the Cooperative Group using the criteria and pharmaceutical companies modified them to adapt new technologies or proposed unclear points in the original literatures, leading to confusion in interpreting the results of a trial. In fact, the application of various response criteria resulted in distinct differences of treatment effects by the same therapeutic method. For those questions, an international work group was established in the mid-19th century to standardize and simplify the criteria for response.

The new criteria, also known as RECIST (Response Evaluation Criteria in Solid tumors) were published in 2000. The initial key features of RECIST included: determination of the minimum measurable lesion; description of lesions to be followed (10 lesions at maximum; 5 lesions per organ at maximum); use of 1 dimension other than 2 dimensions; and overall evaluation of tumor burden. Later, those criteria have been used widely by academic communities, cooperative groups, and the pharmaceutical industry. The initial endpoints of the criteria are objective response or progressive disease. Moreover, the competent authority accepts RECIST as suitable criteria for those evaluation.

2. Purpose of the Guideline:

The Guideline describes a standard method for measuring solid tumors, and clarifies the objective judgment criteria for change of tumor size, used in clinical trials in adults and pediatric patients. It is expected that those criteria will be used in trials in which objective response is used as the primary research endpoint, as well as in trials using steady-state disease evaluation or tumor progression or analyzing time to progression as indicators, as the measurement of all therapeutic effects are based on evaluation of anatomical tumor burden and its change. In this article, the proportion of patients complying with the inclusion criteria are not hypothesized. The patients adopt one trial endpoint that may predict efficacy of one drug or therapeutic regimen: Those definitions are dependent on cancer type in ongoing trials, and on special drugs under investigation. The trial protocol must include an appropriate statistics section, whether the sample size of the trial and the effectiveness parameters on which the inclusion criteria are based are defined. This Guideline provides the definition and criteria to judge tumor response, and also proposes suggestions for report criteria of the results of a clinical trial using tumor response as the trial endpoint.

Although those guidelines may be used in research of malignant cerebral tumors, the criteria for evaluation of response have been published separately in this field. As the international rules for evaluation of lymphoma response have also be published separately, this Guideline is not used in research of malignant lymphoma.

Finally, many oncologists follow patients' malignant disease by multiple imaging investigations in their daily clinical practices, and determine further treatment regimen on both objective and symptom criteria. Those RECIST guidelines may play an important role only when the oncologist during treatment judges as rational.

3. Baseline Tumor Measurement

3.1 Definition

At baseline status, tumor lesion/lymph nodes are divided into measurable and immeasurable categories as follows:

3.1.1 Measurable

**Tumorous lesion:** It must measure accurately to at least one size of not less than the lower limit (of instrument detection) (the maximum diameter in the measuring instrument shall be recorded):

- A 10-mm size is scanned by CT (in a thickness of not less than 5 mm).
- For clinical test, 10 mm is measured by a caliper (a lesion that cannot be measured by a caliper is recorded as immeasurable).
- 20 mm is examined by chest X-ray radiography.

**Malignant lymph nodes**: When evaluated by CT scanning (CT scanning layer thickness is not more than 5 mm as suggested), it is considered as pathological expansion and measurable lesion only when the short axis of lymph nodes must be up to 15 mm. The short axis length is measured and followed in preoperative and subsequent practices. The data for measurement of lymph nodes may be obtained from notes under "Preoperative Documents for Target and Non-target Lesions".

3.1.2 Non-measurable (tumor)

Other all lesions, including small lesions (the maximum diameter is less than 10 mm, or the short axis of pathological lymph nodes ranges between 10 to <15 mm), as well as truly immeasurable lesions. The lesions regarded as truly immeasurable lesions include: Pharmacologically confirmed meningeal diseases, ascites, pleural effusion or pericardial effusion, inflammatory breast diseases, lymph vessel-involved skin or pulmonary, abdominal masses/abdominal organ gigantism, which cannot be measured by reproductive imaging technique.

3.1.3 Special considerations for measurable lesions

Special attention has to be paid to bone lesions, cystic lesions, or lesions previously treated locally:

Bone lesion:

- - Bone scanning, PET scanning, or plain radiography are regarded inadequate for measuring bone lesions. However, those techniques may be used to confirm pharmacologically presence or absence of bone lesions.
  - If the soft tissue part complies with the definition of the above measurable lesions, lytic bone lesion with discernible soft tissues, or mixed acute lytic bone lesion may be evaluated by cross imaging techniques such as CT or MRI, then they are regarded as measurable lesions.
  - Osteogenic lesions are immeasurable.

Cystic lesions:

- - Simple cysts complying with the X-ray definition of the inclusion criteria shall not be regarded as malignant lesions (they are not measurable and are not immeasurable), as they are simple cysts by the definition.
  - Complying with the above definition of measurable lesion, "cystic lesions" characterized by cystic metastasis may be regarded as measurable lesions. However, in case of noncystic lesions present in the body of the same patient, the target lesions are included preferentially.
    Lesions previously treated locally:
  - The lesions located in the previous irradiation area or at the site receiving other local treatment are usually not considered as measurable, unless it has proved that the lesions still persist. The protocol shall explain under what conditions those lesions are considered measurable.

3.2 Specification for measuring methods

3.2.1 Measurement of lesions

For clinical evaluation, the lesions are measured by a caliper (vernier caliper), and all measurements are recorded in metric unit. All baseline evaluations must be carried out as close to treatment as possible, and cannot be as early as 4 weeks ago.

3.2.2. Measuring methods

Each reported lesion shall be described by the same judgment method and technique during the baseline and follow-up periods. Generally they are evaluated by imaging measurements other than clinical examination, unless it is found that the lesions are not suitable for imaging measurements during follow-up.

Clinical lesion: only superficial lesions (such as small subcutaneous nodes) with a diameter of more than 10 mm, as measured by vernier caliper, are considered measurable. For patients with skin lesions, it is suggested that it is recorded by color photographs, in which the proportion for measuring lesion size is annexed. As previously mentioned, when the lesions may be examined clinically or by imaging technique, then it is examined by imaging technique, as imaging evaluation is more objective and may be used for final review of clinical investigation.

Chest X-ray radiography: between chest CT and chest X-ray radiography, chest CT is selected preferentially, especially when progressive disease is used as an important endpoint indicator, as CT scanning is more sensitive in identifying new lesions than X-ray radiography. However, if X-ray radiography visualizes clear border of lesions, surrounded by inflated lung, the lesions are considered measurable.

CT and MRI: Presently, CT is the most efficient and of good repeatability test method used to evaluate lesions. As defined in the Guideline, measurable lesions are based on a layer thickness of not more than 5 mm, if CT scanning is used. When the CT slice thickness is more than 5 mm, the minimum measurable lesion shall be twice the slice thickness. MRI may also be used in some cases (such as whole body scanning).

Ultrasonography: ultrasonography is not suitable to evaluate lesion size, and is not used for measuring methods. Ultrasonography cannot be reproduced completely between two adjacent observations; moreover, the results are dependent on the examiner. Therefore, same technique and same measurement results cannot be assured from one to another examination. If new lesions are found by ultrasonography during research, it is suggested to confirm by CT or MRI. Upon worrying irradiation by CT, MRI may be used instead of examining the lesions.

Endoscopy and laparoscopy: it is not suggested that those techniques are used for efficacy evaluation of tumors. However, they are useful in confirming complete pathological response by biopsy or in determining complete response or relapse after surgical resection.

Tumor marker: Tumor markers cannot be used alone to evaluate objective response of tumors. However, when tumor markers are higher than the normal upper limit, they must be standardized if they are used to judge complete response of a patient. As tumor markers are disease-specific, the description for the measurement technique shall mark the records of baseline measurement for a special disease. The special guidelines for CA-125 changes (in relapse of ovarian carcinoma) and PSA change (in relapse of prostatic carcinoma) have been published. Moreover, the International Group (InterCohort) of Gynecological Oncology has formulated the criteria for CA125 progression, of which all will be used as first-line criteria for objective evaluation of tumors during trials in ovarian carcinoma.

Cytology and histology: if required by the clinical study protocol, those techniques may be used to distinguish partial response and complete response in individual patients (for example, residual benign tumor lesion in the tumor type of germ cell tumor). When it is known that exudate is a potential severe adverse consequence during treatment (such as some paclitaxel chemotherapeutic drugs or angiogenesis inhibitors), to distinguish effective (such as stable disease) and progressive disease, attention must be paid to any neoplastic exudate occurred or exacerbated, as proved cytologically during treatment, even though measurable tumors meet the criteria for effectiveness or stable disease.

4. Tumor Response Evaluation

4.1 Evaluation of all tumors and measurable lesions

In order to evaluate the objective response or possible progression in the future, it is necessary to perform one baseline evaluation of the total burden of all the tumor lesions, as the reference for the measurement result later. In the clinical protocols using the objective response as the primary endpoint, only the patients with measurable lesions at baseline can be enrolled. The definition of measurable lesion is the presence of at least one measurable lesion. And for those trials using progression of disease (time of progression or progression extent on one fixed date) as the primary endpoint, the inclusion criteria of the protocol must make clear whether only the patients with measurable lesions could be enrolled or those without measurable lesions could also be enrolled.

4.2 Baseline records of target and non-target lesions

If there is more than one measurable lesion at baseline assessment, all lesions should be recorded and measured. At a total number of not more than 5 (i.e. not more than 2 lesions for each organ), the target lesion represents all involved organs (i.e. in the patients with one or two involved organs, not more than 2 or 4 lesions are selected as baseline examination lesions).

The target lesion should be selected on the basis of dimension (maximum diameter) and represent all involved organs; and their examination should be well repeatable. If the largest lesion can not be measured repeatedly, another largest lesion of repeatable examination can sometimes be selected again.

Since they are normal tissues and can be examined through the imaging even when there is no tumor metastasis, the lymph nodes should attract a special attention. Only if the minimum diameter is ≥15 mm (at CT examination), the lymph node can be defined as measurable node and even pathological node of target lesion. At baseline, only minimum diameter should be measured. The radiologists should generally, according to the minimum, judge whether the tumor is metastatic to this node. The dimension of node is generally expressed with two-dimensional data of imaging examination (i.e. axial plane for CT; and any of axial, sagittal or coronal plane for MRI). The least value is regarded as minimum diameter. e.g. the abdominal node (dimension 20 mm×30 mm, minimum 20 mm) can be regarded as malignant measurable node. In this case, 20 mm is the measured value of node. The node (diameter 10~15mm) must not be regarded as target lesion. The node (diameter <10 mm) is not a kind of pathological node, and needs not be recorded or further observed.

The sum of diameter of all target lesions (including the maximum diameter of non-nodular lesions and the minimum diameter of nodular lesions) is reported as the sum of baseline diameter. If there is a diameter of lymph node just as mentioned above, the minimum diameter should be included. The sum of baseline diameter will become the reference value for baseline level of disease.

All other lesions (including pathological lymph nodes) can be regarded as non-target lesion, need not be measured, but should be recorded at baseline assessment, e.g. recorded as existing, missing or definitely progressive in few cases. The extensive target lesion can be recorded together with target organ (e.g. pelvic lymph node of massive amplification or extensive liver metastasis).

4.3 Efficacy evaluation criteria

In this section, the criteria for measuring ORR of target lesions of tumors are defined.

4.3.1 Efficacy Evaluation of Target Lesions

Complete response (CR): all target lesions disappear, and the short axis value of any pathological lymph nodes (whether they are target lesions or not) must be <10 mm.

Partial response (PR): using the overall diameter at baseline as reference, the sum of the diameter of all lesions is reduced by at least 30%.

Progressive disease (PD): using the sum of the diameter of minimum lesions (including the sum of the diameter of lesions at baseline, if it is minimum), the sum of the diameter of all target lesions are increased by 20%. Moreover, in addition to the sum of the diameter is increased relatively by 20%, the absolute value of the sum must be increased by at least 5 mm (Note: one or more new lesions occurred may also be considered as progressive disease).

Stable disease (SD): using the sum of the diameter of the target lesions at baseline as reference, the lesion reduction does not meet PR, and using the minimum sum of the diameter of the target lesions during the study, the lesion increase does not meet PD.

4.3.2 Precautions for response evaluation of target lesions

When the target lesions are lymph nodes:

Generally the measured value of its actual short axis (in the same anatomic plane) is recorded, even though all lymph nodes are resided to be in a size of less than 10 mm. This means that, when the target lesions are lymph nodes, even meeting the criteria for response, the sum of the diameter of target lesions is not 0, as the lymph nodes in a short axis value <10 mm are defined as normal lymph nodes. Nodular target lesions may be recorded possibly in design of the Case Report Form, or the collection method of other data. To judge whether there is complete response, it must meet that the short axis value of each node is <10 mm. For PR, SD, and PD, the sum of the diameter of target lesions will include the measured value of the short axis of nodes.

Immeasurable target lesions due to too small size:

For all lesions (nodular and non-nodular lesions) recorded at baseline during the study, their measured values must be recorded in subsequent evaluations, despite too small (such as 2 mm).

However, the signals in CT scanning are sometimes too weak when lesions or lymph nodes are recorded at the critical value, and a radiologist may be possibly unwilling to give an accurate measured value, and reported as "too small and immeasurable".

In such case, it is very important to record the next measured value in the Case Report Form. If a radiologist considers that the lesion may disappear possibly, the measured value may be recorded as 0 mm. If the lesion is present definitely but the signal is too weak, it may be recorded as the default value of 5 mm (this rule is not suitable for lymph nodes, as normal lymph nodes are in a definite size value and are often enwrapped by fatty tissues, such as retroperitoneal lymph nodes. However, if the lymph nodes are present definitely but the signal is too weak and is difficult to measure, they may also be recorded as the default value 5 mm).

The default value of 5 mm is originated from the thickness of CT scanning layers (if the thickness is changed, the default value of 5 mm shall not be changed). The measurements of such lesions (too small and immeasurable) may have no repeatability, and so a default value may prevent any mistaken measurement from which it is evaluated as false healed or false exacerbation. It is emphasized again that if a radiologist may provide an actual measured value, it shall also be recorded as 5 mm, even less than 5 mm.

Ruptured or fused lesions during treatment

When a non-nodular lesion is "ruptured", the sum of the diameter of the target lesions must be calculated by adding up the largest diameter of all fragments. Similarly, when the lesions are fused, the maximum diameter between them may be kept; in this way, it may help obtain the maximum diameter value of each lesion prior to combination. If the lesions are fused completely and are no longer separated, the maximum diameter vector is the maximum diameter of the fused lesion.

4.3.3 Evaluation of non-target lesions

The criteria of response are defined as follows for the tumor of non-target lesion. If some non-target lesions are actually measurable but need not be measured, only a qualitative assessment needs to be made at the time point stipulated in the protocol.

Complete response (CR): All non-target lesions disappear, and the level of tumor marker becomes normal. All lymph nodes are of non-pathological dimension (minimum diameter <10 mm).

Incomplete response/non-progressive disease (non-CR or non PD): There is one or more non-target lesions and/or the level of tumor markers exceeds persistently the normal level.

Progressive disease (PD): a clear progression occurs in existing non-target lesions. Note: PD is also regarded if there is one or more new lesion.

4.3.4 Special precautions for evaluation of non-target lesion progression

Supplementary explanation for defining the progression of non-target lesion: For measurable non-target lesions, a definite progress can be judged on the basis of non-target lesion only when the non-target lesion has deteriorated in an overall intensity necessary for treatment termination, even if the target lesion is assessed as SD or PR. However, the ordinary increase in the dimension of one or more non-target lesions is often insufficient to reach the criteria of PD. Therefore, when the target lesion is assessed as SD or PR, it is nearly very rare to judge the overall the conditions of progression of tumor only according to the change of non-target lesion

When all non-target lesions are unmeasurable, such condition will appear in some Phase III studies if the necessary existence of measurable lesion is not stipulated in the inclusion criteria, and the overall assessment is still made by referring to the above-mentioned criteria because there are unmeasurable data of lesions in this case. Since the deterioration of non-target lesions is difficult to assess (as defined, all non-target lesions should be really unmeasurable), a definite progress is judged according to the non-target lesion and an effective examination method should be established for assessment if the change of non-target lesion increases the overall load of disease in an intensity equivalent to the PD of target lesion. e.g. the increase in tumor load is equivalent to the additional increase of 73% in volume (i.e. equivalent to an increase of 20% in the diameter of measurable lesion); the peritoneal exudates change from trace to massive, with a change in the lesion of lymph duct from localized to extensive (or enough to change the therapeutic method as described in the protocol); or pleural exudates change from trace to massive, with a spreading of involved lymph from original position to distal position (or necessary to change the therapeutic method as described in the protocol). In case of definite progress, PD should be overall judged for this patient at that time point. The objective assessment criteria had better be established for unmeasurable lesion (note: the added criteria should be reliable).

4.3.5 New lesions

Since the emergence of new malignant lesions predicts a PD, it is very important to make some assessment of such new lesions. At present, there are no concrete criteria for imaging examination of lesions, but a new lesion can be found only in a definite way. e.g. PD is not attributed to the difference in imaging technique, the change in imaging morphology or the lesions other than tumor (e.g. some so-called new bone lesions are only the cure of original lesions or the recurrence of original lesions). When the baseline lesion is judged as PR or CR, such treatment is very important, e.g. the necrosis in one liver lesion may be judged as new cystic lesion in the CT report, which does not actually appear.

If a lesion is found during the follow-up but not found at the baseline examination, a new lesion should be judged, and PD is predicted. e.g. when a metastatic lesion is found at skull CT or MRI in the patients with visceral lesion found at baseline examination, such intracranial metastatic lesion is regarded as the basis for PD even though the skull examination is not made at baseline examination.

If a new lesion is not definite for some reasons (e.g. small dimension), a further treatment and follow-up evaluation should be made to determine whether this lesion is new. If a new lesion is verified through the repeated examination, the time of PD should be calculated by starting from the time of its preliminary finding.

For FDG-PET assessment of lesions, an additional examination is generally required for supplementary confirmation, and it is rational to evaluate the conditions of progress by combining the results of FDG-PET examination and supplementary CT examination (especially for new suspicious disease). A new lesion can be definitely judged through the FDG-PET examination according to the following criteria:

The results of baseline FDG-PET examination are negative but the results of subsequent FDG-PET examination are positive at follow-up, indicating a PD;

The baseline FDG-PET examination is not made and the results of subsequent FDG-PET examination are positive;

The new lesion is shown by the positive results of FDG-PET examination at follow-up, which consists with the results of CT examination, verifying a PD.

If the new lesion indicated by the positive results of FDG-PET examination at follow-up is not confirmed by the results of CT examination, CT examination should be made again for confirmation (if such new lesion is confirmed, the time of PD should be calculated by starting from the time of abnormal finding at previous FDG-PET examination).

If the lesion indicated by the positive results of FDG-PET examination at follow-up does not consist with that confirmed by the results of CT examination and the imaging showed no progression of such lesion, PD is not judged.

4.4 Optimal overall efficacy evaluation

The best overall efficacy is evaluated according to the record of best response from the starting to the ending of study, and any necessary condition should be included for confirmation. Since the efficacy response sometimes appears after the completion of treatment, the protocol should specify whether the efficacy evaluation after the completion of treatment is included into the evaluation of best overall efficacy. The protocol should specify how the new treatment before any progress influences the best response response. The best response response are mainly determined according to the following factors: results of target and non-target lesions; manifestations of new lesions; nature of study; requirements of protocol; and assessment criteria of results. To be specific, in the non-randomized trial, the conditions of efficacy response are regarded as primary objective, and the efficacy of PR or CR should be confirmed to determine which is the best overall efficacy response.

4.4.1 Timepoint response

Assuming that an efficacy response appears at an concrete time point of each protocol. The overall efficacy response at each time point in the patient population with measurable diseases at a baseline level is summarized in Table 1.

See Table 2 for the evaluation, if the patients have no measurable lesions (without target lesions).

4.4.2 Explanation for absence of evaluation or non-evaluation

If the imaging or examination of lesions can not be made at a specific time point, this patient is judged as unevaluable at this time point. If only some lesions are evaluated at an evaluation, this patient is generally judged as unevaluable at this time point, unless the lesions of missing evaluation are verified through some evidences as no influence on the evaluation of efficacy response at a specified time point. Such condition probably appears in case of PD. e.g. if there are 3 lesions with a total diameter of 50mm at baseline level (only 2 lesions of which can be evaluated later with a final total diameter of 80mm), PD should be judged at whatever influence of lesions of missing evaluation.

4.4.3 Optimal overall response: all timepoints

Once all materials of patients are obtained, the best overall response can be determined.

Assessment of best overall response not at a necessary confirmation of PR or CR: The best response response means the best response at all time points (e.g. although the efficacy in a patient is judged as SD, PR and PD respectively at the first, second and final cycle, the best overall response is judged as PR). The best overall response can be judged as SD, only after complying with the shortest time (calculated as starting from the baseline level) specified in the protocol. If the stipulated shortest time not met, the best overall response in this patient should be judged according to the subsequent evaluation. e.g. if the efficacy in a patient is judged as SD by not complying with the specified shortest duration of SD, the best overall response is judged as PD; and if this patient is lost of follow-up after the first cycle of SD efficacy, this patient is judged as unevaluable.

Assessment of best overall response at a necessary confirmation of PR or CR: Only when each subject conforms to the criteria of PR or CR stipulated in the protocol which is also confirmed again at the subsequent time point especially stipulated in the protocol (generally after 4 weeks), PR or CR can be judged. In this case, the best overall response is illustrated in Table 3.

4.4.4 Special hints for efficacy evaluation

When the nodular lesion is included into overall assessment of target lesion and this node is shrunk to normal size (<10mm), there should still be scanning report on the size of lesions. To avoid overestimation of the condition reflected on the basis of node size, the measurement results will be recorded, even though the node is normal. Just as mentioned above, for the subjects judged as CR, 0 is not recorded in the CRF.

If it is required to confirm response during trial, optimal efficacy evaluation will be complicated by repeated "immeasurable" timepoints. The analytical plan of study should specify that: while judging the efficacy, such missing data/evaluation can be clearly explained. e.g. in most studies, the response of PR-NE-PR in a certain subject can reflect that the efficacy is confirmed.

When the treatment has to be terminated after the overall deterioration of health conditions but not verified through the objective evidence, a symptomatic progress should be reported. Even after the termination of treatment, the conditions of objective progress should also be assessed as far as possible. The symptomatic deterioration is not the assessment description of objective response, but the reason for termination of treatment. The conditions of objective response in such subjects will be assessed through the conditions of target and non-target lesions as stipulated in Table 1~3.

In special case, an early progress, early death and unevaluable condition can be judged, which should be definitely described in each protocol (according to the interval and cycle of treatment).

In some cases, it is very difficult to distinguish the localized lesions from normal tissues. When CR is judged on the basis of such definition, a biopsy is recommended to be performed before the localized lesion is judged as CR. When a lesion fibrosis or scar formation is considered according to the abnormal results of imaging examination on localized lesions in some subjects, FDG-PET is used as assessment criteria similar to biopsy for confirming the efficacy of CR. In this case, the application of FDG-PET should be described prospectively in the protocol, which is supported through the report on the specialty medical literatures for such conditions. However, to be noteworthy, a false positive result of CR judgment can be caused by the limitation of FDG-PET and biopsy (including their resolution and sensitivity).

**Table 1 Timepoint response: subjects with target lesions (including non-target lesions or not)**

| **Target lesion** | **Non-target lesion** | **New lesions** | **Overall response** |
| --- | --- | --- | --- |
| CR | CR | Non | CR |
| CR | Non CR/non PD | Non | PR |
| CR | Unevaluable | Non | PR |
| PR | Non-progress or incomplete assessment | Non | PR |
| SD | Non-progress or incomplete assessment | Non | SD |
| Incomplete assessment | Non-progress | Non | NE |
| PD | Any conditions | Y or N | PD |
| Any conditions | PD | Y or N | PD |
| Any conditions | Any conditions | Yes | PD |
| CR = complete response | PR = partial response | Stable disease (SD) | PD = progressive disease  NE = Non-evaluable |

**Table 2 Timepoint response - subjects with only non-target lesions**

| **Non-target lesion** | **New lesions** | **Overall response** |
| --- | --- | --- |
| CR | Non | CR |
| Non-CR or non-PD | Non | Non CR/Non PD |
| Incomplete assessment | Non | Unevaluable |
| Indefinite PD | Y or N | PD |
| Any conditions | Yes | PD |

Notes: For non-target lesions, "non CR/non PD" are responses better than SD. Since SD becomes more and more as endpoint index of efficacy evaluation, the efficacy of non-CR or non-PD is stipulated when the conditions of no measurable lesions are not specified.

In case of indefinite progress finding (e.g. very small uncertain new lesions and cystic degeneration or necrotic lesion of original lesions), the treatment can continue until the next assessment of efficacy. If PD is verified at the next assessment, the date of progression should be the date of previous suspicious progression.

**Table 3 Optimal overall response for which it is required to confirm CR and PR**

| **Overall response at the first timepoint** | **Overall response at the subsequent time points** | **Best overall response** |
| --- | --- | --- |
| CR | CR | CR |
| CR | PR | SD, PD or PR^a^ |
| CR | SD | If SD lasts for a sufficient time, SD is judged; and otherwise PD is judged |
| CR | PD | If SD lasts for a sufficient time, SD is judged; and otherwise PD is judged |
| CR | NE | If SD lasts for a sufficient time, SD is judged; and otherwise NE is judged |
| PR | CR | PR |
| PR | PR | PR |
| PR | SD | SD |
| PR | PD | If SD lasts for a sufficient time, SD is judged; and otherwise PD is judged |
| PR | NE | If SD lasts for a sufficient time, SD is judged; and otherwise NE is judged |
| NE | NE | NE |

Notes: CR is complete response, PR is partial response, SD is stable disease, PD is progressive disease, and NE is non-evaluable. Superscript "a": if CR occurs actually at the first timepoint, then for any disease occurred at subsequent timepoints, its response evaluation is still PD at subsequent timepoints (as the disease will appear again after CR), even though the response evaluation reaches PR from baseline. The best response depends on whether SD appears within the minimum treatment interval. However, although CR is judged at the first time point, a small lesion sometimes seems to still appear under the scanning at the subsequent time points; and therefore, the efficacy at the first time point is actually judged as PR but not as CR. In this case, PR (but not CR) should be judged at the first time point, and PR is considered as the best response.

4.5	Frequency for tumor reevaluation

The frequency of tumor re-evaluation during the treatment is determined by the therapeutic protocol, and should conform to the type and schedule of treatment. In the Phase II with an unclear benefit of treatment, it is rational to follow up once in every 6~8 weeks (at the ending point of a cycle), and the time interval can be adjusted in special protocol or case. The protocol should definitely specify which tissue/ position needs to be assessed at baseline level (generally the tissue/ position most probably at a close relation to the type of study tumor) and stipulate the frequency of evaluation repeating. Under the normal conditions, both the target and non-target lesions should be evaluated at each assessment. Under some optional conditions, some non-target lesions can be evaluated less frequently, e.g. the bone scanning can be repeated only when the target disease is confirmed as CR or suspected as progression of bone lesion.

After the completion of treatment, the tumor is re-evaluated according to whether the response rate or the time to occurrence of a certain event (progress/death) is regarded as endpoint of clinical study. If the study endpoint is the time to occurrence of a certain event (e.g. TTP/DFS/PFS), the routine re-evaluation stipulated in the protocol should be performed. Especially in randomized comparative trials, the predefined evaluations should be listed in the schedule (such as 6-8 weeks during treatment, or 3-4 months after treatment), and be not impacted by other factors, such as delayed treatment, administration interval, or any other events that may result in imbalance between treatment arms in selection of disease evaluation times.

4.6	Confirmation of response evaluation/remission period

4.6.1	Confirmation

In the non-randomized clinical study with efficacy as primary study endpoint, the efficacy of PR and CR should be confirmed to ensure that the efficacy is not caused by error in evaluation. Therefore, with the existence of historical data, the results should be explained rationally, but the efficacy in the historical data of these studies should also be confirmed. However, under all other conditions (e.g. in the randomized Phase II or III study or in the studies with SD or PD as primary study endpoint), the efficacy should not be confirmed again, because such efficacy confirmation is useless for explaining the study results. However, after cancelling the requirements of efficacy confirmation, the central review for preventing the offset effect will become more important (especially in the non-blind study).

In case of SD, at least one measurement complies with the specified SD criteria at the minimum time interval (generally not less than 6-8 weeks) after start of the trial.

4.6.2	The overall duration of response

The overall duration of response is calculated as time duration from first conformance with the criteria of CR or PR (whichever is earlier) to first actual recording with recurrence or progression of disease (the minimum measured value recorded in the studies will be used as reference for the calculation of PD). The overall response time is the period from the time when the measurement first meets the CR criteria to the time when disease or progressive disease is first recorded.

4.6.3	Stable disease period

The duration of SD is calculated as time duration from stating treatment to PD (or from randomization in the randomized study), with the minimum sum in the study as reference value (and the sum of baseline level will be used as reference for the calculation of PD, if it the minimum). The clinical relevance during stable disease varies in different studies and for different diseases. If, in a certain trial, the proportion of patients maintaining the minimum stable disease time is used as research endpoint, then the protocol shall describe particularly the minimum time interval between two measurements in the definition of SD.

Notes: The response period and the stable disease period are impacted by follow-up frequency after baseline evaluation. This guideline is not applicable to define the standard frequency of follow-up. The frequency of follow-up should be determined by considering various factors, including type/stage of disease, treatment cycle and standard specifications. However, if a comparison needs to be made between studies, the limitation in the accuracy of these measurement endpoints should be considered.

4.7	PFS/TTP

4.7.1	Phase II clinical trials

This guideline mainly emphasizes the application of objective response as study endpoint in Phase II clinical study. In some cases, the response rate may not be optimum for evaluating the potential anti-tumor activity of new drug/protocol. Under such circumstances, PFS/PPF at the demarcation point may be regarded as an appropriate surrogate indicator to provide original signals of biological activities of a new drug. However, it is obvious that, in a non-controlled trial, those evaluations will be questioned, as seemingly valuable observations may be related with biological factors such as screening of patients, other than the effects of drug intervention. Therefore, a randomized control would better be designed in Phase II clinical study with these variables as study endpoint. However, a non-randomized study is also rational, because some tumors are always of same clinical manifestations (generally always at a poor condition). However under such circumstances, it requires to record carefully response evidences during evaluation of predicted PFS or PPF, due to absence of positive control.

**See the English version for subsequent contents, including Phase III evaluation endpoints, independent evaluation, and result report.**

Attachment 4： Introduction to assessment process of iRECIST for evaluating disease progression

RECIST 1.1 Progress assessment and decision making

For the participant determined to have radiologic evidence of PD by the investigator according to RECIST 1.1, the investigator will decide whether to continue to have the participant use the study therapy until repeated imaging results are obtained by using the management-related iRECIST for the participant. The decision of the investigator should be based on the overall clinical conditions of the participants.

Clinical stability is defined as follows:

- Absence of clinical symptoms or signs indicating clinically significant disease progression.
- No decrease in ECOG performance status
- There is no need for intensive therapies including intensive pain relief, radiotherapy or other palliative therapies.

The participants considered to have unstable clinical disease should stop the investigation and therapy, and confirm PD with iRECIST without receiving tumor imaging reexamination.

If the investigator decides to continue the treatment, the subject is allowed to continue to receive the study therapy, the tumor evaluation should be reexamined after 4 to 8 weeks, and the disease progression should be confirmed with iRECIST according to the investigator's assessment.

New response classifications including the immune unconfirmed progressive disease (iUPD) and immune confirmed progressive disease (iCPD) are defined in iRECIST. For the purpose of iRECIST assessment, the first visit showing disease progression based on R RECIST 1.1 will be classified as visit (overall) response of iUPD regardless of which factor is responsible for disease progression. The target and non target lesions identified by RECIST 1.1 in the visit during the baseline period will be evaluated routinely.

New lesions will be classified as measurable or unmeasurable lesions by using the threshold size and rules the same to those used in the baseline lesion assessment of RECIST 1.1. Up to 5 lesions (up to 2 lesions per organ) may be selected as new targets from the new measurable lesions. The sum of diameters of these lesions will be calculated and distinguished from that of the target lesions at baseline. All other new lesions will be subject to non-target qualitative follow-up and examination as new lesions. Evaluation at confirmatory imaging examination

The participant will be classified as confirmed disease progression (overall response is to be iCPD) or shows ongoing and unconfirmed progress (overall response is to be iUPD) or shows stable or response of disease (ISD / IPR / ICR).

Confirmation of progressive disease

The progression of the disease is considered to be confirmed and the overall response is believed to be iCPD if any of the following conditions occur:

- Any factors forming basis of the initial iUPD shows deterioration.
- For the target lesion, the deterioration refers to a further increase in the total diameter≥ 5 mm compared to that at previous time point of iUPD.
- For non target lesion, the deterioration means any significant increase in the total number of lesions compared to those at previous time points of iUPD; it isn't required to meet the "clear" criteria of RECIST 1.1.
- For new lesion, the deterioration indicates any of the following conditions:
- The total diameter of new lesions increases by 5 mm or above compared to previous time points
- Visible growth of new non-target lesions
- Occurrence of additional new lesion
- Any new factors which can make assessment results of RECIST 1.1 become PD

Ongoing *iUPD*

The disease progression is not considered to be confirmed and the overall response remains iUPD if the following conditions occur:

- No evidence of the disease progression above mentioned occurs, and
- The total diameter of target lesions (initial target lesions) is still greater than initial PD threshold (RECIST 1.1).

Additional imaging examination should be arranged during 4-8 cycles at the time of found iUPD in the imaging examination This may be equivalent to the next visit in former visit plan. Subsequent confirmatory imaging assessments progressing in the same way may have outcomes of iCPD, iUPD and iSD/iPR/iCR.

iUPD Recovery

If the following conditions occurs, the disease progression is not considered to be confirmed and the overall responses areiSD/iPR/iCR:

- No evidence of disease progression above mentioned appears, and
- The total diameter of target lesions (initial target lesions) is not greater than the initial PD threshold.

The response was classified as either iSD or iPR (depending on the sum of target lesion diameters), or ICR if all lesions regresses.

In this case, the initial iUPD is considered as a pseudo-progression, and the level of suspicion on the progress is the "reset". This means that it is reclassified as iUPD according to iRECIST regardless of specific time of next visit to show radiographic progression, and the response condition is evaluated as the reconfirmation process prior to iCPD.

Management after confirmatory imaging examination

If the imaging examination is repeated, no PD is confirmed according to iRECISTand the investigator's assessment, and the clinical conditions of the participant continue to be stable, then the investigational therapy can be continued, and regular imaging examination plan should be followed. If PD is confirmed, the subject will be discontinued from the study therapy.

Discovery of disease progression during follow-up visit after pseudo progression regresses

Regression after the pseudo progression (i.e. up to iSD/iPR/iCR), any of the following events is found to have iUPD:

- Target lesions
- The sum of diameters after the first or previous pseudo progression regress (increase from minimum value satisfies ≥ 20% and ≥ 5 mm) hits the threshold value of PD. The minimum value is always the minimum value of sum of diameters observed before or after the pseudo-progression event throughout the test period.
- Non-target lesions
- If the non target lesion has never shown clear progression, the clear progression firstly shown will cause iUPD.
- If the non-target lesion previously showed clear progression and the progression did not regress, any further significant growth of the non-target lesion (as a whole) will cause iUPD.
- New lesions
- New lesion appears initially
- Occurrence of additional new lesion
- New lesions found before showed that the sum of diameters of new lesions increased by 5 mm or above from its minimum value
- Non-target lesions previously found showing any significant growth

If any of the abovementioned events occur and the total response at the visit is iUPD, the assessment process of iUPD will be repeated (see the assessment at confirmatory imaging abovementioned). The disease progression must be confirmed prior to the occurrence of ICPD.

The decision-making process is the same as the confirmation process for iUPD of initial PD with one exception,in which if a new lesion appears at the previous iUPD and the load of new lesion increases from its minimum value (the sum of diameters for new lesion increases from its minimum value ≥ 5 mm), iUPD fails to regress to iSD or iPR. It will remain iUPD until the load of new lesion is reduced to iSD or iPR or until confirmed factors causes iCPD.

For more information about iRECIST, see [Seymour, L.et al2017], the publication of iRECIST.

Attachment 5： Recommendations for alternative therapy of hypothyroidism

Levothyroxine Sodium (common brandname, Euthyrox) is recommended for patients developing the investigational therapy-related hypothyroidism.

The dose recommended in this Appendix is a general rule. A patient's personal daily dose should be determined from the results of laboratory tests and clinical examinations. As T4 and fT4 levels in many patients would be increased, the baseline concentration of serum thyroid stimulating hormone is a reliable basis to determine a therapeutic method. Generally, thyroid hormone therapy should be started from a low dose, and increased gradually in dose every 2-4 weeks, until a sufficient dose. Generally speaking, patients with hypothyroidism requires a lifelong oral administration of the drug.

A special attention should be exercised in the initiation phase of thyroid hormone therapy in elderly patients, or patients with coronary heart disease or chronic hypothyroidism; a low initial dose (such as 12.5μg/d) should be selected and the oral dose should be increased slowly in a long period of time (such as increase of 12.5μg/d every 2 weeks).

If the final maintenance dose of a patient is lower than the optimal dose, then it cannot correct completely its TSH level.

Experiences have demonstrated that, a low dose is effective for patients with a light body weight or patients with macronodular goiter.

Levothyroxine Sodium should be taken under a fasted state half an hour prior to breakfast, one daily dose with an adequate amount of liquid (such as half glass of water).

**Recommended Doses**

|  | **Dose** | **50 μg tablets** | **100 μg tablets** | **Administration method** |
| --- | --- | --- | --- | --- |
| Initial dose  (After the initial dose, increase 25-50 μg every 2-4 weeks, until the maintenance dose) | 25-50 μg | ½-1 tablet | ¼-1/2 tablet | QD |
| Maintenance dose | 100-200 μg | 2-4 tablets | 1-2 tablets | QD |

For secondary hypothyroidism, its cause must be defined prior to alternative therapy of the drug; glucocorticoid may be administered as supplementary therapy if necessary. Once the Levothyroxine therapy is determined, it is suggested to adjust its dose, based on the patient's clinical responses and laboratory test results if it switches to another drug.

For details, please refer to the package insert of the respective product.

Attachment 6： Precautions for allergies

**Required devices**

- Tourniquet
- Oxygen
- According to the practice standard, subcutaneous, intravenous, and/or tracheal epinephrine are ready
- Antihistamines
- Corticosteroids
- Solution for intravenous infusion,infusion tube, catheter, and adhesive tape

**Procedures**

If it is suspected that allergies occur during drug infusion in the study, the following procedure should be implemented:

- The investigational product is not infused.
- Bind a tourniquet at the proximal end of the injection site, to delay systematic absorption of the investigational product. Do not impede arterial bloodstream of limbs.
- Maintain airway patent.
- Antihistamine, epinephrine, or other drugs are administered, as specifically determined by the doctor, aiming at the patient's condition.
- The patient is under continued observation and observation results are recorded.
