# Supplementary material for: Toripalimab plus chemotherapy for first line treatment of advanced non-small cell lung cancer (CHOICE-01): final OS and biomarker exploration of a randomized, double-blind, phase 3 trial
Source: Signal Transduct Target Ther. 2024 Dec 24;9:369. doi: 10.1038/s41392-024-02087-6 (PMC11666711; doi:10.1038/s41392-024-02087-6)
Supplement: Supplementary file 3 — Supplement 3 [file 41392_2024_2087_MOESM3_ESM.pdf]

A Randomized, Double-blinded, Placebo-controlled, Multicenter, Phase III Study to Evaluate the Efficacy and Safety of Toripalimab Injection (JS001) or Placebo in Combination with Standard First-line Chemotherapy in Patients with Untreated Advanced Non-Small Cell Lung Cancer (NSCLC)

Protocol Number: JS001-019-III-NSCLC
Sponsor of Clinical Trial: Shanghai Junshi Biosciences Co., Ltd.

Statistical Analysis Plan

Version No.: 2.0
Date: September 18, 2021

Statistical Analysis Plan Signature Page

Study Title: A Randomized, Double-blinded, Placebo-controlled, Multicenter, Phase III Study to Evaluate the Efficacy and Safety of Toripalimab Injection (JS001) or Placebo in Combination with Standard First-line Chemotherapy in Patients with Untreated Advanced Non-Small Cell Lung Cancer (NSCLC)

Protocol No.: **JS001-019-III-NSCLC**

Version No./Date: **2.0/ September 18, 2021**

YU, Wenbo	Yu, Wenbo	September 18, 2021
Associate Director, Biostatistics<br>Shanghai Junshi Biosciences Co., Ltd.	Signature	Date
TANG, Xiongwen	[signature]	18 Sep 2021
Director, Biostatistics<br>Shanghai Junshi Biosciences Co., Ltd.	Signature	Date
Wang, Yanyan	Yanyan Wang	18/Sep/2021
Director, Medical Science<br>Shanghai Junshi Biosciences Co., Ltd.	Signature	Date

Table of Contents

1 Introduction 7
2 Study Objective 7
2.1 Primary objectives: 7
2.2 Secondary objectives: 7
2.3 Exploratory objectives: 7
3 Study design 8
3.1 Overall design 8
3.2 Drug Administration 10
3.3 Study Schema 10
4 Study Endpoints 23
4.1 Efficacy endpoint 23
4.1.1 Primary efficacy endpoint 23
4.1.2 Secondary efficacy endpoints 23
4.1.3 Exploratory efficacy endpoints 24
4.2 Safety endpoints 26
4.3 Immunogenicity 27
4.4 Biomarker 27
5 Sample Size Estimation 27
6 Interim Analyses 28
6.1 Post-PFS-Interim-Analysis Changes 30
7 Analysis Population 30
8 Statistical Analysis 30
8.1 General principles 31
8.2 Conventions for data processing 32
8.2.1 Handling of missing data 32
8.2.2 Definition of baseline data 34
8.2.3 Handling of overall tumor response dates 34
8.2.4 Handling of data after cut-off date 35
8.2.5 Handling of PFS data censoring with new anti-tumor therapy 35
8.2.6 Handling of use of incorrect stratification factors 35
8.3 Study population 35
8.3.1 Subject disposition 35
8.3.2 Protocol Deviation 36
8.4 Demographic and baseline characteristics 37
8.4.1 Demographics 37
8.4.2 Smoking history and alcohol use history 37
8.4.3 Medical history of non-small cell lung cancer (NSCLC) 37
8.4.4 Testing of tumor cell genes and PD-L1 37
8.4.5 Baseline tumor assessments 38
8.4.6 Virology at baseline 38
8.4.7 Past medical history: 38
8.4.8 Prior anti-NSCLC treatment history 38
8.5 Prior/concomitant medications, non-drug therapies, and new anti-tumor therapies

.... 38
8.6 Efficacy analysis.. 39
8.6.1 Primary efficacy analysis 39
8.6.2 Secondary efficacy analysis 41
8.6.3 Exploratory efficacy analysis 43
8.7 Safety analysis... 43
8.7.1 Drug exposure and compliance 43
8.7.2 Adverse event... 44
8.7.3 Laboratory test.. 47
8.7.4 Vital signs and physical examination 49
8.7.5 Electrocardiogram 49
8.7.6 Echocardiography.. 50
8.7.7 Pulmonary function test 50
8.7.8 ECOG performance status.. 50
9 References 50
10 Attachments.. 51
10.1 Cut-off date rule for SDTM.. 51
10.2 Mapping rules for tumor assessment visits 53
10.3 Calculation rule for derived AE dataset 54
11 Version Control 58

List of Abbreviations

Abbreviations and Terminologies	Definition
ADA	Anti-drug antibody
AE	adverse event
ALK	Anaplastic lymphoma kinase
ALT	Alanine aminotransferase
AST	Aspartate aminotransferase
BIRC	Blinded Independent Review Committee
BSA	Body surface area
CFDA	China Food and Drug Administration
CI	Confidence interval
CM	Concomitant medications
CR	Complete response
CRF	Case Report Form
CTCAE	Common Terminology Criteria for Adverse Events
DBL	Database lock
DCR	Disease control rate
DOR	Duration of response
ECOG	Eastern Cooperative Oncology Group
EGFR	Epidermal growth factor receptor
FAS	Full analysis set
GCP	Good Clinical Practice
HR	Hazard ratio
IDMC	Independent Data Monitoring Committee
INV	Investigator
ITT	Intention-to-treat principle
IWRS	Interactive web randomization system
MedDRA	Medical Dictionary for Regulatory Activities
NE	Not evaluable
NGS	Next-generation sequencing
NSCLC	Non-small cell lung cancer
ORR	Objective response rate
OS	Overall survival
PBMC	Peripheral blood mononuclear cell

Abbreviations and Terminologies	Definition
PD	Progressive disease
PFS	Progression-free survival
PPS	Per protocol analysis set
PR	Partial response
PT	Preferred term
RECIST	Response Evaluation Criteria in Solid Tumors
SAE	Serious adverse event
SAP	Statistical Analysis Plan
SD	Stable disease
SOC	System Organ Class
SOP	Standard operating procedure
SS	Safety analysis set
TEAE	Treatment-emergent adverse events
TTR	Time to objective response
WES	Whole-exome sequencing
WHO	World Health Organization

1 Introduction

This statistical analysis plan (SAP) is generated by the statistical vendor and the Sponsor in accordance with the protocol of "A Randomized, Double-blinded, Placebo-controlled, Multicenter, Phase III Study to Evaluate the Efficacy and Safety of Toripalimab Injection (JS001) or Placebo in Combination with Standard First-Line Chemotherapy in Patients with Untreated Advanced Non-Small Cell Lung Cancer (NSCLC)" (Protocol No.: JS001-019-III-NSCLC, version: 4.0, version date: June 24, 2020), Good Clinical Practice (GCP) issued by the National Medical Products Administration (NMPA), the Guideline on Structure and Content of Clinical Study Reports for Chemical Drugs, and the Technical Guideline of Biostatistics for Clinical Trials of Chemical Drug and Biological Product. The SAP specifies the content and format of the interim analysis and the final analysis, and will be finalized and approved prior to database lock (DBL) for the interim analysis and final analysis, respectively.

2 Study Objective

2.1 Primary objectives:

- To evaluate the progression-free survival (PFS) of toripalimab injection (JS001) versus placebo in combination with standard first-line chemotherapy in untreated advanced non-small cell lung cancer (NSCLC)

2.2 Secondary objectives:

- To evaluate the overall survival (OS), objective response rate (ORR), duration of response (DOR), disease control rate (DCR), and time to disease response (TTR) per RECIST 1.1 of toripalimab versus placebo in combination with standard first-line chemotherapy in untreated advanced NSCLC
- To evaluate the safety and tolerability of toripalimab versus placebo in combination with standard first-line chemotherapy in untreated advanced NSCLC

2.3 Exploratory objectives:

- To evaluate PFS, ORR, DOR, DCR and TTR per iRECIST of toripalimab versus placebo in combination with standard first-line chemotherapy in untreated advanced NSCLC
- To evaluate the immunogenicity of toripalimab, and explore the potential relationship of its immunogenic response with safety and efficacy

- To explore subpopulation with best efficacy predictions through biomarker analysis (including but not limited to PBMCs, PD-L1, WES, RNA-Seq, etc.)

3 Study design

3.1 Overall design

This is a randomized, double-blinded, placebo-controlled, multicenter, Phase III study designed to evaluate the efficacy and safety of toripalimab or placebo in combination with standard first-line chemotherapy in patients with untreated advanced NSCLC.

Approximately 450 subjects with advanced NSCLC without sensitizing EGFR mutation or ALK fusion will be randomized in a 2:1 ratio to receive toripalimab in combination with first-line standard chemotherapy in the test group or placebo in combination with first-line standard chemotherapy in the control group. Randomization will be stratified by the following factors:

- PD-L1 expression (tumor cells (TC) ≥ 1% vs TC < 1%);
  Note: Patients who are not evaluable for PD-L1 tumor expressionwill be included in the TC < 1% group.
- Smoking status (regular vs nonsmoking or accasional smoking);
  Note: Smoking status is shown by smoking index, smoking index = number of cigarettes smoked per day × number of years of smoking, and regular smoking is defined as ≥ 400 cigarette-years.
- Pathological type (squamous vs non-squamous);
  Note: Patients with adenosquamous cell carcinoma type will be included in the squamous cell carcinoma subgroup and treated with nab-paclitaxel plus carboplatin chemotherapy.

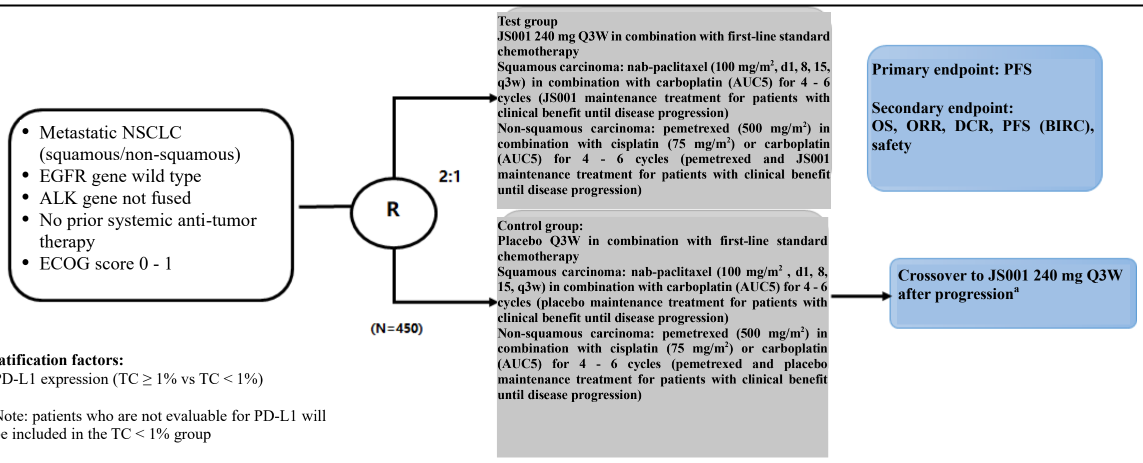

Stratification factors:

- PD-L1 expression (TC ≥ 1% vs TC < 1%)

  Note: patients who are not evaluable for PD-L1 will be included in the TC < 1% group

- Smoking status (frequent vs nonsmoking or little smoking)

- Pathological type (squamous vs non-squamous)

a: Patients in the control group are allowed to cross over to the test group after disease progression to continue to receive the study drug JS001, but they should be reassessed for inclusion eligibility

Figure 1 Study flowchart

3.2 Drug Administration

Study drug (toripalimab/placebo): 240 mg toripalimab / 6 mL placebo will be administered on Day 1 of each 21-day cycle. The treatment will continue until the subject meets the discontinuation criteria, i.e., documented disease progression, unacceptable adverse events (AEs), ineligible for continued treatment in the opinion of the investigator, withdrawal of consent by the subject, cumulative toripalimab treatment received for a total of 2 years, or other reasons specified in the protocol.

First-line standard chemotherapy:

Body surface area (BSA) defined by Dubois formula: BSA (m^2) = 0.20247 × height $(m)^{0.725}$ × weight $(kg)^{0.425}$.

Subjects with squamous NSCLC:

- Nab-paclitaxel + carboplatin: albumin-bound (nab)-paclitaxel 100 mg/m^2 by intravenous infusion on Day 1, 8, 15 (the nab-paclitaxel administration on Day 15 is at the discretion of the investigator); carboplatin AUC 5 on Day 1 in a 21-day cycle with no more than 4 – 6 cycles.

Subjects with non-squamous NSCLC:

- Cisplatin or carboplatin is determined by the researcher, who will choose one of the following two regimens:
  - Pemetrexed + cisplatin: pemetrexed 500 mg/m^2 and cisplatin 75 mg/m^2 will be administrated by IV infusion on Day 1 of each 21-day cycle for a maximum of 4 - 6 cycles.
  - Pemetrexed + carboplatin: pemetrexed 500 mg/m^2 and carboplatin AUC 5 will be administered by IV infusion on Day 1 of each 21-day cycle for a maximum of 4 - 6 cycles.
- Patients whose disease has not progressed after 4 - 6 cycles of treatment may continue to receive pemetrexed alone as maintenance therapy.
- The administration of cisplatin or carboplatin is at the discretion of the investigator.

Continuation of toripalimab/placebo after disease progression: extensive evidence indicates that a small number of subjects treated with immunotherapy may still receive clinical benefit after the initial evidence of disease progression (PD) is observed. The criteria for continued treatment and assessment of subjects who discontinue treatment after disease progression are detailed in Section 5.14 of the protocol.

3.3 Study Schema

The study consists of a screening period, a treatment period (21-day treatment cycles for up to 2 years), a safety follow-up period, and a survival follow-up period. Subjects will be unblinded after determination of PD according to RECIST 1.1. If subjects are treated with placebo and meets the requirements of the crossover, they will be switched to toripalimab treatment. The study schema and procedures are summarized in **Table 1** and **Table 2**.

Table 1. Study Schema - Treatment Phase

	Screening period (Baseline)		Treatment period[2] Every 21 days (± 3 days)															End of treatment visit[27]	Subsequent treatment visit[27]		
Treatment cycle			Cycle 1			Cycle 2			Cycle 3			Cycle 4			Cycle 5 and beyond				Safety follow-up	Follow-up visit	Survival follow-up
Time window (days)	≥28 days prior to randomization	≥14 days prior to randomization	C1D1	C1D8±1	C1D15±1	C2D1±3	C2D8±1	C2D15±1	C3D1±3	C3D8±1	C3D15±1	C4D1±3	C4D8±1	C4D15±1	CxD1±3	CxD8±1	CxD15±1	Treatment discontinuation ±3	30 days ± 7 days after last dose	For subjects who discontinue treatment for reasons other than disease progression	Every 3 months ± 14 days
General assessment																					
Informed consent[1]	×																				
Informed consent form for optional biomarker subgroup studies	×																				
Demographic information[3]	×																				
Inclusion/exclusion criteria	×																				
Past medical history	×																				
Concomitant medications[4]	×	×	×			×			×			×			×			×	×		
Prior tumor history[5]	×																				
Subject enrollment[6]			×																		
Subsequent anti-tumor therapy																		×	×	×	×
Survival status follow-up																				×	×
Clinical assessment																					

	Screening period (Baseline)		Treatment period[2] Every 21 days (± 3 days)															End of treatment visit[27]	Subsequent treatment visit[27]		
Treatment cycle			Cycle 1			Cycle 2			Cycle 3			Cycle 4			Cycle 5 and beyond				Safety follow-up	Follow-up visit	Survival follow-up
Time window (days)	≥28 days prior to randomization	≥14 days prior to randomization	C1D1	C1D8±1	C1D15±1	C2D1±3	C2D8±1	C2D15±1	C3D1±3	C3D8±1	C3D15±1	C4D1±3	C4D8±1	C4D15±1	CxD1±3	CxD8±1	CxD15±1	Treatment discontinuation ±3	30 days ± 7 days after last dose	For subjects who discontinue treatment for reasons other than disease progression	Every 3 months ± 14 days
Collection of adverse events (AEs)[7]			×			×			×						×			×	×		
Complete physical examination[8]	×																				
Targeted physical examination[9]			×			×			×			×			×			×	×		
Body height and weight		×	×	×	×	×	×	×	×	×	×	×	×	×	×	×	×	×	×		
Vital signs	×		×			×			×			×			×			×	×		
12-lead electrocardiogram		×				×			×			×			×			×	×		
Echocardiography[10]	×								×						×			×			
Pulmonary function test[11]	×																				
ECOG score	×		×			×			×			×			×			×	×		
Laboratory tests (local laboratory)[12]																					
Serum pregnancy test (if applicable)[13]		×				×			×			×			×			×	×		
Coagulation function[14]		×				×			×			×			×			×	×		
Hematology[15]		×		×	×	×	×	×	×	×	×	×	×	×	×	×	×	×	×		
Blood chemistry[16]		×				×			×			×			×			×	×		

	Screening period (Baseline)		Treatment period[2] Every 21 days (± 3 days)															End of treatment visit[27]	Subsequent treatment visit[27]		
Treatment cycle			Cycle 1			Cycle 2			Cycle 3			Cycle 4			Cycle 5 and beyond				Safety follow-up	Follow-up visit	Survival follow-up
Time window (days)	≥28 days prior to randomization	≥14 days prior to randomization	C1D1	C1D 8±1	C1D1 5±1	C2D 1±3	C2D 8±1	C2D1 5±1	C3D 1±3	C3D 8±1	C3D1 5±1	C4D 1±3	C4D 8±1	C4D1 5±1	CxD 1±3	CxD 8±1	CxD1 5±1	Treatment discontinuation ±3	30 days ± 7 days after last dose	For subjects who discontinue treatment for reasons other than disease progression	Every 3 months ± 14 days
Creatinine clearance[17]		×				×			×			×			×			×	×		
Urinalysis[18]		×				×			×			×			×			×	×		
Stool routine + occult blood[18]		×				×			×			×			×			×	×		
Virological test[19]	×																				
Thyroid function[20]		×				×			×			×			×			×	×		
Laboratory tests (central laboratory)																					
Anti-drug antibody (ADA) and trough serum concentrations[21]			×												×			×			
Peripheral blood biomarker testing[22]			×						×						×			×			
Tumor tissue collection																					
PDL1 and TMB analysis for fresh tissue biopsy[23]	×																				
Tissue samples are to be collected in the event of partial response or progression of disease, but limited to patients who have signed informed consent for the study in optional biomarker subgroup																					
Testing for EGFR mutation and ALK fusion[24]	×																				
Tumor assessment																					

	Screening period (Baseline)		Treatment period[2] Every 21 days (± 3 days)															End of treatment visit[27]	Subsequent treatment visit[27]		
Treatment cycle			Cycle 1			Cycle 2			Cycle 3			Cycle 4			Cycle 5 and beyond				Safety follow-up	Follow-up visit	Survival follow-up
Time window (days)	≥28 days prior to randomization	≥14 days prior to randomization	C1D1	C1D8±1	C1D15±1	C2D1±3	C2D8±1	C2D15±1	C3D1±3	C3D8±1	C3D15±1	C4D1±3	C4D8±1	C4D15±1	CxD1±3	CxD8±1	CxD15±1	Treatment discontinuation ±3	30 days ± 7 days after last dose	For subjects who discontinue treatment for reasons other than disease progression	Every 3 months ± 14 days
Tumor assessments[25]	×								×						×			×		×	
Administration of protocol-specified drugs[26]																					
Toripalimab or placebo			×			×			×			×			×						
Pemetrexed			×			×			×			×			×						
Cisplatin or carboplatin[28]			×			×			×			×									
Nab-paclitaxel[29]			×	×	×	×	×	×	×	×	×	×	×	×	×	×	×				

Note:

1. Informed consent form must be obtained prior to conduct of study-specific procedures;
2. Every 3 weeks is a treatment cycle. For subjects who continue to receive treatment after progression, treatment cycles remain every three week;
3. Demographic data: including date of birth, gender, self-reported ethnicity;
4. All medications taken from 30 days prior to the first dose until the safety follow-up visit must be recorded in the case report form, including the generic name and daily dose of the drug, reason for use of the drug, the start date and the end date;
5. Prior treatment history for NSCLC including the date of tumor diagnosis, the start/end date of the previous treatment regimen, the best treatment assessment, and the date of the disease progression; radiotherapy history should include the start/end date, and the site of radiotherapy;

6. Subject enrollment: after confirming that the subject is eligible, it is required to log into the RTSM system for randomization within 3 (including) days prior to the first dose;
7. Adverse events (AEs) should be collected from the start of the first dose to 30 days after the last dose or start of new anti-tumor therapy, whichever occurs first. Only serious adverse events will be reported within the period from signing of the informed consent form until the first dose;
8. Physical examination: including head, eyes, ears, nose, throat, neck, heart, chest (including lungs), abdomen, extremities, skin, lymph nodes, nervous system and patient's general condition;
9. For cycles in which a complete physical examination is not required in accordance with the study flowchart, the investigator will perform a specialized physical examination as clinically applicable prior to the study treatment. New clinically significant abnormal findings should be recorded as adverse events;
10. Echocardiography will be used to examine left ventricular ejection fraction. From C1D1 onwards, the examination is carried out every 2 cycles for the first 12 cycles and every 3 cycles thereafter;
11. Pulmonary function tests: including maximum vital capacity, maximum mid-expiratory flow (FEF25-75), maximum peak expiratory flow (PEF), forced expiratory volume in 1 second, and diffusing lung capacity for carbon monoxide (DLCO), are performed at screening period;
12. With the exception of C1D1, all laboratory tests during the treatment period must be completed before each dose. Hematology, coagulation function, and blood chemistry (including creatinine clearance) will be performed only within 3 days before dosing, and other laboratory tests within 7 days prior to dosing. Only after laboratory test results meet the criteria for continued medication as judged by the investigator, can the drug be administered;
13. For women of childbearing potential, a serum pregnancy test should be performed within 72 hours prior to the first dose of study treatment. Then, the pregnancy test will be performed within 7 days prior to treatment in each cycle until the safety follow-up visit.
14. Coagulation function includes: international normalized ratio (INR), prothrombin time (PT), activated partial thrombin time (aPTT); screening examinations should be completed within 7 days prior to the first dose.
15. Hematology: red blood cell count, hemoglobin, hematocrit, white blood cell count and differential (neutrophils, lymphocytes, eosinophils, monocytes, basophils), and platelet count; screening examinations should be completed within 7 days prior to the first dose. If neutrophils $\leq 1.0 \times 10^9$/L or platelets $\leq 50 \times 10^9$/L, reexamination frequency (once/2 - 3 days) should be increased; if dose interruption or dose modification due to hematologic toxicity occurs, hematology should be repeated weekly until it becomes normal; visit procedures on Day 8 and 15 are only applicable for subjects treated with nab-paclitaxel and should be completed within 2 days prior to the treatment.
16. Blood chemistry includes total protein, albumin, globulin, blood glucose, total cholesterol, low density lipoprotein, high density lipoprotein, triglycerides, urea/urea nitrogen, creatinine, alkaline phosphatase, lactate dehydrogenase, creatine kinase, creatine kinase isoenzyme, total bilirubin, direct bilirubin, indirect bilirubin, AST, ALT, calcium, phosphorus, magnesium, potassium, sodium, chloride, serum amylase, and uric acid; screening examinations should be completed within 7 days prior to the first dose. A 3-fold increase in ALT or AST or a 2-fold increase from the baseline abnormal value during the study requires an increase in the examination frequency (1 - 2 times/week is recommended);

17. Creatinine clearance (Ccr) is calculated as: Ccr = (140 - age) × weight (kg) / [72 (kg) × Scr (mg/dl)] or Ccr = [(140 - age) × weight (kg)] / [0.814 × Scr(μmol/L)]; for units to be noted in the calculation of creatinine clearance, results should be multiplied with 0.85 for females
18. Urinalysis includes specific gravity, PH, urine glucose, protein, casts, ketone bodies, blood cells (white blood cells urine, red blood cells urine); if the test result of urine protein is ++ or above or is abnormal and clinically significant as judged by the physician, a 24-hour urine protein quantitative measurement is required; stool routine examination includes: stool color and shape, red blood cells, white blood cells, and occult blood;
19. Virological test includes hepatitis testing (HBV DNA copy number is required if HBsAg is positive and/or HBcAb is positive), HCV antibody, and HIV antibody; HCV RNA is required when HCV antibody is positive
20. Thyroid function test includes: thyroid stimulating hormone (TSH), serum tri-iodothyronine free (FT3), and serum thyroxine free (FT4), and it should be performed once before administration and at the end of treatment visit in each cycle; if there is a clinically significant change in thyroid function, the endocrinology department is recommended to consult and perform relevant pituitary function tests;
21. The collection should be conducted before the first dose of the study treatment period and within 60 min before dosing every 4 cycles in the first year, and within 60 min before dosing every 8 cycles thereafter;
22. Peripheral blood biomarker sampling and testing are required only for subjects in the participating institution of biomarker studies (the collection of this sample will be performed in full compliance with the patient's voluntary provision except for the first blood sampling). Collections will be performed prior to the first dose after randomization and at each subsequent radiographic assessment until disease progression. Peripheral venous blood of 8 mL will be collected for biomarker analysis at each time point.
23. All subjects must provide tumor tissue samples for PD-L1 testing by the central laboratory before enrollment, and retesting may be required once if the test results cannot be assessed due to various conditions; TMB will be performed using next-generation sequencing (NGS) whole-exome sequencing (WES).
24. For patients with unknown EGFR and ALK status, local laboratory reports are acceptable (but a well-validated, NMPA-approved kit must be used), and the testing for EGFR and ALK status is not mandatory for patients with squamous carcinoma
25. Tumors are evaluated according to RECIST v1.1 and iRECIST. Screening tumor assessments must be performed within 4 weeks prior to the first dose, and enhanced CT scans of the chest, abdomen (including liver and adrenal glands), pelvis (unless contraindicated, oral/IV contrast is required) should be performed; if clinically indicated, any other known or suspected sites of disease, such as a head MRI, bone scan, or CT scan of the neck, may be examined using appropriate methods; for tumor imaging conducted prior to signing the informed consent form by a patient in routine diagnosis and treatment, if it is performed within 4 weeks prior to enrollment and at the study site, repetition is not required. The same imaging modality should be used at baseline and subsequent assessments which should be performed by the same investigator as possible. Tumor assessment cycles should be calculated from C1D1, i.e., every 6 weeks (7 days window) for the first 12 months and every 9 weeks (7 days window) after 12 months, and unaffected by discontinuation. An additional tumor assessment should be performed for patients suspected of disease progression prior to the start of next scheduled tumor assessment.

26. The study treatment consists of two periods: induction treatment period and maintenance treatment period. The induction treatment period will include 4 - 6 courses of treatment. A course of treatment for the induction treatment period is 21 (± 3 days) days. Enrolled patients will receive a fixed dose of 240 mg toripalimab/placebo intravenously on the first day of each course, followed by pemetrexed + platinum or nab-paclitaxel combined with carboplatin chemotherapy, until occurrence of the following (whichever occurs first): 4 - 6 courses of treatment completed; disease progression. After the induction treatment period, patients with non-squamous NSCLC will continue to receive maintenance treatment, for which the course of treatment will still be 21 (± 3 days) days. Enrolled patients will receive a fixed dose of 240 mg toripalimab/placebo intravenously on the first day of each course, followed by pemetrexed monotherapy, until, in the judgment of the investigator, the subject is unable to continue to benefit, the disease progresses, intolerable toxicity occurs, the investigator decides, the subject withdraws the informed consent form, or the subject dies. toripalimab is administered every 3 weeks.
27. Subsequent treatment visits: if the end of treatment visit falls within the safety follow-up period, the safety visit does not need to be repeated. Survival follow-up will be performed every 3 months after the last dose (end of treatment) to collect subsequent anti-tumor therapy and interpret SAEs related to the investigational product; for subjects who discontinue treatment for reasons other than disease progression, radiographic assessments should also be performed every 6 weeks for the first 12 months and every 9 weeks after 12 months until disease progression, death, start of new anti-tumor therapy, or withdrawal of consent, whichever occurs first. Thereafter, survival follow-up is performed every 3 months (if applicable).
28. Drug selection for cisplatin versus carboplatin: pemetrexed in combination with cisplatin or carboplatin may be used in patients with non-squamous carcinoma (platinum selection is at the discretion of the investigator), and patients with squamous carcinoma are limited to use nab-paclitaxel plus carboplatin only.
29. Administration of nab-paclitaxel on Day 15 of each cycle is at the discretion of the investigator.

Table 2. Study Schema - Cross-over Phase

	Treatment period[1]																Follow-up period[2]	Survival follow-up[3]
Treatment cycle	1[4]	2	3	4	5	6	7	8	9	10	11	12	13	≥14	End of treatment visit[5]	Safety follow-up visit[6]	Follow-up visit and thereafter	Survival follow-up
Time window (days)	+ 3	± 3	± 3	± 3	± 3	± 3	± 3	± 3	± 3	± 3	± 3	± 3	± 3	± 3	Day study drug is discontinued± 3 days	30 days ± 7 days after last dose	Every 3 months after safety visit ± 3 days	Every 3 months ± 7 days
General assessment																		
Prior and concomitant medications	X	X	X	X	X	X	X	X	X	X	X	X	X	X	X	X		
Subsequent anti-tumor therapy															X	X	X	X
Survival status follow-up																		X
Clinical assessment																		
Collection of adverse events (AEs)[7]	X	X	X	X	X	X	X	X	X	X	X	X	X	X	X	X	X	
Complete physical examination	X																	
Targeted physical examination		X	X	X	X	X	X	X	X	X	X	X	X	X	X	X		
Vital signs and weight	X	X	X	X	X	X	X	X	X	X	X	X	X	X	X	X		
ECOG performance status	X	X	X	X	X	X	X	X	X	X	X	X	X	X	X	X		
12-lead ECG[8]	X	X	X	X	X	X	X	X	X	X	X	X	X	X	X	X		
Echocardiography[9]	X			X			X			X			X	X	X			
Laboratory tests (local laboratory)[10]																		
Hematology	X	X	X	X	X	X	X	X	X	X	X	X	X	X	X	X		

	Treatment period[1]																Follow-up period[2]	Survival follow-up[3]
Treatment cycle	1[4]	2	3	4	5	6	7	8	9	10	11	12	13	≥14	End of treatment visit[5]	Safety follow-up visit[6]	Follow-up visit and thereafter	Survival follow-up
Time window (days)	+ 3	± 3	± 3	± 3	± 3	± 3	± 3	± 3	± 3	± 3	± 3	± 3	± 3	± 3	Day study drug is discontinued± 3 days	30 days ± 7 days after last dose	Every 3 months after safety visit ± 3 days	Every 3 months ± 7 days
Coagulation function	X	X	X	X	X	X	X	X	X	X	X	X	X	X	X	X		
Blood chemistry	X	X	X	X	X	X	X	X	X	X	X	X	X	X	X	X		
Urinalysis	X				X				X				X	X	X	X		
Stool routine + occult blood	X				X				X				X	X	X	X		
Thyroid function	X	X		X		X		X		X		X		X	X	X		
Serum pregnancy test (if applicable)	X[11]														X			
**Efficacy Assessment**																		
Tumor imaging[12]	X			X			X			X			X	X	X	X	X[13]	
**Study Drug Administration**																		
Toripalimab	X	X	X	X	X	X	X	X	X	X	X	X	X	X				

	**Treatment period[1]**																**Follow-up period[2]**	**Survival follow-up[3]**
**Treatment cycle**	**1[4]**	**2**	**3**	**4**	**5**	**6**	**7**	**8**	**9**	**10**	**11**	**12**	**13**	**≥14**	**End of treatment visit[5]**	**Safety follow-up visit[6]**	**Follow-up visit and thereafter**	**Survival follow-up**
**Time window (days):**	**+ 3**	**± 3**	**± 3**	**± 3**	**± 3**	**± 3**	**± 3**	**± 3**	**± 3**	**± 3**	**± 3**	**± 3**	**± 3**	**± 3**	**Day study drug is discontinued ± 3 days**	**30 days ± 7 days after last dose**	**Every 3 months after safety visit ± 3 days**	**Every 3 months ± 7 days**
General assessment																		

	**Treatment period[1]**																**Follow-up period[2]**	**Survival follow-up[3]**
**Treatment cycle**	**1[4]**	**2**	**3**	**4**	**5**	**6**	**7**	**8**	**9**	**10**	**11**	**12**	**13**	**≥14**	**End of treatment visit[5]**	**Safety follow-up visit[6]**	**Follow-up visit and thereafter**	**Survival follow-up**
**Time window (days):**	**+ 3**	**± 3**	**± 3**	**± 3**	**± 3**	**± 3**	**± 3**	**± 3**	**± 3**	**± 3**	**± 3**	**± 3**	**± 3**	**± 3**	**Day study drug is discontinued ± 3 days**	**30 days ± 7 days after last dose**	**Every 3 months after safety visit ± 3 days**	**Every 3 months ± 7 days**
Prior and concomitant medications	X	X	X	X	X	X	X	X	X	X	X	X	X	X	X	X		
Subsequent anti-tumor therapy															X	X	X	X
Survival status																	X	X
Clinical assessment																		
Collection of adverse events (AEs)[7]	X	X	X	X	X	X	X	X	X	X	X	X	X	X	X	X	X	
Complete physical examination	X																	
Targeted physical examination		X	X	X	X	X	X	X	X	X	X	X	X	X	X	X		
Vital signs and weight	X	X	X	X	X	X	X	X	X	X	X	X	X	X	X	X		
ECOG performance status	X	X	X	X	X	X	X	X	X	X	X	X	X	X	X	X		
12-lead ECG[8]	X	X	X	X	X	X	X	X	X	X	X	X	X	X	X	X		
Echocardiography[9]	X			X			X			X			X	X	X			
Laboratory tests (local laboratory)[10]																		
Hematology	X	X	X	X	X	X	X	X	X	X	X	X	X	X	X	X		
Coagulation function	X	X	X	X	X	X	X	X	X	X	X	X	X	X	X	X		
Blood chemistry	X	X	X	X	X	X	X	X	X	X	X	X	X	X	X	X		
Urinalysis	X			X			X			X			X	X	X	X		
Stool routine + occult blood	X			X			X			X			X	X	X	X		
Thyroid function	X	X		X		X		X		X		X		X	X	X		

	**Treatment period[1]**																**Follow-up period[2]**	**Survival follow-up[3]**
**Treatment cycle**	**1[4]**	**2**	**3**	**4**	**5**	**6**	**7**	**8**	**9**	**10**	**11**	**12**	**13**	**≥14**	**End of treatment visit[5]**	**Safety follow-up visit[6]**	**Follow-up visit and thereafter**	**Survival follow-up**
**Time window (days):**	**+ 3**	**± 3**	**± 3**	**± 3**	**± 3**	**± 3**	**± 3**	**± 3**	**± 3**	**± 3**	**± 3**	**± 3**	**± 3**	**± 3**	**Day study drug is discontinued ± 3 days**	**30 days ± 7 days after last dose**	**Every 3 months after safety visit ± 3 days**	**Every 3 months ± 7 days**
Serum pregnancy test (if applicable)[11]	X														X	X		
**Efficacy Assessment**																		
Tumor imaging[12]	X			X			X			X			X	X	X		X[13]	
**Study Drug Administration**																		
toripalimab	X	X	X	X	X	X	X	X	X	X	X	X	X	X				

1. Unless otherwise specified, assessments/examinations are generally performed prior to the first dose of study treatment on Day 1 of each cycle. The treatment cycle is 3 weeks (21 days ± 3 days). If treatment cycles are adjusted, all procedures will be performed by number of cycles instead of weeks of treatment except for imaging, which will be performed once every 9 weeks (63 days ± 7 days), starting from the first dose of trial treatment, regardless of treatment delay.
2. For the convenience of subjects, all follow-up assessments may be performed at the same visit at which imaging is available. Subjects who experience disease progression or initiate a new anti-tumor therapy will enter the survival follow-up directly. Subjects with AEs > Grade 1 will continue to be followed until the AE resolves to Grade 0 - 1 or until start of new anti-tumor therapy, whichever occurs first.
3. Once a subject discontinues radiographic assessments for this protocol (e.g., because of PD or initiation of a new anti-tumor therapy), the subject will enter the survival follow-up period and should be followed up by telephone every 3 months to assess survival status. Post-study treatment and subject responses will also be collected.
4. All procedures and assessments completed at the time of withdrawal from the main study may be used for the start of the crossover phase of the study, if appropriate.
5. A visit should be performed when study drug is discontinued for any reason. If the stop visit occurs 30 days after the last dose of study treatment and concurrently with the mandatory safety follow-up visit, procedures do not need to be repeated.

6. A mandatory safety follow-up visit should be performed for all subjects approximately 30 days after the last dose of trial treatment or before the initiation of a new anti-tumor therapy, whichever occurs first. Subjects with AEs > Grade 1 will continue to be followed until the AE resolves to Grade 0 - 1 or until start of new anti-tumor therapy, whichever occurs first. If the end of treatment visit falls within the safety follow-up period, it can be used as the safety follow-up visit.

7. During the study, all adverse events will be recorded until 30 days after the last dose of study drug or initiation of new anti-tumor therapy, whichever occurs first; AEs suspected to be related to study medication will be collected up to 90 days after the last dose or initiation of new anti-tumor therapy, whichever occurs first.

8. A 12-lead ECG is performed within 14 days prior to the first dose of trial treatment. After Cycle 1, electrocardiograms may be performed 72 hours prior to dosing.

9. Echocardiography is performed within 14 days prior to the first dose of trial treatment. Then, it is conducted once every 6 cycles in and after Cycle 14.

10. Screening laboratory tests will be performed within 14 days prior to the first dose of trial treatment. After Cycle 1, hematology, coagulation function, and blood chemistry (including creatinine clearance) will be performed only within 3 days before dosing, and other laboratory tests within 7 days prior to dosing. Laboratory events must be known prior to dosing. Urinalysis and stool routine: once every 6 cycles from Cycle 14 onwards. T3 or FT3, FT4 and TSH: once every 2 cycles from Cycle 14 onwards;

11. For women of childbearing potential, a serum pregnancy test should be performed within 72 hours prior to the first dose of study treatment. Pregnancy tests will be performed within 7 days prior to treatment and up to the safety follow-up visit 30 days after the last dose in each cycle thereafter.

12. Tumor response assessments are required every 9 weeks (63 ± 7 days) until PD or the subject starts other anti-tumor therapy. Assessment of disease response or progression should be determined by the investigator.

13. Tumor imaging is not required for subjects who initiate other anti-tumor regimens.

4 Study Endpoints

4.1 Efficacy endpoint

4.1.1 Primary efficacy endpoint

The primary efficacy endpoint of this study is the progression-free survival assessed by the investigator (INV-PFS) per Response Evaluation Criteria in Solid Tumors (RECIST 1.1).

PFS is defined as the time from randomization to the first documented PD or death for any cause, whichever occurs first. Subjects who die without reporting any disease progression are considered to experience a PFS event on the date of death.

Censoring rules for PFS will be described in Section 8.6.1 of primary efficacy analysis.

4.1.2 Secondary efficacy endpoints

(1) Overall survival (OS)
OS is defined as the time from randomization to death due to any cause. Subjects who do not die will be censored at the last known alive date.

The 1-year survival rate is defined as the probability that the subject is still alive 1 year after randomization.

(2) Progression-free survival assessed by BIRC (BIRC-PFS) per RECIST 1.1 criteria
BIRC-PFS is defined as the time from randomization to the first documented PD (assessed by BIRC) or death for any cause, whichever occurs first. Subjects who died without reporting any disease progression are considered to experience a PFS event on the date of death. The censoring rules are the same as those for INV-PFS.

(3) PFS rate at 6 months and 1 year
The 6-month and 1-year PFS rates are defined as the proportion of subjects who do not have disease progression (assessed by the investigator or BIRC) or do not die from any cause at 6 months and 1 year after randomization.

(4) Objective response rate (ORR) assessed by investigators or BIRC per RECIST 1.1,
ORR is defined as the proportion of subjects with a best overall response (BOR) of complete response (CR) or partial response (PR).

BOR refers to the best response result recorded from the date of randomization to the date of documented progression according to RECIST 1.1 or the date of initiation of new anti-tumor

therapy, whichever occurs first. Confirmation of response is not required when response evaluation results are CR or PR; stable disease (SD) does not need to meet the minimum duration.

(5) Disease control rate (DCR) assessed by investigators or BIRC per RECIST 1.1
DRC is defined as the proportion of subjects with a BOR of CR, PR, or SD.

(6) Duration of response (DOR) assessed by investigators or BICR per RECIST 1.1
DOR is defined as the time from the first documented response (CR or PR) to the first documented disease progression or death due to any cause, whichever occurs first. For subjects who neither progressed nor died, the censoring treatment for duration of objective response is the same as that in the definition of BIRC-PFS or INV-PFS. This analysis of DOR will be performed only based on subjects who achieve a BOR of CR or PR.

(7) Time to response (TTR) assessed by investigators or BIRC per RECIST 1.1
TTR is defined as the time from randomization to the first documented response (CR or PR). This analysis of TTR will be performed only based on subjects who achieve a BOR of CR or PR.

4.1.3 Exploratory efficacy endpoints

(1) Progression-free survival assessed by the investigator according to iRECIST (INV-iPFS), the INV-iPFS event dates are specified as follows:

- Date of the initial unconfirmed progression of disease (iUPD), if the initial confirmed progression of disease (iCPD) is confirmed at least 4 weeks after the iUPD.
- After iUPD, the iUPD date cannot be used as the iPFS event date if the tumor assessment is iSD, iPR, or iCR;
- If an iUPD is reached but is not subsequently confirmed as an iCPD, the first iUPD date will be recorded as the iPFS event date in the following scenarios:
  - No further tumor assessment (possibly due to subject's refusal to assessment, withdrawal of consent, protocol violation, crossover, death);
  - Or all tumor assessments after the first iUPD are iUPD without iCPD;
  - Or subsequent death;
- If death occurs when the iUPD is not reached, the death date is the iPFS event date;
- In other cases, censoring will be performed at the date of the last tumor assessment,

and further censoring rules are in the same as those for the primary analysis of INV-PFS analysis (see Table 4 for details).

(2) Objective response rate assessed by the investigator per iRECIST criteria (INV-iORR)

Proportion of subjects with a best overall response (iBOR) of complete response (iCR) or partial response (iPR) as assessed by the investigator per iRECIST criteria.

INV-iBOR is defined as the best response recorded from the start of treatment until the iCPD. iPR, iCR, and stable disease (iSD) occurring after iUPD can still be used to determine INV-iBOR.

(3) Disease control rate assessed by the investigator per iRECIST criteria (INV-iDCR)

Proportion of subjects with INV-iBOR of iCR, iPR, iSD as assessed by the investigator per iRECIST criteria.

(4) Duration of response assessed by the investigator per iRECIST criteria (INV-iDOR)

INV-iDOR is defined as the date from the first documented response (iCR or iPR assessed by the investigator per iRECIST) to the occurrence of iPFS event. For subjects without an iPFS event, the censoring rules for iDOR are the same as those for INV-iPFS. This evaluation is performed only in subjects who achieve an iBOR of iCR or iPR.

(5) Time to objective response assessed by the investigator per iRECIST criteria (INV-iTTR)

INV-iTTR is defined as the time from randomization to the first documented response (iCR or iPR assessed by the investigator per iRECIST). The analysis of INV-iTTR will be performed based on subjects who achieve an iBOR of CR or PR.

(6) Progression-free survival in the crossover phase as assessed by the investigator per RECIST 1.1 criteria (INV-PFS2)

INV-PFS2 in the crossover phase is defined as the time from the first dose of toripalimab after crossover to the first documented disease progression or death due to any cause, whichever occurs first, in the crossover phase for subjects randomized to placebo group. Subjects who died without any disease progression reported are considered to experience a PFS event on the date of death. This endpoint is applicable only to subjects who are randomized to the placebo arm and receive toripalimab after crossover.

(7) Objective response rate in the crossover phase as assessed by the investigator per

RECIST 1.1 criteria (INV-ORR2)

INV-ORR2 in the crossover phase is defined as the proportion of subjects with a BOR of CR or PR assessed by investigators according to RECIST 1.1 in the crossover phase.

BOR in the crossover phase refers to the best response recorded from the first dose of toripalimab crossover to the date of documented progression per RECIST 1.1 or the date of initiation of subsequent anti-tumor therapy, whichever occurs first, in the crossover period for subjects randomized to the placebo arm. This endpoint is applicable only to subjects who are randomized to the placebo arm and receive toripalimab after crossover.

(8) Disease control rate in the crossover phase as assessed by the investigator per RECIST 1.1 criteria (INV-DCR2)

INV-DCR2 in the crossover phase is defined as the proportion of subjects with a BOR of CR, PR, or SD assessed by investigators according to RECIST 1.1 in the crossover phase. This endpoint is applicable only to subjects who are randomized to placebo group and receive toripalimab after crossover.

4.2 Safety endpoints

Safety endpoints in this study include the following:

(1) Adverse events (AEs)

- Adverse events: refer to treatment-emergent adverse events that may or may not be causally related to treatment.
- Serious adverse event: refers to any adverse medical event during the study that results in one of the following consequences: 1. death; 2. life-threatening condition; 3. hospitalization or prolonged hospitalization; 4. permanent or significant disability/incapacity; 5. congenital malformation or birth defect; 6. other medically significant events: that may not result in immediate death, life-threatening condition or hospitalization, but may jeopardize the patient based on medical judgment and require medical or surgical intervention to prevent any of the above.
- The relationship of the adverse event to the study drug is judged as "related" or "unrelated".

- Adverse events are judged as Grade 1 (mild), Grade 2 (moderate), Grade 3 (severe), Grade 4 (life-threatening), and Grade 5 (death) in severity according to NCI CTCAE v5.0.

(2) Laboratory tests: including hematology, blood chemistry, coagulation function, thyroid function, urinalysis, stool routine, virology, etc.

(3) Vital signs, physical examination, 12-lead ECG, echocardiography, pulmonary function test

(4) ECOG performance status score

4.3 Immunogenicity

The number and proportion of ADA-positive and ADA-negative patients will be summarized by treatment arm during the treatment and follow-up period.

4.4 Biomarker

The analysis of biomarker parameters other than PD-L1 is not specified in this SAP.

5 Sample Size Estimation

The sample size of the study is based on the primary efficacy endpoint - PFS assessed by investigators per RECIST 1.1. Patients are treated in a 2:1 randomization ratio, and approximately 450 patients (300 in the toripalimab group and 150 in the placebo group) need to be enrolled. The primary efficacy analysis is expected to be performed when 356 PFS events are observed (approximately 27 months after randomization of the first subject), resulting in 85% statistical power to demonstrate the PFS improvement (corresponding hazard ratio HR = 0.7) of toripalimab versus placebo in combination with standard first-line chemotherapy in untreated advanced NSCLC at a one-sided significance level of 0.025. An interim analysis of PFS is planned when 214 PFS events (60% information ratio) are observed.

ORR, assessed by investigator per RECIST 1.1, will be analyzed at the interim and final analyses of PFS. Assuming the ORR in the placebo arm is 30%, the full sample size of 450 patients will provide 99% power to detect an improvement of 20% increase in the toripalimab arm. ORR will be formally tested only if PFS reaches its statistical significance.

The final analysis of OS is planned when 262 deaths are observed (approximately 3 years

after the first subject is randomized), which provide 83% power to detect the OS improvement of HR=0.68 at a one-sided significance level of 0.025. Interim analyese of OS will be performed at the interim and final analyses of PFS, respectively. Details of OS interim analyses are specified in Section 6. OS will be formally tested only if both INV-PFS and ORR reach their statistical significance.

The sample size is calculated based on the following assumptions and using EAST 6.5:

- PFS and OS are exponentially distributed.
- The median PFS is 6 months in the placebo arm.
- The median OS is 15 months in the placebo arm.
- The overall type I error rate of the interim and final analyses of PFS is controlled by Pocock boundary as approximated by the Lan-DeMets alpha spending function.
- The overall type I error rate of the interim and final analyses of OS is controlled by O'Brien-Fleming boundary as approximated by the Lan-DeMets alpha spending function.
- 450 subjects will be recruited over a 15-month period.
- During the first 12 months of observation for PFS and OS events, the drop-out rate is 5% for each arm.

6 Interim Analyses

One interim analysis of PFS is planned at the 60% information (approximately 214 PFS events). The independent statistical support group (iSSG) will perform the analysis and provide a summary report, and the Independent Data Monitoring Committee (iDMC) will review the interim analysis results and provide recommendations to the Sponsor. Details on the formation, responsibilities, and data monitoring plans of iDMC can be referred to the charter.

When the boundary prespecified for the interim analysis of PFS is crossed, iDMC may recommend that the sponsor unblind the study and start the biologic license application (BLA) work based on the results of the interim analysis. In this case, the interim analysis results of the primary efficacy endpoint will be treated as the final efficacy conclusion for this study.

The efficacy boundaries for PFS interim and final analyses are presented in Table 3 below, and they will be updated based on the actual number of PFS events at the interim and final analyses.

Table 3. Efficacy boundary conditions for PFS interim and final analyses using Pocock-type α-spending functions (approximated by Lan-DeMets method)

PFS Analysis	Information Ratio[a]	Number of PFS Events	Efficacy Boundary (One-sided p-value)
Interim analysis	60.1%	214	0.0177
	60.7%	216	0.0179
	61.2%	218	0.0180
	61.8%	220	0.0181
Final analysis	100%	356	0.0128

[a]Information ratio is the proportion of PFS events required for the analysis to the total number of PFS events required for the planned final analysis.

Two interim analyses of OS are planned: one at the interim analysis of PFS and one at the final analysis of PFS. It is expected that approximately 94 and 191 deaths will be observed at the interim and final analyses of PFS, respectively. The boundaries of the OS interim analyses will be calculated based on the actual number of deaths upon each interim analysis. The final analysis of OS will be performed when approximately 262 deaths have been observed in the ITT population. A nominal alpha of 10^{-6} (two-sided) will be assigned to any ad hoc analysis of OS (e.g. as requested by health authories for the purpose of preBLA communication). The overall two-sided alpha level of OS will be controlled at 0.05 minus the total alpha assigned to each ad hoc analyses (i.e., 10^{-6} multiplied by the number of ad hoc analyses).

The interim analysis will include:

- Summary of study populations;
- Summary of demographic and baseline characteristics;
- Analysis of primary efficacy endpoint (INV-PFS)
- Analysis of study secondary efficacy endpoints, including OS, BIRC-PFS, INV-ORR, BIRC-ORR, INV-DCR, BIRC-DCR, as detailed in Sections 4.1.1, 4.1.2, and 8.6;
- Analysis of efficacy endpoints in subgroups of squamous and non-squamous carcinoma, respectively, based on ITT, including INV-PFS, BIRC-PFS, INV-ORR, BIRC-ORR, OS, if applicable;
- Analysis of safety endpoints, as detailed in Section 8.7;

The shells of the tables, listings, figures used for the interim analysis will be provided in a separate document.

6.1 Post-PFS-Interim-Analysis Changes

The planned interim analysis of PFS was performed with the data cutoff on 17 November 2020, when 218 PFS events, as assessed by investigators per RECIST 1.1, had been observed in the ITT population. The PFS result generated at the interim analysis was highly statistically significant and was treated as definitive. The final PFS analysis will then be performed for the descriptive purpose only. The final PFS analysis, as well as the second interim analysis of OS, will be performed when approximately 191 OS events have been observed in the ITT population, which is expected in October of 2021.

7 Analysis Population

Intent-to-treat analysis set (ITT): includes all randomized subjects, and this analysis set will be used as the primary analysis set for efficacy analysis.

Per-protocol analysis set (PPS): includes all ITT patients with valid baseline tumor assessments and no major protocol violations that may potentially affect the efficacy analysis and. Subjects included in the PPS population will be identified based on actual protocol violations prior to database lock for the study. The PPS population will be used for sensitivity analysis of the primary efficacy endpoint, as well as some secondary efficacy endpoints.

Safety analysis set (SS): includes all subjects who have received at least one dose of investigational drug (toripalimab/placebo/chemotherapy).

Crossover analysis set: includes all subjects randomized tothe placebo arm, who crossover after disease progression and receive at least one dose oftoripalimab.
Demographic and baseline data are analyzed based on ITT. Efficacy analysis uses ITT as the primary analysis set, and PPS as the sensitivity analysis set. Safety analysis is performed using safety analysis set (SS). The crossover analysis set is used for efficacy and safety analyses in subjects who cross over.

8 Statistical Analysis

In this study, subjects are allowed to be unblinded after PD according to RECIST 1.1 criteria. If the subject is treated with placebo and meets the requirements of the crossover, toripalimab may be continued. In order to maintain the scientificity of data statistics and reduce bias, the personnel involved in the statistics are divided into blinded statistical analysis team and unblinded statistical analysis team.

The blinded statistical analysis team maintains the blindness during the trial until the SAP is finalized and the database is locked (frozen); this team is responsible for the development of relevant rules and interpretation of results during the statistical analysis, and does not contact subject-level information that may include information on trial randomization. This team consists of the sponsor statistician and statistical procedure personnel. The blinded statistical analysis team cannot log into the EDC or the randomization system directly to browse the study data and will not participate in all discussions regarding the unblinding of subsequent treatment for the subject's disease progression.

The unblinded statistical analysis team is responsible for programming, quality control and generating statistical graphs and tables according to the SAP and programming instructions confirmed by the blinded statistical analysis team, will not participate in the demarcation of populations and interpretation of results, and will be an external statistical analysis team. Prior to database lock (frozen), data management personnel will provide only the subject data to the unblinded statistical analysis team.

8.1 General principles

In this study, SAS 9.4 statistical software is used for statistical analysis.

For continuous variables, descriptive statistics will include mean, median, standard deviation, maximum, and minimum, and if not specified, means and medians will be rounded to numbers with 1 more decimal place than the original data, and standard deviations will have 1 more decimal place than means, but all statistics will have no more than 3 decimal places after rounding. For categorical variables, descriptive statistics will include the number of subjects, percentage (rounded to 1 decimal place), and/or number of events. Percentage will not be calculated if the number of subjects is 0. If not specified, the denominator for the calculation of percentage is the total number of subjects in the respective group of analysis population.

Time-to-event variables will be statistically analyzed using the Kaplan-Meier method; the median, 25% and 75% quartiles and 95% confidence intervals of the time-to-event parameters will be calculated for each treatment group, and Kaplan-Meier curves will be plotted. Differences between groups will be statistically tested using a two-sided log-rank test, a COX proportional hazards model will be used to estimate the hazard ratio (HR) between the two groups, and the corresponding 95% confidence interval is calculated. The decimal place of

time-to-event parameters is specified as follows, and 1 decimal place is retained for survival time, including median time, quartile time, range, and 95% confidence interval. One decimal place is kept for survival rate, 2 decimal places for 95% confidence interval and hazard ratio both, and 3 decimal places for 95% confidence interval.

8.2 Conventions for data processing

8.2.1 Handling of missing data

(1) Lab test values recorded as below (and equal to) or above (and equal to) the range (e.g., < x, ≤ x, > x, ≥ x) will be treated as the range of test value (i.e., = x) when included in the summary of descriptive statistics, but be presented as results recorded in CRF when included in data listing, i.e., "< x", "≤ x", "> x", or "≥ x".

(2) In calculating years of items such as age, if the date is missing, if not specified, the missing date will be imputed as follows:

- If the year, month and day are missing, the date is recorded as missing;
- If only the year is known, the missing month and day will be imputed with July 1;
- If only the day is missing, the missing day will be imputed with 15.

(3) Missing dates for concomitant medication (CM) are imputed as follows:

- Missing of CM start date
  - If the year and month are known, the first day of the known month will be used for imputation;
  - If only the year is known, imputation would be performed using "Jan 1";
  - If the year, month, and day are missing, the CM start date will not be imputed, and the CM needs to be counted as prior medication.
- Missing of CM end date
  - If the year and month are known, the last day of the known month would be used for imputation.
  - If only the year is known, imputation would be performed using "December 31".
  - If the imputed end date is later than the last visit date, the last visit date will be used as the corresponding end date.
  - If the year, month, and day are missing, the CM end date will not be imputed, and the CM needs to be counted as concomitant medication.

(4) Missing dates for AEs will be imputed as follows:

- Missing of AE start date
- If the year and month are known and the year and month are earlier than the year and month of the first dose of the investigational product, the last day of the known month will be used for imputation.
- If the year and month are known and the year and month are equal to the year and month of the first dose of the investigational product, the AE start date will be equal to the date of the first dose of the investigational product (date refers to "MM/DD").
- If the year and month are known and the year and month are later than the year and month of the first dose of the investigational product, the first day of the known month will be used for imputation.
- If only the year is known and the year is earlier than the year of the first dose of the investigational product, imputation will be performed using "December 31".
- If only the year is known and the year is equal to the year of the first dose of the investigational product, the AE start date would be equal to the date of the first dose of the investigational product (date refers to "MM/DD").
- If only the year is known and the year is later than the year of the first dose of the investigational product, "January 1" will be used for imputation.
- If the year, month and day are missing, the date of the first dose of the investigational drug would be used as the corresponding start date.

- Missing of AE end date
  - If the year and month are known, the last day of the known month would be used for imputation.
  - If only the year is known, imputation would be performed using "December 31".
  - If the imputed start date is later than the end date, the end date would be used as the corresponding start date.
  - The remainder would be considered as missing.

(5) When the relationship of an AE to study drug is judged to be missing, the AE will be summarized as a drug-related adverse event.

(6) Missing of efficacy endpoints:

- Handling of missing post-baseline tumor assessment data for objective response rate (ORR): treated as "non-responder"
- Missing of date of death:
  - ➢ If the year, month, and day are missing, the date of death will remain missing. Death will not be treated as an event for PFS assessment; OS will be censored at the last known alive date.
  - ➢ If the month and day are missing, the date of death will remain missing. Death will not be treated as an event for PFS assessment; OS will be censored at the last known alive date.
  - ➢ If the day only is missing, the date of death will be imputed with the first day of the month of the year. Note: if the imputed death date is earlier than or equal to the last known alive date, it should be updated using the last known alive date + 1. Deaths with an imputed date will be used in the derivation of PFS and OS.
- Handling of missing measurement data: no imputation

(7) Missing safety data will not be imputed.

8.2.2 Definition of baseline data

If not specified, baseline data from the double-blind phase of the main study are defined as the most recent non-missing data collected prior to the first dose of study drug. If the subject is not taking study drug after randomization, the date of randomization will be used as the reference date for the baseline definition. Baseline data from the crossover phase are defined as the most recent non-missing data collected before the first dose of study drug in the crossover period in the control group.

8.2.3 Handling of overall tumor response dates

Multiple dates are involved in each the visit tumor assessment per RECIST 1.1 or iRECIST, including the date of imaging used to assess target lesion, non-target lesion, new lesion, and the date of overall tumor response assessment. These dates are often not on the same day, and the date of overall tumor response assessment is usually later than the imaging date.

Therefore, the following rules will be applied when calculating the dates for overall response:

1. When the overall response is "PD", the date of overall tumor response is the earliest date of imaging then used to assess target lesions (assessment of "PD"), non-target

lesions (assessment of "PD"), and new lesions (presence of new lesions);

2. When the overall tumor response is "non-PD", the date of overall response is the latest date of imaging then used to assess target lesions, non-target lesions, and new lesions

8.2.4 Handling of data after cut-off date

Data from the interim analysis will be processed according to data cut-off rules of Junshi, and the detailed rules are shown in Attachment 10.1 Data Cutoff Rules.

8.2.5 Handling of PFS data censoring with new anti-tumor therapy

All new anti-tumor systemic therapies collected in the EDC will result in censoring of PFS events; some of other new anti-tumor therapies, new anti-tumor surgical therapies, and new anti-tumor radiotherapy will not result in censoring of PFS events, such as palliative care. The sponsor's medical department will review relevant data prior to database lock (frozen) and unblinding and determine which therapies will not result in censoring of PFS events.

- Missing of new anti-tumor therapy start date:
  - Missing years, months, and days will be kept missing.
  - Missing months and days will be kept missing.
  - For missing days, the start date of a new anti-tumor therapy will be imputed with the first day of the month of the year.

8.2.6 Handling of use of incorrect stratification factors

Subjects may be randomized using incorrect stratification factors for reasons such as procedural errors, and stratification factors recorded in the interactive web randomization system (IWRS) should be used in the statistical analysis model using stratification factors as covariates according to the ITT principle.

Unless otherwise specified, the actual stratification factors should be used when performing the subgroup analyses.

8.3 Study population

8.3.1 Subject disposition

The number of screened subjects, the number of subjects in intent-to-treat analysis set (ITT),

per-protocol analysis set (PPS), safety analysis set (SS), and crossover analysis set, and the number of subjects unblinded to continue toripalimab monotherapy after disease progression in the toripalimab group will be summarized, as well as the percentage (calculated based on the number of randomized subjects); the summary for the subgroups of squamous and non-squamous carcinoma, PD-L1 expression of TC≥1% and TC<1% will be provided separately.

The number and percentage of subjects who discontinue treatment and study for each reason are summarized, and the primary reason for ending treatment and study and corresponding dates are tabulated at the same time.

Follow-up time for subjects in the study is defined as the time from the start of randomization to the last visit or death without censoring, and the mean, median, standard deviation, maximum and minimum of the follow-up time will be summarized.

Main reasons for subjects to discontinue the treatment can be summarized as: "death", "RECIST 1.1-confirmed disease progression", "iRECIST-confirmed disease progression (iCPD)", "adverse event", "withdrawal from treatment as judged by the investigator due to serious protocol violation", "pregnancy of subject (female)", "withdrawal of consent", "lost to follow-up of subject", "study termination by sponsor", "other circumstances requiring withdrawal from treatment as judged by the investigator", "discontinuation of study drug by subject (or legal representative)", "study drug interruption exceeds the maximum dose interruption time specified in the protocol", "completion of 2 years of toripalimab/placebo treatment (patients in the control group may receive treatment for up to 2 years after crossover to toripalimab treatment)", and "others".

Main reasons for subjects to discontinue the study can be summarized as: "death", "withdrawal of consent", "lost to follow-up", "study termination by the sponsor", and "other reasons".

The primary reasons for screening failure by category will be summarized and a listing of the details of screening failure are tabulated.

8.3.2 Protocol Deviation

The number and percentage of subjects with major protocol deviations in the double-blind and crossover phases will be summarized, and a listing of subjects with all protocol violations will be tabulated.

8.4 Demographic and baseline characteristics

Demographic and baseline characteristics will be analyzed based on ITT and summarized by each treatment group and total, respectively. For continuous variables, descriptive statistics including mean, median, standard deviation, maximum, and minimum will be provided; for categorical variables, descriptive statistics including the number and percentage will be provided.

8.4.1 Demographics

Demographic data include age (years), age group (＜ 65 years vs. ≥ 65 years), gender, ethnicity, height (cm), weight (kg), body mass index (kg/m^2), ECOG score, stratification factor PD-L1 expression (TC ≥ 1% vs TC < 1%), smoking status (frequent vs nonsmoking or little smoking), and pathological type (squamous vs non-squamous). The summary of demographic data for the subgroups of squamous and non-squamous carcinoma, PD-L1 expression of TC≥1% and TC＜1% will be provided separately.

The stratification factors in IWRS will be compared with the information collected by the CRF for consistency.

8.4.2 Smoking history and alcohol use history

The current smoking status, smoking years, average number of cigarettes smoked per day, smoking index, current drinking status, years and frequency of drinking will be summarized.

8.4.3 Medical history of non-small cell lung cancer (NSCLC)

The years from the first pathological diagnosis of non-small cell lung cancer to therandomization date, pathological diagnosis method, pathological type, clinical stage at first diagnosis, tumor TNM stage, clinical stage at enrollment, tumor TNM stage, primary tumor site, metastasis, metastatic site and number of metastatic organs will be summarized.

The summary of medical history of NSCLC for the subgroups of squamous and non-squamous carcinoma, PD-L1 expression of TC≥1% and TC＜1% will be provided separately.

8.4.4 Testing of tumor cell genes and PD-L1

The ALK gene fusion test results, activated EGFR mutation test results and PD-L1 test results of tumor cells collected on CRF will be summarized.

8.4.5 Baseline tumor assessments

Tumor assessments at baseline will be summarized: including number of target lesions, longest diameter of target lesions or sum of short axis of lymph nodes, number of non-target lesions assessed by the investigator per RECIST 1.1.

The summary of baseline tumor assessments for the subgroups of squamous and non-squamous carcinoma, PD-L1 expression of TC≥1% and TC＜1% will be provided separately.

8.4.6 Virology at baseline

The patient number and percentage of virology (HBsAb, HBsAg, HBeAg, HBeAb, HBcAb, HCV antibody, HIV antibody) at baseline will be summarized; HBV DNA levels will be summarized descriptively for HBsAg-positive subjects, HCV RNA levels will be summarized descriptively for subjects with HCV antibody positive. A listing of test results will be tabulated.

8.4.7 Past medical history:

Medical history will be coded per MedDRA 22.0 and will be summarized by system organ class (SOC) and preferred term (PT). A listing of medical history will be provided.

8.4.8 Prior anti-NSCLC treatment history

Prior systemic, radiotherapeutic, surgical, and other therapies for anti-NSCLC will be summarized. A listing of all anti-NSCLC therapies will be provided.

The summary of prior anti-NSCLC treatment history for the subgroups of squamous and non-squamous carcinoma, PD-L1 expression of TC≥1% and TC＜1% will be provided separately.

8.5 Prior/concomitant medications, non-drug therapies, and new anti-tumor therapies

Prior medications are defined as medications with a start date earlier than the first dose of study drug or without a start date, and concomitant medications are defined as medications with an end date on the same day of or later than the first dose of study drug, or without an end date. The analysis of prior/concomitant medications will be performed in the ITT set, and prior/concomitant medications will be coded using the WHO Drug Dictionary B3 March 1, 2019 or later; the number and percentage of subjects will be summarized by the coded

medication.

The number and percentage of subjects who have received each category of concomitant medication will be summarized separately by the purpose of concomitant medication (adverse events, past medical history, disease under this study, prophylactic medication, others).

A listing of prior/concomitant medications will also be tabulated, including drug name, single dose, unit, frequency, route of administration, start and end date, ongoing or not, and indication; prior/concomitant non-drug therapies for subjects will also be tabulated, including treatment name, indication, start and end date, and ongoing or not.

New anti-tumor therapies, including systemic, surgical, radiotherapeutic, and other therapies, will be summarized by double-blind treatment period, monotherapy/crossover period, and end of treatment, and a listing of new anti-tumor therapies will be provided.

8.6 Efficacy analysis

Unless specified, efficacy analyses will be performed based on the ITT set by the treatment group assigned at randomization.

8.6.1 Primary efficacy analysis

Progression-free survival (INV-PFS) assessed by investigator per RECIST 1.1 criteria: Kaplan-Meier method is used to estimate the progression-free survival rate and median PFS at different time points in each treatment group, and to plot Kaplan-Meier curves; the 95% confidence interval for progression-free rate at different time points (6 months and 1 year) is estimated using Greenwood's formula, and the 95% confidence interval for median PFS is estimated using the Brookmeyer-Crowley method with normal approximation after log-log transformation. Differences between groups will be evaluated using the stratified log-rank test. The stratified COX proportional hazards model is used to estimate the hazard ratio (HR) between groups and the corresponding 95% confidence interval, where the tied event times are handled using the exact method. The stratified analyses use the stratification factors collected for randomization, including: (1) PD-L1 tumor expression (TC ≥ 1% vs TC < 1%); (2) smoking status (regular vs nonsmoking or occasional smoking); and (3) pathological type (squamous vs non-squamous carcinoma).

The censoring rules for INV-PFS are presented below in Table 4.

Table 4. Censoring rules for primary analysis and sensitivity analysis of PFS

Scenario	Primary censoring	Sensitivity censoring
Incomplete or no baseline tumor assessments	Censored on the randomization date	Censored on the randomization date
No deaths, no tumor assessments after randomization	Censored on the randomization date	Censored on the randomization date
Death prior to the first tumor assessment	PFS event on the date of death	PFS event on the date of death
Death after the first tumor assessment	PFS event on the date of death	PFS event on the date of death
No PD[1], death, or new anti-tumor therapy	Censored on the date of last evaluable tumor assessment	Censored on the date of last evaluable tumor assessment
No PD or death, but initializing new anti-tumor therapy [2](including crossover from placebo to toripalimab)	Censored on the date of last evaluable tumor assessment prior to new anti-tumor therapy	Recorded as a PFS event on the date starting new anti-tumor therapy
PD or death after ≥ 2 consecutive missing tumor assessments	For patients who have ≥ 2 consecutive missing tumor assessments due to COVID-19, if subsequent tumor assessments become available and there is no immediate disease progression, the subsequent tumor assessments will be used in the PFS analysis; if the subsequent immediate tumor assessment is disease progression, PFS will be censored on the date of last evaluable tumor assessment prior to the missing tumor assessments	Sensitivity censoring rule 1: PFS event on the date of death or PD[3]<br><br>Sensitivity censoring rule 2: Censored on the date of last evaluable tumor assessment prior to the missing tumor assessments[4]

[1]PD is tumor assessment as documented by RECIST 1.1
[2]Before unblinding, medical monitors will identify the new anti-tumor therapy for the PFS calculation.
[3]If there is no PD or death after ≥ 2 consecutive missing tumor assessments, censoring should also be performed on the date of last evaluable tumor assessment
[4]If there is no PD or death after ≥ 2 consecutive missing tumor assessments, censoring should be performed on the date of last evaluable tumor assessment prior to ≥ 2 consecutive missing tumor assessments

The sensitivity censoring rules in Table 4 will be applied to the sensitivity analyses of INV-PFS based on ITT only. Other efficacy analyses will use the primary censoring rules in Table 4 when applicable.

PD-L1 tumor expression (TC ≥ 1% vs TC < 1%), smoking status (regular vs nonsmoking or occasional smoking), and pathological type (squamous vs non-squamous carcinoma) collected on the CRF will be used as factors for stratified analysis based on the primary censoring rules in the ITT set, and this analysis is not required if the randomization stratification factors are identical to those collected on the CRF. The hazard ratio (HR) and its 95% confidence interval estimated by the unstratified COX proportional hazards model will be provided, and the difference of PFS between groups will be evaluated using the unstratified log-rank test.

Subgroup analyses of INV-PFS will be performed, including but not limited to the following variables, providing the number of PFS events, hazard ratio (HR) and its 95% confidence interval estimated by the unstratified COX proportional hazards model, p-value using the unstratified log-rank test, the median PFS of each treatment group estimated using Kaplan-Meier method, and its 95% confidence interval estimated using the Brookmeyer-Crowley method with normal approximation after log-log transformation. Results of subgroup analyses are tabulated and presented with forest plots.

- Age: ＜65 years vs ≥65 years
- Gender: male vs female
- Baseline ECOG status: 0 vs 1
- PD-L1 tumor expression collected in IWRS: TC < 1% vs TC ≥ 1%
- Smoking status collected in IWRS: regular vs nonsmoking or occasional smoking
- Pathological type: squamous vs non-squamous carcinoma
- PD-L1 tumor expression collected on CRF: TC < 1% vs 1% ≤ TC < 50% vs TC ≥ 50%
- Smoking status collected on CRF: regular vs nonsmoking or occasional smoking
- Pathological type collected on CRF: squamous vs non-squamous carcinoma
- Metastasis site: liver metastasis, bone metastasis
- Disease stage: IIIB, IIIC vs IVA vs IVB
- Prior neoadjuvant/adjuvant therapy for Anti-NSCLC: yes vs no
- Prior surgical treatment for anti-NSCLC: yes vs no
- Prior radiotherapy for anti-NSCLC: yes vs no

8.6.2 Secondary efficacy analysis

Overall survival (OS) and 1-year survival rate: OS will be statistically analyzed based on the ITT and PPS sets with the same method as used in the primary efficacy analysis.

The **OS** in subgroups of squamous and non-squamous carcinoma, PD-L1 expression of TC≥1% and TC＜1% will be analyzed separately based on ITT with the same analysis methods as used for INV-PFS, and the 1-year OS rate in each subgroup will be estimated.

Progression-free survival assessed by BIRC per RECIST 1.1(BIRC-PFS): BIRC-PFS will be analyzed based on ITT and PPS sets with an analysis method and censoring rules the same as those in the primary efficacy analysis.

The **BIRC-PFS** in subgroups of subjects with squamous and non-squamous carcinoma, PD-L1 expression of TC≥1% and TC<1% will be statistically analyzed separately based on ITT with a statistical analysis method the same as that of INV-PFS, and the 6-month and 1-year BIRC-PFS rate in each subgroup needs to be estimated.

Objective response rate (ORR) assessed by investigators or BIRC per RECIST 1.1: ORRwill be statistically analyzed in ITT and PPS Sets. The number of subjects achieving the best overall response (CR or PR) will be calculated respectively for each group, and point estimates of ORR will be calculated, and their 95% confidence intervals will be estimated using the exact method (Clopper-Pearson). The nominal P-value for inter-group comparison will be calculated using stratified Cochran-Mantel-Haenszel method, and 95% CI for group difference of ORR will be estimated based on Mantel-Haenszel, where the stratification factors are the same as those of the primary efficacy analysis. The nominal P-value for inter-group comparison will be also calculated using a chi-square test as an unstratified analysis, and the 95% CI for group difference will be estimated using normal approximation. In addition, the number and percentage of subjects achieving the best overall tumor responses (CR, PR, SD, PD, or NE) will be summarized.

ORR for subgroups of patients with squamous and non-squamous cell carcinoma, PD-L1 expression of TC≥1% and TC<1% will be analyzed based on ITT, respectively, and the statistical analysis method is the same as stated above.

Disease control rates assessed by investigators or BIRC per RECIST 1.1: the number and percentage of subjects achieving the best overall response of CR, PR, or SD will be calculated in each group, respectively, and the analysis method is the same as for ORR.

BIRC-DCR and INV-DCR for subgroups of subjects with squamous and non-squamous cell carcinoma, PD-L1 expression of TC≥1% and TC<1% will be analyzed based on ITT, respectively, and the statistical analysis method is the same as above.

Duration of response (DOR) and time to response (TTR) assessed by investigators or BIRC per RECIST 1.1. This analysis will only be performed based on subjects achieving the best overall response of CR or PR, and the analysis method is the same as the primary analysis of INV-PFS.

DOR and TTR for subgroups of patients with squamous and non-squamous cell carcinoma, PD-L1 expression of TC≥1% and TC<1% will be analyzed in the same way as stated above.

Descriptive statistics will be used to summarize the absolute value and percentage of best changes from baseline in tumor size assessed by investigators or BIRC per RECIST 1.1, and the assessment of tumor size is mapped to each scheduled assessment visit by actual assessment date. For specific rules, please refer to Attachment 10.2. Percentage of best changes from baseline in tumor size will be summarized for each subject using a waterfall plot in the order of increasing from the largest tumor to the largest tumor shrinkage, with reference lines of + 20% versus -30% annotted. Tumor size is defined as the sum of the longest axis of non-lymph node target lesion and the shortest axis of lymph node target lesion.

8.6.3 Exploratory efficacy analysis

Progression-free survival (INV-iPFS), duration of response (INV-iDOR), time to objective response (INV-iTTR) assessed by the investigator per iRECIST: the analysis method is the same as the primary analysis of INV-PFS.

Objective response rate (INV-iORR) and disease control rate (INV-iDCR) assessed by the investigator per iRECIST: the analysis method is the same as that of ORR.

Descriptive statistical analysis will be performed for INV-PFS2, INV-ORR2, and INV-DCR2 based on crossover analysis set.

8.7 Safety analysis

Safety analysis will be performed in the SS and crossover analysis sets, respectively, and safety data in the double-blind and cross-over phases of the main study will be analyzed respectively. Safety analysis includes drug exposure, compliance, adverse events, laboratory tests, vital signs, physical examinations, electrocardiograms, and ECOG scores, etc.

8.7.1 Drug exposure and compliance

Descriptive statistics will be used to summarize the exposure of subjects to toripalimab/placebo and various chemotherapy drugs during the study, including:

- Actual number of medication
- Actual duration of medication in weeks
- Actual cumulative dose, which is defined as the sum of actual doses administered in each cycle
- Planned cumulative dose, which is defined as the sum of planned doses administered in each cycle

- Compliance, defined as the actual cumulative dose/planned cumulative dose × 100%, will be statistically described by continuous variables, and categorized by the percentage of compliance (good compliance, ≥ 80% to ≤ 120%, and poor compliance, < 80% or > 120%). The infusion dose for the toripalimab arm is 240 mg × infusions.
- Actual dose intensity (ADI), defined as the actual drug dose received on average per week, $\frac{\text{Actual cumulative dose}}{\text{Actual duration of medication (weeks)+weeks in 1 planned cycle } (e.g.\ 3\ weeks)}$
- Relative dose intensity (RDI): $\frac{\text{Actual dose intensity (ADI)}}{\text{Planned dose intensity}} * 100\%$, where planned dose intensity is defined as the first (first treatment cycle) planned dose divided by the number of weeks (e.g., 3 weeks) of 1 planned cycle

A summary and statistical analysis of drug exposure and compliance are required for subgroups of subjects with squamous and non-squamous cell carcinoma, PD-L1 expression of TC≥1% and TC＜1%.

The medication of subjects will be tabulated by visit.

8.7.2 Adverse event

All adverse events will be coded using the latest version of MedDRA coding system. The number of subjects, number of cases, incidence and severity of adverse events will be summarized by study phase (double-blind and crossover phase of the main study), by treatment group, and system organ class (SOC) and preferred term (PT), respectively. Similar terms will be aggregated, and the aggregation rules will be clarified in the clinical research report.

All adverse events summarized in the summary table are treatment-emergent adverse events (TEAEs) (new or worsening from baseline): TEAEs in the double-blind phase of the main study are defined as all adverse events occurring the first dose to 90 days after the last dose or before the initiation of new systemic antitumor therapy or before the first cross-over with toripalimab in the placebo arm, whichever occurs first; TEAEs in the crossover phase are defined as all adverse events that occur after the first dose of toripalimab crossover to 90 days after the last dose of toripalimab or before the initiation of new systemic anti-tumor therapy (whichever occurs first) after the crossover.

TEAEs will be summarized by the following categories:

- All TEAEs will be summarized and TEAEs occurring in ≥ 5% and ≥ 10% in the double-blind phase of the main study will be summarized, respectively
- TEAEs related to study drug (toripalimab/placebo) will be summarized and TEAEs related to study drug (toripalimab/placebo) occurring in ≥ 5% and ≥ 10% in the double-blind phase of the main study will be summarized, respectively
- TEAEs related to treatment regimen (toripalimab/placebo/any chemotherapy drug)
- TEAEs with CTCAE Grade 3 or above
- TEAEs related to study drug (toripalimab/placebo) with CTCAE Grade 3 or above
- TEAEs related to any drug (toripalimab/placebo/any chemotherapy drug) with CTCAE Grade 3 or above
- Serious adverse events
- Serious adverse events related to study drug (toripalimab/placebo)
- Serious adverse events related to treatment regimen (toripalimab/placebo/any chemotherapy drug)
- Serious adverse events with CTCAE Grade 3 or above
- Serious adverse events related to study drug (toripalimab/placebo) with CTCAE Grade 3 or above
- Serious adverse events related to treatment regimen with CTCAE Grade 3 or above (toripalimab/placebo/any chemotherapy drug)
- TEAEs leading to interruption of study drug (toripalimab/placebo)
- TEAEs related to study drug (toripalimab/placebo) and leading to interruption of study drug (toripalimab/placebo)
- TEAEs leading to interruption of all drugs (toripalimab/placebo/any chemotherapy drug)
- TEAEs leading to interruption of all drugs (toripalimab/placebo/chemotherapy drugs)
- TEAEs leading to permanent discontinuation of study drug (toripalimab/placebo)
- TEAEs related to study drug (toripalimab/placebo) and leading to permanent discontinuation of study drug (toripalimab/placebo)
- TEAEs leading to permanent discontinuation of study drug (toripalimab/placebo/any chemotherapy drug)
- TEAEs leading to permanent discontinuation of all drugs (toripalimab/placebo/chemotherapy drugs)

- TEAEs of special interest
- TEAEs of special interest related to study drug (toripalimab/placebo)
- TEAEs with outcome of death
- TEAEs with outcome of death related to study drug (toripalimab/placebo)
- TEAEs with outcome of death related to treatment regimen (toripalimab/placebo/any chemotherapy drug)
- Immune-related adverse events (irAEs)
- IrAEs with CTCAE Grade 3 or above
- Infusion reactions
- Maximum severity of adverse events

Time to first onset of AEs (SOC), duration of AEs, and time to AE resolution will be summarized using descriptive statistics. Time to first onset of AEs is defined as the time from the first dose to the first onset of each AE. When multiple AEs of the same type occur in the same subject, the earliest AE will be taken. AE duration is defined as the time from onset to end of each AE. When multiple AEs of the same type occur in the same subject, the mean duration is the mean time of the same type of AE, and the cumulative duration is the sum of the same type of AE. When a subject withdraws from the study or dies, the duration of AEs will be cutoff to the date of withdrawal or death, whichever occurrs first. Time to AE response is defined as the time from onset to end of an AE that is converted to resolution without sequelae. When multiple AEs of the same type that are converted to resolution without sequelae occur in the same subject, the time to response is the mean time of the same type of AE.

According the categories described above, AE will be listed. AE ID, AE sequence number, MedDRA system organ class, preferred term, adverse event reported term, start/end date, NCI CTCAE grade, impact on study drug, carboplatin, pemetrexed, albumin-bound paclitaxel, and cisplatin, action taken for AE, outcome, infusion reaction or not, time point of infusion reaction, immune-related adverse events or not, SAE or not, SAE type, causal relationship to toripalimab, relationship to carboplatin, pemetrexed, albumin-bound paclitaxel, and cisplatin will be provided in a listing, including all TEAEs and non-TEAEs that occurr during the study.

When collecting AE information in this study, the same AE will be re-entered into the EDC system when there is a change in toxicity grade, resulting in more AE entries recorded in the

EDC than actual AEs. Therefore, AE entries recorded in the EDC will be pooled to form a derived AE dataset, and an AE entry with the same AE event ID for the same subject will be considered the same AE. Before pooling, the AE entries will be determined whether occur after crossover based on the crossover date, and AE entries that occurr after crossover will be pooled with other AE entries, respectively. The AE summaries and listings described above are based on this derived AE dataset. The variables and calculation rules included in the derived AE dataset are detailed in Attachment 10.3.

8.7.3 Laboratory test

Baseline measurements for laboratory tests (hematology, blood chemistry, coagulation, urinalysis, stool routine, thyroid function) and post-baseline measurements by visit, as well as changes from baseline will be summarized using descriptive statistics.

Shifts from baseline to worst postbaseline clinical assessments will be summarized. The results of applicable laboratory tests will be graded per CTCAE V5.0 (refer to Table 5 for grading criteria); shifts from baseline to the postbaseline worst CTCAE grade will be summarized.

Worst CTCAE grades (all and ≥ level 3) will be summarized for those laboratory tests worsening than baseline.

Table 5. Comparison table of important laboratory tests with CTCAE 5.0 Grade

CTCAE v5.0 Term	Grade 1	Grade 2	Grade 3	Grade 4
Anemia	Hemoglobin < lower limit of normal (LLN)-10.0 g/dL; < LLN-6.2 mmol/L; < LLN-100 g/L	Hemoglobin < 10.0-8.0 g/dL; < 6.2-4.9 mmol/L; < 100-80 g/L	Hemoglobin < 8.0 g/dL; < 4.9 mmol/L; < 80 g/L;	NA
Platelet count decreased	< LLN-75,000/mm^3; < LLN-75.0 x 10^9/L	< 75,000-50,000/mm^3； < 75.0 - 50.0x10^9/L	< 50,000 - 25,000/mm^3； < 50.0 - 25.0 × 10^9/L	< 25,000/mm^3； < 25.0 × 10^9/L
White blood cell count decreased	< LLN-3000/mm^3; < LLN-3.0 × 10^9/L	< 3000-2000/mm^3； < 3.0-2.0 × 10^9/L	< 2000-1000/mm^3； < 2.0 - 1.0 × 10^9/L	< 1000/mm^3； < 1.0 × 10^9/L
Neutrophil count decreased	< LLN-1500/mm^3; < LLN-1.5 × 10^9/L	< 1500 - 1000/mm^3； < 1.5-1.0 × 10^9/L	< 1000 - 500/mm^3； <1.0-0.5 × 10^9/L	< 500/mm^3； < 0.5 × 10^9/L
Lymphocyte count decreased	< LLN-800/mm^3; < LLN-0.8 × 10^9/L	<800～500/mm^3； <0.8～0.5×10^9/L	< 500 - 200/mm^3； < 0.5 - 0.2 × 10^9/L	< 200/mm^3； < 0.2 × 10^9/L

CTCAE v5.0 Term	Grade 1	Grade 2	Grade 3	Grade 4
Creatinine increased	> Upper limit of normal (ULN)-1.5 × ULN	> 1.5-3.0× baseline; > 1.5-3.0× ULN	> 3.0 × baseline; > 3.0-6.0 × ULN	> 6.0 × ULN
Blood bilirubin increased	> 1 - 1.5 × ULN (if baseline is normal); > 1 - 1.5 × baseline (if baseline is abnormal)	> 1.5-3.0× ULN (if baseline is normal); > 1.5-3.0 × baseline (if baseline is abnormal)	> 3 - 10 × ULN (if baseline is normal); > 3 -10 × baseline (if baseline is abnormal)	> 10 × ULN (if baseline is normal); > 10 × baseline (if baseline is abnormal)
Alanine aminotransferase increased	> 1 - 3 × ULN (if baseline is normal); > 1.5 -3.0 × baseline (if baseline is abnormal)	> 3 - 5 × ULN (if baseline is normal); > 3.0 -5.0 × baseline (if baseline is abnormal)	> 5 – 20 × ULN (if baseline is normal); > 5 - 20× baseline (if baseline is abnormal)	> 20 × ULN (if baseline is normal); > 20 × baseline (if baseline is abnormal)
Aspartate aminotransferase increased	> 1 - 3 × ULN (if baseline is normal); > 1.5 -3.0 × baseline (if baseline is abnormal)	> 3 - 5 × ULN (if baseline is normal); > 3.0 -5.0 × baseline (if baseline is abnormal)	> 5-20 × ULN (if baseline is normal); > 5 – 20 × baseline (if baseline is abnormal)	> 20 × ULN (if baseline is normal); > 20 × baseline (if baseline is abnormal)
Creatine phosphokinase increased	> ULN-2.5× ULN	> 2.5-5× ULN	> 5-10× ULN	>10× ULN
Amylase increased	> Upper limit of normal (ULN)-1.5× ULN	> 1.5 - 2 × ULN	> 2 – 5 × ULN	> 5 × ULN
Blood potassium increased	> ULN-5.5 mmol/L	>5.5 - 6.0 mmol/L	> 6.0 - 7.0 mmol/L	> 7.0 mmol/L
Blood potassium decreased	NA	< LLN - 3.0 mmol/L	< 3.0 - 2.5 mmol/L	< 2.5 mmol/L
Blood sodium decreased	< LLN-130 mmol/L	NA	< 130 - 120 mmol/L	< 120 mmol/L
Hypoalbuminemia	< LLN-3 g/dL; < LLN-30 g/L	<3 - 2 g/dL; <30 - 20 g/L	< 2 g/dL; < 20 g/L	NA
Sugar blood indreased	Fasting glucose concentration > ULN-160 mg/dL; fasting glucose concentration > ULN-8.9 mmol/L	Fasting glucose concentration > 160 - 250 mg/dL; fasting glucose concentration > 8.9 - 13.9 mmol/L	Fasting glucose concentration > 250 - 500 mg/dL; fasting glucose concentration > 13.9 - 27.8 mmol/L;	Fasting glucose concentration > 500 mg/dL; fasting glucose concentration > 27.8 mmol/L;
Sugar blood decreased	< LLN-55 mg/dL; < LLN-3.0 mmol/L	< 55 - 40 mg/dL；< 3.0 - 2.2 mmol/L	< 40 - 30 mg/dL；< 2.2 - 1.7mmol/L	< 30 mg/dL； < 1.7 mmol/L；
Activated partial thromboplastin time prolonged	> 1 - 1.5 × ULN	> 1.5 - 2.5 × ULN	> 2.5 × ULN	NA

CTCAE v5.0 Term	Grade 1	Grade 2	Grade 3	Grade 4
Proteinuria	1+ proteinuria; 24-hour urine protein ≥ ULN- < 1.0 g	Adult: 2+ and 3+ proteinuria; 24-hour urine protein 1.0 - 3.5 g;	Adults: proteinuria 4 +, 24-hour urine protein ≥ 3.5 g	NA

Laboratory tests (including scheduled and unscheduled visits) will be tabulated by subject screening No., visit, examination date, test parameters, normal range, test results, unit, and clinical assessment.

8.7.4 Vital signs and physical examination

For vital signs, baseline measurements (blood pressure, respiration, pulse, body temperature, weight) and postbaseline measurements by visit, as well as changes from baseline will be summarized using descriptive statistics.

For physical examination, shifts from baseline to the worst postbaseline clinical assessment will be summarized.

A listing of test results and examination dates for vital signs and physical examination for all visits (including scheduled and unscheduled visits) will be provided.

8.7.5 Electrocardiogram

Baseline measurements for 12-lead electrocardiograms (heart rate, PR interval, QRS interval, QT interval, QTc interval) and post-baseline measurements by visit, as well as changes from baseline will be summarized using descriptive statistics. Shifts from baseline to the worst postbaseline clinical assessment will be summarized.

The number and percentage of subjects who meet the following criteria (Table 6) will be summarized during the treatment.

Table 6. Electrocardiogram scope of special interest

Heart rate (HR)	✓ = < 50 bpm with a decrease of > = 20 bpm from baseline<br>✓ > = 120 bmp with an increase of > = 20 bpm from baseline
PR interval	> = 220 ms with an increase of >= 20 ms from baseline
QRS	>= 120 ms
Absolute value of QTc	✓ > 450 ms and = < 480 ms<br>✓ > 480 ms and = < 500 ms<br>✓ > 500 ms
Change from baseline in QTc	✓ > 30 ms increased from baseline and = < 60 ms<br>✓ > 60 ms increased from baseline

A listing of examination results and dates for electrocardiogram will be provided for all visits, including scheduled and unscheduled visits.

8.7.6 Echocardiography

Baseline data for left ventricular ejection fraction (LVEF) and measurements by treatment cycle after study drug administration, as well as changes from baseline in measurements will be summarized using descriptive statistics. Shifts in the worst clinical assessment before and during treatment (defined as TEAEs during treatment) will be summarized using crossover tables.

A listing of examination results and dates for cardiac echocardiography will be provided for all visits, including scheduled and unscheduled visits.

8.7.7 Pulmonary function test

Baseline data for pulmonary function tests (maximum vital capacity, maximum mid-expiratory flow, maximum peak expiratory flow rate, maximum expiratory volume per second, diffusing capacity for carbon monoxide, oxygen saturation) and measurements by treatment cycle after study drug administration, as well as changes from baseline in measurements will be summarized using descriptive statistics. Shifts in the worst clinical assessment before and during treatment (defined as TEAEs during treatment) will be summarized using crossover tables.

A listing of pulmonary function test results and dates for pulmonary function will be provided for for all visits, including scheduled and unscheduled visits.

8.7.8 ECOG performance status

ECOG score at baseline and postbaseline will be summarized by visit, and shifts from baseline to the worst postbaseline ECOG score will be summarized.

A listing of ECOG score and examination dates will be provided for all visits, including scheduled and unscheduled visits.

9 References

None

10 Attachments

10.1 Cut-off date rule for SDTM

Domain*	Cut-off date Rule	Timing Variables and Imputation Rule
DM	If **RFICDTC** is after the cut-off date, these subjects will be removed from DM and all other domains.	**1) RFICDTC**<br>➢ Set **ARM/ARMCD/ACTARM/ACTARMCD** to missing if RFICDTC is before cut-off date but randomization date is after cut-off date, set **ARMNRS** to 'not randomized'<br>➢ Set **ACTARM/ACTARMCD** to missing and **ARMNRS** to 'not administered' if randomization date is before cut-off date and first dosing date is after cut-off date<br>**2) RFSTDTC/RFXSTDTC**<br>➢ Set to missing if RFSTDTC/RFXSTDTC is after cut-off date<br>**3) RFENDTC/RFXENDTC**<br>➢ Set to missing if RFSTDTC is after cut-off date<br>➢ Set to cut-off date if RFSTDTC is before cut-off date and RFENDTC is after cut-off date<br>**4) RFPENDTC**<br>➢ Set to cut-off date if RFPENDTC is after cut-off date<br>**5) DTHDTC**<br>➢ Set to missing if DTHDTC is after cut-off date<br>➢ Set **DTHFL** to missing if DTHDTC is after the cut-off date
AE, COAE	If **AESTDTC** is after the cut-off date, remove these records from corresponding domains.	For any case whose **AESTDTC** before the cut-off date and **AEENDTC** after the cut-off date:<br>**1) AESTDTC**<br>**2) AEENDTC**<br>➢ Set **AEENDTC/AEENDY** to missing<br>➢ Set **AEENRF** to 'ongoing'<br>➢ Set **AEOUT** to 'not recovered/ persistent' (for each study, please refer to CRF)
EX, EC, SV, SE	If **xxSTDTC** is after the cut-off date, remove these records from corresponding domains.	**1) xxSTDTC**<br>**2) xxENDTC**<br>➢ Set to cut-off date if xxENDTC is after cut-off date<br>➢ **xxENDY** should be recalculated based on xxENDTC

CM, PR, COPR, CE, MH, SU	If **xxSTDTC** is after the cut-off date , remove these records from corresponding domains.	**1) xxSTDTC**<br>**2) xxENDTC**<br>➢ Set **xxENDTC/xxENDY** to missing if xxENDTC is after cut-off date<br>➢ Except for PR, set **xxENRF** to 'ongoing' if xxENDTC is after cut-off date<br>➢ For PR domain, set **PRENTPT** to cut-off date and **PRENRTPT** to 'ongoing' if PRENDTC is after cut-off date
DS, DV	If **xxSTDTC** is after the cut-off date remove these records from corresponding domains.	**xxSTDTC**
DD, EG, IE, IS, FA, LB, COLB, MB, MI, MO, CV, PE, PF, QS, RS, SS, TR, TU, VS, XO, XU	If **xxDTC** is after the cut-off date , remove these records from corresponding domains.	**xxDTC**

*For any domain, if timing variable is not collected, all records should be kept for analysis.

10.2 Mapping rules for tumor assessment visits

Time	Integrated Analyses		
	**Target Day**	**Range**	**Selection Criteria**
Baseline	1	<=1	Closest to Day 1, prior to Day 2
Week 7	43	2 - 64	Closest to target date, later if ties
Week 13	85	65 - 106	Closest to target date, later if ties
Week 19	127	107 - 148	Closest to target date, later if ties
Week 25	169	149 - 190	Closest to target date, later if ties
Week 31	211	191 - 232	Closest to target date, later if ties
Week 37	253	233 - 274	Closest to target date, later if ties
Week 43	295	275 - 316	Closest to target date, later if ties
Week 49	337	317 - 358	Closest to target date, later if ties
Week 55	379	359 - 410	Closest to target date, later if ties
Week 64	442	411 - 473	Closest to target date, later if ties
Week 73	505	474 - 536	Closest to target date, later if ties
Week 82	568	537 - 599	Closest to target date, later if ties
Week 91	631	600 - 662	Closest to target date, later if ties
Week 100	694	663 - 725	Closest to target date, later if ties
Week 109	757	>=726	Closest to target date, later if ties

10.3 Calculation rule for derived AE dataset

Derived variables	Calculation rules
Derived AE event #	The same as the AE event # in the EDC dataset
Derived AE sequence number	The sequence numbers of all AEs under the same "AE event #" are merged in an ascending order of "start date", and separated by semicolons.
Derived AE name	The same as the AE name in the EDC dataset
Derived start date	The first "start date" under the same "AE event #"
Derived ongoing or not	The AE with same "AE event #" is "ongoing or not" on the last (latest) "start date"
Derived end date	The "end date" corresponding to the last (latest) "start date" under the same "AE event #"
Derived initial severity (NCI CTCAE 5.0)	The "severity (NCI CTCAE 5.0)" under the same "AE event #" on the first (earliest) "start date"
Derived highest severity (NCI CTCAE 5.0)	The highest "severity (NCI CTCAE 5.0)" under the same "AE event #"
Derived start date of AE with the highest severity (DD/MMMYYY)	The "start date" of the AE reaching the highest "severity (NCI CTCAE 5.0)" for the first time under the same "AE event #"
Derived effect on study drug (JS001)	The highest degree of effect under the same "AE Event #". The degree of effect decreases as follows: permanent discontinuation > drug interruption > dose unchanged > not applicable
Derived effect on study drug (carboplatin)	The highest degree of effect under the same "AE Event #". The degree of effect decreases as follows: permanent discontinuation > dose reduction > drug interruption > dose unchanged > not applicable
Derived effect on study drug (pemetrexed)	The highest degree of effect under the same "AE Event #". The degree of effect decreases as follows: permanent discontinuation > dose reduction > drug interruption > dose unchanged > not applicable
Derived effect on study drug (nab-paclitaxel)	The highest degree of effect under the same "AE Event #". The degree of effect decreases as follows: permanent discontinuation > dose reduction > drug interruption > dose unchanged > not applicable
Derived effect on study drug (cisplatin)	The highest degree of effect under the same "AE Event #". The degree of effect decreases as follows: permanent discontinuation > dose reduction > drug interruption > dose unchanged > not applicable
Derived actions taken on AEs – concomitant medications	If at least one of AEs with the same "AE event #" is selected as "concomitant medication", it will be calculated as "Yes"; otherwise, it will be calculated as "No"
Derived actions taken on AEs – concomitant non-drug therapy	If at least one of AEs with the same "AE event #" is selected as "concomitant non-drug therapy", it will be calculated as "Yes"; otherwise, it will be calculated as "No"
Derived actions taken on AEs – others	If at least one of AEs with the same "AE event #" is selected as "others", it will be calculated as "Yes"; otherwise, it will be calculated as "No"
Derived actions taken on AEs – others, please specify	All non-empty instructions under the same "AE event #" are merged after removing duplicates in an ascending order of "start date", and separated by semicolons.

Derived variables	Calculation rules
Derived leading to end of treatment or not	If at least one of AEs with the same "AE event #" is recorded as "Yes", it will be calculated as "Yes"; otherwise, it will be calculated as "No"
Derived outcome	The outcome corresponding to the AE with the same "AE event #" on the last (latest) "start date"
Derived infusion reaction or not	If the values expressing whether AEs with the same "AE event #" are caused by infusion reaction should be consistent, the derived variable is equal to the raw data
Derived onset time of infusion reaction	If the values expressing the onset time of infusion reaction under the same "AE event #" should be consistent, the derived variable is equal to the raw data
Derived irAE or not	If at least one of AEs with the same "AE event #" is recorded as "Yes" under the item of "irAE or not", it will be calculated as "Yes"; otherwise, it will be calculated as "No"
Derived AESI or not	If at least one of AEs with the same "AE event #" is recorded as "Yes" under the item of "AESI or not ", it will be calculated as "Yes"; otherwise, it will be calculated as "No"
Derived SAE or not	If at least one of AEs with the same "AE event #" is recorded as "Yes" under the item of "SAE or not", it will be calculated as "Yes"; otherwise, it will be calculated as "No"
Derived SAE type – leading to death	If at least one of SAEs with the same "AE event #" is recorded as "leading to death", it will be calculated as "Yes"; otherwise, it will be calculated as "No"
Derived SAE type – life threatening	If at least one of SAEs with the same "AE event #" is recorded as "life threatening", it will be calculated as "Yes"; otherwise, it will be calculated as "No"
Derived SAE type – requiring hospitalization or prolongation of hospitalization	If at least one of SAEs with the same "AE event #" is recorded as "requiring hospitalization or prolongation of hospitalization", it will be calculated as "Yes"; otherwise, it will be calculated as "No"
Derived SAE type – leading to permanent or severe disability/dysfunction	If at least one of SAEs with the same "AE event #" is recorded as "leading to permanent or severe disability/dysfunction", it will be calculated as "Yes"; otherwise, it will be calculated as "No"
Derived SAE type – resulting in congenital anomalies or birth defects	If at least one of SAEs with the same "AE event #" is recorded as "resulting in congenital anomalies or birth defects", it will be calculated as "Yes"; otherwise, it will be calculated as "No"
Derived SAE type – important medical event	If at least one of SAEs with the same "AE event #" is recorded as "important medical event", it will be calculated as "Yes"; otherwise, it will be calculated as "No"
Derived date of meeting the criteria of an SAE	This variable will be added to CRF6.0. If the recorded values of this variable should be consistent after the AEs with the same "AE event #" meets the criteria of an SAE, the derived variable is equal to the raw data.
Derived cumulative length of stay	Cumulative length of stay under the same "AE

Derived variables	Calculation rules
	event #". Note: duplicate records should be removed.
Derived causality to study drug (toripalimab)	− If at least 1 record about "causality to toripalimab " under the same "AE event #" is documented as "related to", it is calculated as "related to";<br>− otherwise, it is calculated as "not related to";
Derived causality to study drug (carboplatin)	− If at least 1 record about "causality to carboplatin" under the same "AE event #" is documented as "related to", it is calculated as "related to";<br>− otherwise, it is calculated as "not related to";<br>− If all records under the same "AE event #" are documented as "not applicable", it is calculated as "not applicable".
Derived causality to study drug (pemetrexed)	− If at least 1 record about "causality to pemetrexed" under the same "AE event #" is documented as "related to", it is calculated as "related to";<br>− otherwise, it is calculated as "not related to";<br>− If all records under the same "AE event #" are documented as "not applicable", it is calculated as "not applicable".
Derived causality to study drug (nab-paclitaxel)	− If at least 1 record about "causality to nab-paclitaxel" under the same "AE event #" is documented as "related to", it is calculated as "related to";<br>− otherwise, it is calculated as "not related to";<br>− If all records under the same "AE event #" are documented as "not applicable", it is calculated as "not applicable".
Derived causality to study drug (cisplatin)	− If at least 1 record about "causality to cisplatin" under the same "AE event #" is documented as "related to", it is calculated as "related to";<br>− otherwise, it is calculated as "not related to";<br>− If all records under the same "AE event #" are documented as "not applicable", it is calculated as "not applicable".
Derived correlation with others except for study drug – related to underlying disease	If at least 1 record about "correlation with others except for study drug" under the same "AE event #" is documented as "related to underlying disease", it will be calculated as "Yes"; otherwise, it will be calculated as "No"
Derived correlation with others except for study drug – related to concomitant disease	If at least 1 record about "correlation with others except for study drug" under the same "AE event #" is documented as "related to concomitant disease", it will be calculated as "Yes"; otherwise, it will be calculated as "No"
Derived correlation with others except for study drug – related to concomitant medications	If at least 1 record about "correlation with others except for study drug" under the same "AE event #" is documented as "related to concomitant medications", it will be calculated as "Yes"; otherwise, it will be calculated as "No"
Derived correlation with others except for study drug – related to operating procedures	If at least 1 record about "correlation with others except for study drug" under the same "AE event #"

Derived variables	Calculation rules
	is documented as "related to operating procedures", it will be calculated as "Yes"; otherwise, it will be calculated as "No"
Derived correlation with others except for study drug – others	If at least 1 record about "correlation with others except for study drug" under the same "AE event #" is documented as "others", it will be calculated as "Yes"; otherwise, it will be calculated as "No"
Derived correlation with others except for study drug – other, please specify	The AEs with the same "AE event #" are merged after removing duplicates in an ascending order of "start date", and separated by semicolons.

11 Version Control

Changes of SAP Version 2.0		
**Chapter number and Title**	**Description of the Changes**	**Reasons**
Cover page	1. "Version number" was changed to "2.0";<br>2. The "Version Date" was changed to " August 17, 2021".	SAP version change
Statistical Analysis Plan Signature Page	1. "Version No./Date" was changed to "2.0/ August 17, 2021"	SAP version change
The whole document	Changed “primary analysis” to “final analysis” for PFS	To improve accuracy and consistency.
Section 6	1. Added “The final analysis of OS will be performed when approximately 262 deaths have been observed in the ITT population. A norminal alpha of 10^{-6} (two-sided) will be assigned to each ad hoc analysis of OS (e.g. as requested by health authories for the purpose of preBLA communication). The overall two-sided alpha level of OS will be controlled at 0.05 minus the total alpha assigned to all ad hoc analyses (i.e., 10^{-6} multiplied by the number of ad hoc analyses).”<br><br>2. Added a sub section about the changes made to the final PFS analysis as follows:<br>6.1 Post-PFS-Interim-Analysis Changes<br>The planned interim analysis of PFS was performed with the data cutoff on 17 November 2020, when 218 PFS events, as assessed by investigators per RECIST 1.1, had been observed in the ITT population. The PFS result generated at the interim analysis was highly statistically significant and was treated as definitive. The final PFS analysis will then be performed for the descriptive purpose only. The final PFS analysis, as well as the second interim analysis of OS, will be performed when approximately 191 OS events have been observed in the ITT population, which is expected in October of 2021.	To protect the data integrity, norminal alpha should be assigned to any ad hoc analysis of OS.<br><br>The timing of the final PFS analysis was adjusted considering it will be performed for the description purpose only.
Section 8.2.6	1.Added “Unless otherwise specified, the actual stratification factors should be used when performing the subgroup analyses.”	To clarify how to handle incorrect stratification factors in the subgroup analysis
Section 8.3.1	1. Added the summary of the subject disposition for the subgroup of PD-L1 expression of TC≥1% and TC<1%.	To add the subgroup analysis as needed
Section 8.4.1	1. The age group of ≤65 years vs. >65 years was changed to < 65 years vs. ≥ 65 years;<br>2. Added “The summary of demographic data for the subgroups of squamous and non-squamous carcinoma, PD-L1 expression of TC≥1% and TC<1% will be	To update the age subgroups<br><br>To add subgroup summary for the

Changes of SAP Version 2.0		
**Chapter number and Title**	**Description of the Changes**	**Reasons**
	provided separately.”	demographic data as needed
Section 8.4.3, 8.4.5 and 8.4.8	Added summary of Medical history of non-small cell lung cancer, Baseline tumor assessments and Prior anti-NSCLC treatment history for the subgroups of squamous and non-squamous carcinoma, PD-L1 expression of TC ≥1% and TC＜1% will be provided separately.	To add more subgroup summary as needed
Section 8.6.1	1. Changes in Table 4. Censoring rules for primary analysis and sensitivity analysis of PFS<br>1.1. “No PD, death, or initiation of new anti-tumor therapy (or crossover from placebo to toripalimab)” was updated to “No PD or death, but initializing new anti-tumor therapy (including crossover from placebo to toripalimab)”<br>1.2. The primary censoring rule for the scenario of “PD or death after ≥ 2 consecutive missing tumor assessments” was changed to “For patients who have ≥ 2 consecutive missing tumor assessments due to COVID-19, if subsequent tumor assessments become available and there is no immediate disease progression, the subsequent tumor assessments will be used in the PFS analysis; if the subsequent immediate tumor assessment is disease progression, PFS will be censored on the date of last evaluable tumor assessment prior to ≥ 2 consecutive missing tumor assessments”<br>1.3. Added one additional sensitivity censoring rule for the scenario of “PD or death after ≥ 2 consecutive missing tumor assessments”: censored on the date of last evaluable tumor assessment prior to ≥ 2 consecutive missing tumor assessments.<br>1.4 Added “[2] Before unblinding, medical monitors will identify the new anti-tumor therapy for the PFS calculation.” to the footnotes of the table<br>1.5 Updated the footnotes of the table as<br>[3] If there is no PD or death after ≥ 2 consecutive missing tumor assessments, censoring should also be performed on the date of last evaluable tumor assessment<br>[4] If there is no PD or death after ≥ 2 consecutive missing tumor assessments, censoring should be performed on the date of last evaluable tumor	To improve the accurary and align the censor rules for the impact of COVID-19 across studies of Shanghai Junshi Biosciences Co., Ltd.;<br><br>To clarify when and how to identify the new anti-tumor therapy for the PFS calculation;<br><br>To update age subgroups.

Changes of SAP Version 2.0		
**Chapter number and Title**	**Description of the Changes**	**Reasons**
	assessment prior to ≥ 2 consecutive missing tumor assessments<br>2 Changes in the text<br>2.1 The age group of ≤65years vs. >65years was changed to <65 years vs. ≥65 years	
Section 8.6.2	1. Added analysis of OS, BIRC-PFS, BIRC and INV ORR, BIRC and INV DCR, BIRC and INV DOR, BIRC and INV TTR for the subgroups of PD-L1 expression of TC≥1% and TC<1%.	To add subgroup anlaysis for PD-L1 expression
Section 8.7.1	1. Added summary of drug exposure and compliance for the subgroups of PD-L1 expression of TC≥1% and TC <1%.	To add subgroup summary for PD-L1 expression
Section 8.7.2	1. Added “Similar terms will be aggregated, and the aggregation rules will be clarified in the clinical research report.”<br>2. Reworded the definition of TEAE<br>3. Added “According the categories described above, AE will be listed.”	To clarify that AE term aggregation was applied<br><br>To reword the TEAE definition<br><br>To request more listings for AE
Section 8.7.3	1. Added “Worst CTCAE grades (all and ≥ level 3) will be summarized for those laboratory tests worsening than baseline.”	To add more summary for lab tests as needed
Section 8.7.5	1. Updated QTcF to QTc;<br>2. Deleted the correction formula of QTcF	RR interval was not collected and QTcF could not be derived
